# Supplementary material for: Be ExPeRT (Behavioral Health Expansion in Pediatric Residency Training): A Case-Based Seminar
Source: MedEdPORTAL. 2023 Aug 1;19:11326. doi: 10.15766/mep_2374-8265.11326 (PMC10392710; doi:10.15766/mep_2374-8265.11326)

## Slide 1
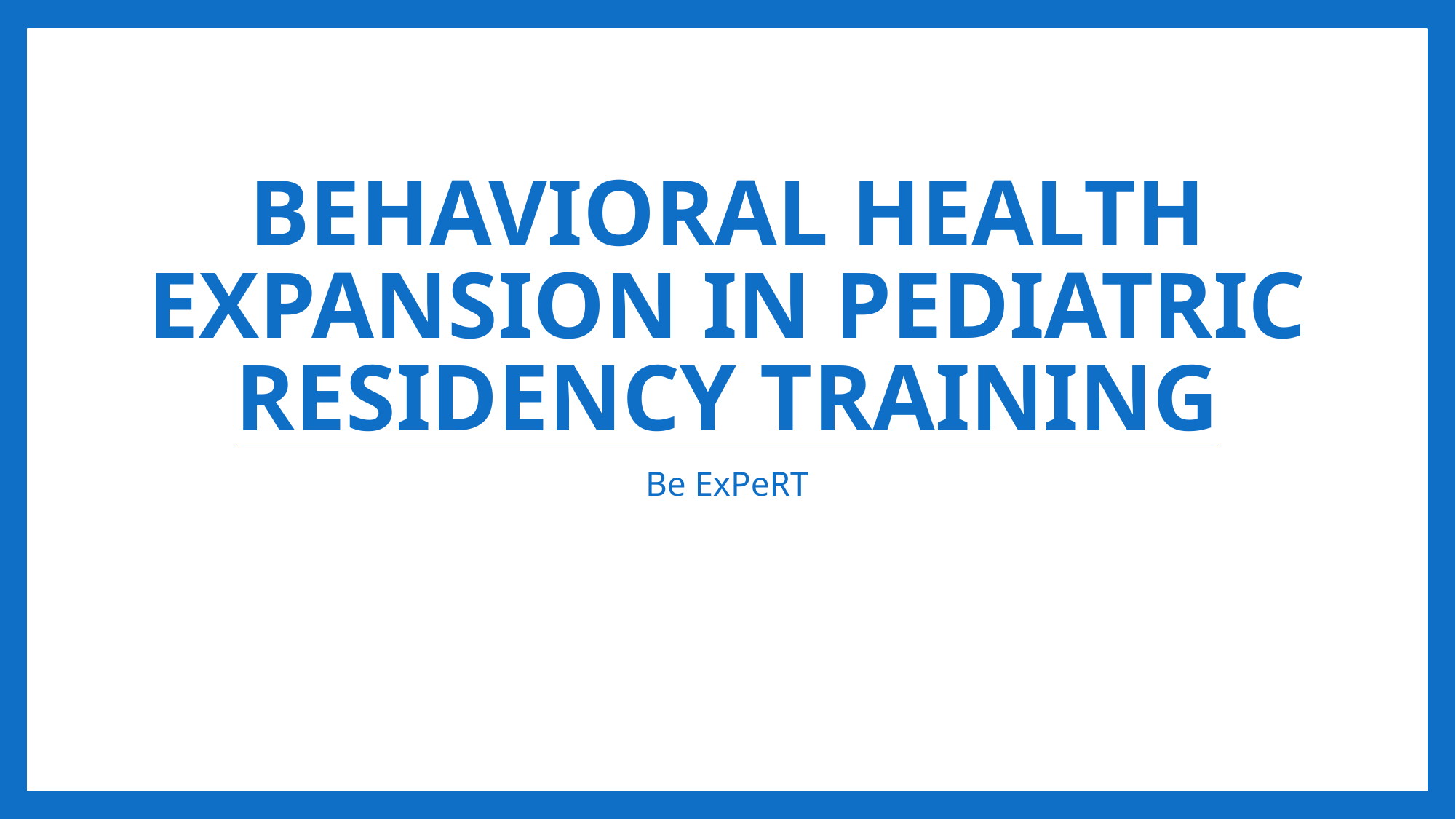

# Behavioral health expansion in Pediatric Residency Training
Be ExPeRT

## Slide 2
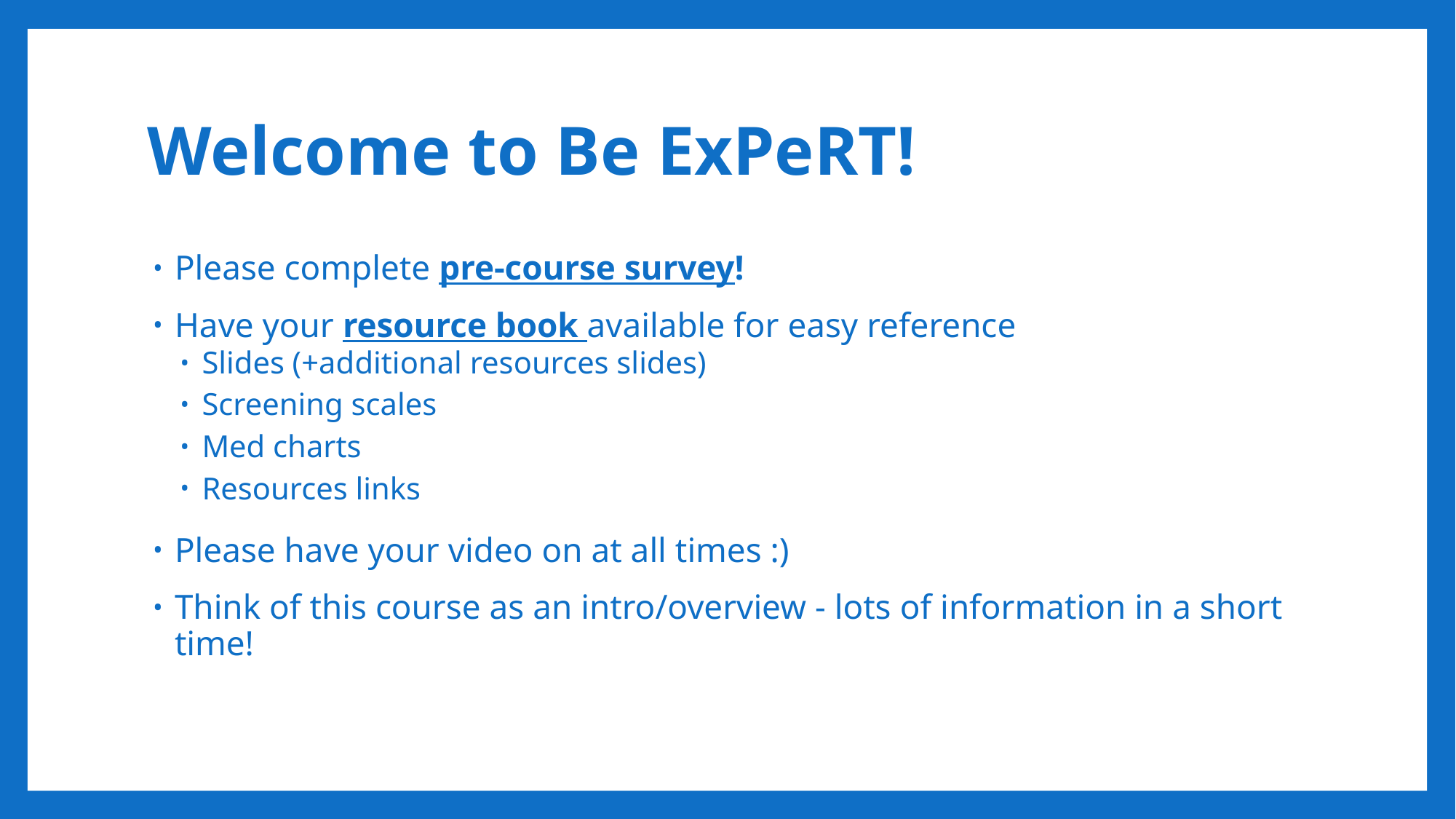

# Welcome to Be ExPeRT!
Please complete pre-course survey!
Have your resource book available for easy reference
Slides (+additional resources slides)
Screening scales
Med charts
Resources links
Please have your video on at all times :)
Think of this course as an intro/overview - lots of information in a short time!

## Slide 3
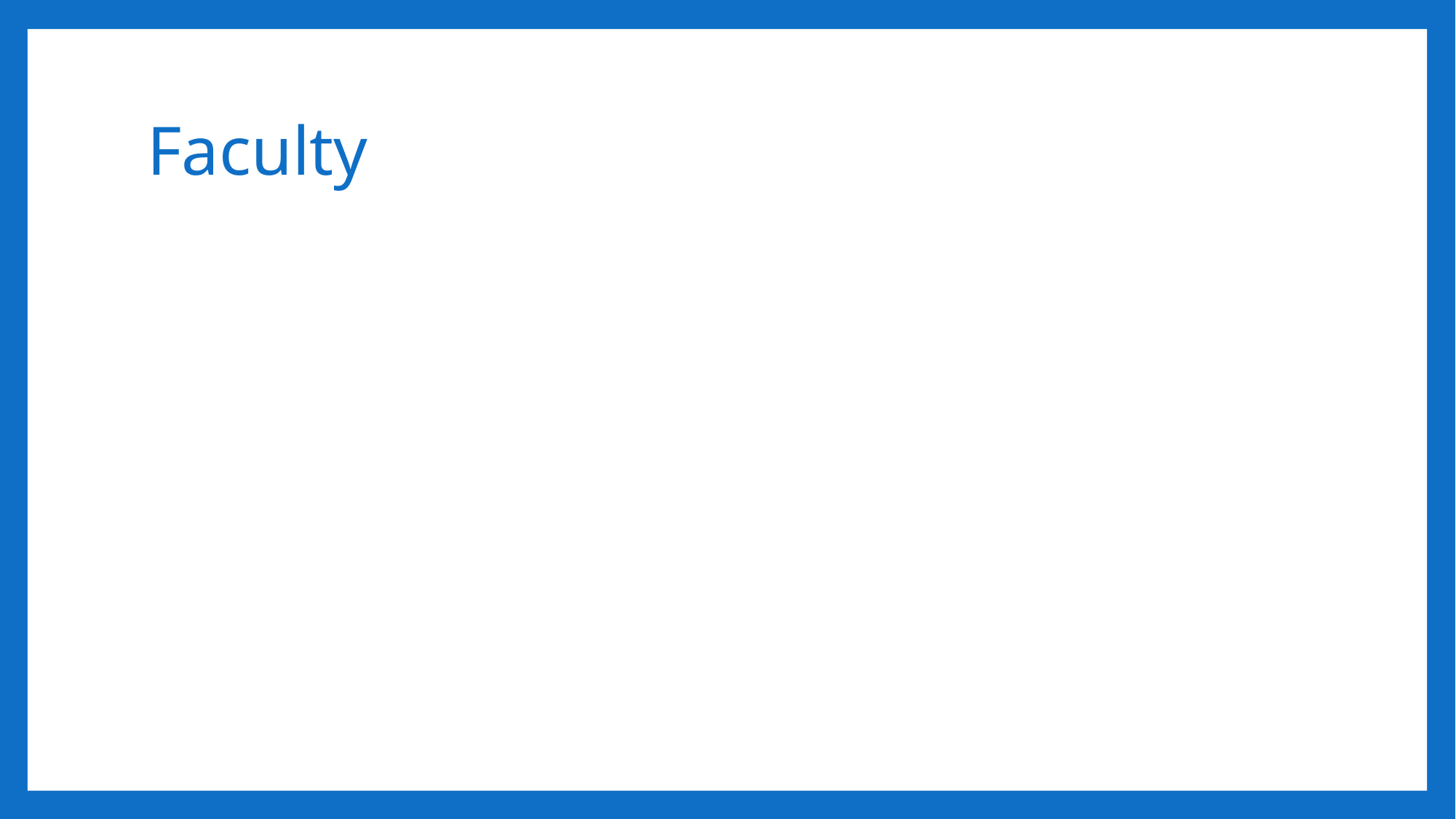

# Faculty

## Slide 4
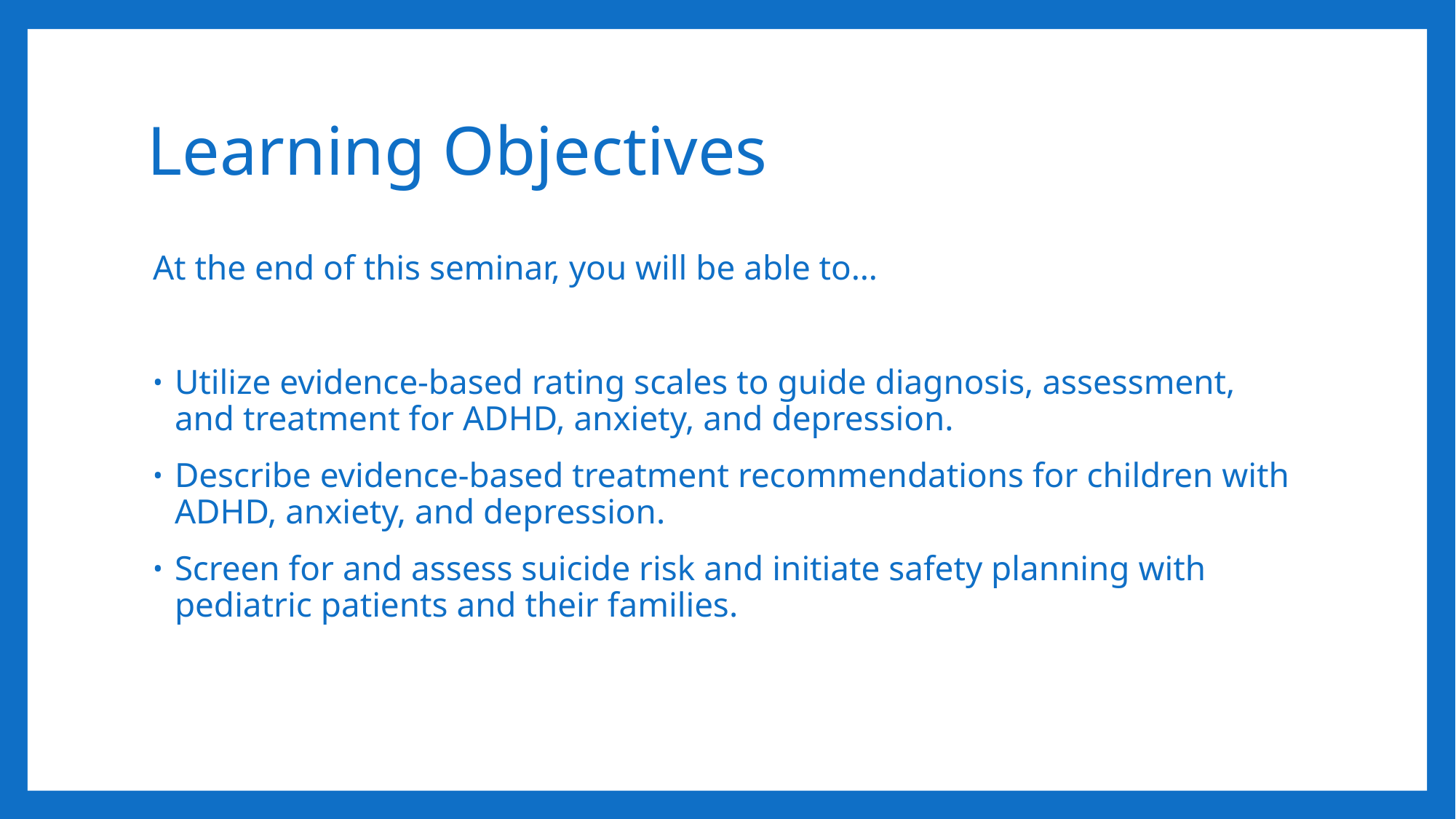

# Learning Objectives
At the end of this seminar, you will be able to…
Utilize evidence-based rating scales to guide diagnosis, assessment, and treatment for ADHD, anxiety, and depression.
Describe evidence-based treatment recommendations for children with ADHD, anxiety, and depression.
Screen for and assess suicide risk and initiate safety planning with pediatric patients and their families.

## Slide 5
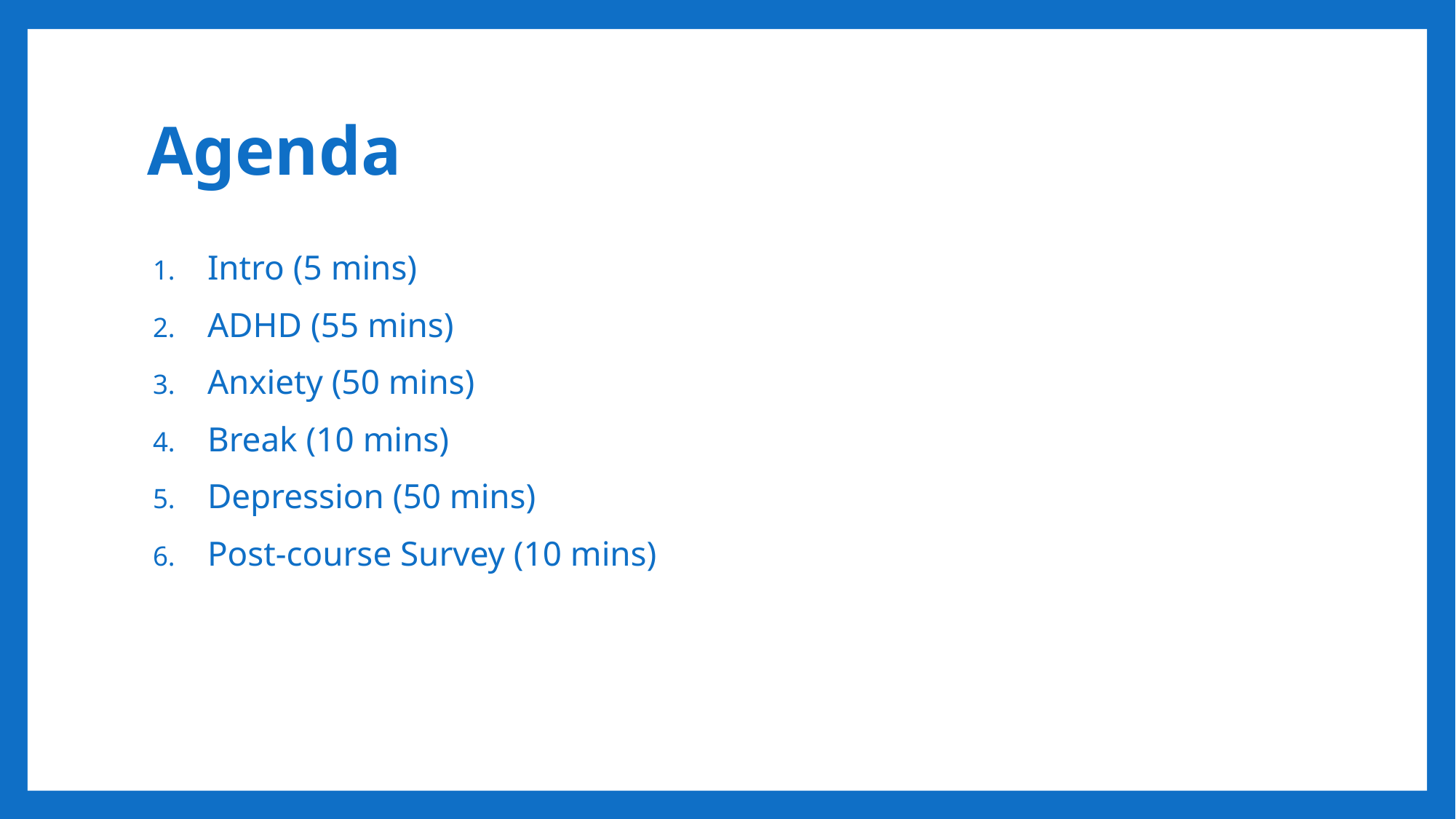

# Agenda
Intro (5 mins)
ADHD (55 mins)
Anxiety (50 mins)
Break (10 mins)
Depression (50 mins)
Post-course Survey (10 mins)

## Slide 6
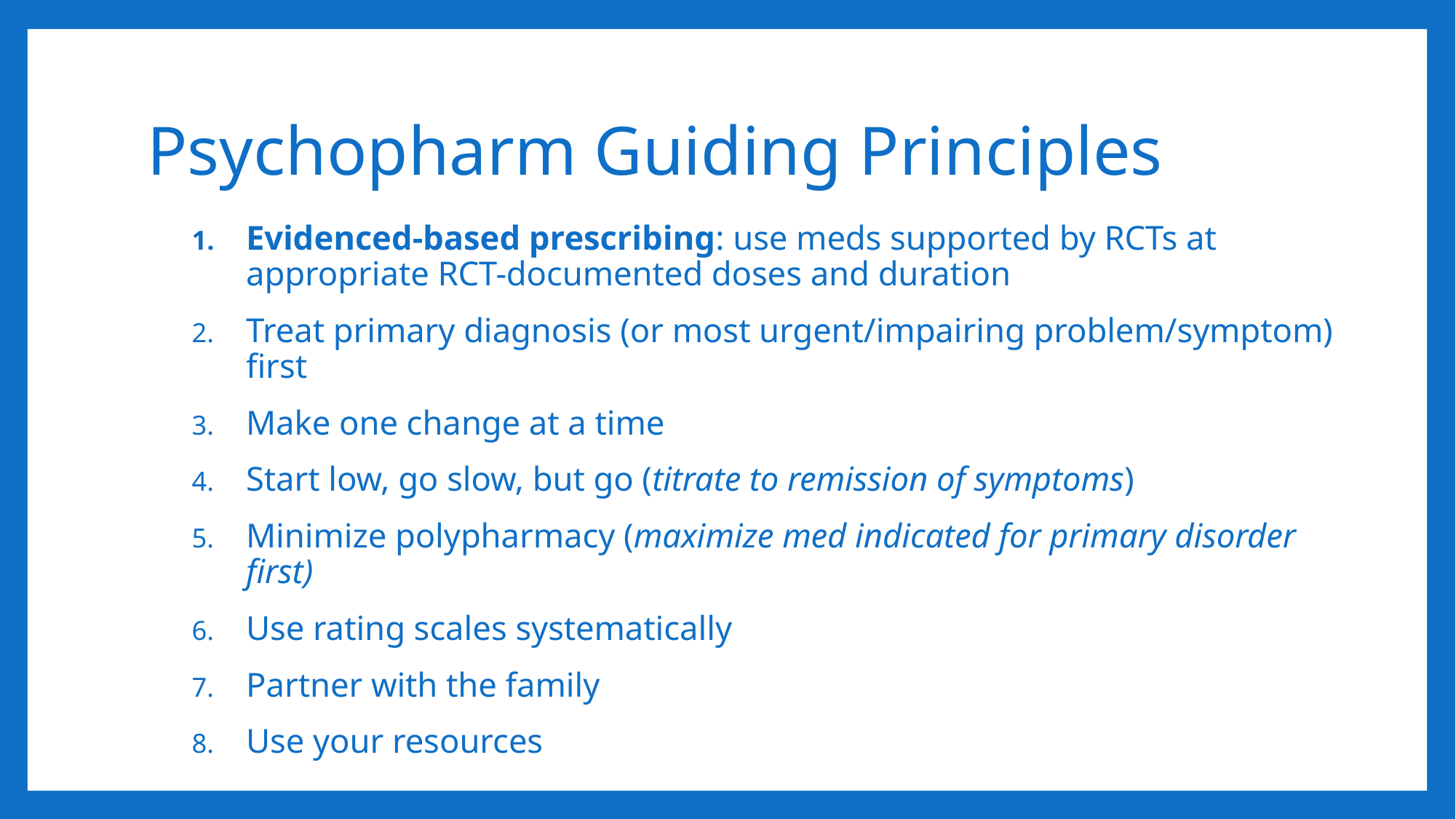

# Psychopharm Guiding Principles
Evidenced-based prescribing: use meds supported by RCTs at appropriate RCT-documented doses and duration
Treat primary diagnosis (or most urgent/impairing problem/symptom) first
Make one change at a time
Start low, go slow, but go (titrate to remission of symptoms)
Minimize polypharmacy (maximize med indicated for primary disorder first)
Use rating scales systematically
Partner with the family
Use your resources

## Slide 7
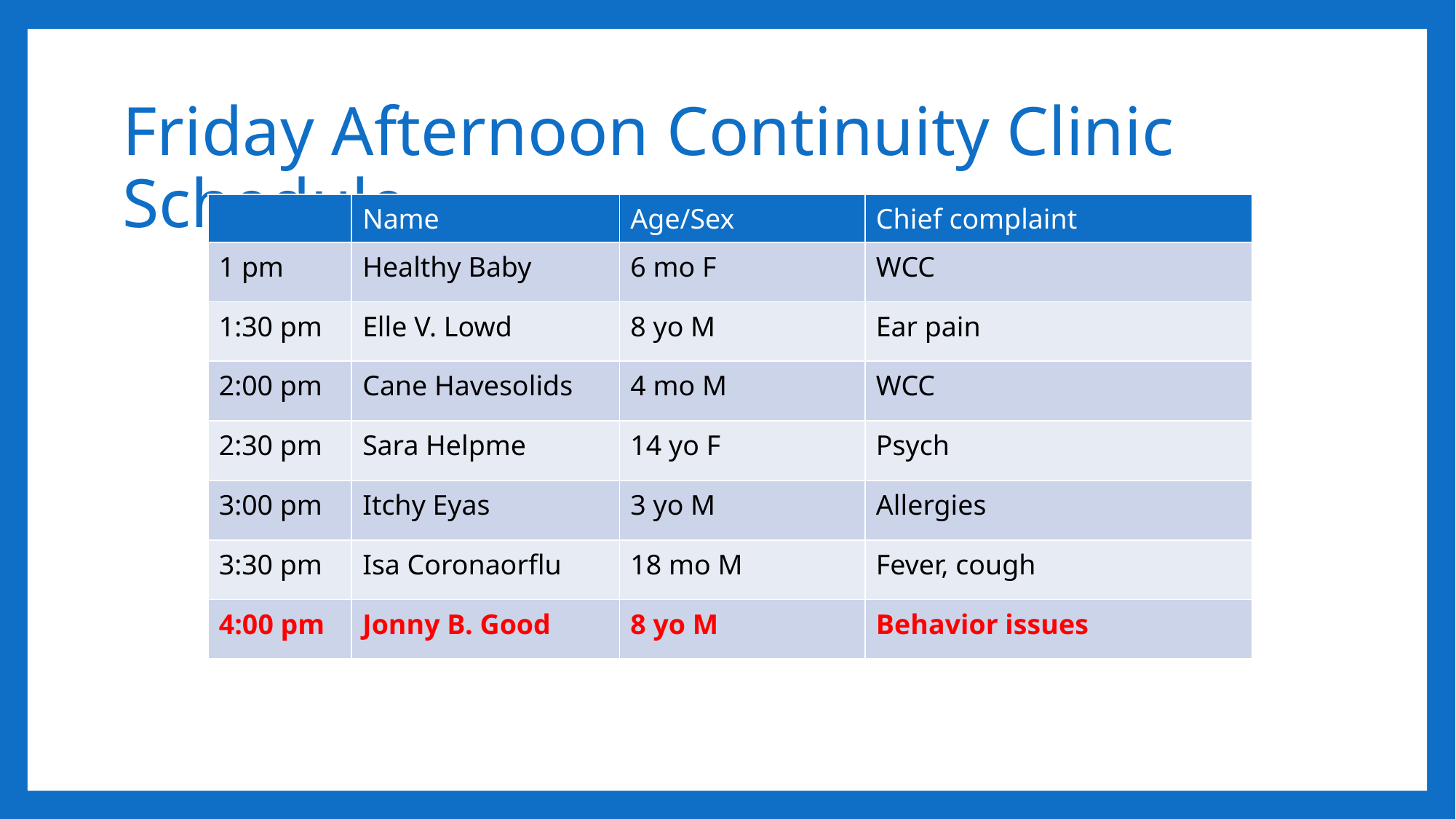

Friday Afternoon Continuity Clinic Schedule
| | Name | Age/Sex | Chief complaint |
| --- | --- | --- | --- |
| 1 pm | Healthy Baby | 6 mo F | WCC |
| 1:30 pm | Elle V. Lowd | 8 yo M | Ear pain |
| 2:00 pm | Cane Havesolids | 4 mo M | WCC |
| 2:30 pm | Sara Helpme | 14 yo F | Psych |
| 3:00 pm | Itchy Eyas | 3 yo M | Allergies |
| 3:30 pm | Isa Coronaorflu | 18 mo M | Fever, cough |
| 4:00 pm | Jonny B. Good | 8 yo M | Behavior issues |

## Slide 8
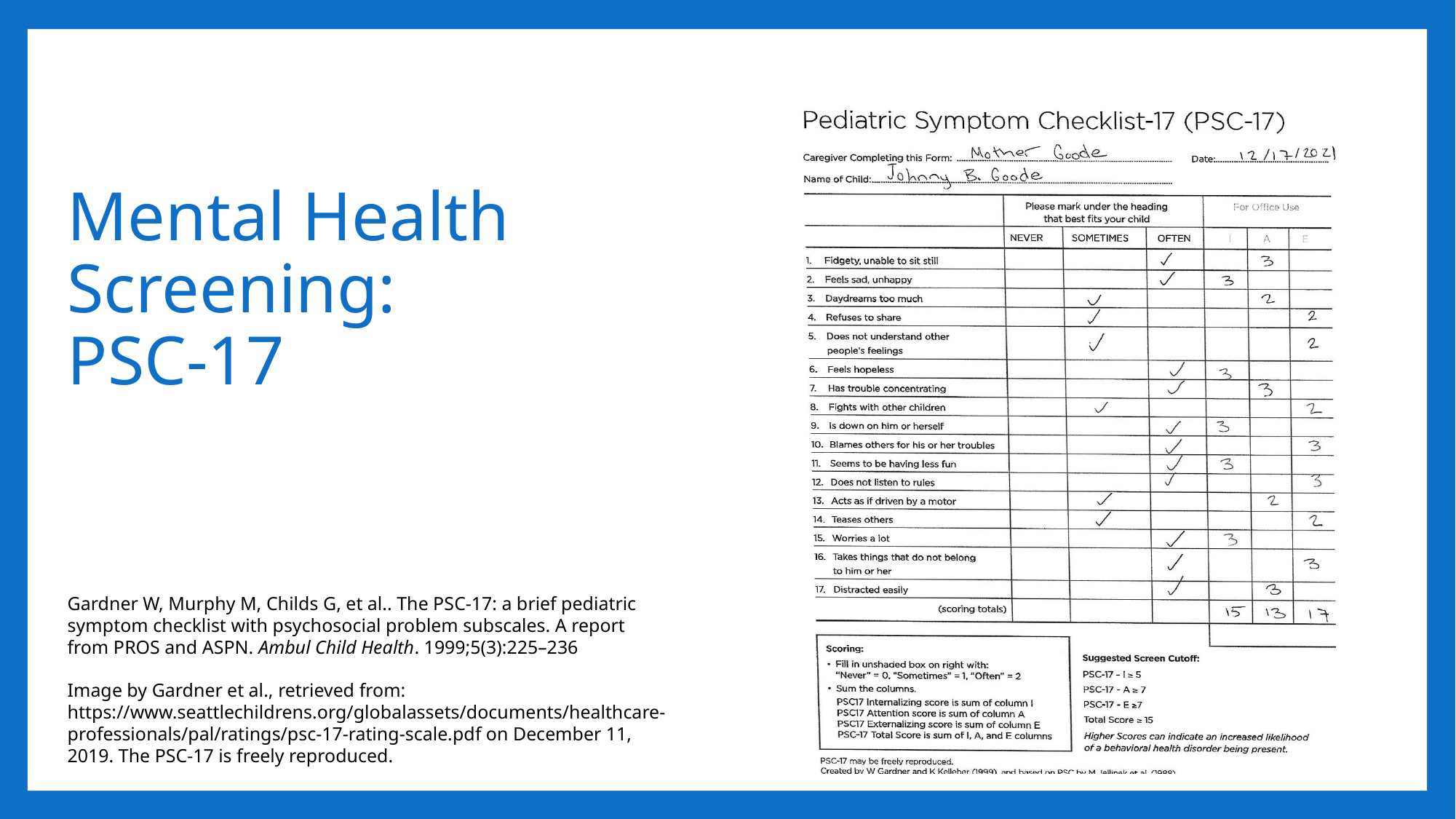

# Mental Health Screening: PSC-17
Gardner W, Murphy M, Childs G, et al.. The PSC-17: a brief pediatric symptom checklist with psychosocial problem subscales. A report from PROS and ASPN. Ambul Child Health. 1999;5(3):225–236
Image by Gardner et al., retrieved from: https://www.seattlechildrens.org/globalassets/documents/healthcare-professionals/pal/ratings/psc-17-rating-scale.pdf on December 11, 2019. The PSC-17 is freely reproduced.

## Slide 9
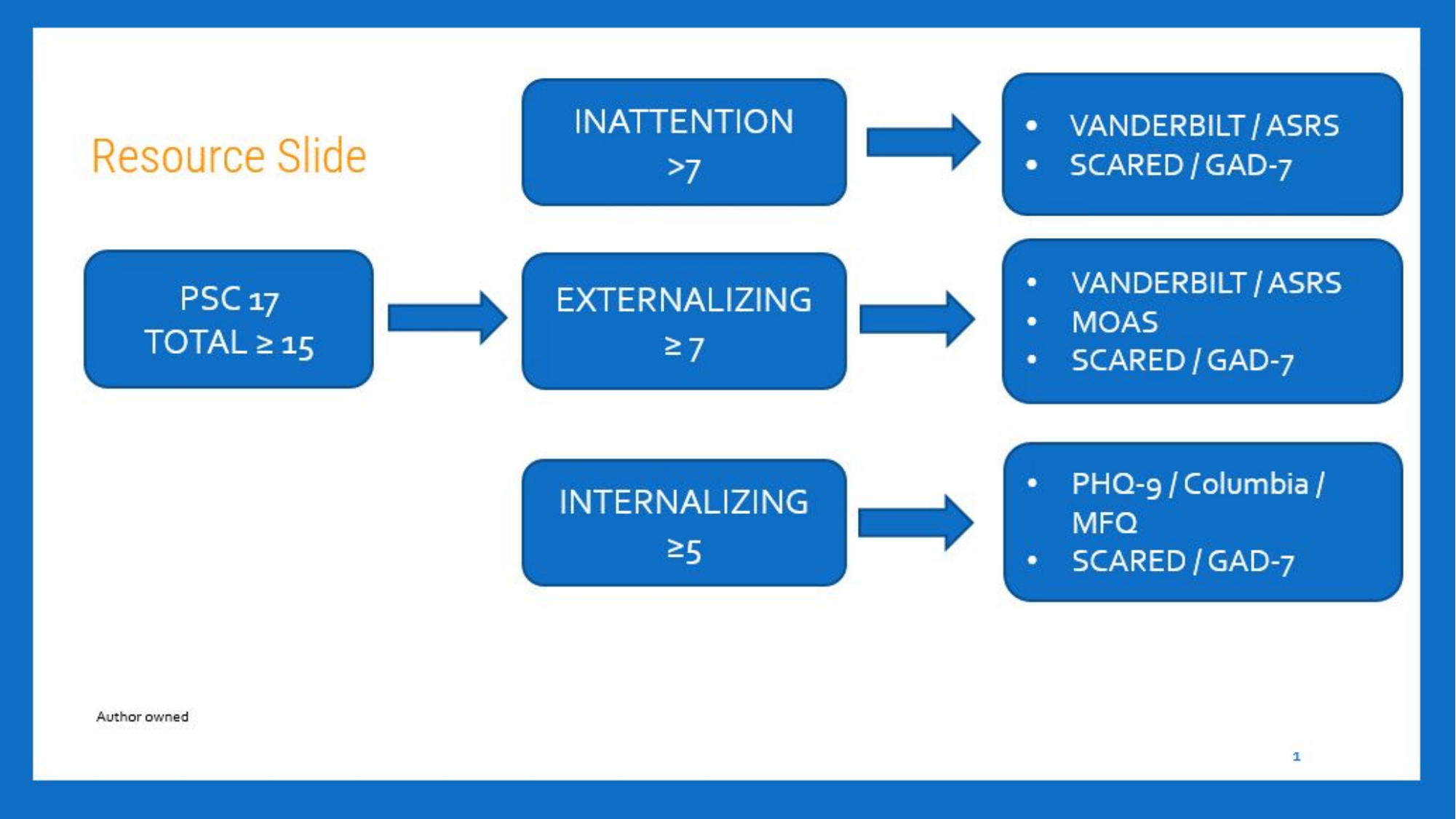

## Slide 10
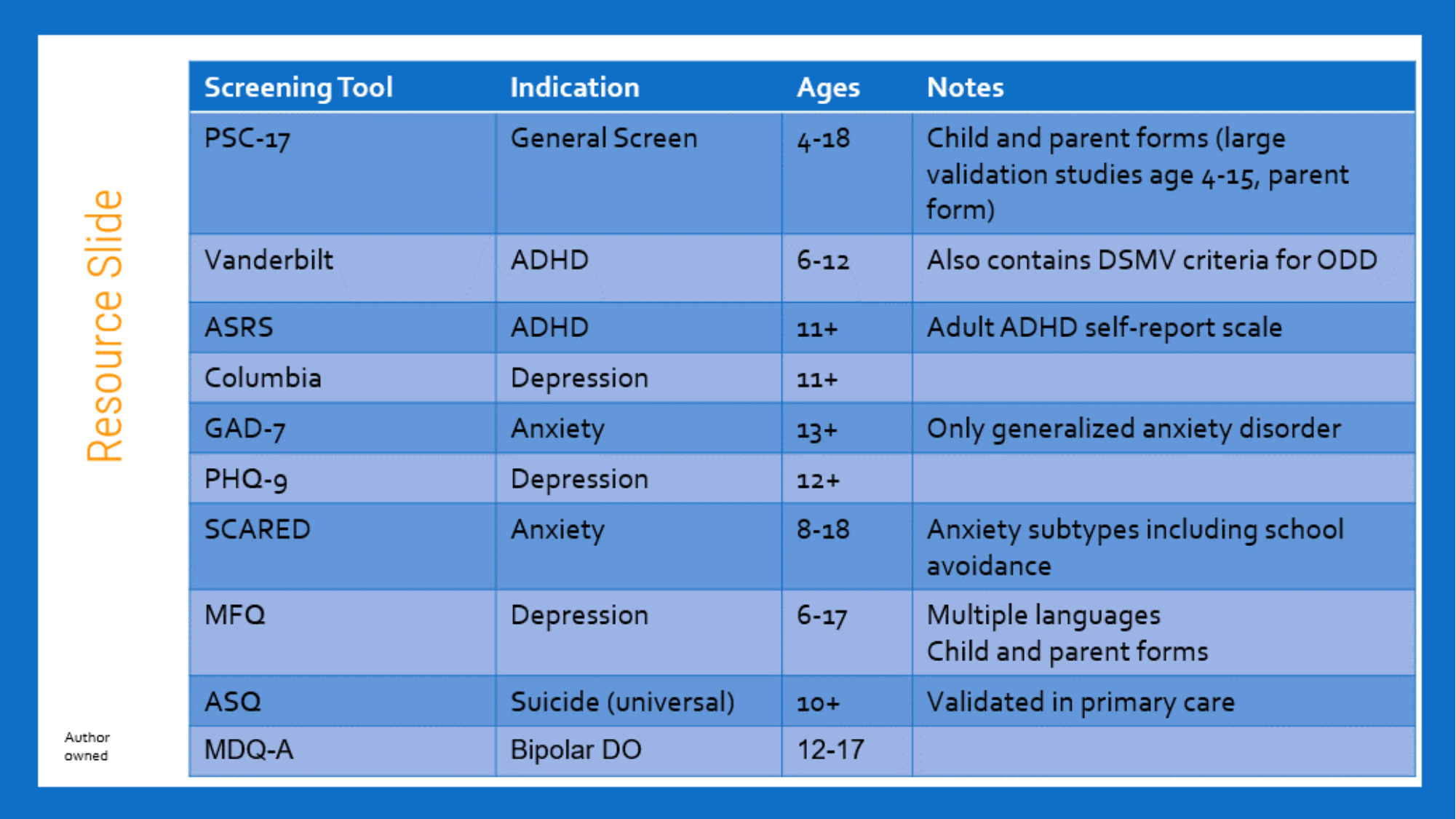

Supplement: Supplementary file 1 — Facilitator Guide.docxBe ExPeRT Introduction.pptxADHD in Primary Care Pediatrics.pptxAnxiety in Primary Care Pediatrics.pptxDepression in Primary Care Pediatrics.pptxBe ExPeRT Reference Slides.pptxParticipant Guide.docxBe ExPeRT Postsurvey.docxBe ExPeRT Case Discussion Form.docxBe ExPeRT Presurvey.docx [file mep_2374-8265.11326-s001.zip › B. Be ExPeRT Introduction.pptx]
