# Supplementary material for: Be ExPeRT (Behavioral Health Expansion in Pediatric Residency Training): A Case-Based Seminar
Source: MedEdPORTAL. 2023 Aug 1;19:11326. doi: 10.15766/mep_2374-8265.11326 (PMC10392710; doi:10.15766/mep_2374-8265.11326)
Supplement: Supplementary file 1 — Facilitator Guide.docxBe ExPeRT Introduction.pptxADHD in Primary Care Pediatrics.pptxAnxiety in Primary Care Pediatrics.pptxDepression in Primary Care Pediatrics.pptxBe ExPeRT Reference Slides.pptxParticipant Guide.docxBe ExPeRT Postsurvey.docxBe ExPeRT Case Discussion Form.docxBe ExPeRT Presurvey.docx [file mep_2374-8265.11326-s001.zip › C. ADHD in Primary Care Pediatrics.pptx]

## Slide 1
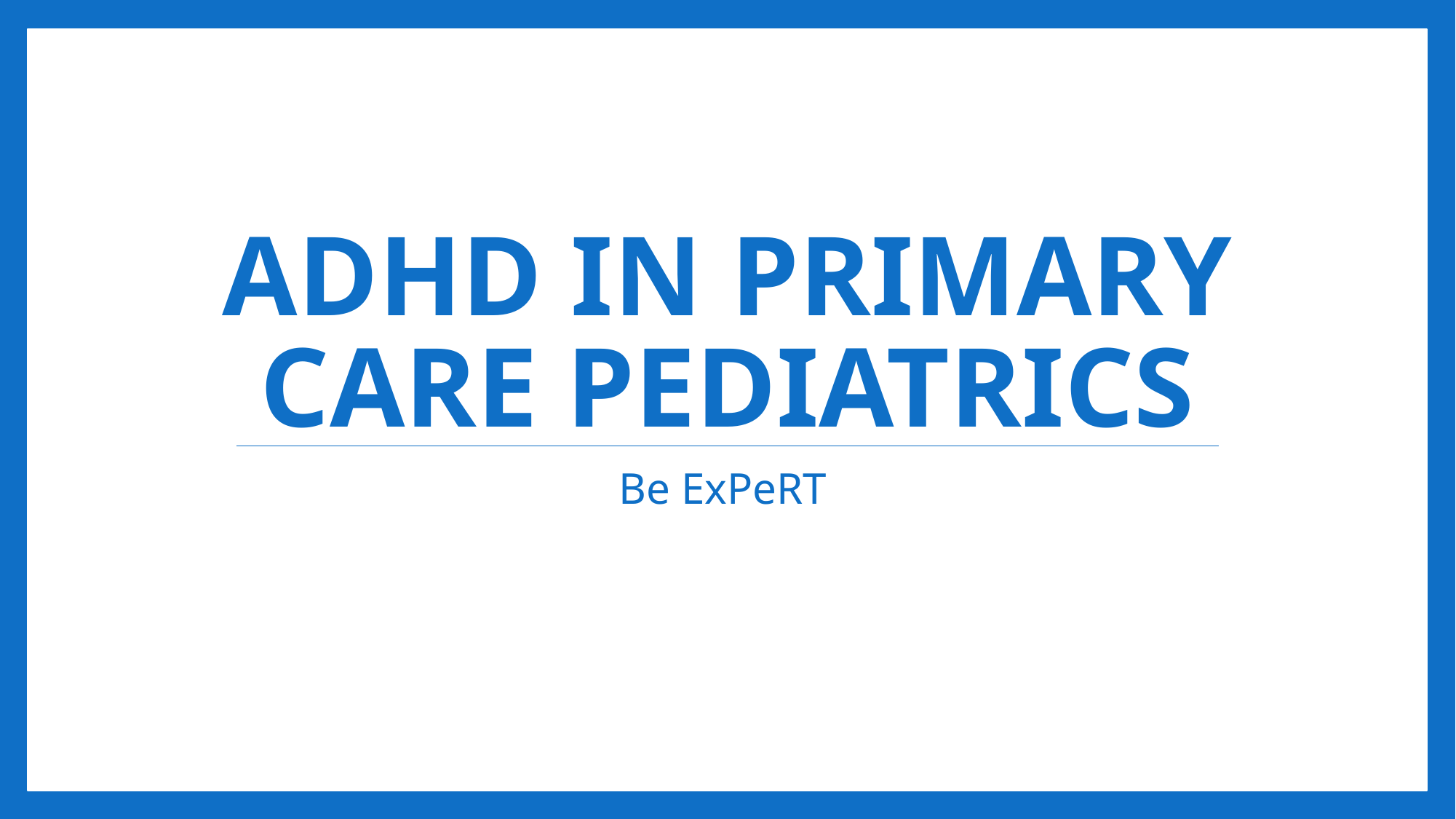

# Adhd in primary Care Pediatrics
Be ExPeRT

## Slide 2
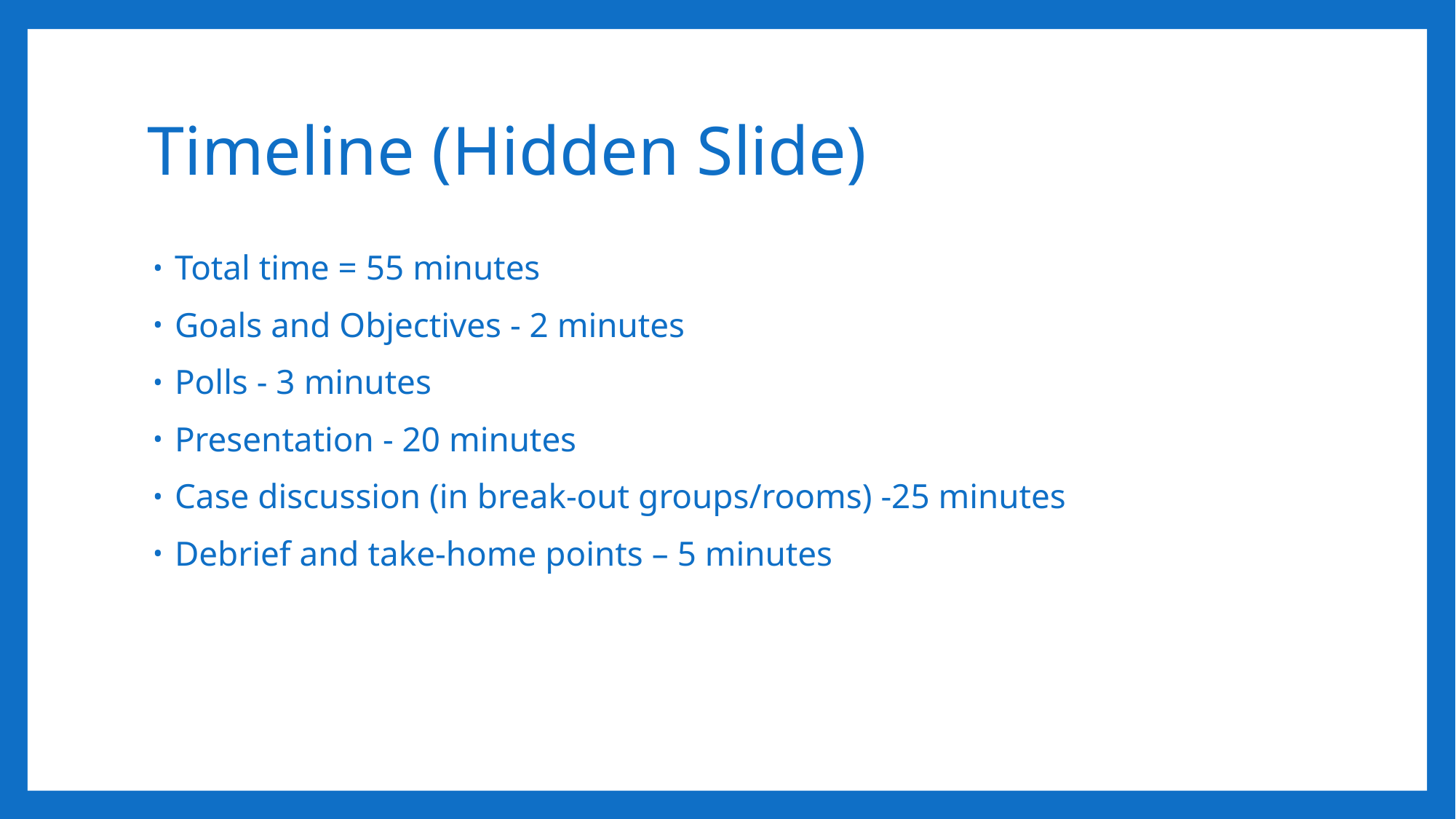

# Timeline (Hidden Slide)
Total time = 55 minutes
Goals and Objectives - 2 minutes
Polls - 3 minutes
Presentation - 20 minutes
Case discussion (in break-out groups/rooms) -25 minutes
Debrief and take-home points – 5 minutes

## Slide 3
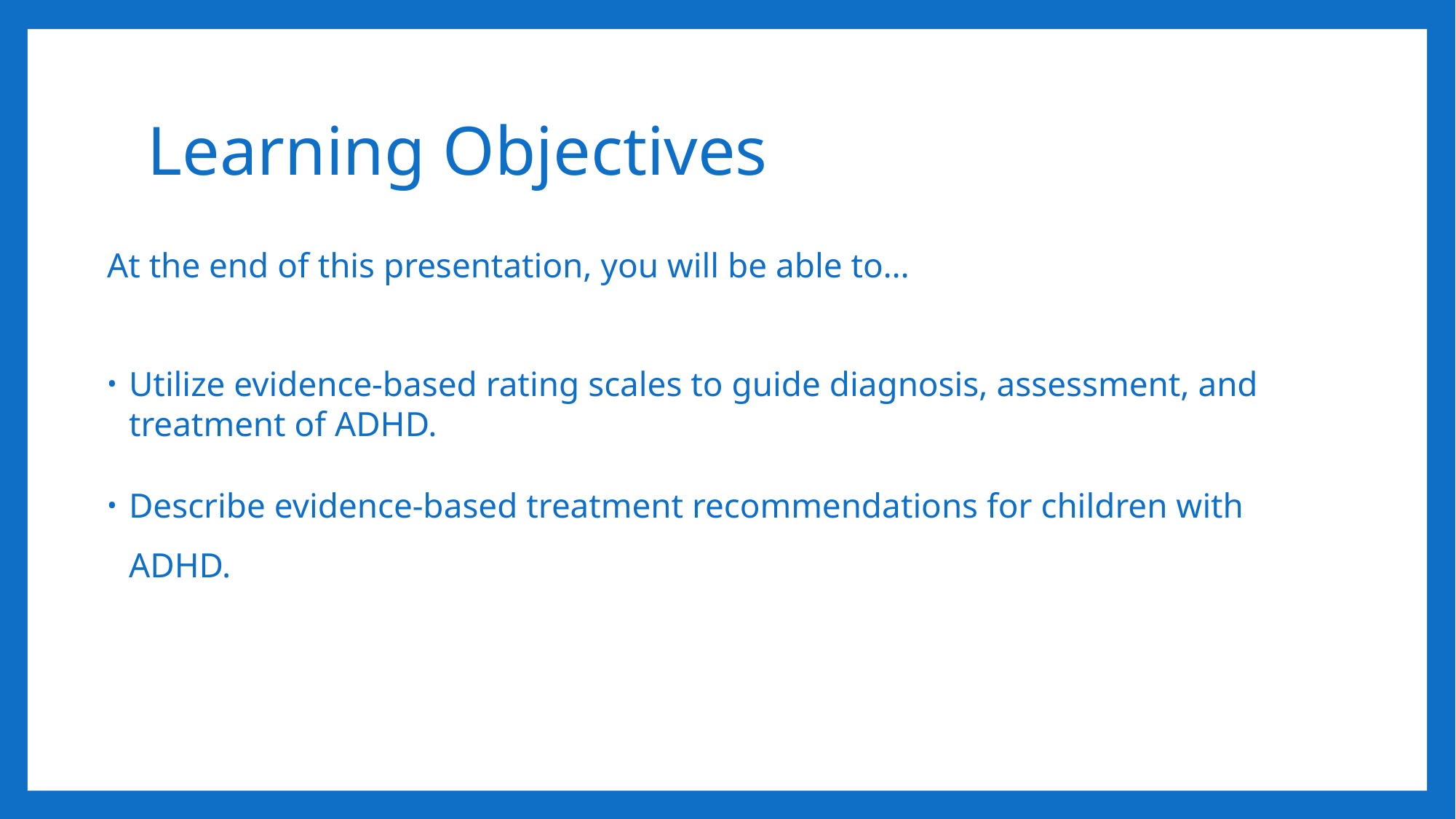

# Learning Objectives
At the end of this presentation, you will be able to…
Utilize evidence-based rating scales to guide diagnosis, assessment, and treatment of ADHD.
Describe evidence-based treatment recommendations for children with ADHD.

## Slide 4
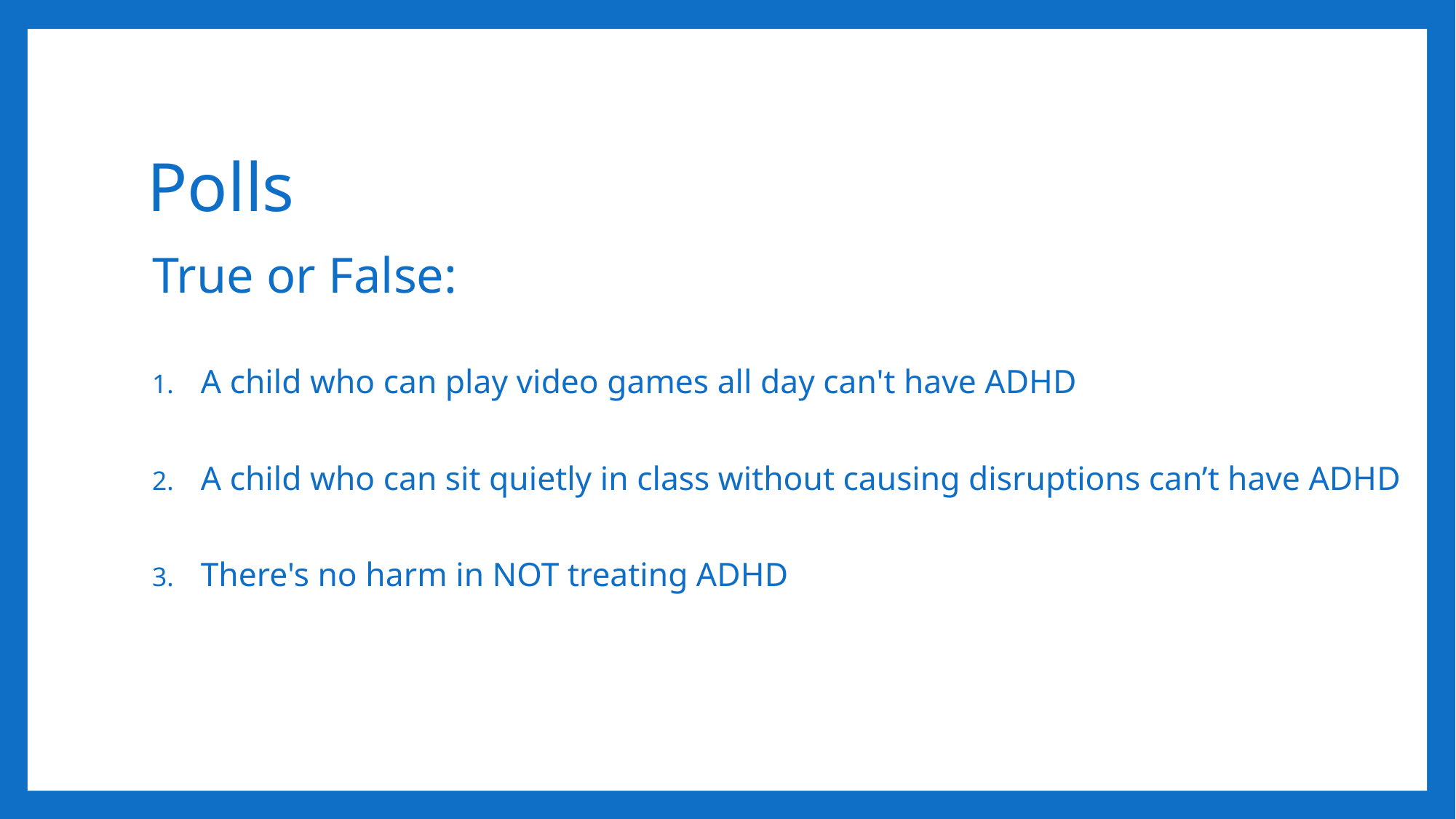

# Polls
True or False:
A child who can play video games all day can't have ADHD
A child who can sit quietly in class without causing disruptions can’t have ADHD
There's no harm in NOT treating ADHD

## Slide 5
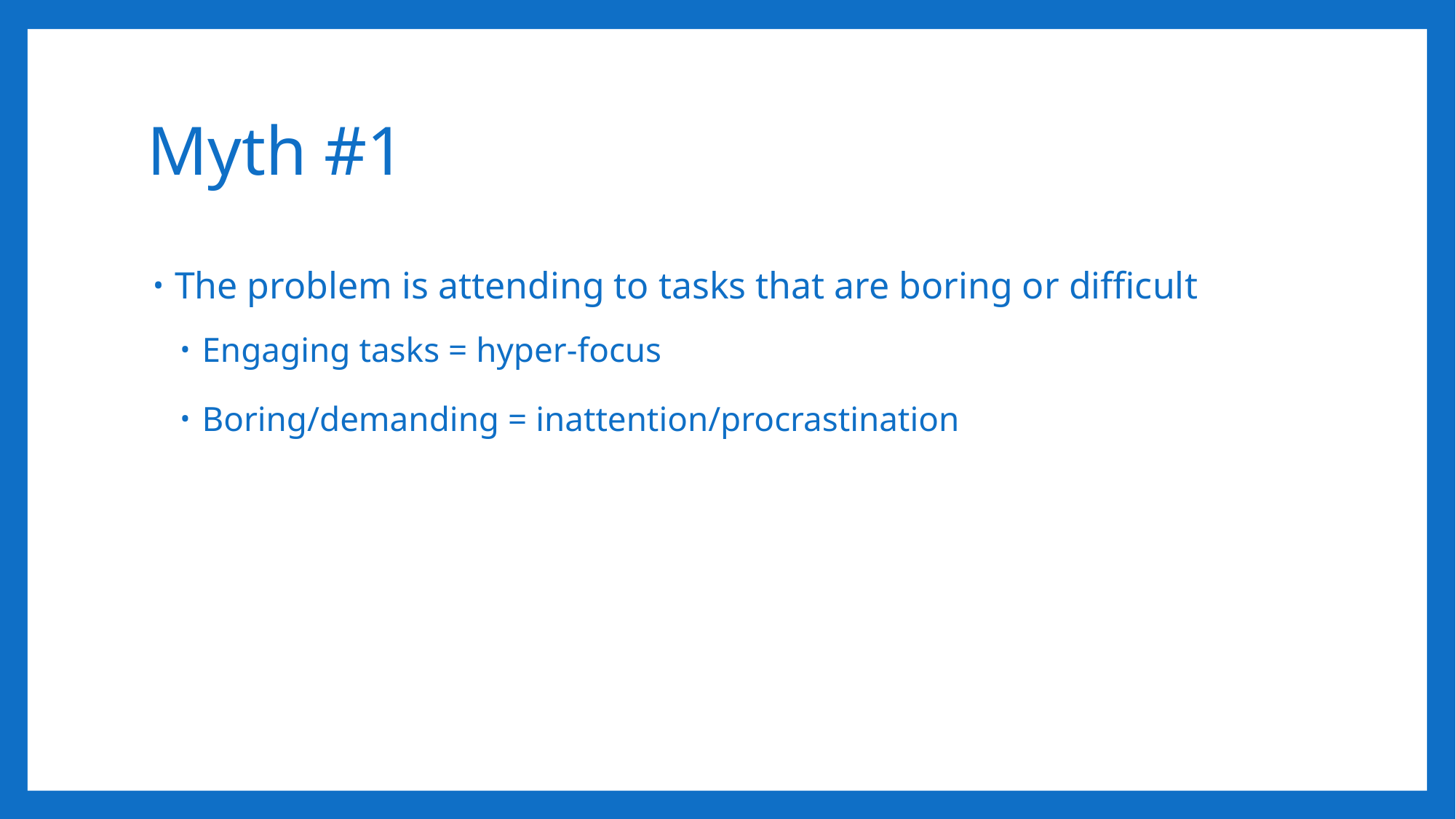

# Myth #1
The problem is attending to tasks that are boring or difficult
Engaging tasks = hyper-focus
Boring/demanding = inattention/procrastination

## Slide 6
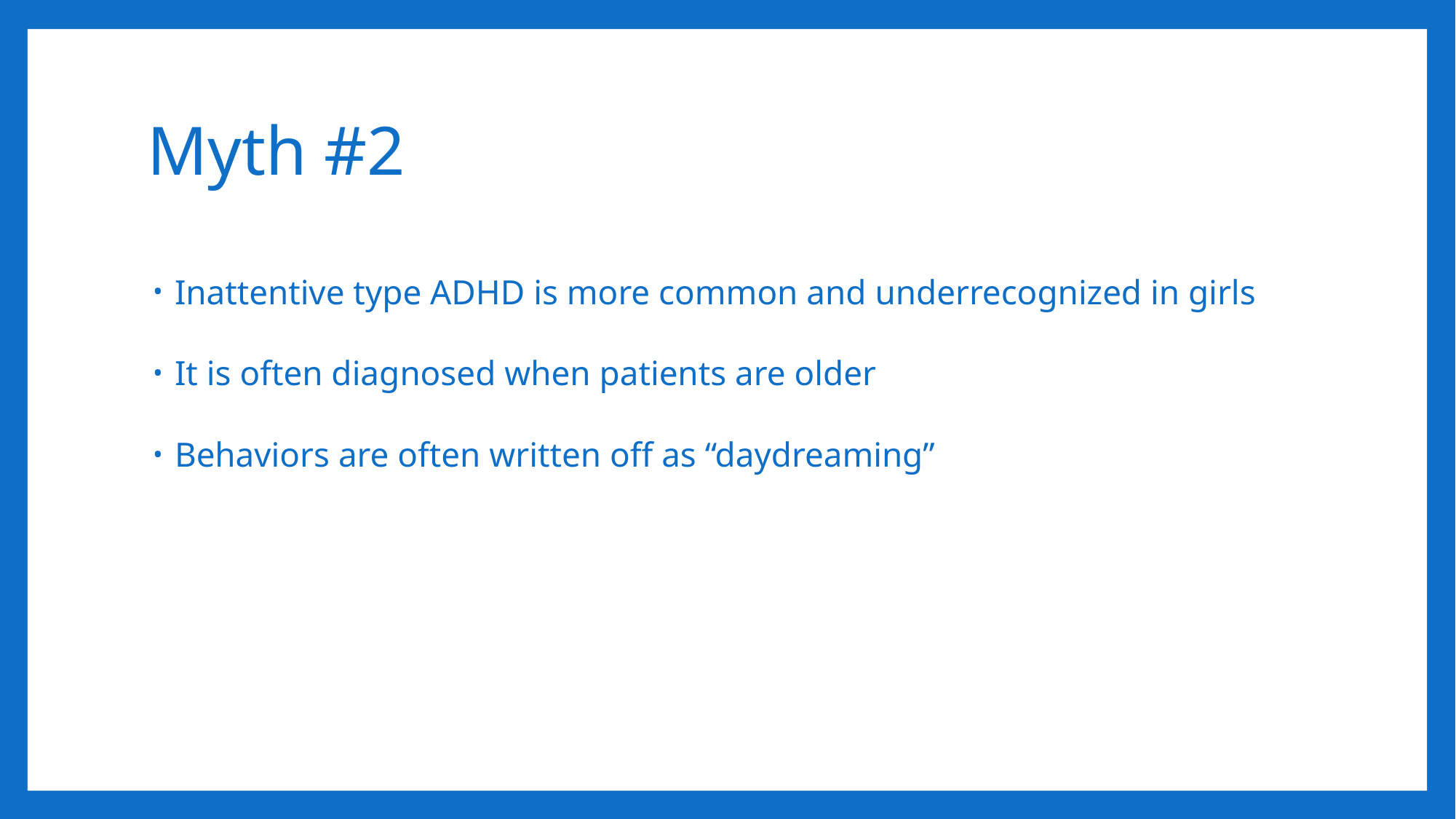

# Myth #2
Inattentive type ADHD is more common and underrecognized in girls
It is often diagnosed when patients are older
Behaviors are often written off as “daydreaming”

## Slide 7
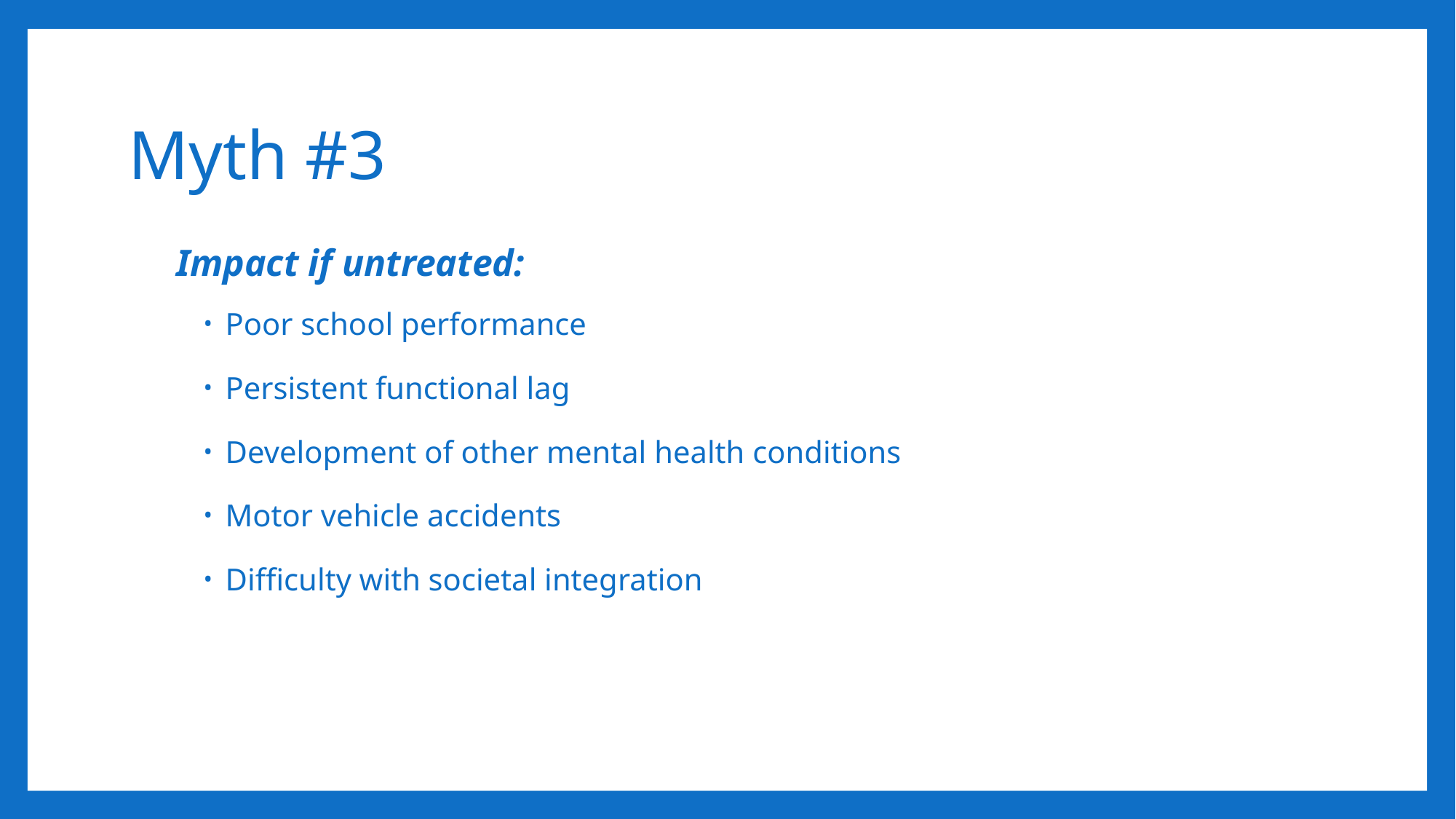

# Myth #3
Impact if untreated:
Poor school performance
Persistent functional lag
Development of other mental health conditions
Motor vehicle accidents
Difficulty with societal integration

## Slide 8
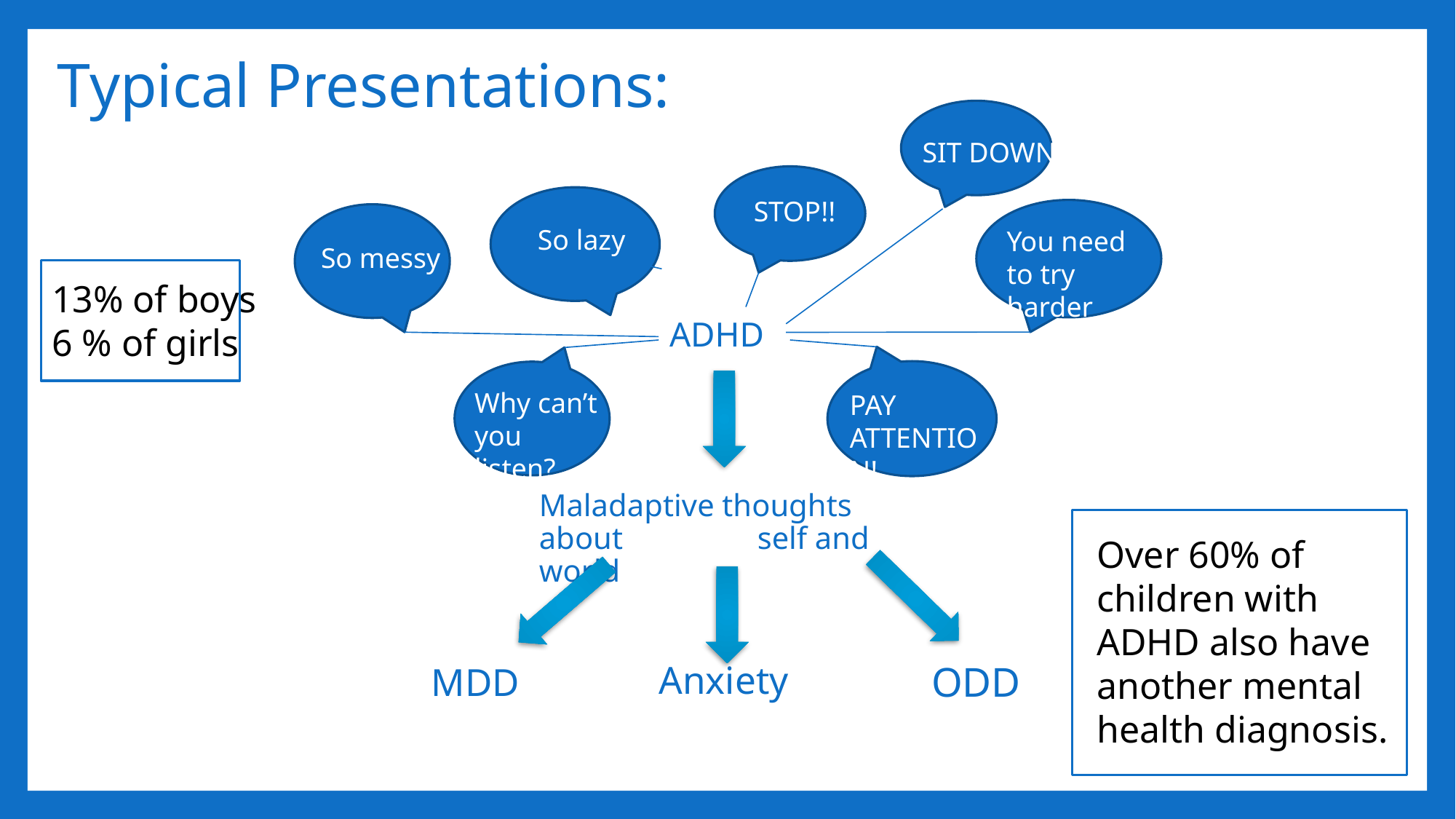

# Typical Presentations:
SIT DOWN
STOP!!
So lazy
You need to try harder
So messy
13% of boys
6 % of girls
ADHD
Why can’t
you listen?
PAY ATTENTION!
Maladaptive thoughts about 	self and world
Over 60% of children with ADHD also have another mental health diagnosis.
Anxiety
ODD
MDD

## Slide 9
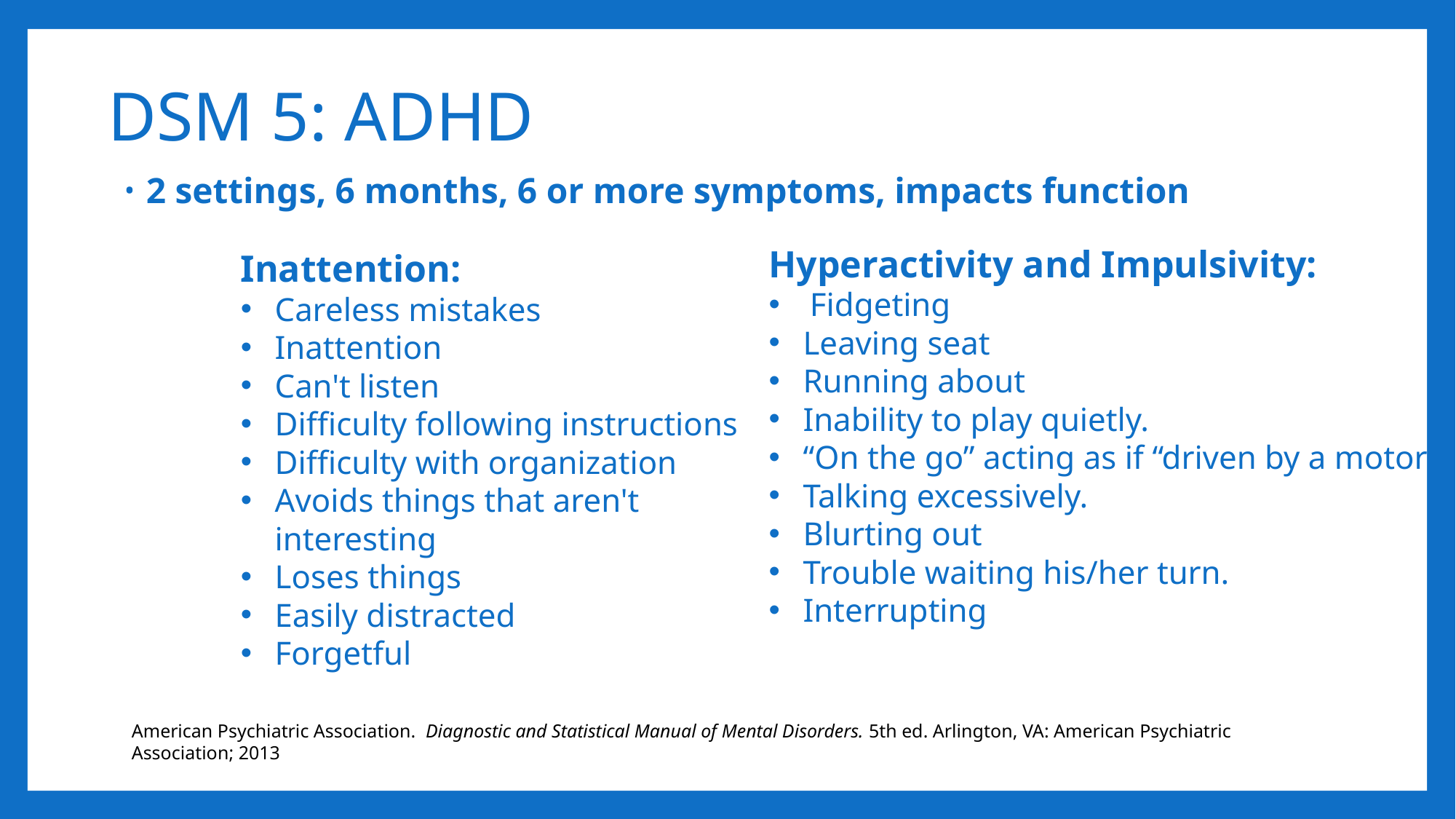

# DSM 5: ADHD
2 settings, 6 months, 6 or more symptoms, impacts function
Hyperactivity and Impulsivity:
Fidgeting
Leaving seat
Running about
Inability to play quietly.
“On the go” acting as if “driven by a motor”.
Talking excessively.
Blurting out
Trouble waiting his/her turn.
Interrupting
Inattention:
Careless mistakes
Inattention
Can't listen
Difficulty following instructions
Difficulty with organization
Avoids things that aren't interesting
Loses things
Easily distracted
Forgetful
American Psychiatric Association.  Diagnostic and Statistical Manual of Mental Disorders. 5th ed. Arlington, VA: American Psychiatric Association; 2013

## Slide 10
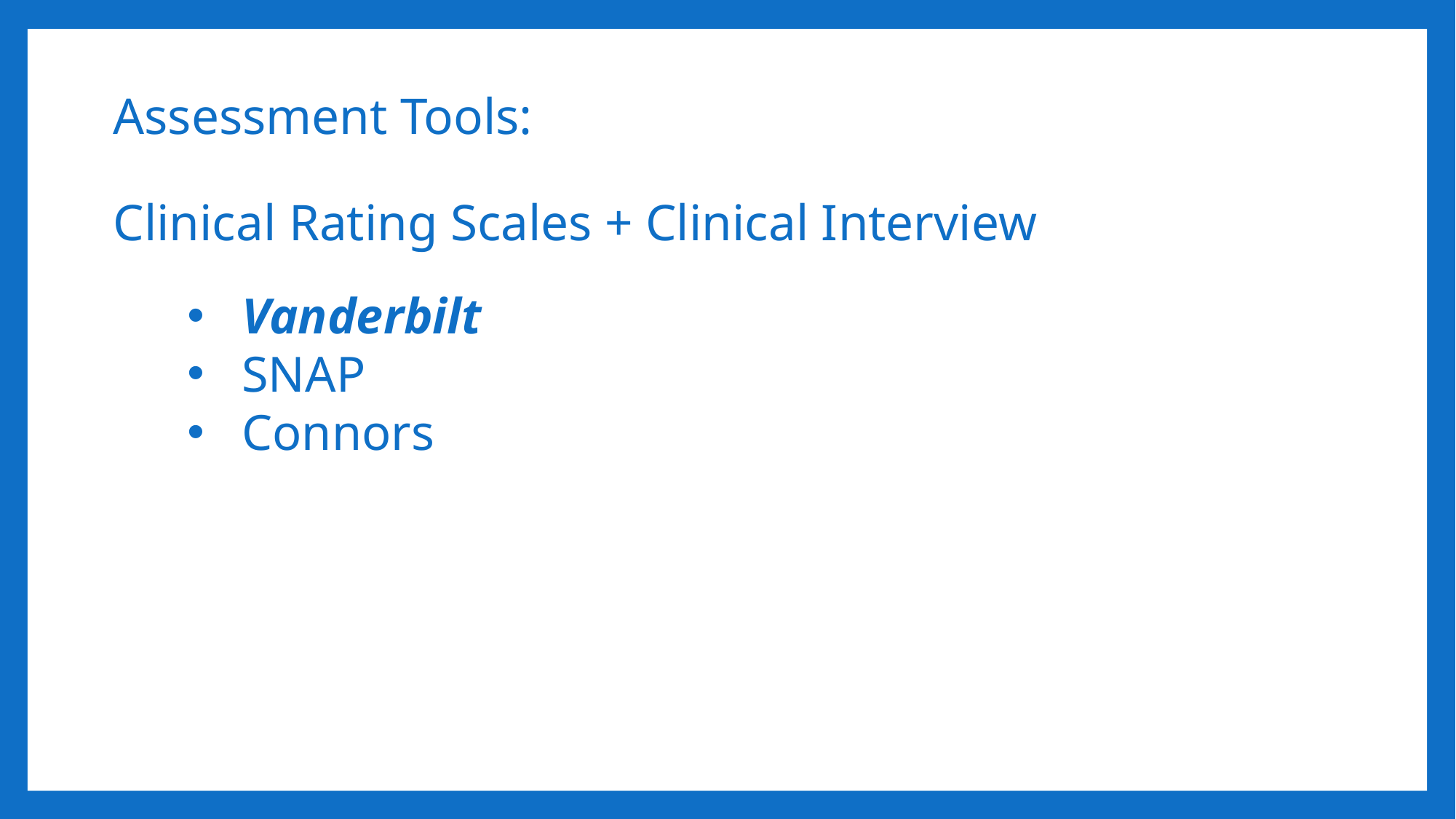

# Assessment Tools: Clinical Rating Scales + Clinical Interview
Vanderbilt
SNAP
Connors

## Slide 11
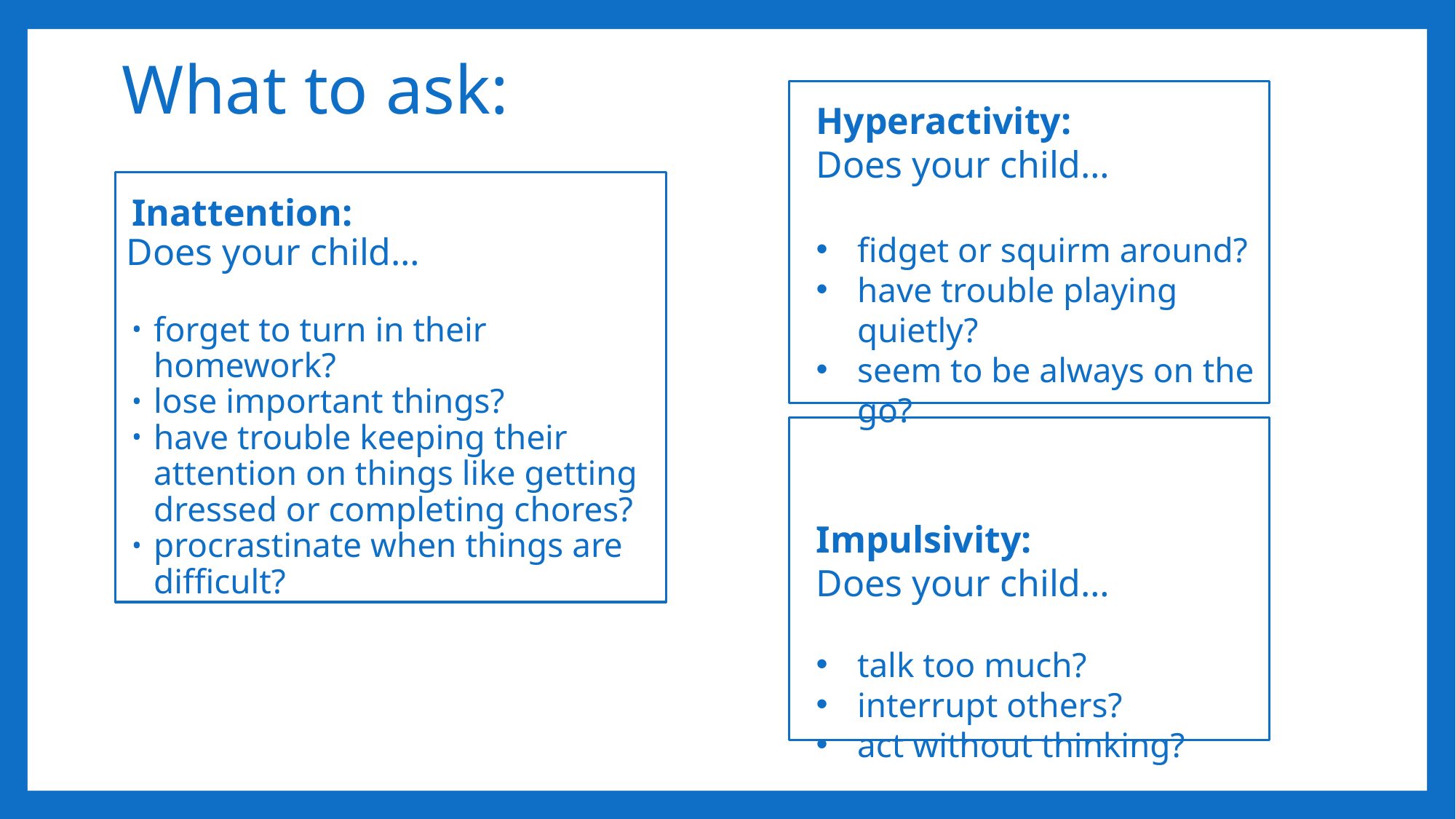

# What to ask:
Hyperactivity:
Does your child…
fidget or squirm around?
have trouble playing quietly?
seem to be always on the go?
Impulsivity:
Does your child…
talk too much?
interrupt others?
act without thinking?
Inattention:
Does your child…
forget to turn in their homework?
lose important things?
have trouble keeping their attention on things like getting dressed or completing chores?
procrastinate when things are difficult?

## Slide 12
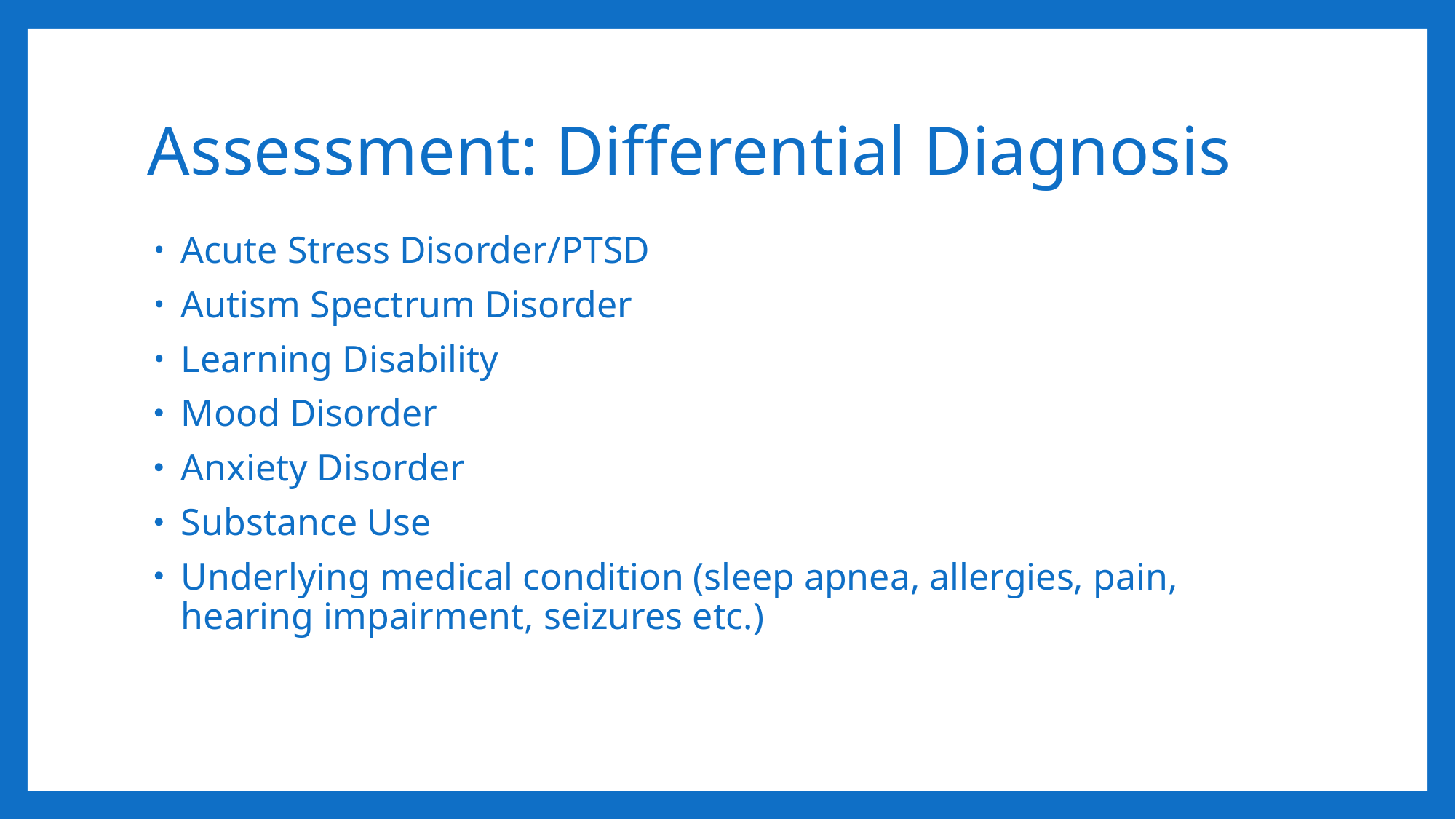

# Assessment: Differential Diagnosis
Acute Stress Disorder/PTSD
Autism Spectrum Disorder
Learning Disability
Mood Disorder
Anxiety Disorder
Substance Use
Underlying medical condition (sleep apnea, allergies, pain, hearing impairment, seizures etc.)

## Slide 13
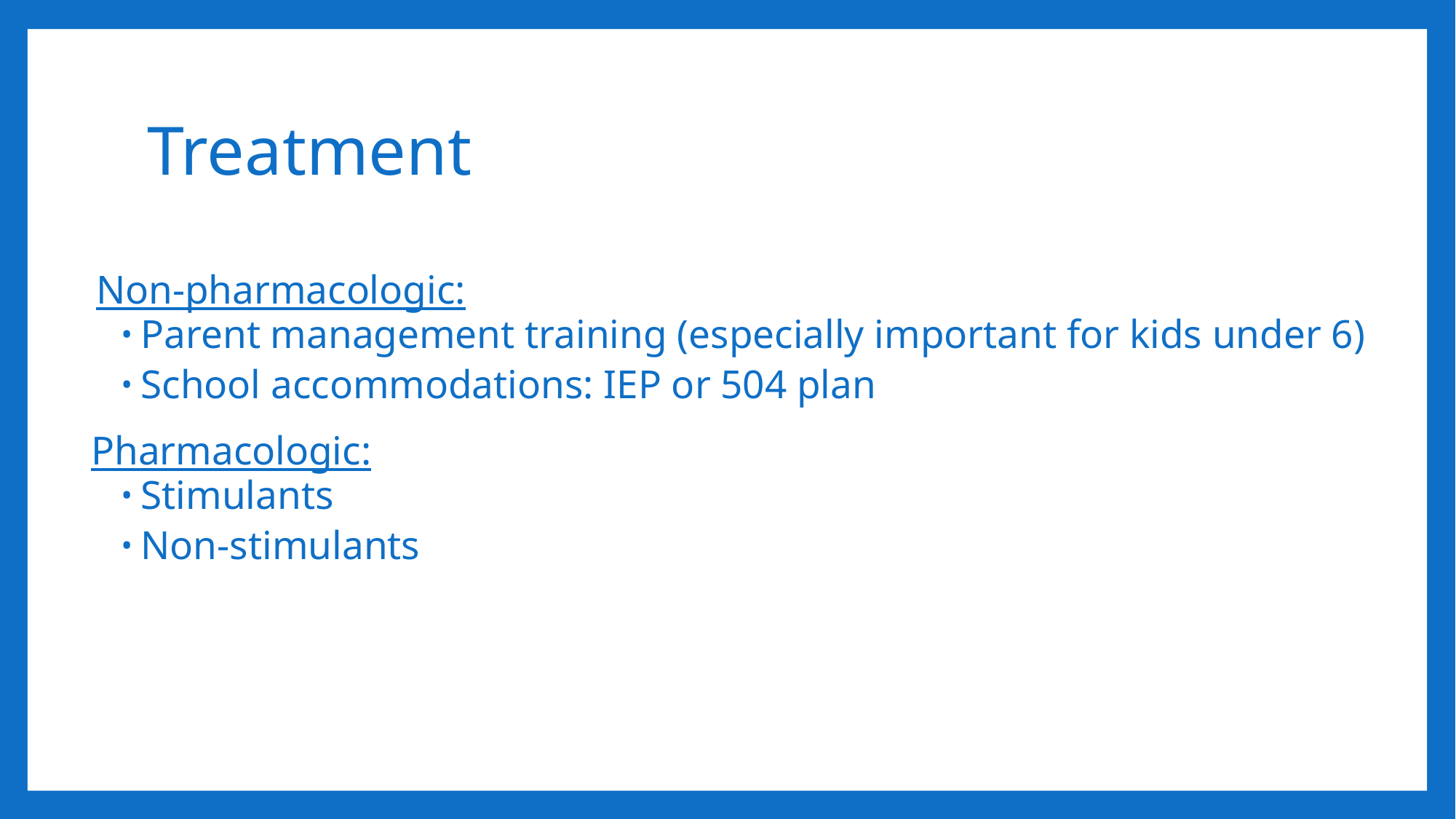

# Treatment
Non-pharmacologic:
Parent management training (especially important for kids under 6)
School accommodations: IEP or 504 plan
Pharmacologic:
Stimulants
Non-stimulants

## Slide 14
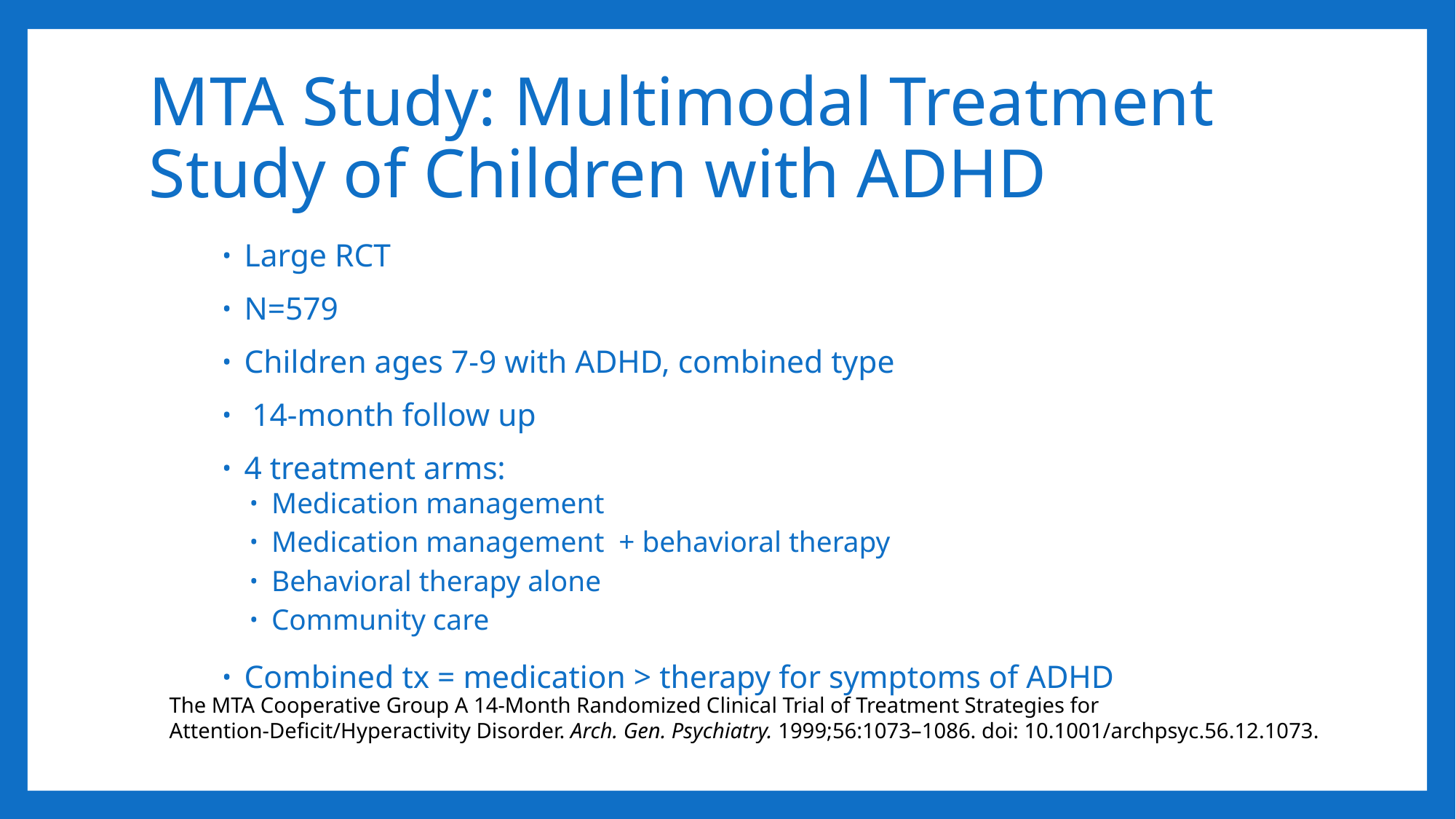

# MTA Study: Multimodal Treatment Study of Children with ADHD
Large RCT
N=579
Children ages 7-9 with ADHD, combined type
 14-month follow up
4 treatment arms:
Medication management
Medication management + behavioral therapy
Behavioral therapy alone
Community care
Combined tx = medication > therapy for symptoms of ADHD
The MTA Cooperative Group A 14-Month Randomized Clinical Trial of Treatment Strategies for Attention-Deficit/Hyperactivity Disorder. Arch. Gen. Psychiatry. 1999;56:1073–1086. doi: 10.1001/archpsyc.56.12.1073.

## Slide 15
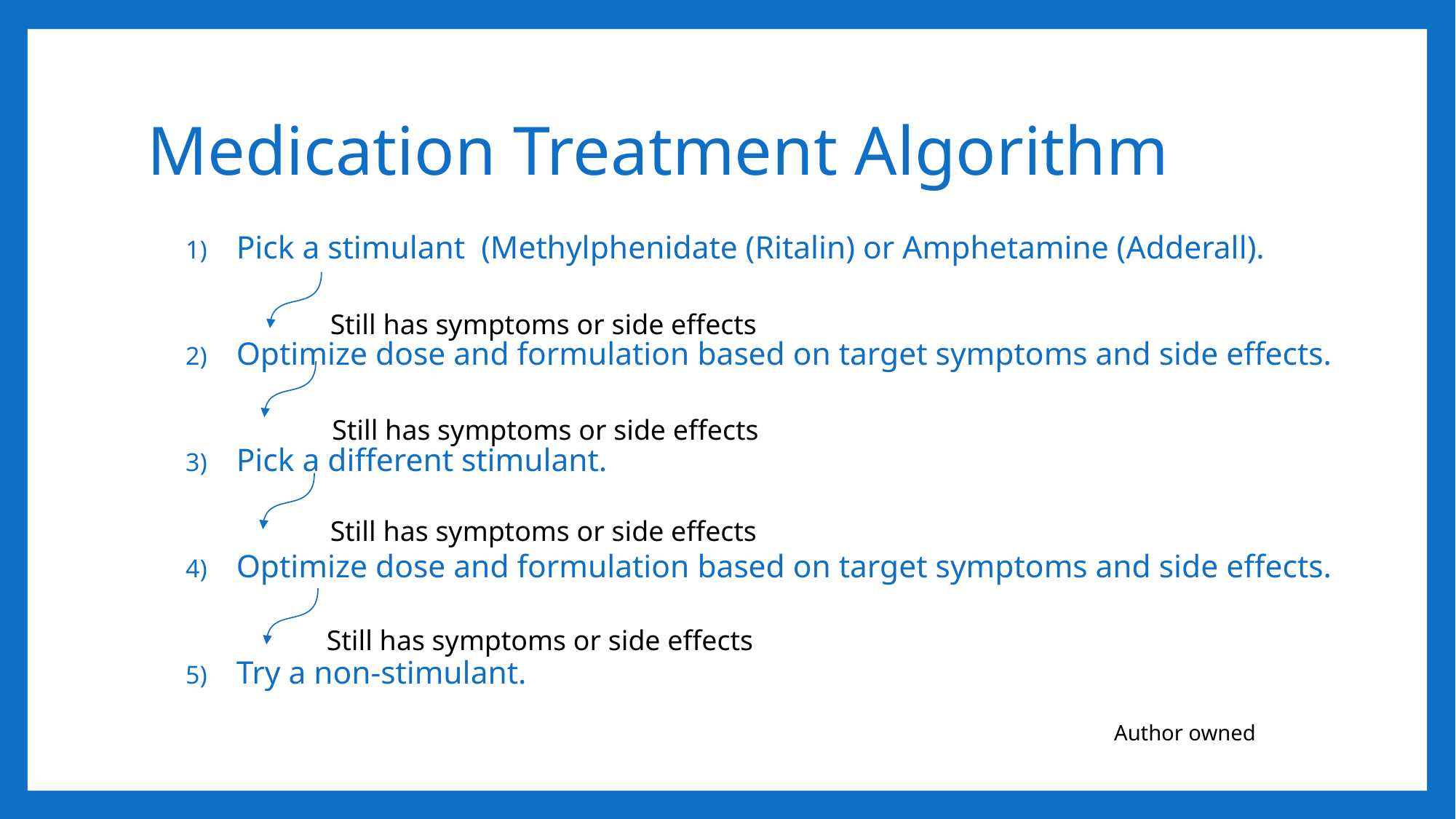

# Medication Treatment Algorithm
Pick a stimulant (Methylphenidate (Ritalin) or Amphetamine (Adderall).
Optimize dose and formulation based on target symptoms and side effects.
Pick a different stimulant.
Optimize dose and formulation based on target symptoms and side effects.
Try a non-stimulant.
Still has symptoms or side effects
Still has symptoms or side effects
Still has symptoms or side effects
Still has symptoms or side effects
Author owned

## Slide 16
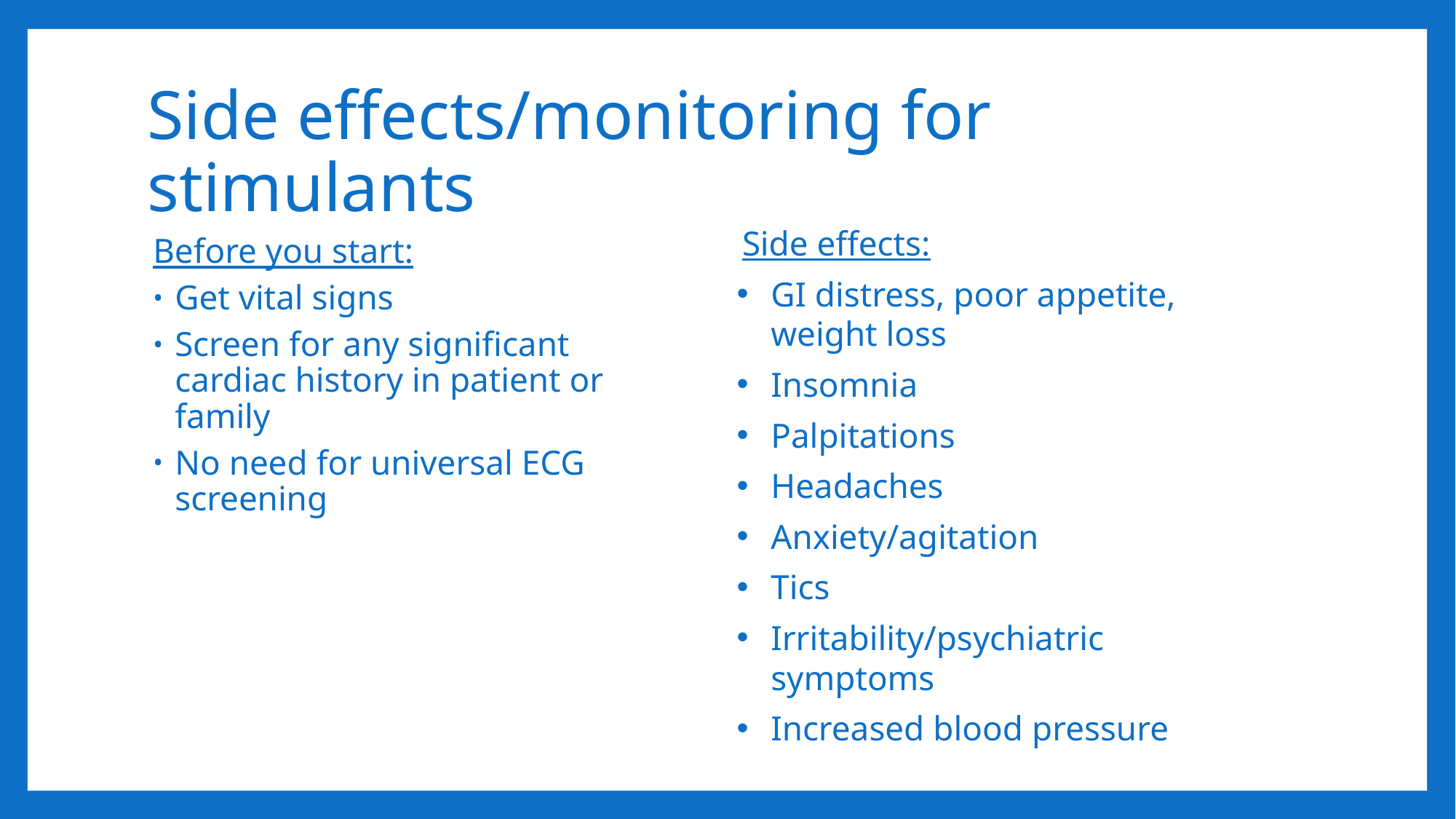

# Side effects/monitoring for stimulants
Side effects:
GI distress, poor appetite, weight loss
Insomnia
Palpitations
Headaches
Anxiety/agitation
Tics
Irritability/psychiatric symptoms
Increased blood pressure
Before you start:
Get vital signs
Screen for any significant cardiac history in patient or family
No need for universal ECG screening

## Slide 17
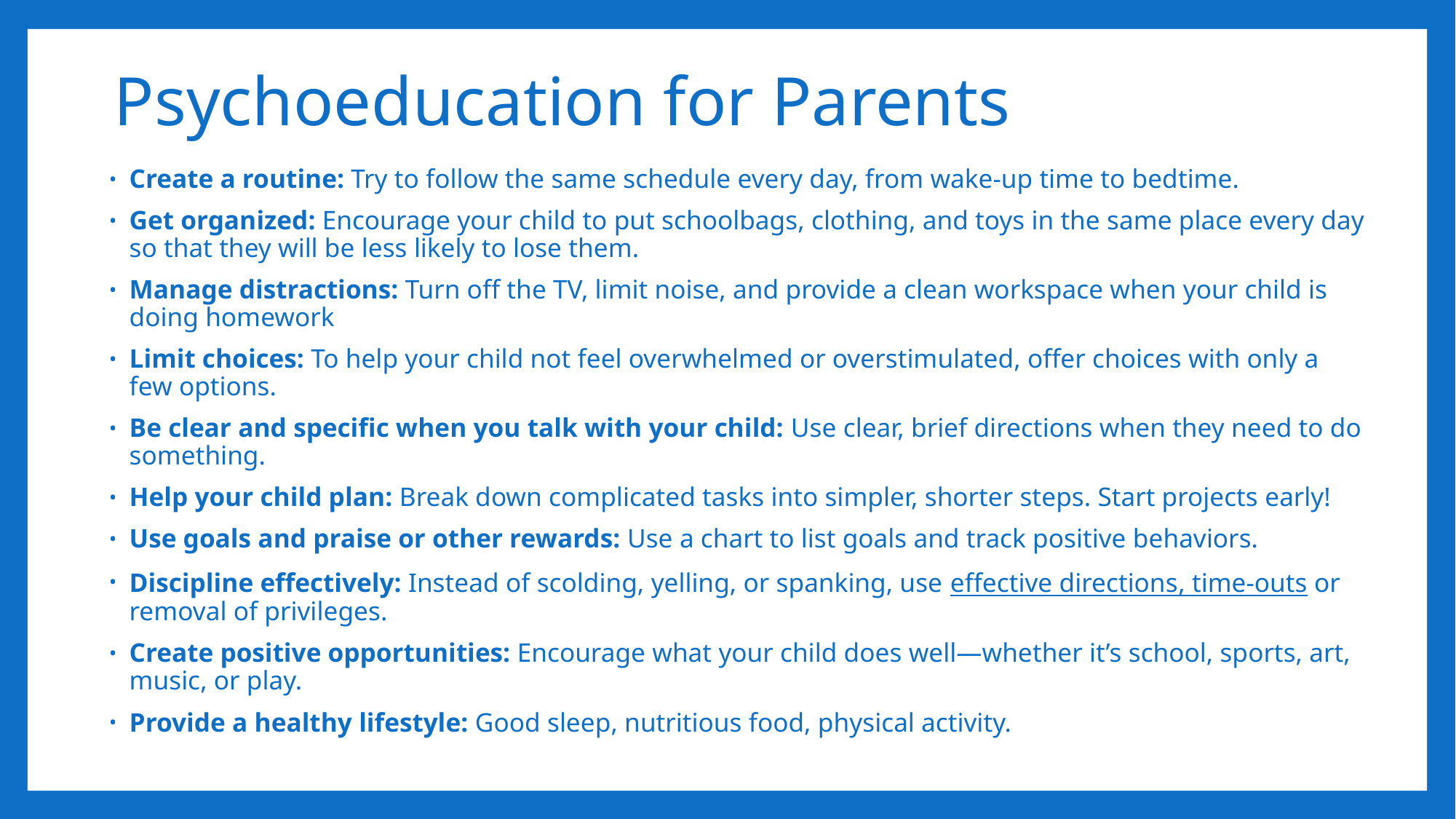

# Psychoeducation for Parents
Create a routine: Try to follow the same schedule every day, from wake-up time to bedtime.
Get organized: Encourage your child to put schoolbags, clothing, and toys in the same place every day so that they will be less likely to lose them.
Manage distractions: Turn off the TV, limit noise, and provide a clean workspace when your child is doing homework
Limit choices: To help your child not feel overwhelmed or overstimulated, offer choices with only a few options.
Be clear and specific when you talk with your child: Use clear, brief directions when they need to do something.
Help your child plan: Break down complicated tasks into simpler, shorter steps. Start projects early!
Use goals and praise or other rewards: Use a chart to list goals and track positive behaviors.
Discipline effectively: Instead of scolding, yelling, or spanking, use effective directions, time-outs or removal of privileges.
Create positive opportunities: Encourage what your child does well—whether it’s school, sports, art, music, or play.
Provide a healthy lifestyle: Good sleep, nutritious food, physical activity.

## Slide 18
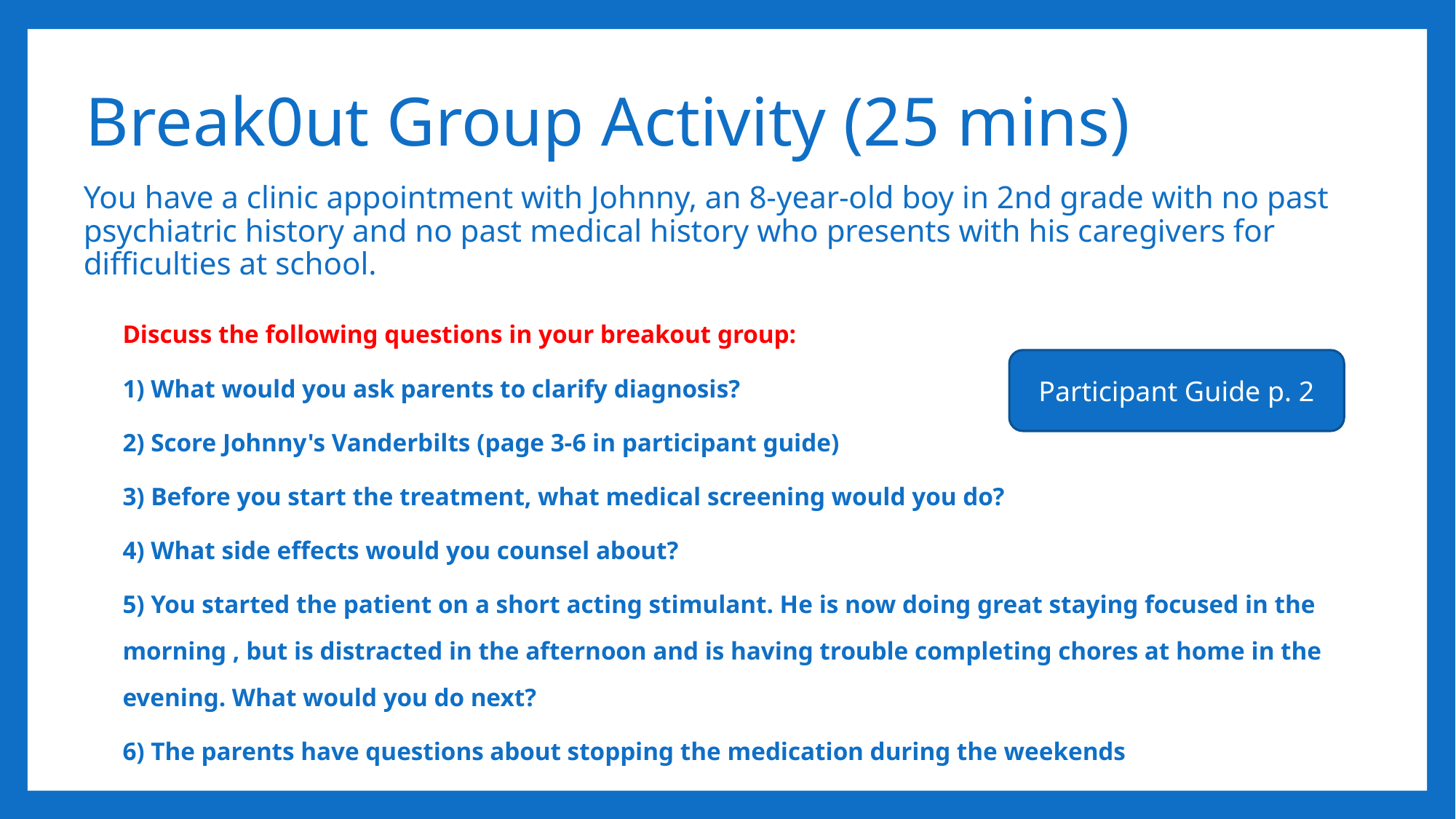

# Break0ut Group Activity (25 mins)
You have a clinic appointment with Johnny, an 8-year-old boy in 2nd grade with no past
psychiatric history and no past medical history who presents with his caregivers for difficulties at school.
Discuss the following questions in your breakout group:
1) What would you ask parents to clarify diagnosis?
2) Score Johnny's Vanderbilts (page 3-6 in participant guide)
3) Before you start the treatment, what medical screening would you do?
4) What side effects would you counsel about?
5) You started the patient on a short acting stimulant. He is now doing great staying focused in the morning , but is distracted in the afternoon and is having trouble completing chores at home in the evening. What would you do next?
6) The parents have questions about stopping the medication during the weekends
Participant Guide p. 2

## Slide 19
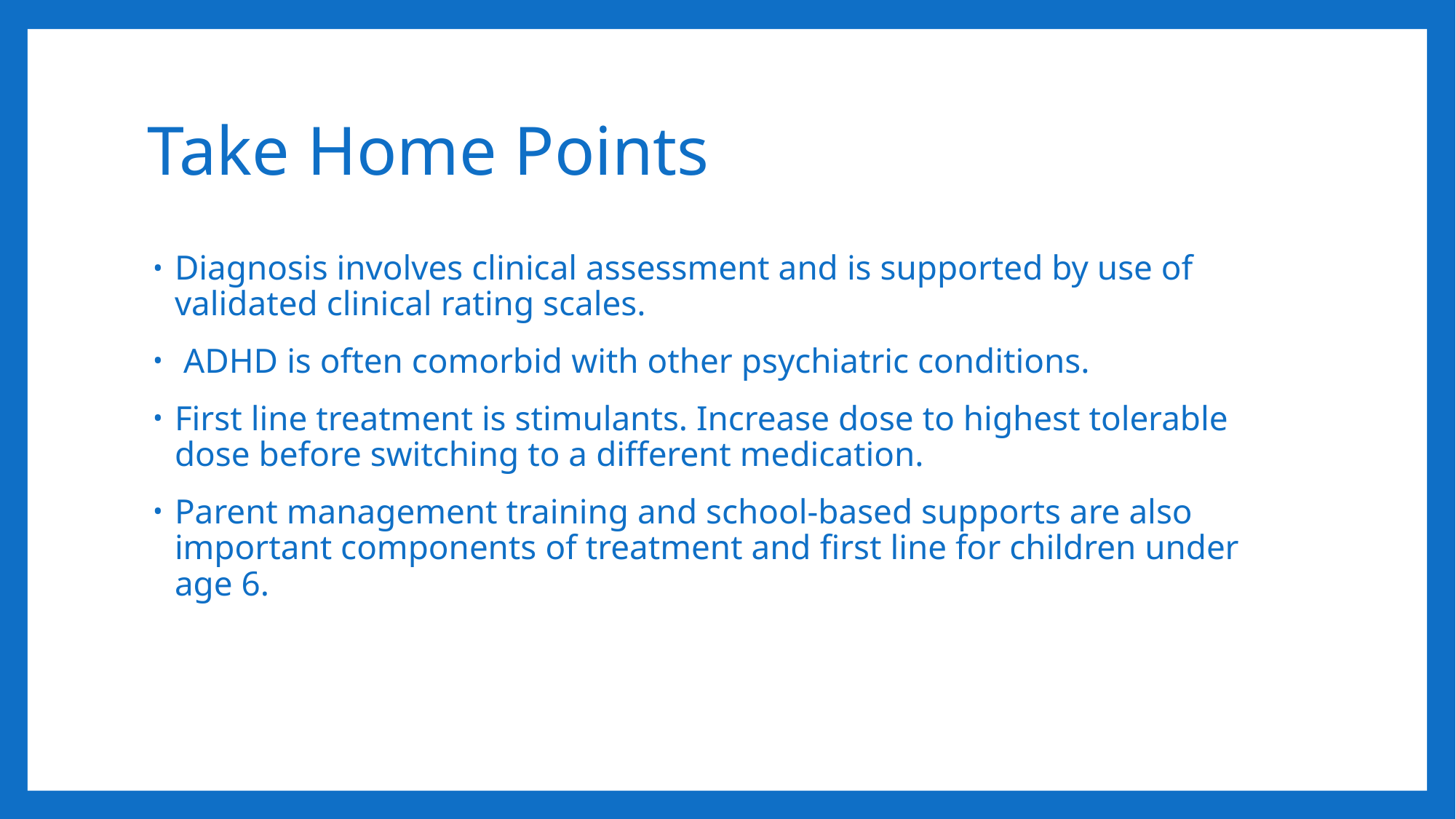

# Take Home Points
Diagnosis involves clinical assessment and is supported by use of validated clinical rating scales.
 ADHD is often comorbid with other psychiatric conditions.
First line treatment is stimulants. Increase dose to highest tolerable dose before switching to a different medication.
Parent management training and school-based supports are also important components of treatment and first line for children under age 6.
