# Supplementary material for: Be ExPeRT (Behavioral Health Expansion in Pediatric Residency Training): A Case-Based Seminar
Source: MedEdPORTAL. 2023 Aug 1;19:11326. doi: 10.15766/mep_2374-8265.11326 (PMC10392710; doi:10.15766/mep_2374-8265.11326)
Supplement: Supplementary file 1 — Facilitator Guide.docxBe ExPeRT Introduction.pptxADHD in Primary Care Pediatrics.pptxAnxiety in Primary Care Pediatrics.pptxDepression in Primary Care Pediatrics.pptxBe ExPeRT Reference Slides.pptxParticipant Guide.docxBe ExPeRT Postsurvey.docxBe ExPeRT Case Discussion Form.docxBe ExPeRT Presurvey.docx [file mep_2374-8265.11326-s001.zip › D. Anxiety in Primary Care Pediatrics.pptx]

## Slide 1
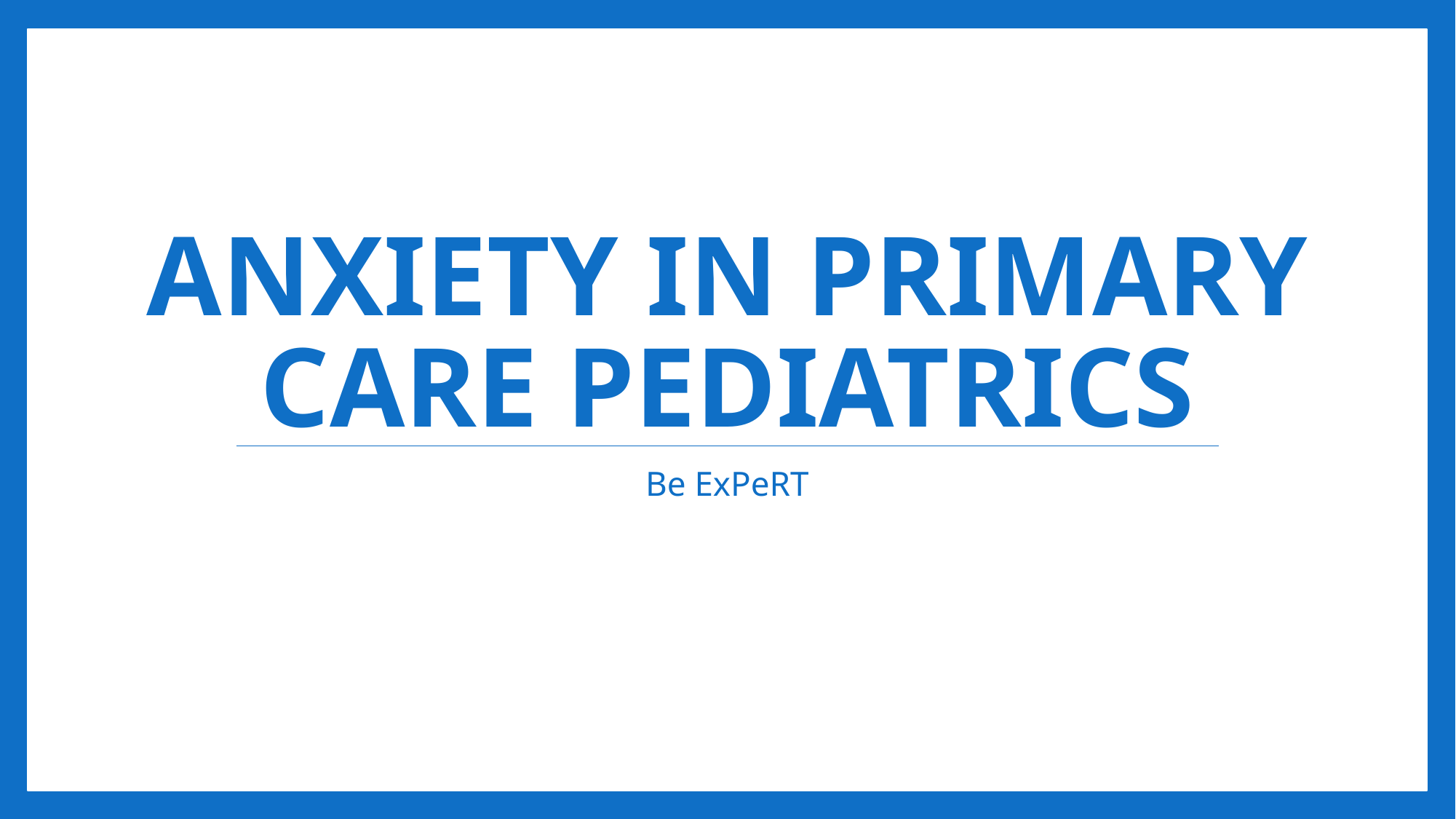

# Anxiety in primary Care Pediatrics
Be ExPeRT

## Slide 2
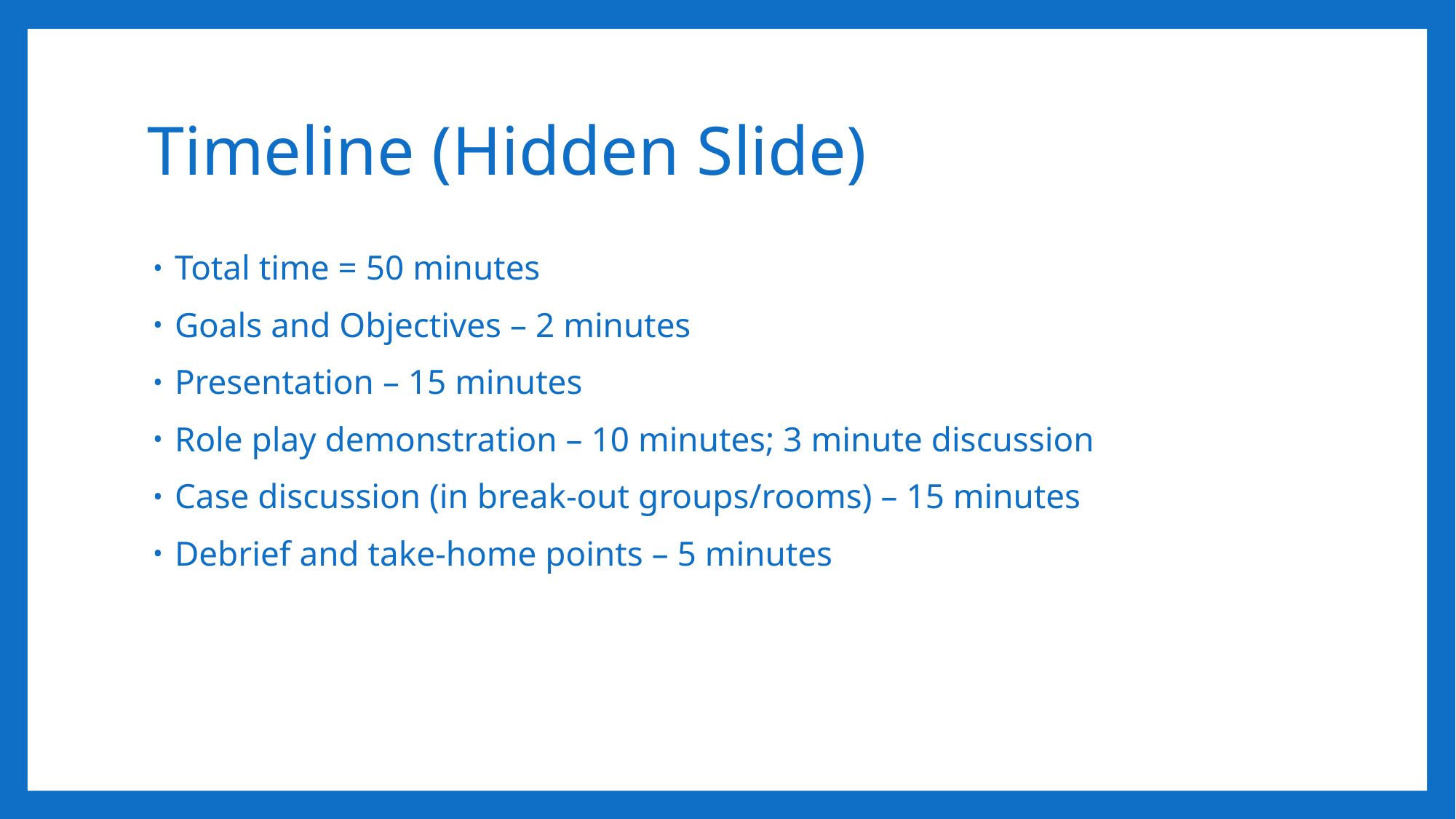

# Timeline (Hidden Slide)
Total time = 50 minutes
Goals and Objectives – 2 minutes
Presentation – 15 minutes
Role play demonstration – 10 minutes; 3 minute discussion
Case discussion (in break-out groups/rooms) – 15 minutes
Debrief and take-home points – 5 minutes

## Slide 3
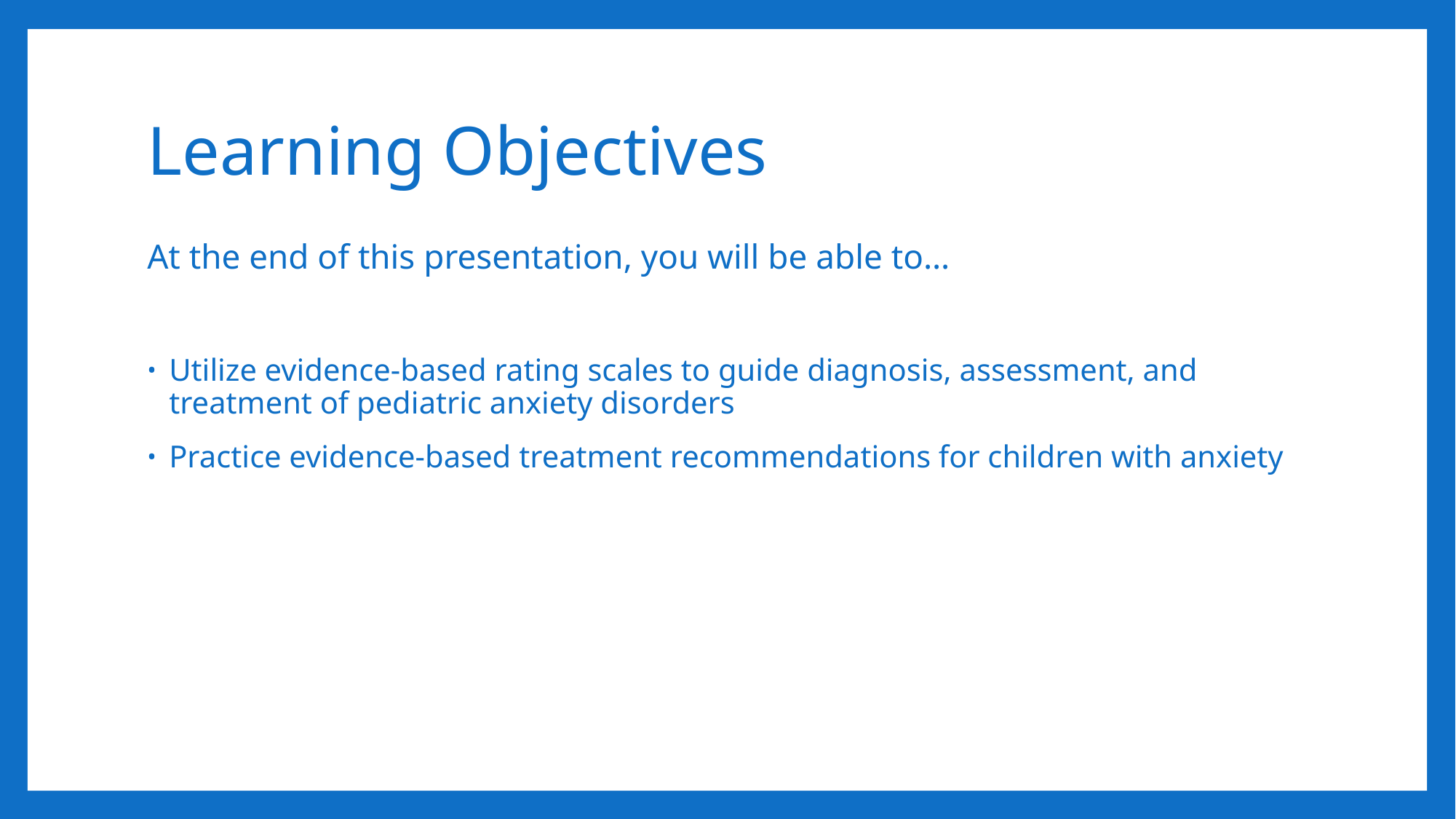

# Learning Objectives
At the end of this presentation, you will be able to…
Utilize evidence-based rating scales to guide diagnosis, assessment, and treatment of pediatric anxiety disorders
Practice evidence-based treatment recommendations for children with anxiety

## Slide 4
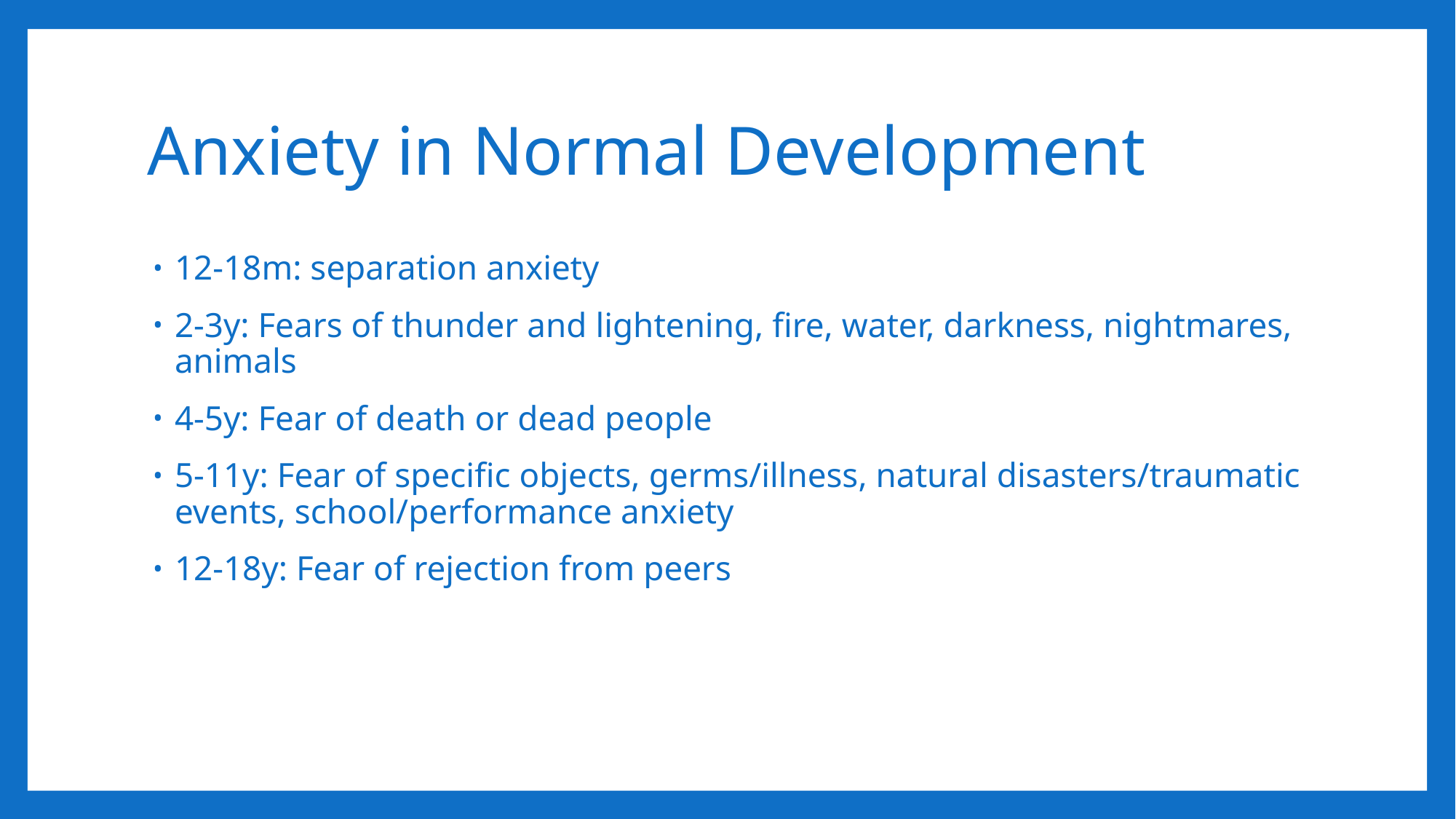

# Anxiety in Normal Development
12-18m: separation anxiety
2-3y: Fears of thunder and lightening, fire, water, darkness, nightmares, animals
4-5y: Fear of death or dead people
5-11y: Fear of specific objects, germs/illness, natural disasters/traumatic events, school/performance anxiety
12-18y: Fear of rejection from peers

## Slide 5
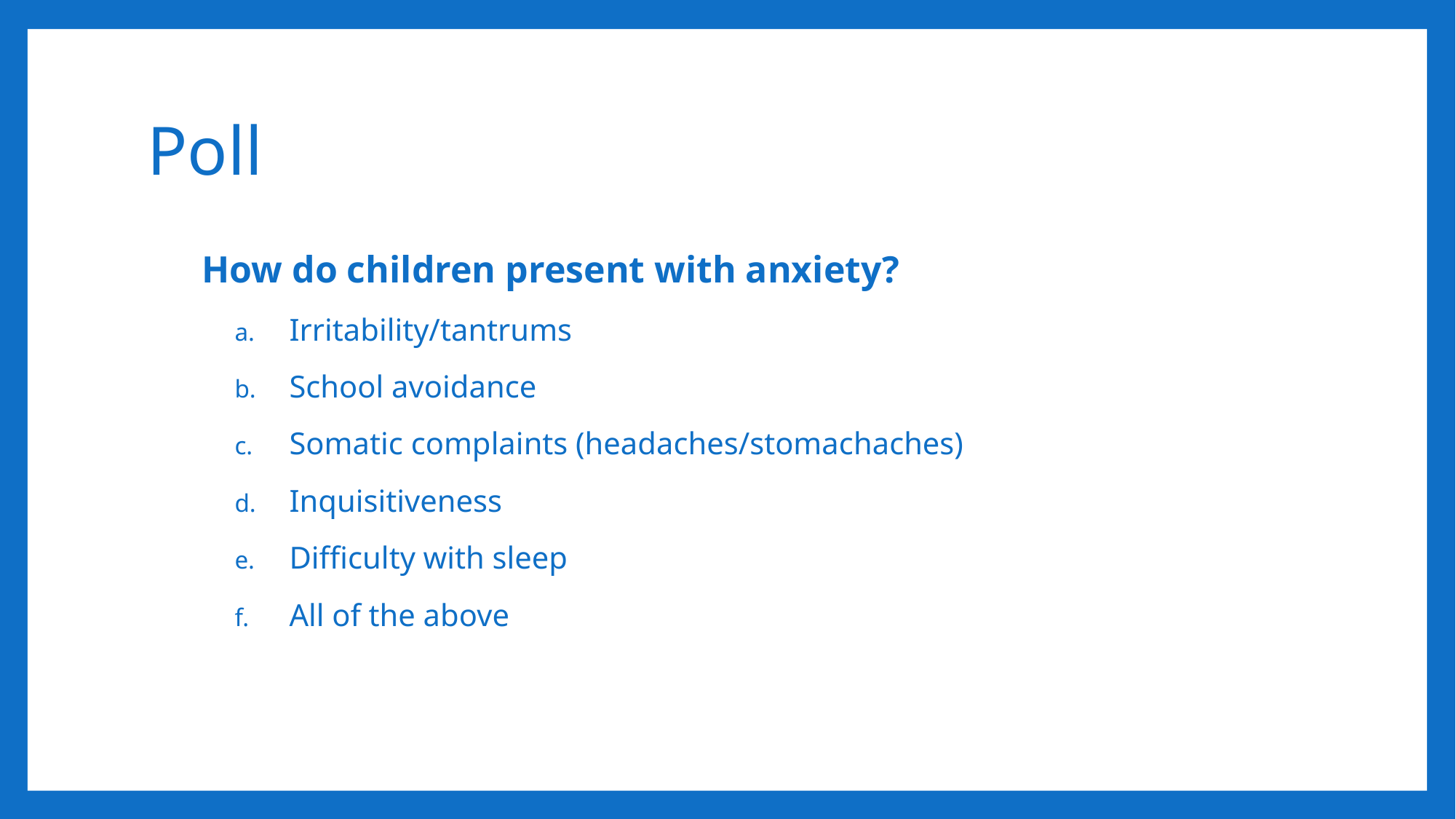

# Poll
How do children present with anxiety?
Irritability/tantrums
School avoidance
Somatic complaints (headaches/stomachaches)
Inquisitiveness
Difficulty with sleep
All of the above

## Slide 6
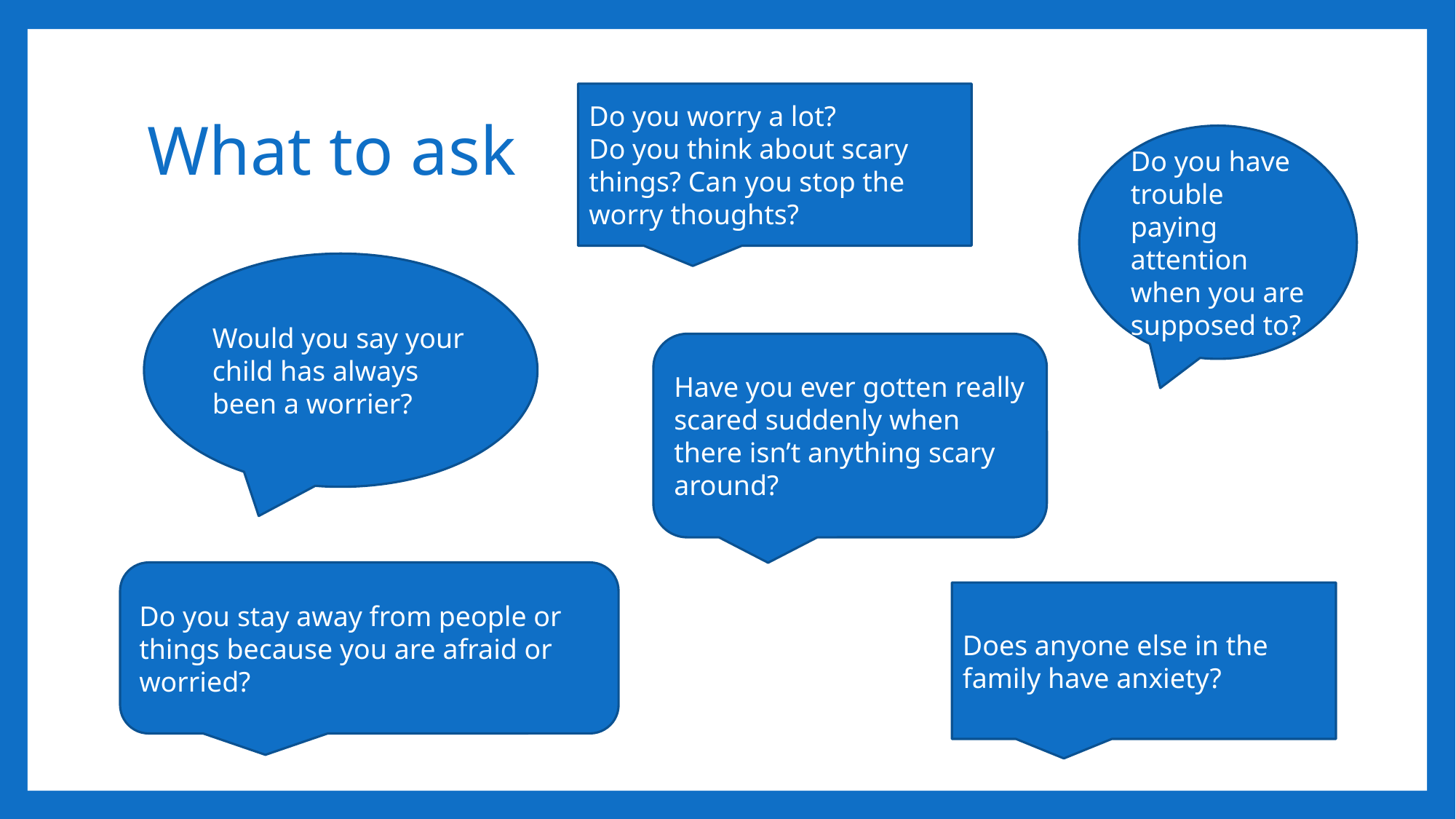

# What to ask
Do you worry a lot?
Do you think about scary things? Can you stop the worry thoughts?
Do you have trouble paying attention when you are supposed to?
Would you say your child has always been a worrier?
Have you ever gotten really scared suddenly when there isn’t anything scary around?
Do you stay away from people or things because you are afraid or worried?
Does anyone else in the family have anxiety?

## Slide 7
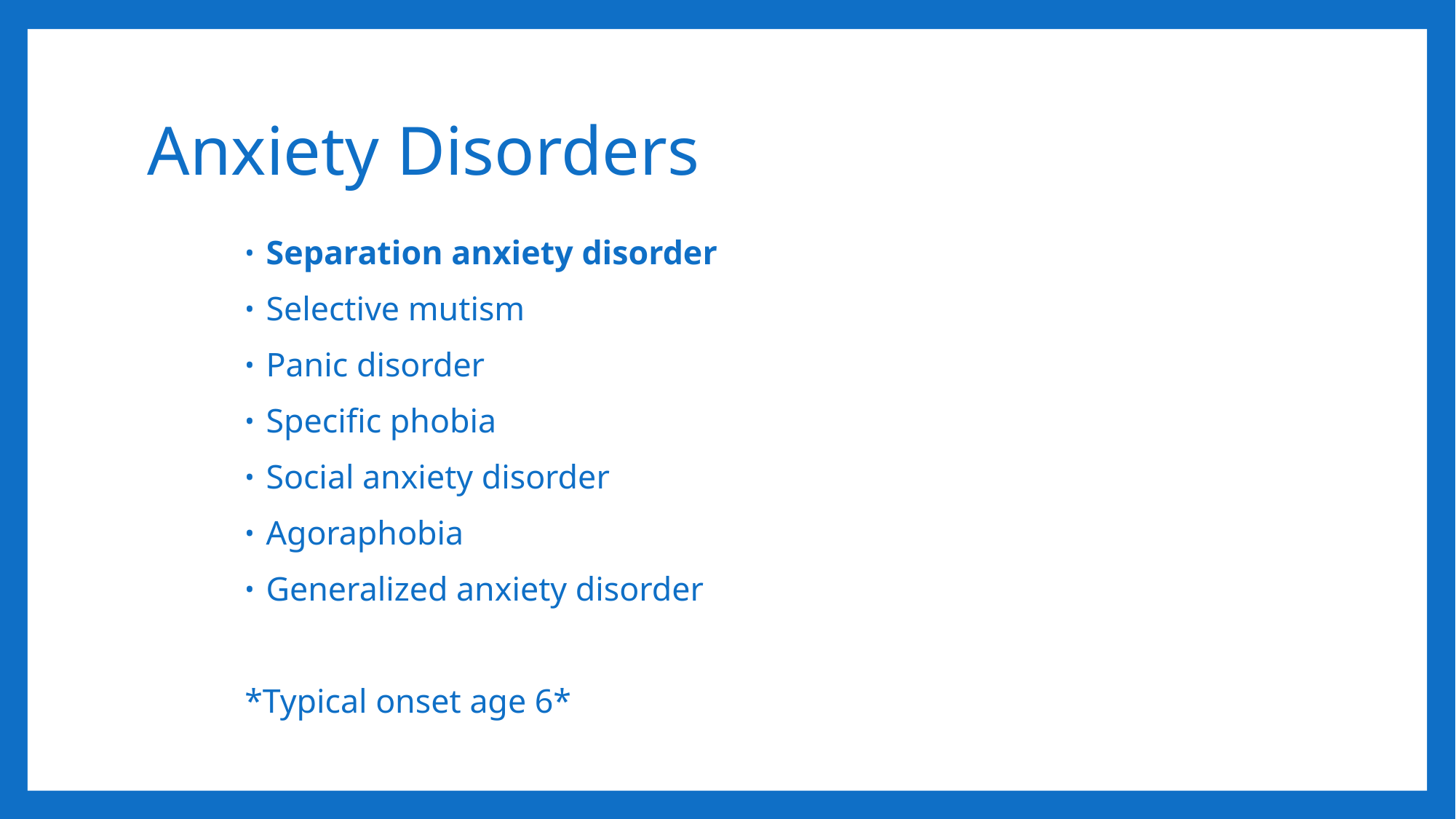

# Anxiety Disorders
Separation anxiety disorder
Selective mutism
Panic disorder
Specific phobia
Social anxiety disorder
Agoraphobia
Generalized anxiety disorder
*Typical onset age 6*

## Slide 8
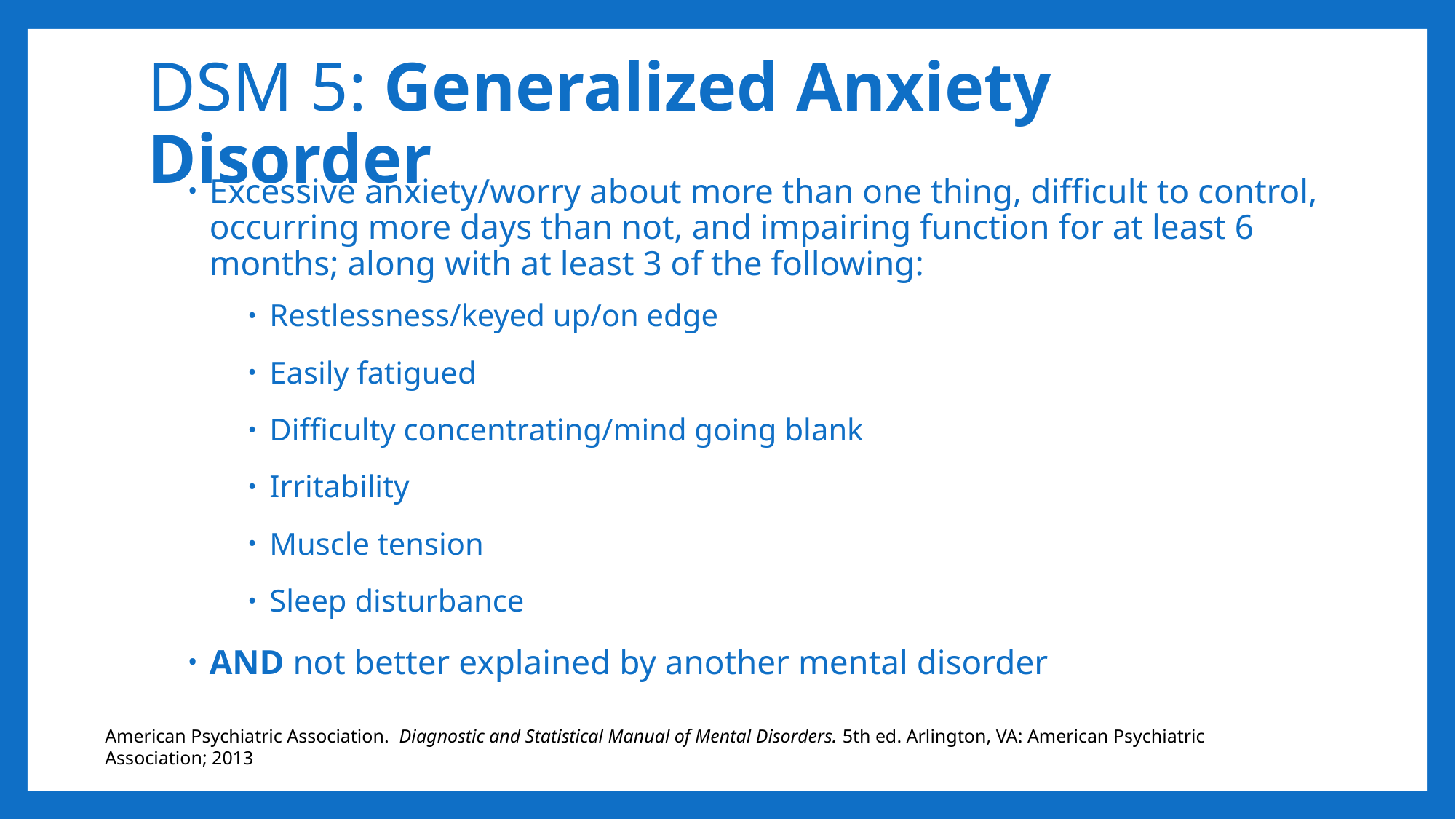

# DSM 5: Generalized Anxiety Disorder
Excessive anxiety/worry about more than one thing, difficult to control, occurring more days than not, and impairing function for at least 6 months; along with at least 3 of the following:
Restlessness/keyed up/on edge
Easily fatigued
Difficulty concentrating/mind going blank
Irritability
Muscle tension
Sleep disturbance
AND not better explained by another mental disorder
American Psychiatric Association.  Diagnostic and Statistical Manual of Mental Disorders. 5th ed. Arlington, VA: American Psychiatric Association; 2013

## Slide 9
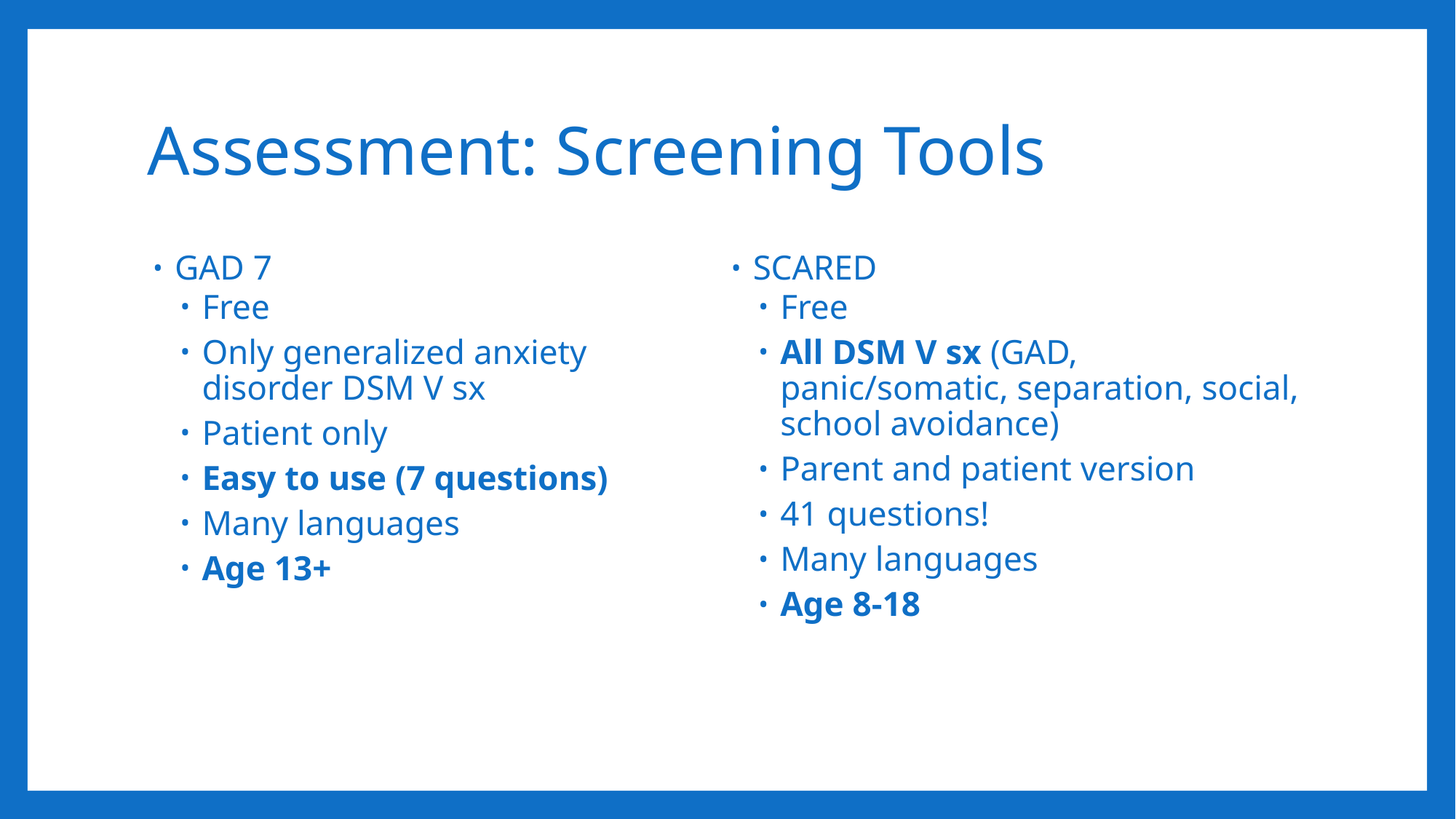

# Assessment: Screening Tools
GAD 7
Free
Only generalized anxiety disorder DSM V sx
Patient only
Easy to use (7 questions)
Many languages
Age 13+
SCARED
Free
All DSM V sx (GAD, panic/somatic, separation, social, school avoidance)
Parent and patient version
41 questions!
Many languages
Age 8-18

## Slide 10
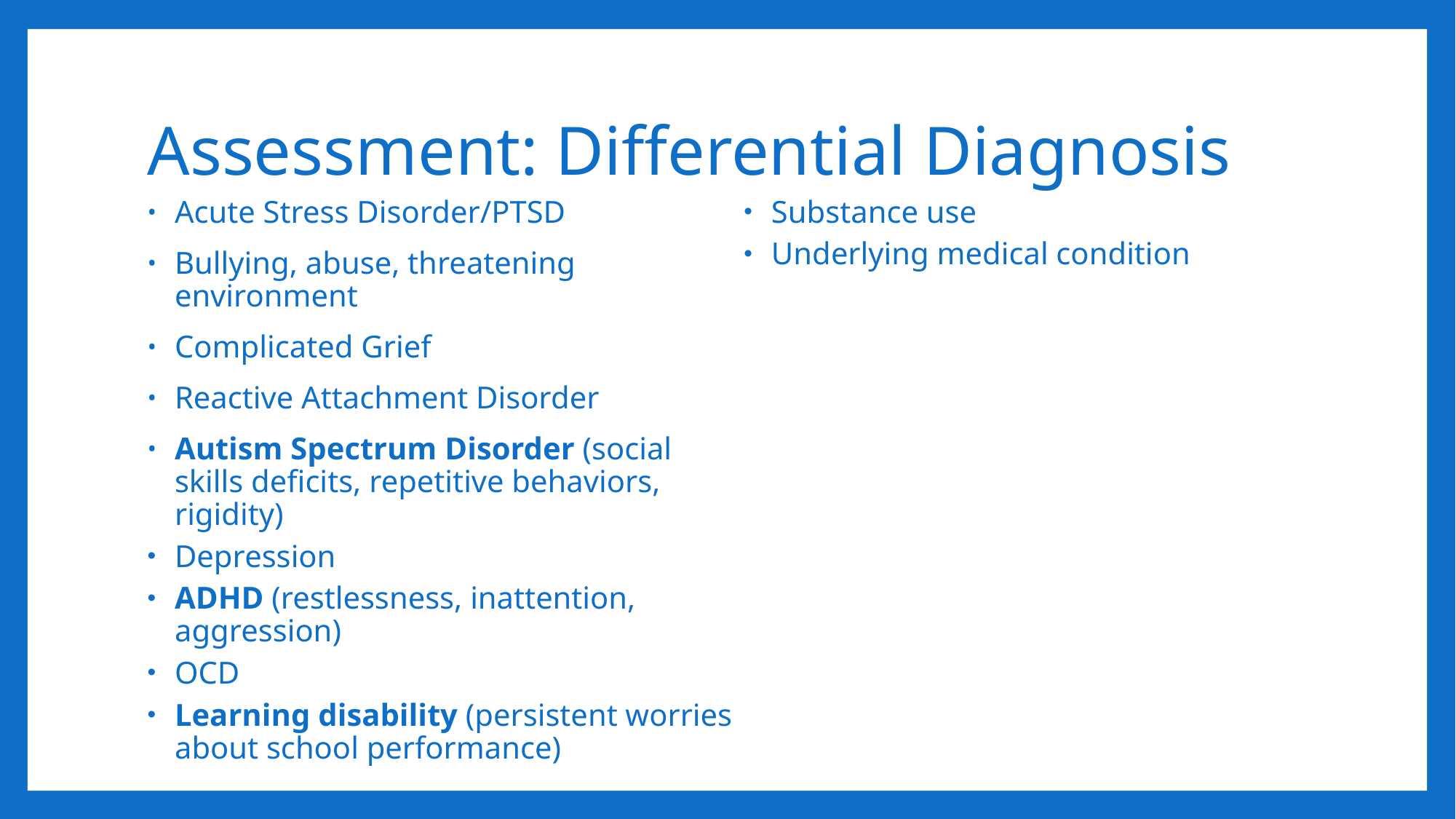

# Assessment: Differential Diagnosis
Acute Stress Disorder/PTSD
Bullying, abuse, threatening environment
Complicated Grief
Reactive Attachment Disorder
Autism Spectrum Disorder (social skills deficits, repetitive behaviors, rigidity)
Depression
ADHD (restlessness, inattention, aggression)
OCD
Learning disability (persistent worries about school performance)
Substance use
Underlying medical condition

## Slide 11
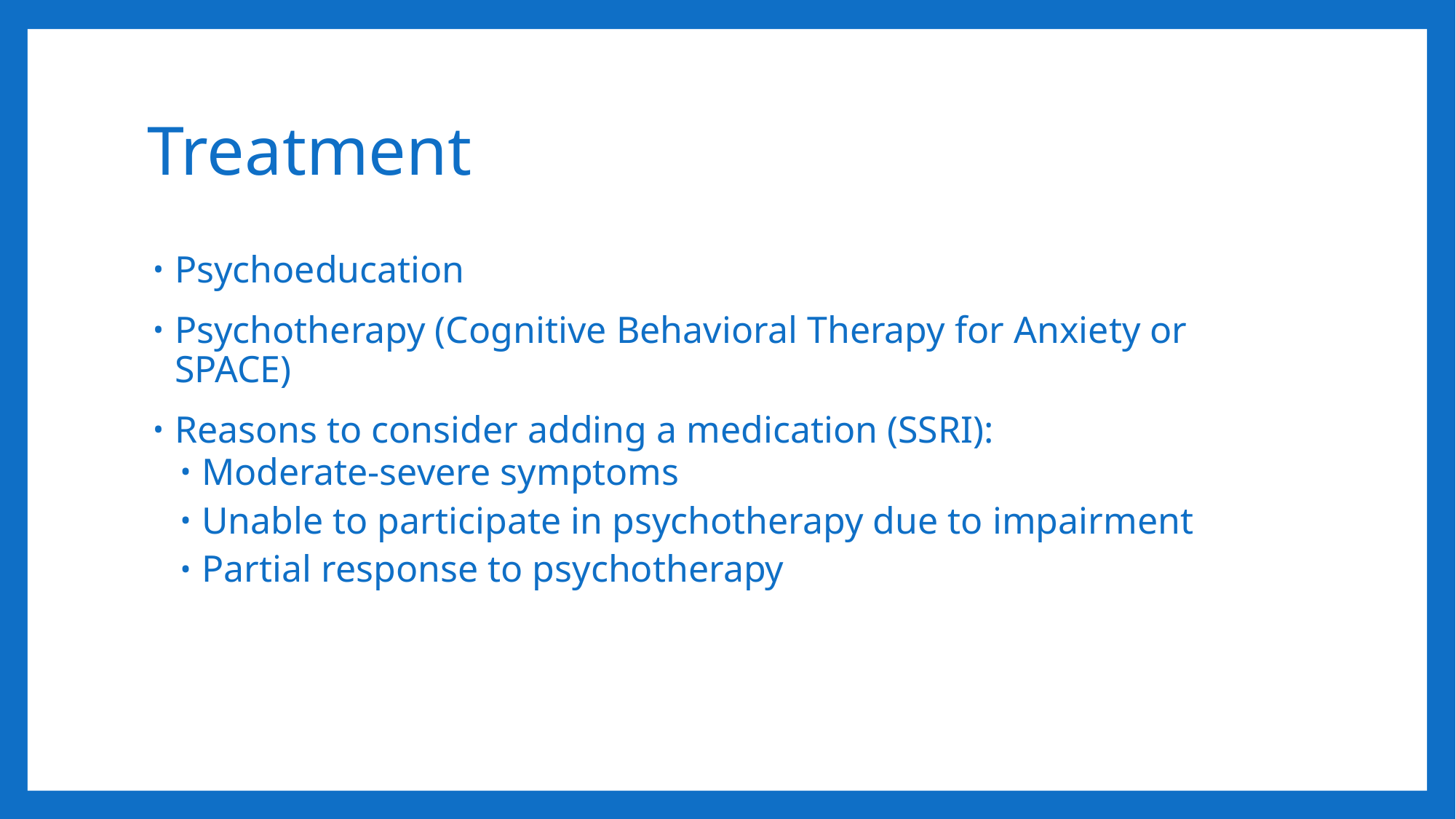

# Treatment
Psychoeducation
Psychotherapy (Cognitive Behavioral Therapy for Anxiety or SPACE)
Reasons to consider adding a medication (SSRI):
Moderate-severe symptoms
Unable to participate in psychotherapy due to impairment
Partial response to psychotherapy

## Slide 12
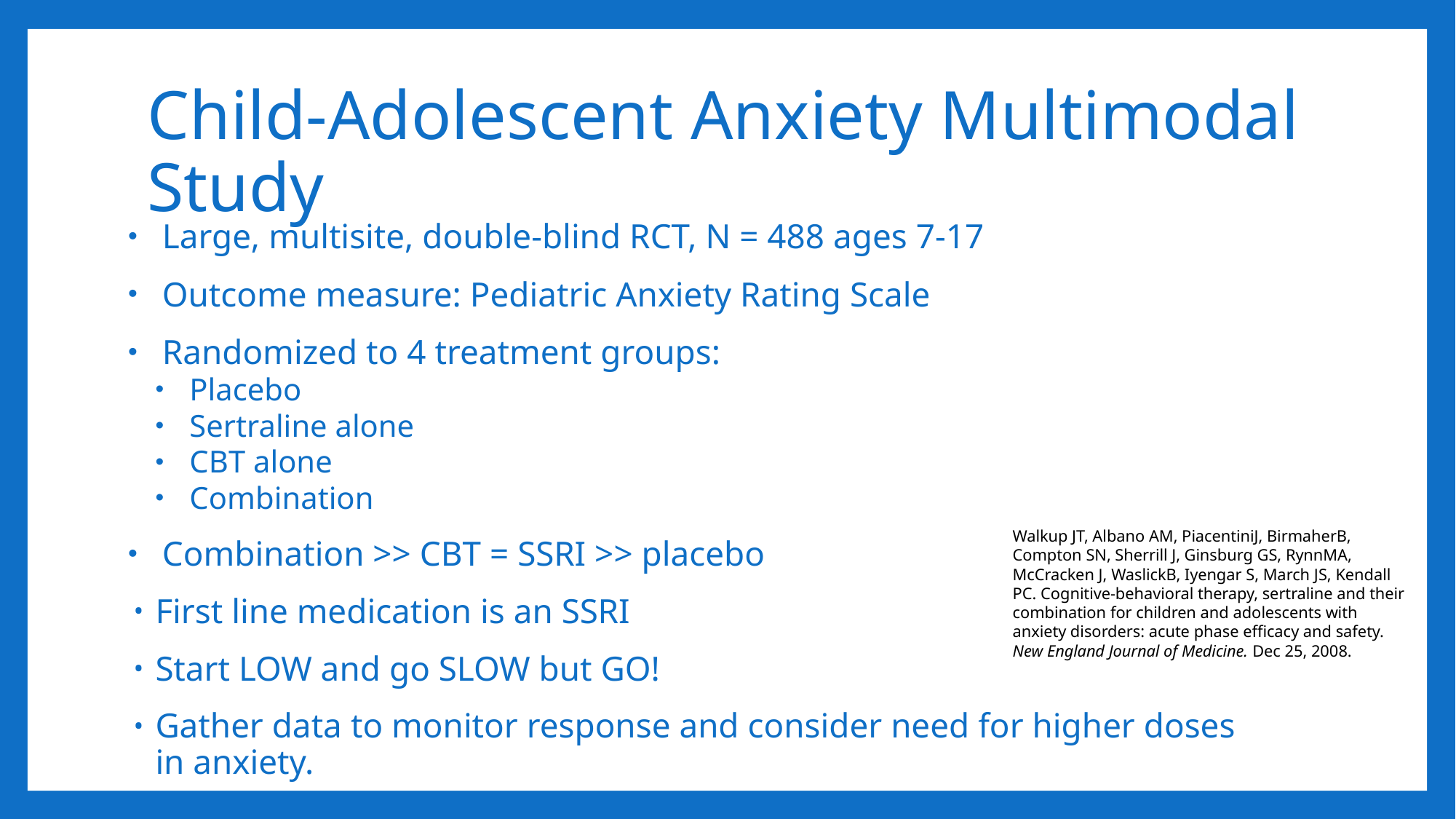

# Child-Adolescent Anxiety Multimodal Study
Large, multisite, double-blind RCT, N = 488 ages 7-17
Outcome measure: Pediatric Anxiety Rating Scale
Randomized to 4 treatment groups:
Placebo
Sertraline alone
CBT alone
Combination
Combination >> CBT = SSRI >> placebo
First line medication is an SSRI
Start LOW and go SLOW but GO!
Gather data to monitor response and consider need for higher doses in anxiety.
Walkup JT, Albano AM, PiacentiniJ, BirmaherB, Compton SN, Sherrill J, Ginsburg GS, RynnMA, McCracken J, WaslickB, Iyengar S, March JS, Kendall PC. Cognitive-behavioral therapy, sertraline and their combination for children and adolescents with anxiety disorders: acute phase efficacy and safety. New England Journal of Medicine. Dec 25, 2008.

## Slide 13
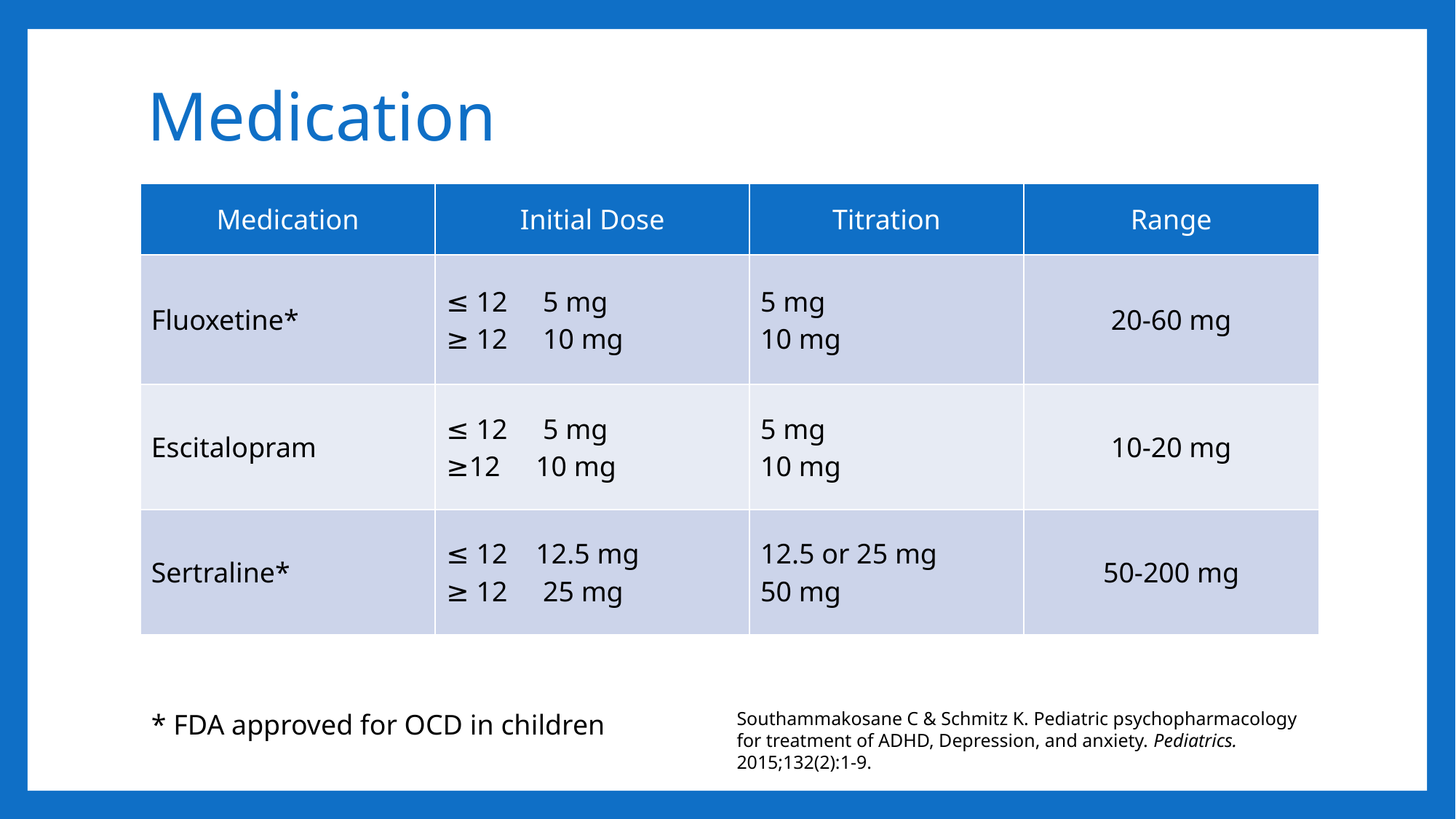

# Medication
| Medication​ | Initial Dose​ | Titration​ | Range​ |
| --- | --- | --- | --- |
| Fluoxetine\*​ | ≤ 12     5 mg​ ≥ 12     10 mg​ | 5 mg​ 10 mg​ | 20-60 mg​ |
| Escitalopram | ≤ 12     5 mg​ ≥12     10 mg​ | 5 mg​ 10 mg​ | 10-20 mg​ |
| Sertraline\*​ | ≤ 12    12.5 mg​ ≥ 12     25 mg​ | 12.5 or 25 mg​ 50 mg​ | 50-200 mg​ |
Southammakosane C & Schmitz K. Pediatric psychopharmacology for treatment of ADHD, Depression, and anxiety. Pediatrics. 2015;132(2):1-9.
* FDA approved for OCD in children

## Slide 14
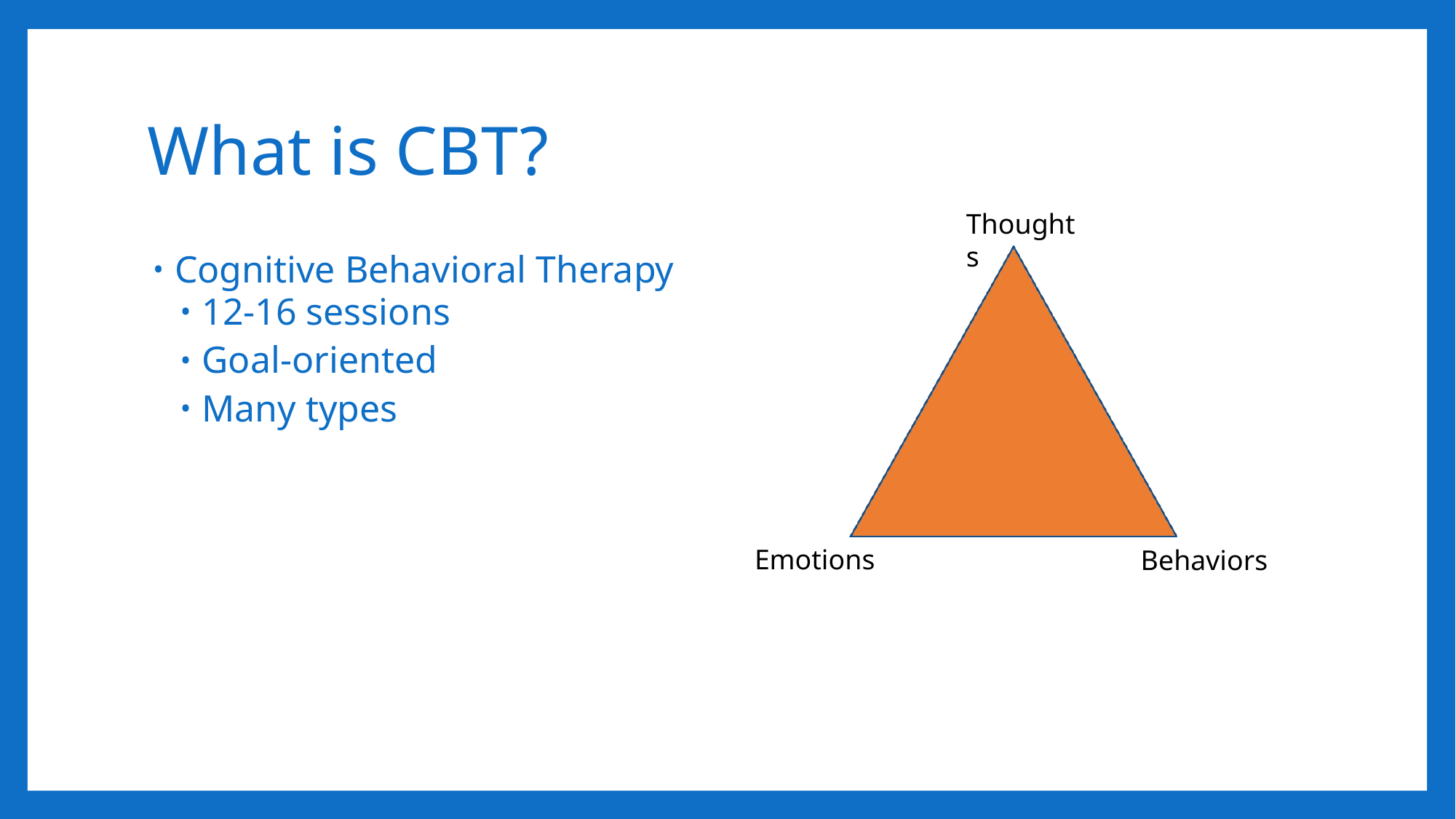

# What is CBT?
Thoughts
Cognitive Behavioral Therapy
12-16 sessions
Goal-oriented
Many types
Emotions
Behaviors

## Slide 15
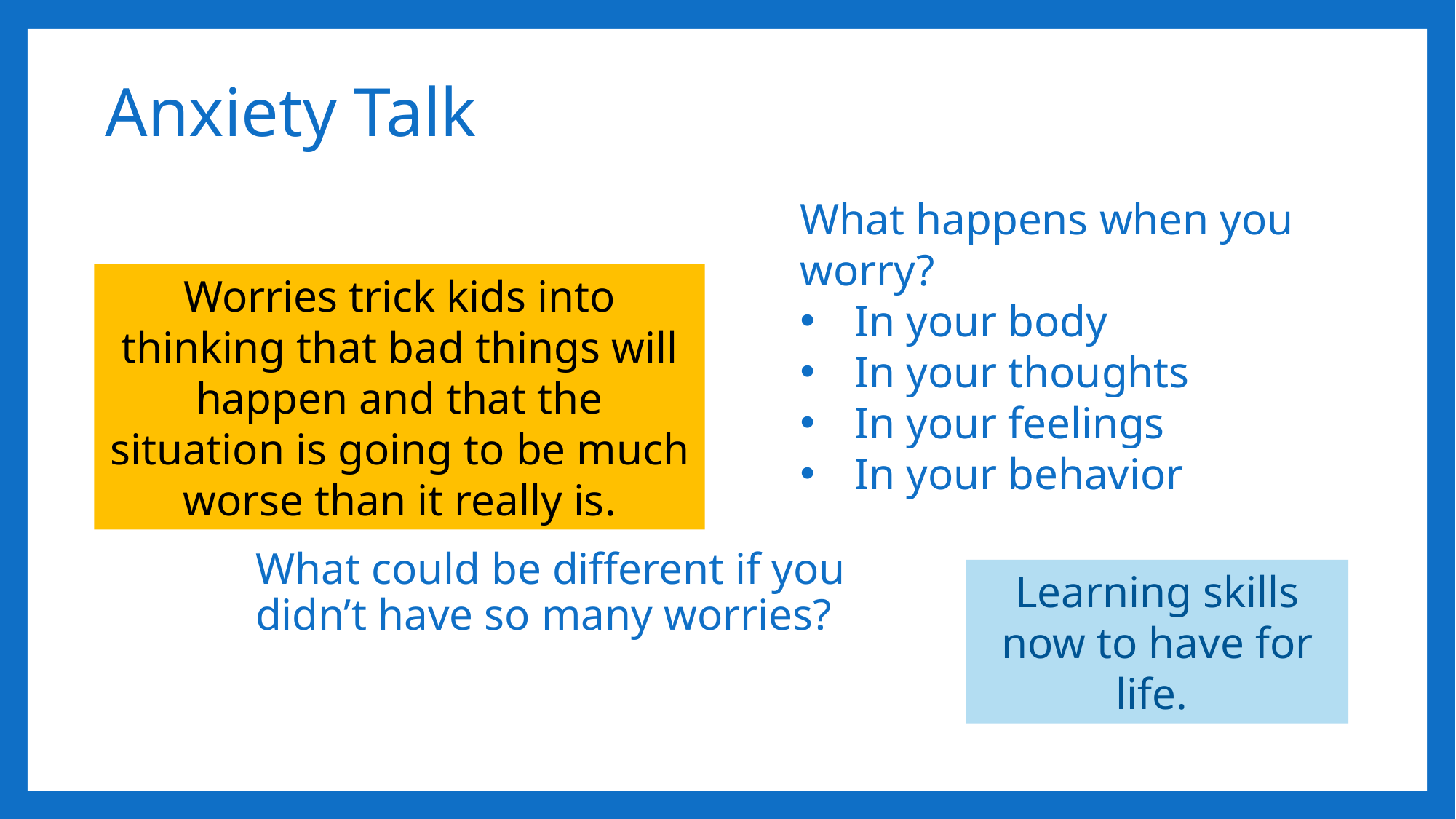

# Anxiety Talk
What happens when you worry?
In your body
In your thoughts
In your feelings
In your behavior
Worries trick kids into thinking that bad things will happen and that the situation is going to be much worse than it really is.
What could be different if you didn’t have so many worries?
Learning skills now to have for life.

## Slide 16
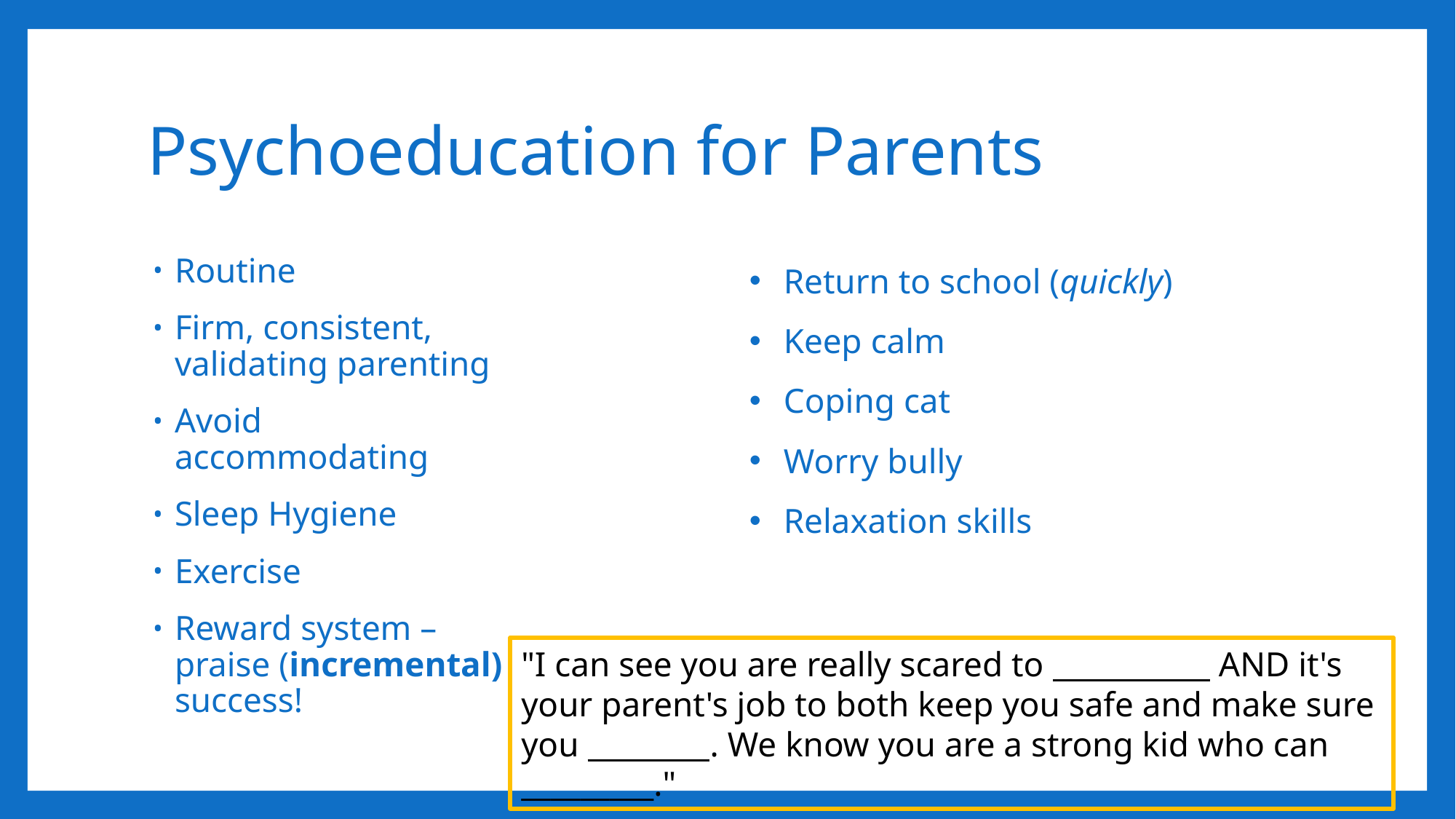

# Psychoeducation for Parents
Return to school (quickly)
Keep calm
Coping cat
Worry bully
Relaxation skills
Routine
Firm, consistent, validating parenting
Avoid accommodating
Sleep Hygiene
Exercise
Reward system – praise (incremental) success!
"I can see you are really scared to                    AND it's your parent's job to both keep you safe and make sure you               . We know you are a strong kid who can _________."

## Slide 17
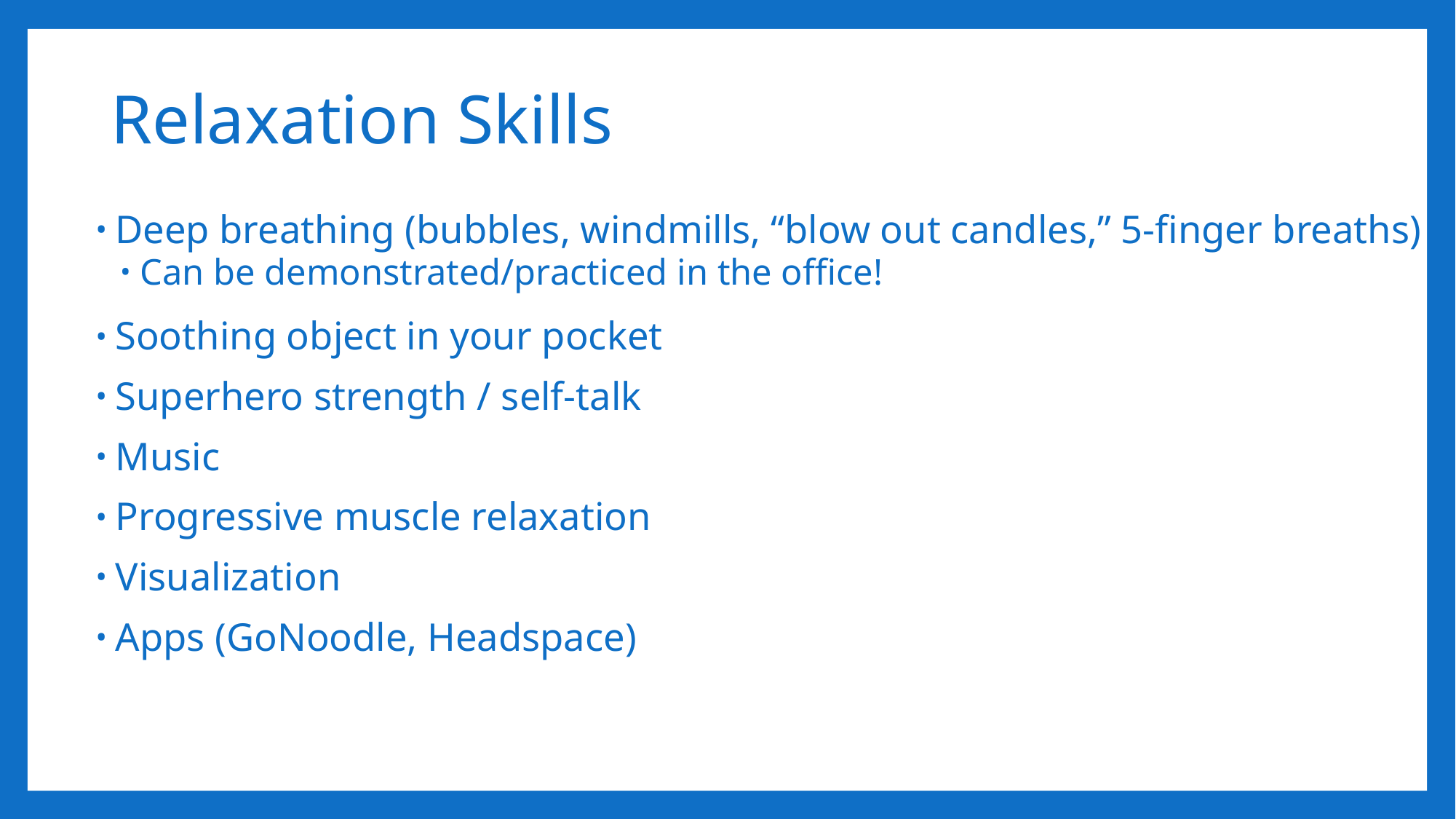

# Relaxation Skills
Deep breathing (bubbles, windmills, “blow out candles,” 5-finger breaths)
Can be demonstrated/practiced in the office!
Soothing object in your pocket
Superhero strength / self-talk
Music
Progressive muscle relaxation
Visualization
Apps (GoNoodle, Headspace)

## Slide 18
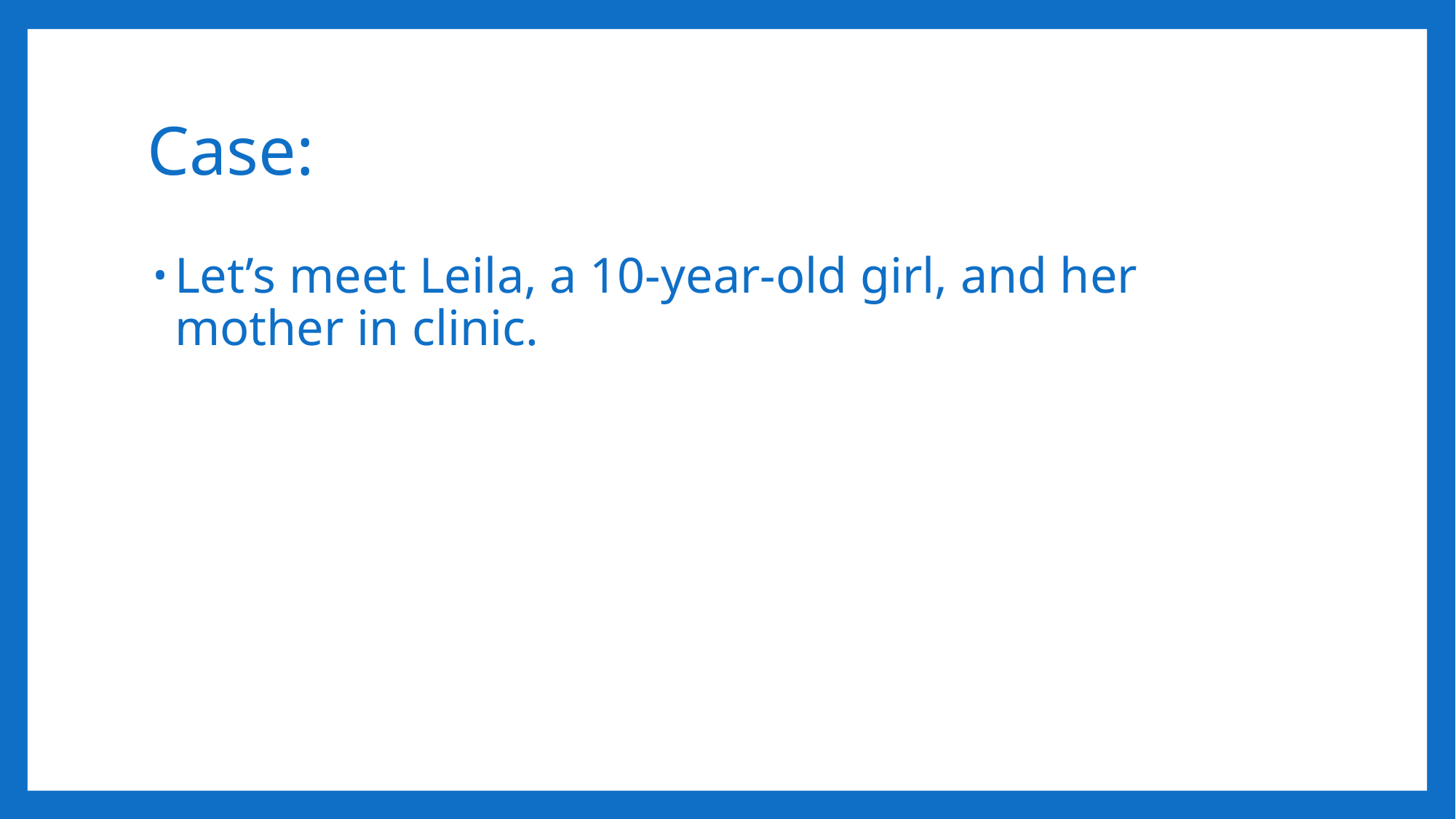

# Case:
Let’s meet Leila, a 10-year-old girl, and her mother in clinic.

## Slide 19
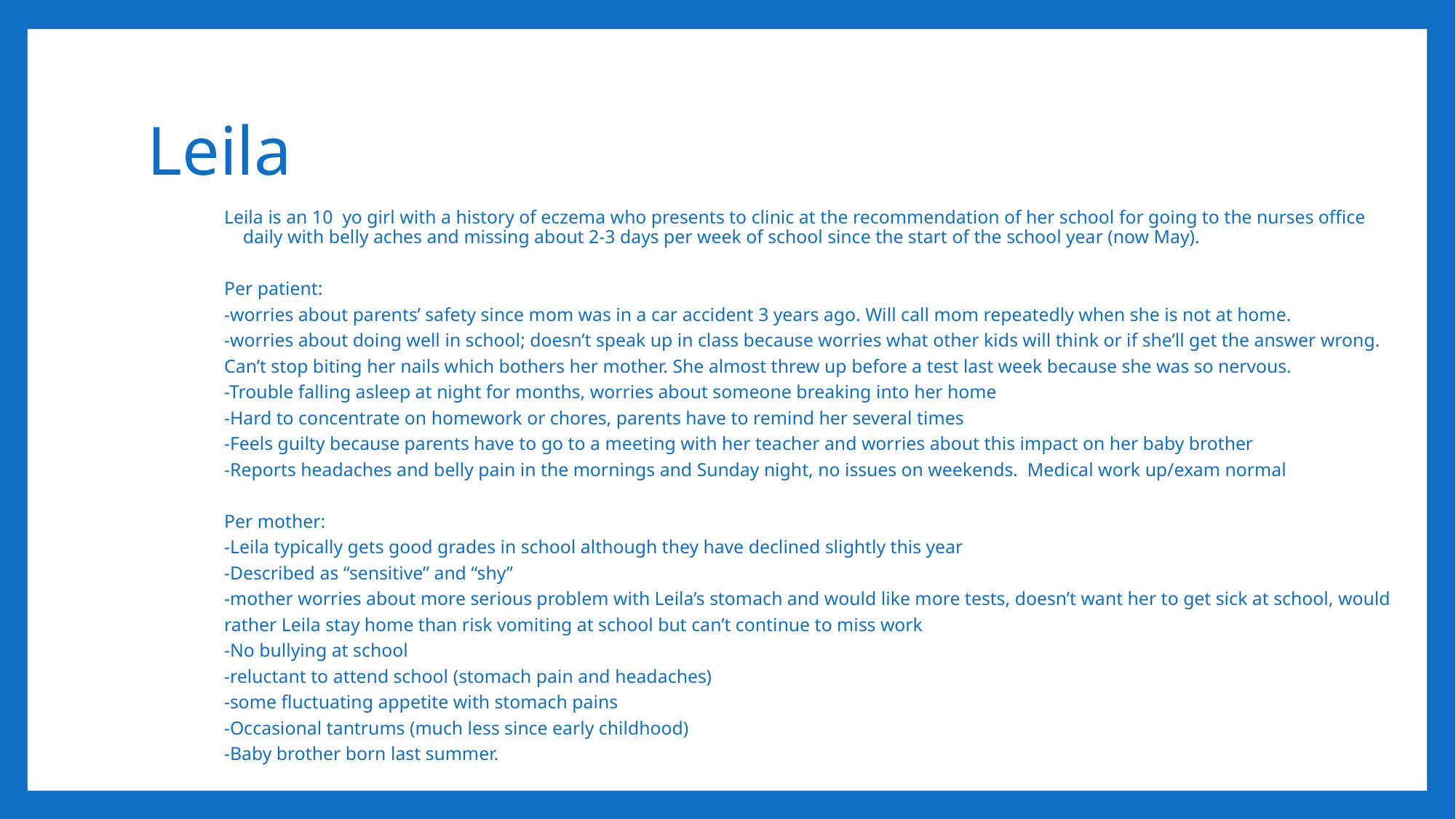

# Leila
Leila is an 10  yo girl with a history of eczema who presents to clinic at the recommendation of her school for going to the nurses office daily with belly aches and missing about 2-3 days per week of school since the start of the school year (now May).
Per patient:
-worries about parents’ safety since mom was in a car accident 3 years ago. Will call mom repeatedly when she is not at home.
-worries about doing well in school; doesn’t speak up in class because worries what other kids will think or if she’ll get the answer wrong.  Can’t stop biting her nails which bothers her mother. She almost threw up before a test last week because she was so nervous. 
-Trouble falling asleep at night for months, worries about someone breaking into her home 
-Hard to concentrate on homework or chores, parents have to remind her several times
-Feels guilty because parents have to go to a meeting with her teacher and worries about this impact on her baby brother
-Reports headaches and belly pain in the mornings and Sunday night, no issues on weekends.  Medical work up/exam normal
Per mother:
-Leila typically gets good grades in school although they have declined slightly this year
-Described as “sensitive” and “shy”
-mother worries about more serious problem with Leila’s stomach and would like more tests, doesn’t want her to get sick at school, would rather Leila stay home than risk vomiting at school but can’t continue to miss work
-No bullying at school
-reluctant to attend school (stomach pain and headaches)
-some fluctuating appetite with stomach pains
-Occasional tantrums (much less since early childhood)
-Baby brother born last summer.

## Slide 20
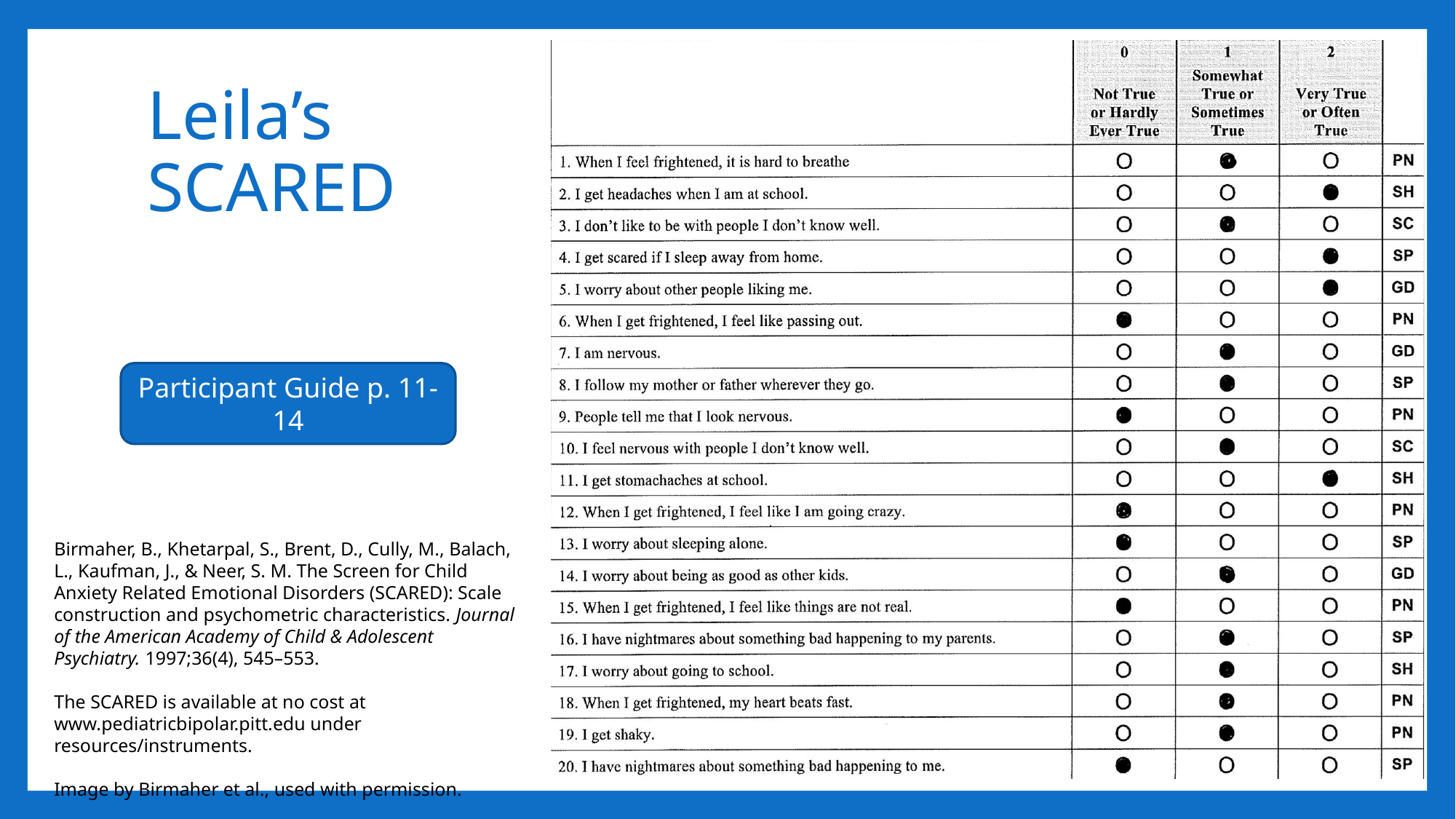

# Leila’s SCARED
Participant Guide p. 11-14
Birmaher, B., Khetarpal, S., Brent, D., Cully, M., Balach, L., Kaufman, J., & Neer, S. M. The Screen for Child Anxiety Related Emotional Disorders (SCARED): Scale construction and psychometric characteristics. Journal of the American Academy of Child & Adolescent Psychiatry. 1997;36(4), 545–553.
The SCARED is available at no cost at www.pediatricbipolar.pitt.edu under resources/instruments.
Image by Birmaher et al., used with permission.

## Slide 21
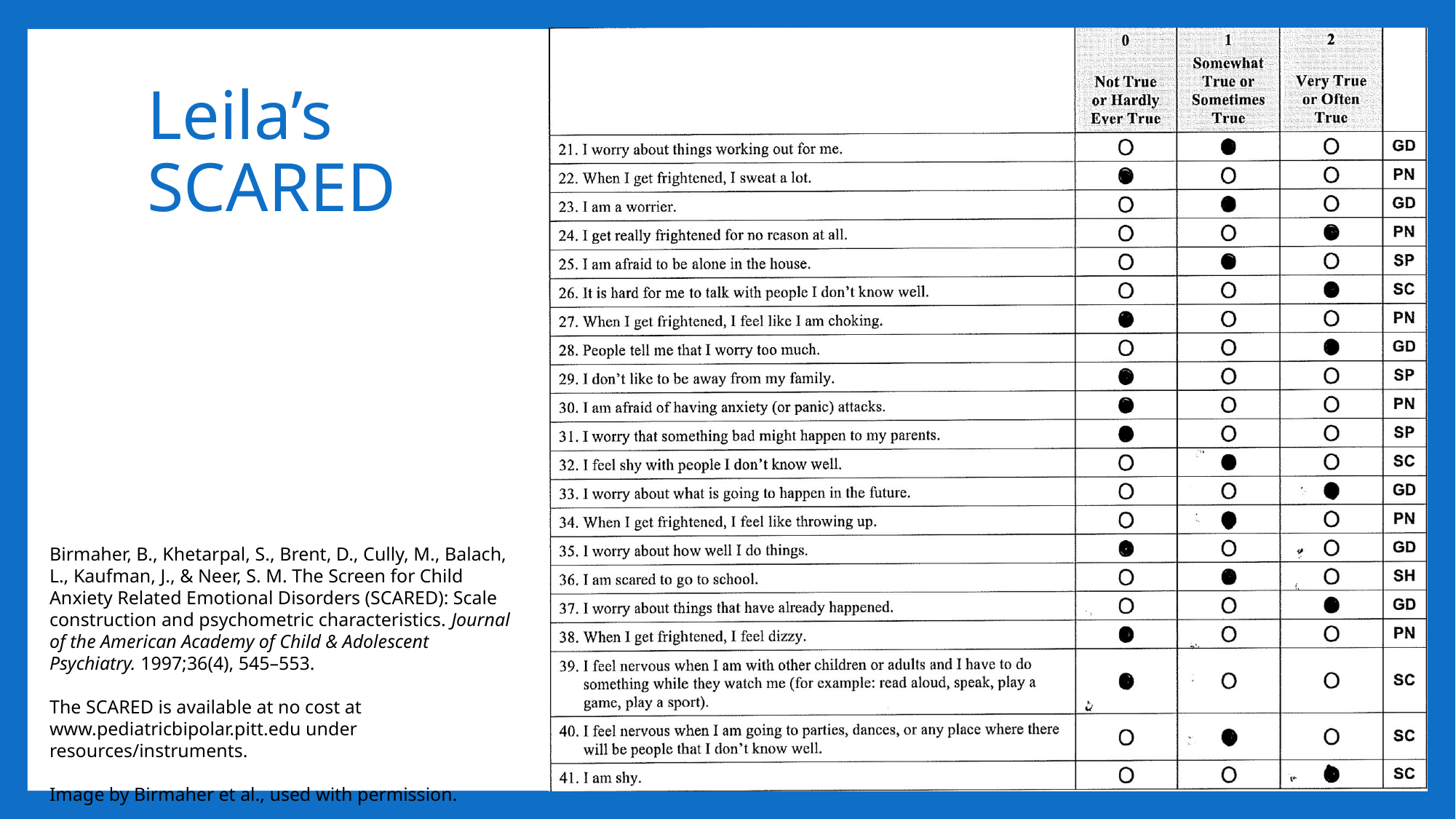

# Leila’s SCARED
Birmaher, B., Khetarpal, S., Brent, D., Cully, M., Balach, L., Kaufman, J., & Neer, S. M. The Screen for Child Anxiety Related Emotional Disorders (SCARED): Scale construction and psychometric characteristics. Journal of the American Academy of Child & Adolescent Psychiatry. 1997;36(4), 545–553.
The SCARED is available at no cost at www.pediatricbipolar.pitt.edu under resources/instruments.
Image by Birmaher et al., used with permission.

## Slide 22
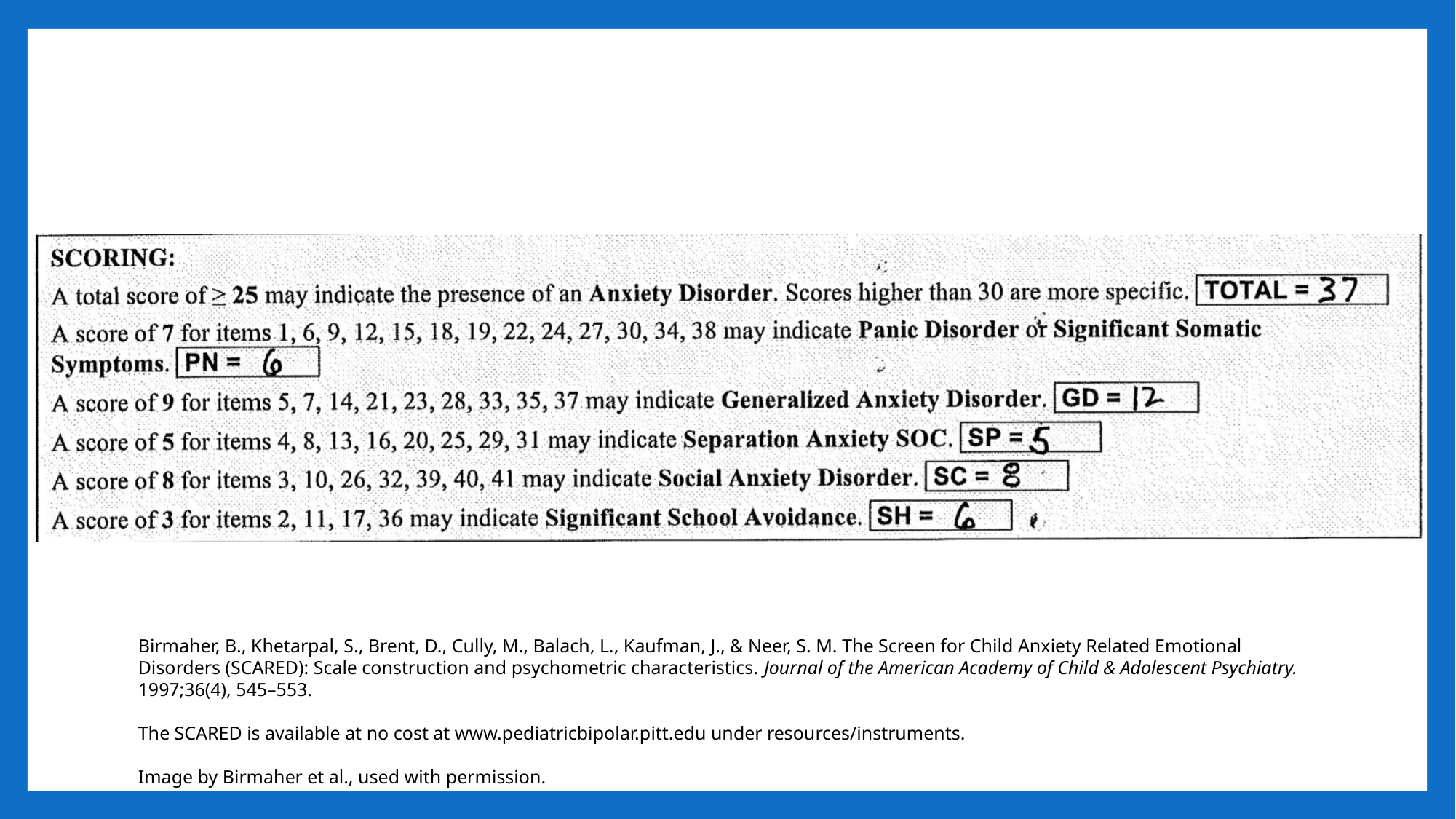

Birmaher, B., Khetarpal, S., Brent, D., Cully, M., Balach, L., Kaufman, J., & Neer, S. M. The Screen for Child Anxiety Related Emotional Disorders (SCARED): Scale construction and psychometric characteristics. Journal of the American Academy of Child & Adolescent Psychiatry. 1997;36(4), 545–553.
The SCARED is available at no cost at www.pediatricbipolar.pitt.edu under resources/instruments.
Image by Birmaher et al., used with permission.

## Slide 23
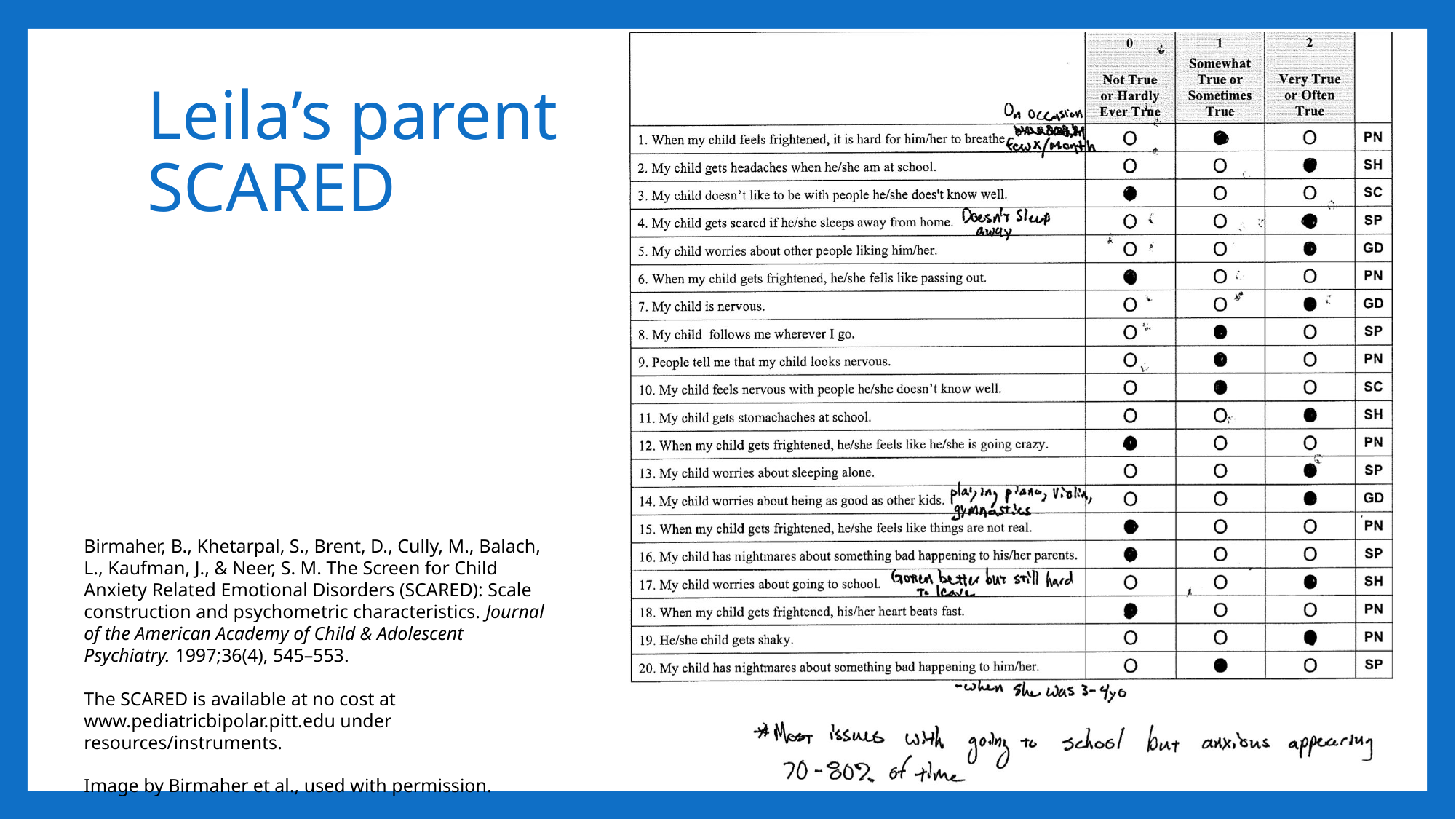

# Leila’s parent SCARED
Birmaher, B., Khetarpal, S., Brent, D., Cully, M., Balach, L., Kaufman, J., & Neer, S. M. The Screen for Child Anxiety Related Emotional Disorders (SCARED): Scale construction and psychometric characteristics. Journal of the American Academy of Child & Adolescent Psychiatry. 1997;36(4), 545–553.
The SCARED is available at no cost at www.pediatricbipolar.pitt.edu under resources/instruments.
Image by Birmaher et al., used with permission.

## Slide 24
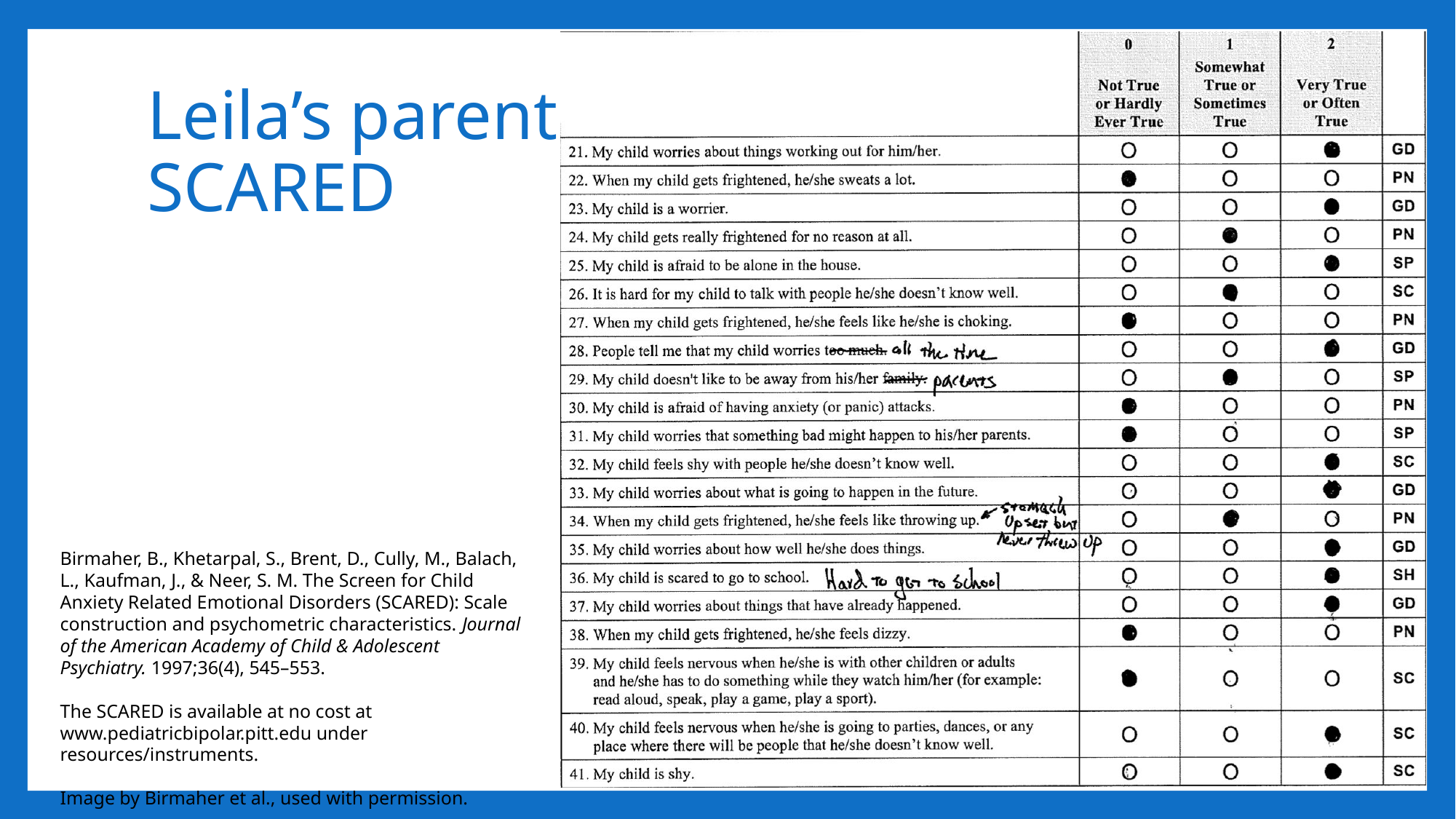

# Leila’s parent SCARED
Birmaher, B., Khetarpal, S., Brent, D., Cully, M., Balach, L., Kaufman, J., & Neer, S. M. The Screen for Child Anxiety Related Emotional Disorders (SCARED): Scale construction and psychometric characteristics. Journal of the American Academy of Child & Adolescent Psychiatry. 1997;36(4), 545–553.
The SCARED is available at no cost at www.pediatricbipolar.pitt.edu under resources/instruments.
Image by Birmaher et al., used with permission.

## Slide 25
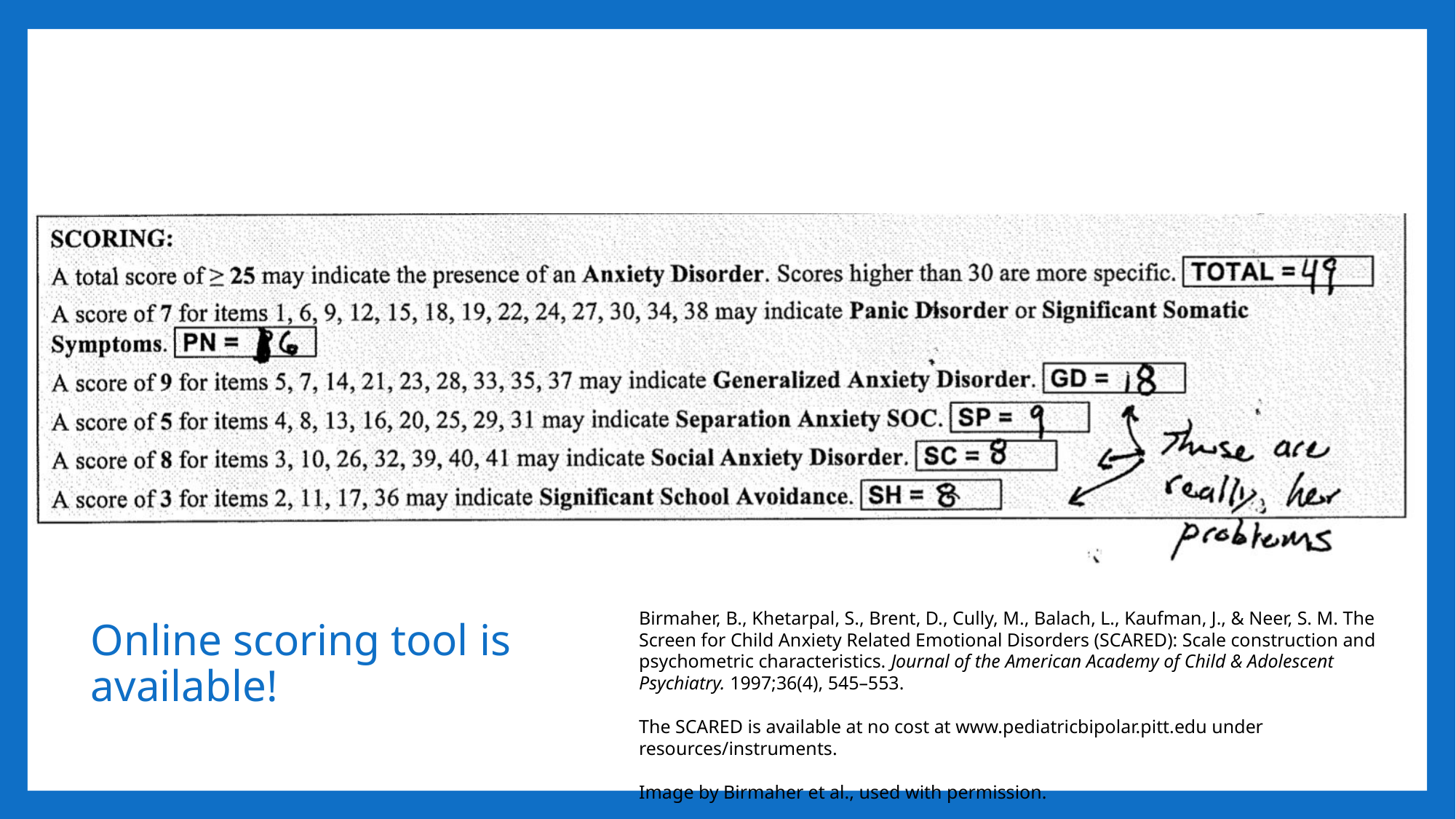

# Online scoring tool is available!
Birmaher, B., Khetarpal, S., Brent, D., Cully, M., Balach, L., Kaufman, J., & Neer, S. M. The Screen for Child Anxiety Related Emotional Disorders (SCARED): Scale construction and psychometric characteristics. Journal of the American Academy of Child & Adolescent Psychiatry. 1997;36(4), 545–553.
The SCARED is available at no cost at www.pediatricbipolar.pitt.edu under resources/instruments.
Image by Birmaher et al., used with permission.

## Slide 26
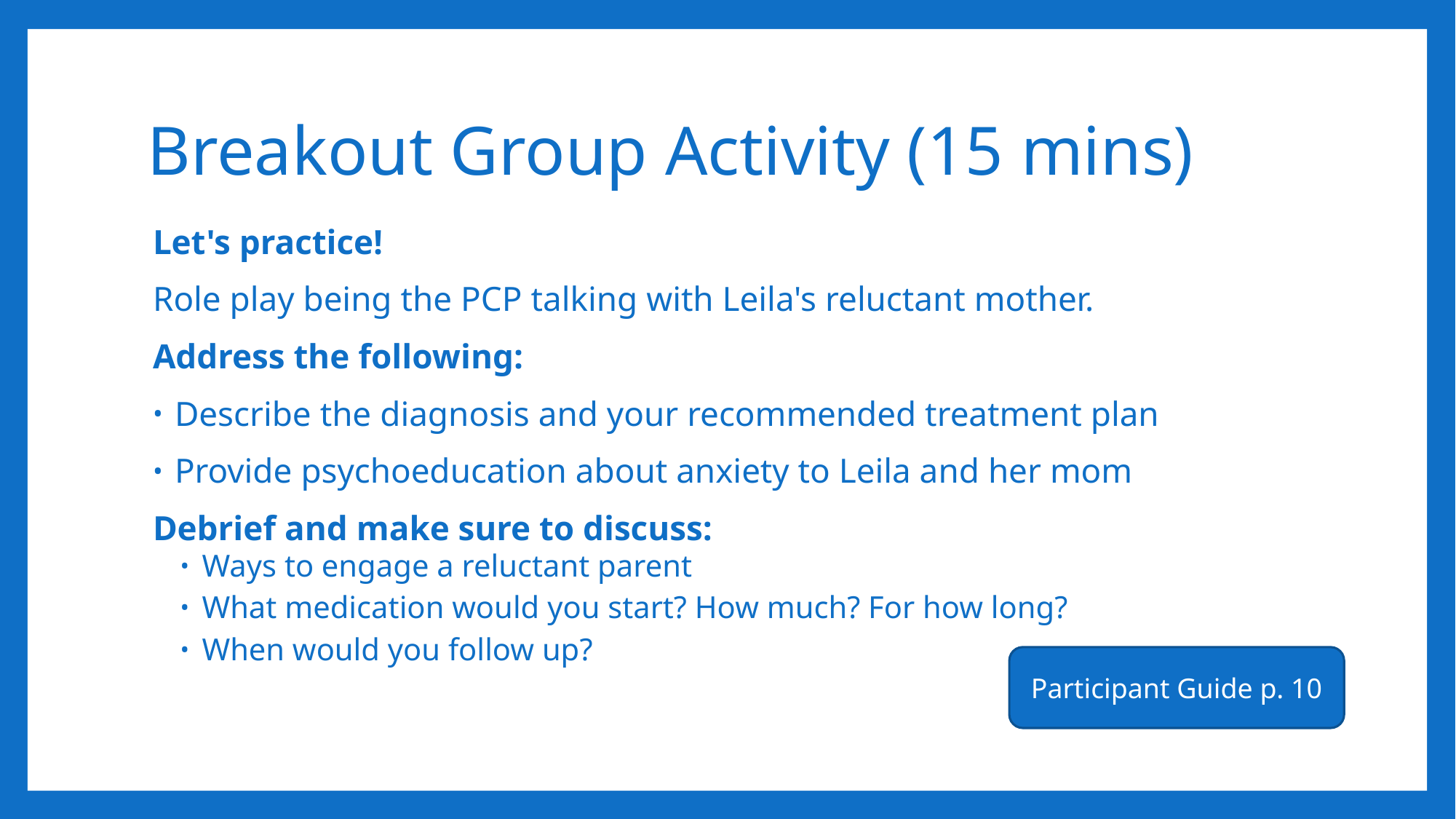

# Breakout Group Activity (15 mins)
Let's practice!
Role play being the PCP talking with Leila's reluctant mother.
Address the following:
Describe the diagnosis and your recommended treatment plan
Provide psychoeducation about anxiety to Leila and her mom
Debrief and make sure to discuss:
Ways to engage a reluctant parent
What medication would you start? How much? For how long?
When would you follow up?
Participant Guide p. 10

## Slide 27
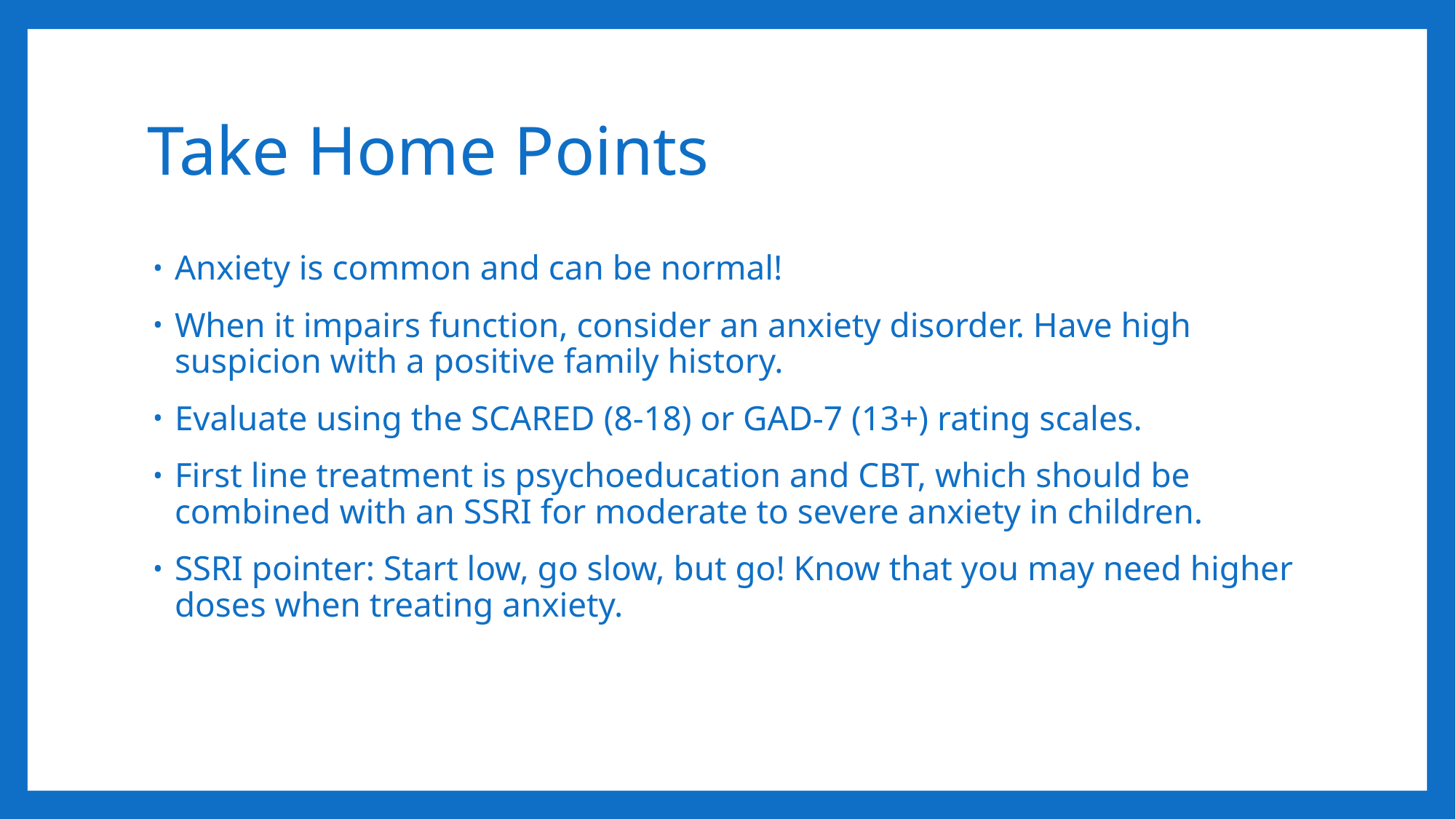

# Take Home Points
Anxiety is common and can be normal!
When it impairs function, consider an anxiety disorder. Have high suspicion with a positive family history.
Evaluate using the SCARED (8-18) or GAD-7 (13+) rating scales.
First line treatment is psychoeducation and CBT, which should be combined with an SSRI for moderate to severe anxiety in children.
SSRI pointer: Start low, go slow, but go! Know that you may need higher doses when treating anxiety.
