# Supplementary material for: Be ExPeRT (Behavioral Health Expansion in Pediatric Residency Training): A Case-Based Seminar
Source: MedEdPORTAL. 2023 Aug 1;19:11326. doi: 10.15766/mep_2374-8265.11326 (PMC10392710; doi:10.15766/mep_2374-8265.11326)
Supplement: Supplementary file 1 — Facilitator Guide.docxBe ExPeRT Introduction.pptxADHD in Primary Care Pediatrics.pptxAnxiety in Primary Care Pediatrics.pptxDepression in Primary Care Pediatrics.pptxBe ExPeRT Reference Slides.pptxParticipant Guide.docxBe ExPeRT Postsurvey.docxBe ExPeRT Case Discussion Form.docxBe ExPeRT Presurvey.docx [file mep_2374-8265.11326-s001.zip › E. Depression in Primary Care Pediatrics.pptx]

## Slide 1
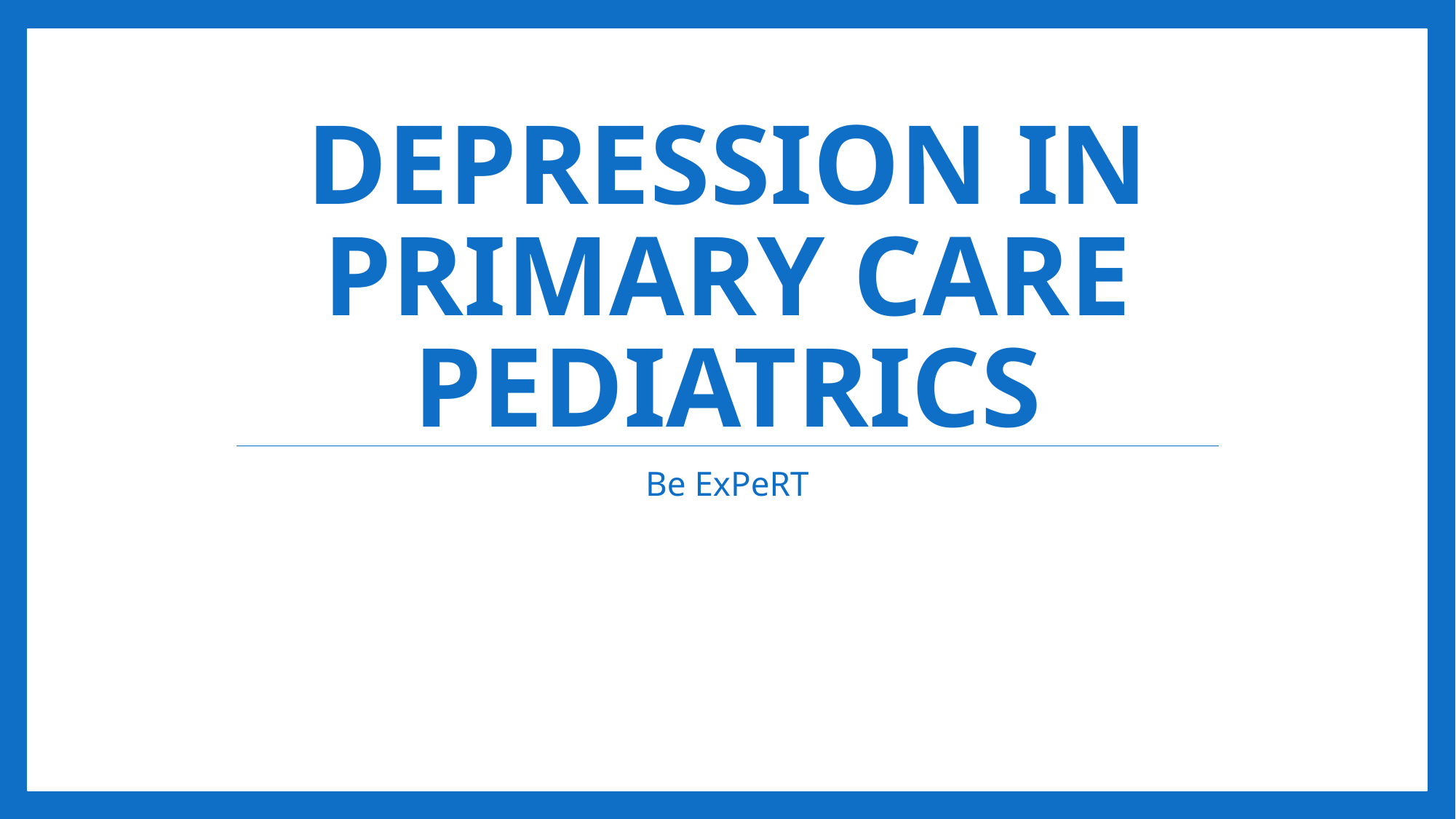

# Depression in Primary Care Pediatrics
Be ExPeRT

## Slide 2
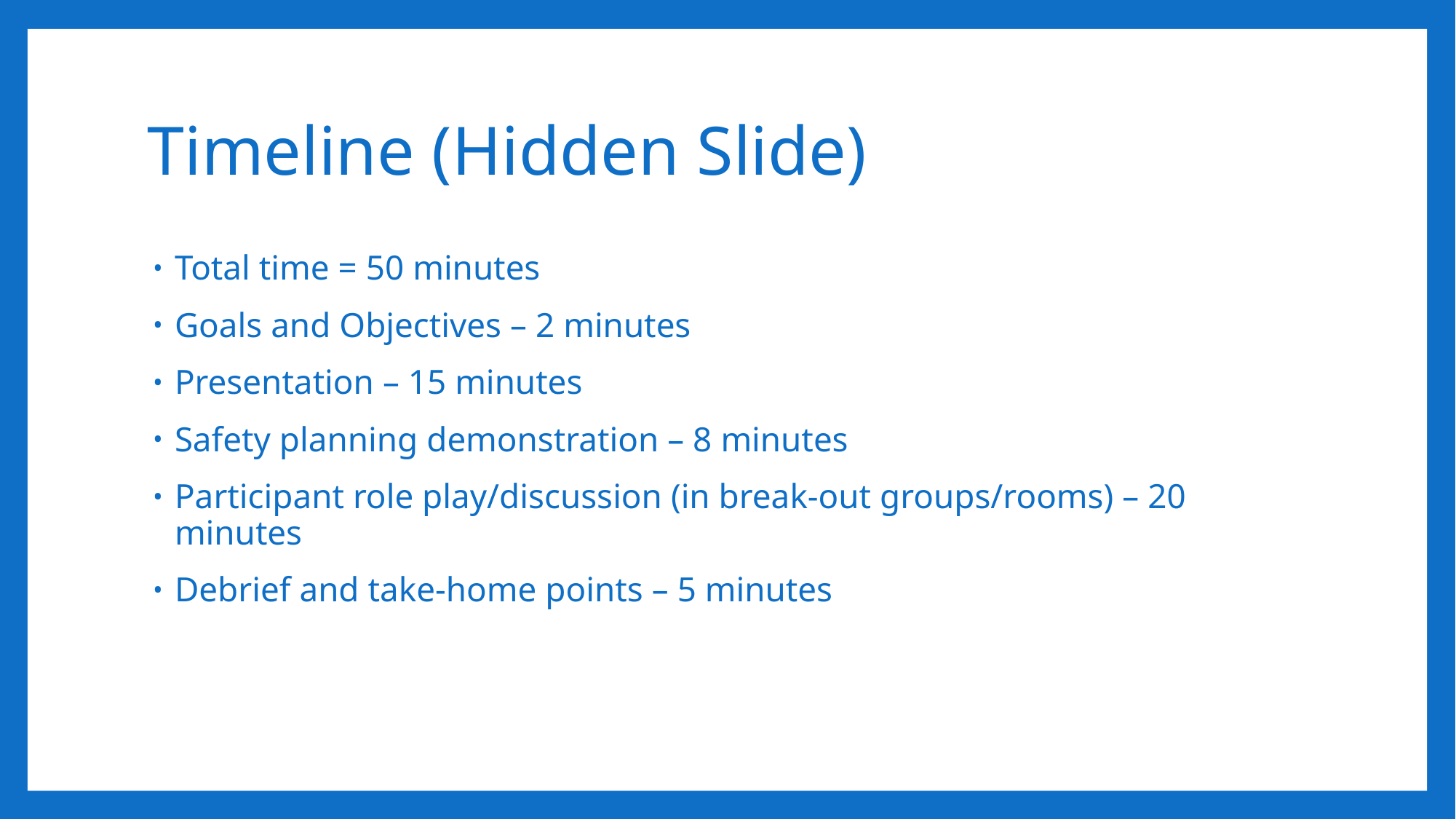

# Timeline (Hidden Slide)
Total time = 50 minutes
Goals and Objectives – 2 minutes
Presentation – 15 minutes
Safety planning demonstration – 8 minutes
Participant role play/discussion (in break-out groups/rooms) – 20 minutes
Debrief and take-home points – 5 minutes

## Slide 3
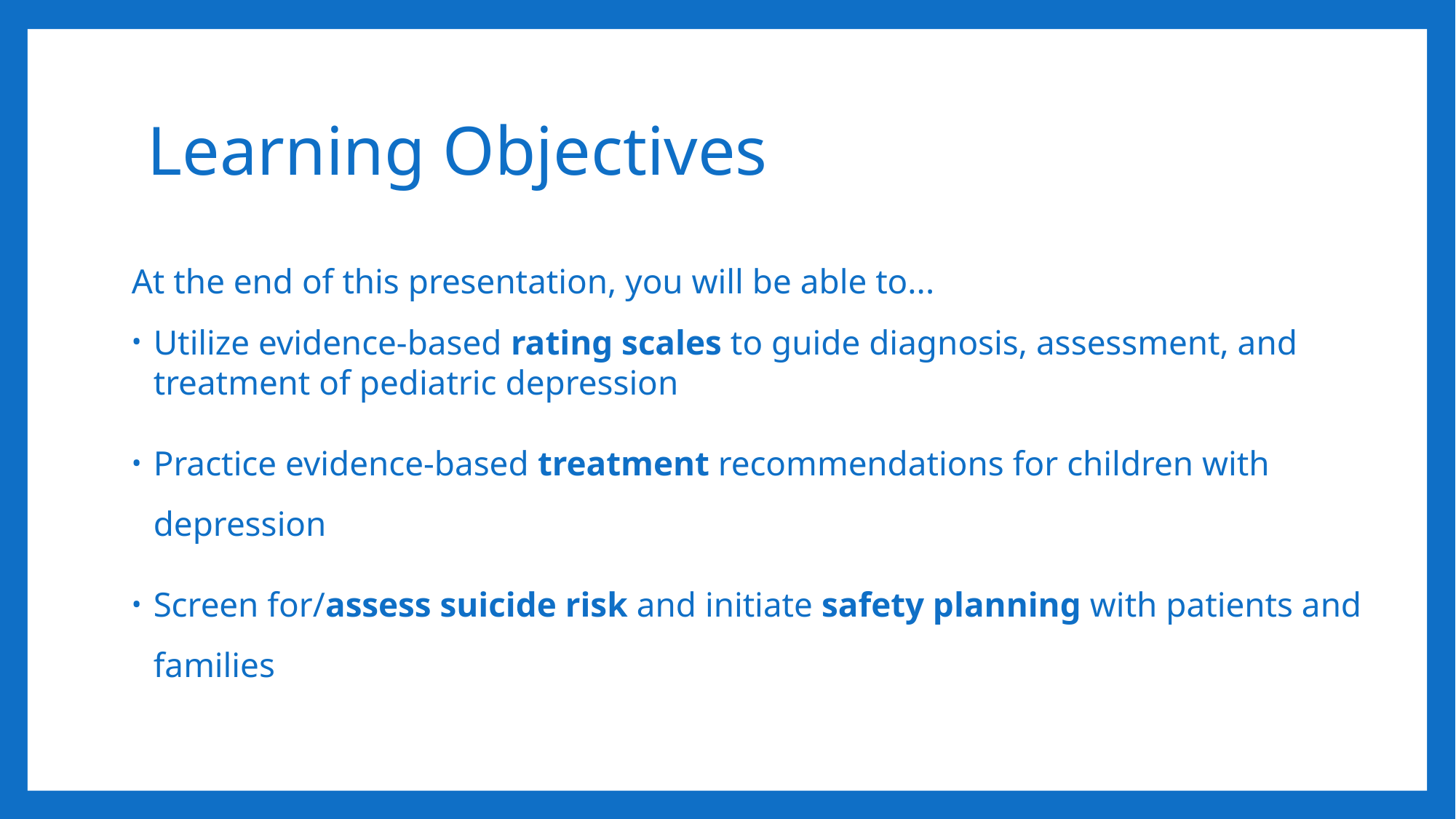

# Learning Objectives
At the end of this presentation, you will be able to...
Utilize evidence-based rating scales to guide diagnosis, assessment, and treatment of pediatric depression
Practice evidence-based treatment recommendations for children with depression
Screen for/assess suicide risk and initiate safety planning with patients and families

## Slide 4
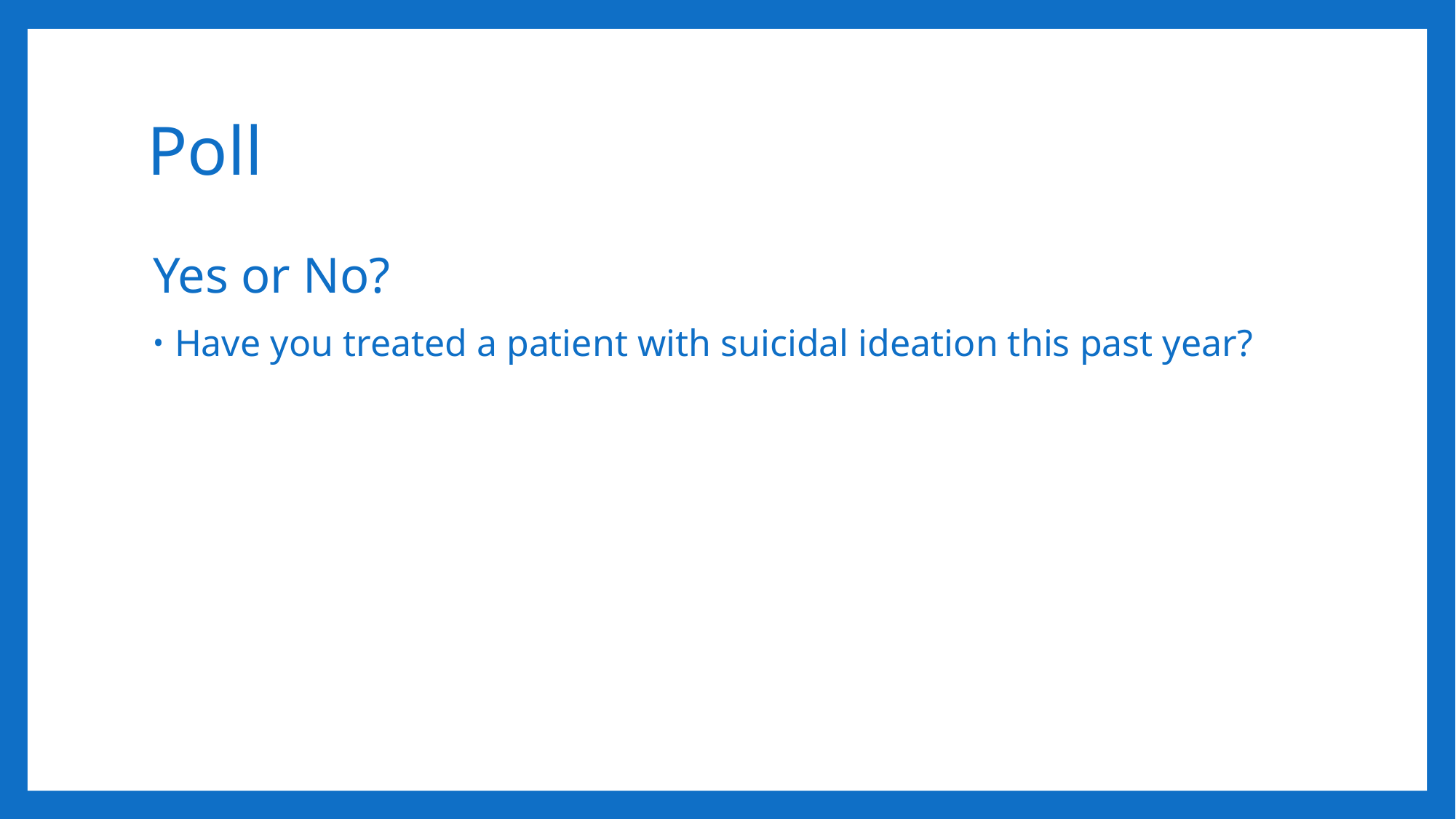

# Poll
Yes or No?
Have you treated a patient with suicidal ideation this past year?

## Slide 5
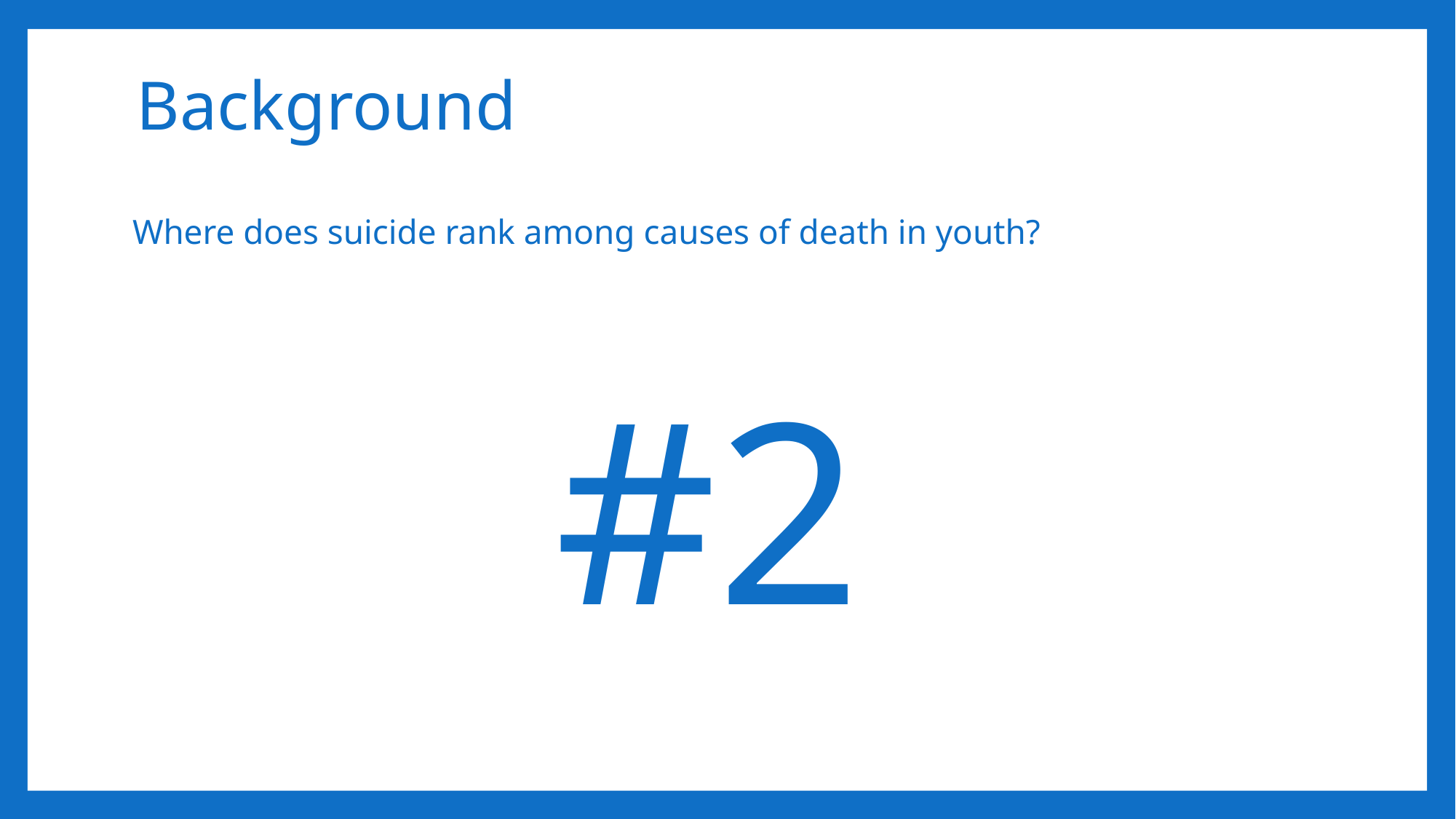

# Background
Where does suicide rank among causes of death in youth?
#2

## Slide 6
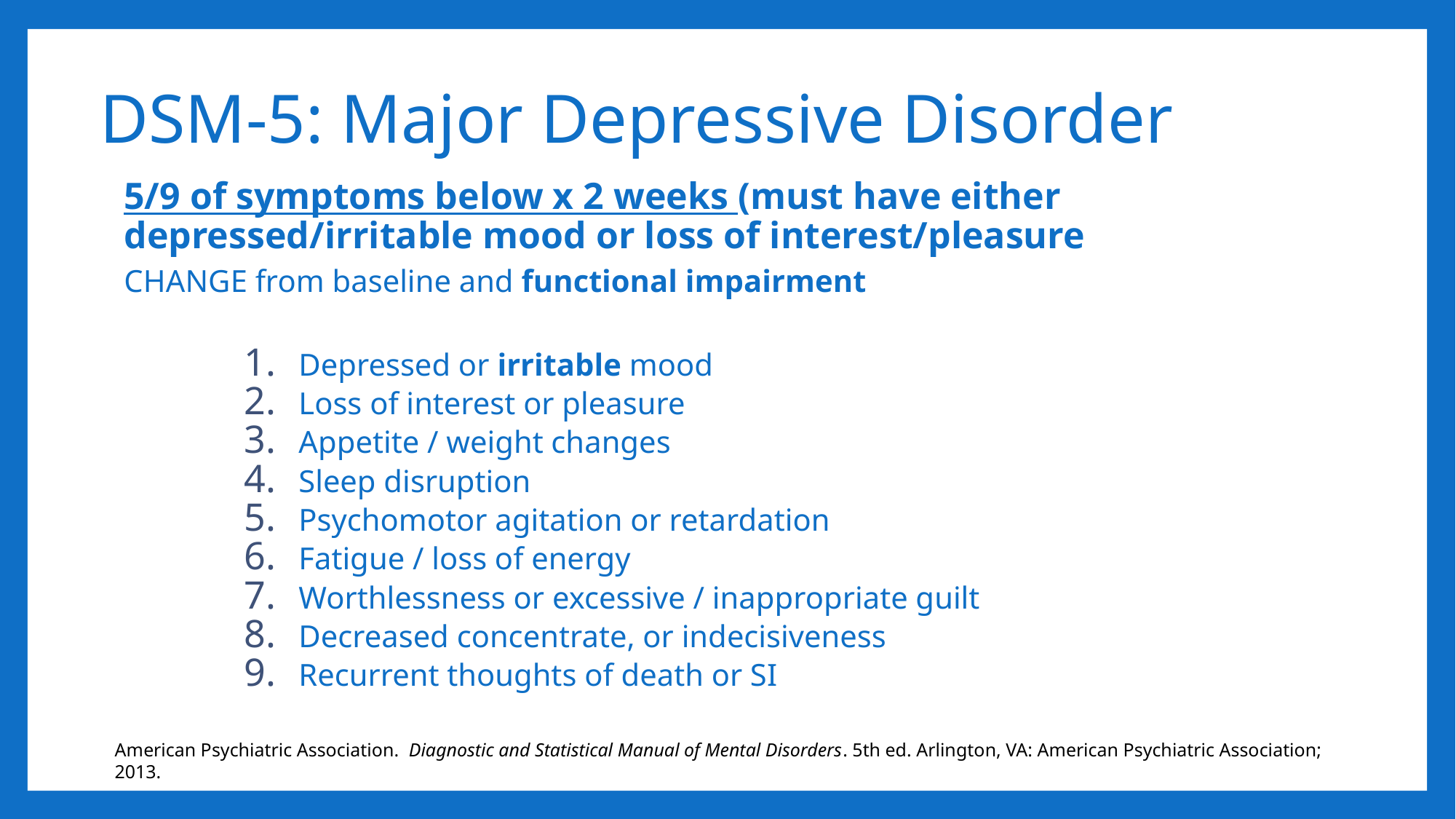

# DSM-5: Major Depressive Disorder
5/9 of symptoms below x 2 weeks (must have either depressed/irritable mood or loss of interest/pleasure
CHANGE from baseline and functional impairment
Depressed or irritable mood
Loss of interest or pleasure
Appetite / weight changes
Sleep disruption
Psychomotor agitation or retardation
Fatigue / loss of energy
Worthlessness or excessive / inappropriate guilt
Decreased concentrate, or indecisiveness
Recurrent thoughts of death or SI
American Psychiatric Association.  Diagnostic and Statistical Manual of Mental Disorders. 5th ed. Arlington, VA: American Psychiatric Association; 2013.

## Slide 7
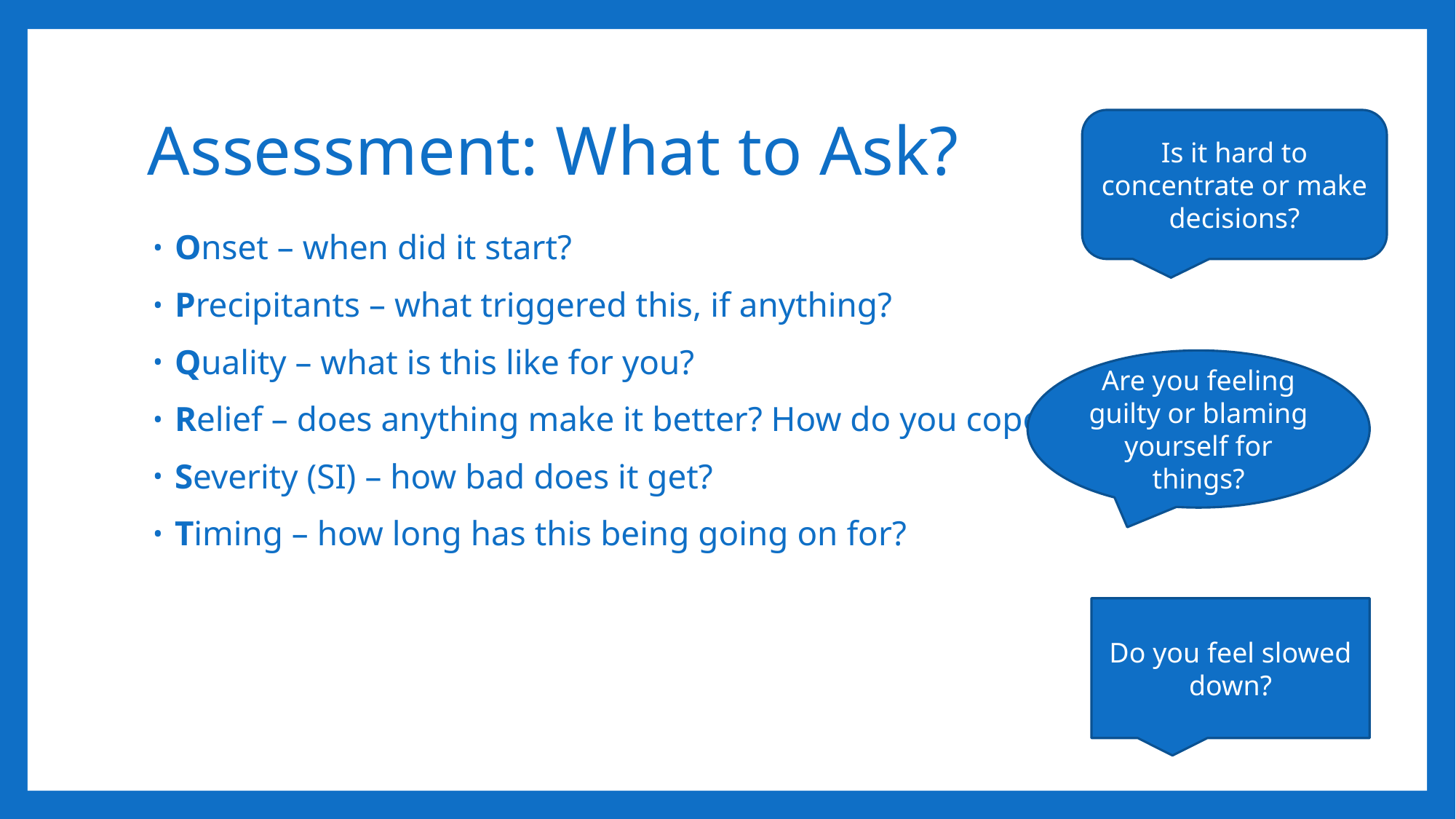

# Assessment: What to Ask?
Is it hard to concentrate or make decisions?
Onset – when did it start?
Precipitants – what triggered this, if anything?
Quality – what is this like for you?
Relief – does anything make it better? How do you cope?
Severity (SI) – how bad does it get?
Timing – how long has this being going on for?
Are you feeling guilty or blaming yourself for things?
Do you feel slowed down?

## Slide 8
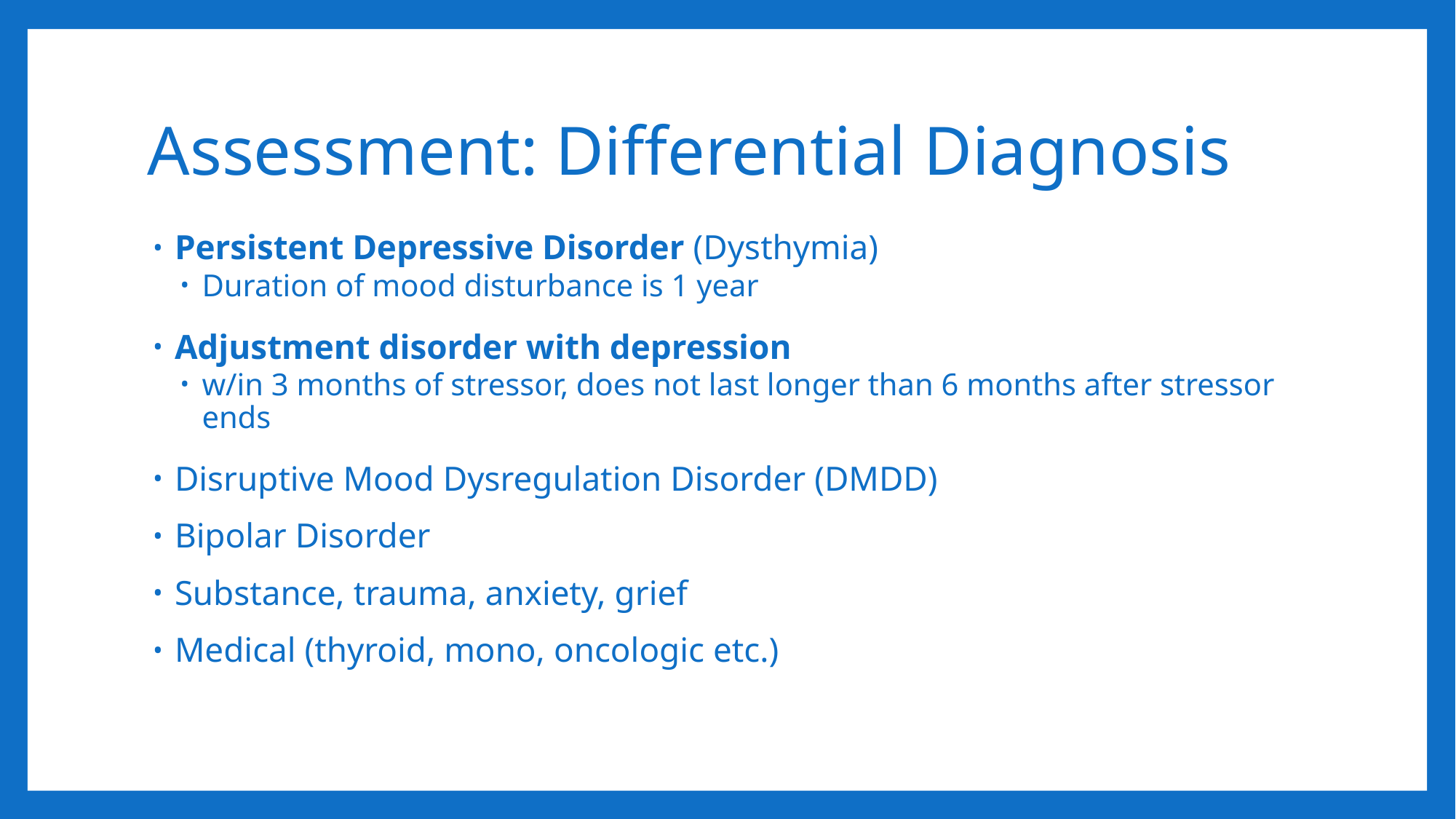

# Assessment: Differential Diagnosis
Persistent Depressive Disorder (Dysthymia)
Duration of mood disturbance is 1 year
Adjustment disorder with depression
w/in 3 months of stressor, does not last longer than 6 months after stressor ends
Disruptive Mood Dysregulation Disorder (DMDD)
Bipolar Disorder
Substance, trauma, anxiety, grief
Medical (thyroid, mono, oncologic etc.)

## Slide 9
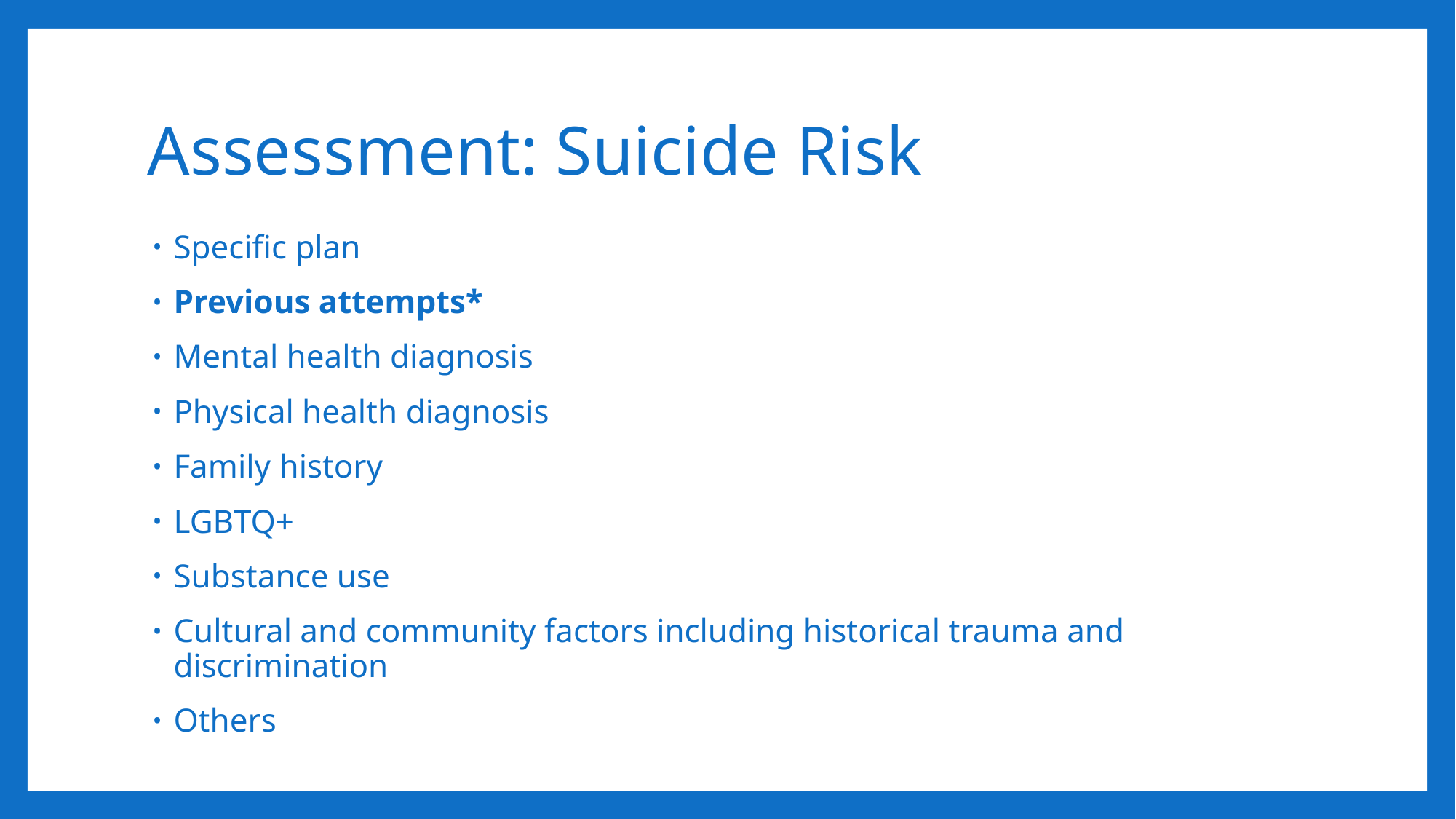

# Assessment: Suicide Risk
Specific plan
Previous attempts*
Mental health diagnosis
Physical health diagnosis
Family history
LGBTQ+
Substance use
Cultural and community factors including historical trauma and discrimination
Others

## Slide 10
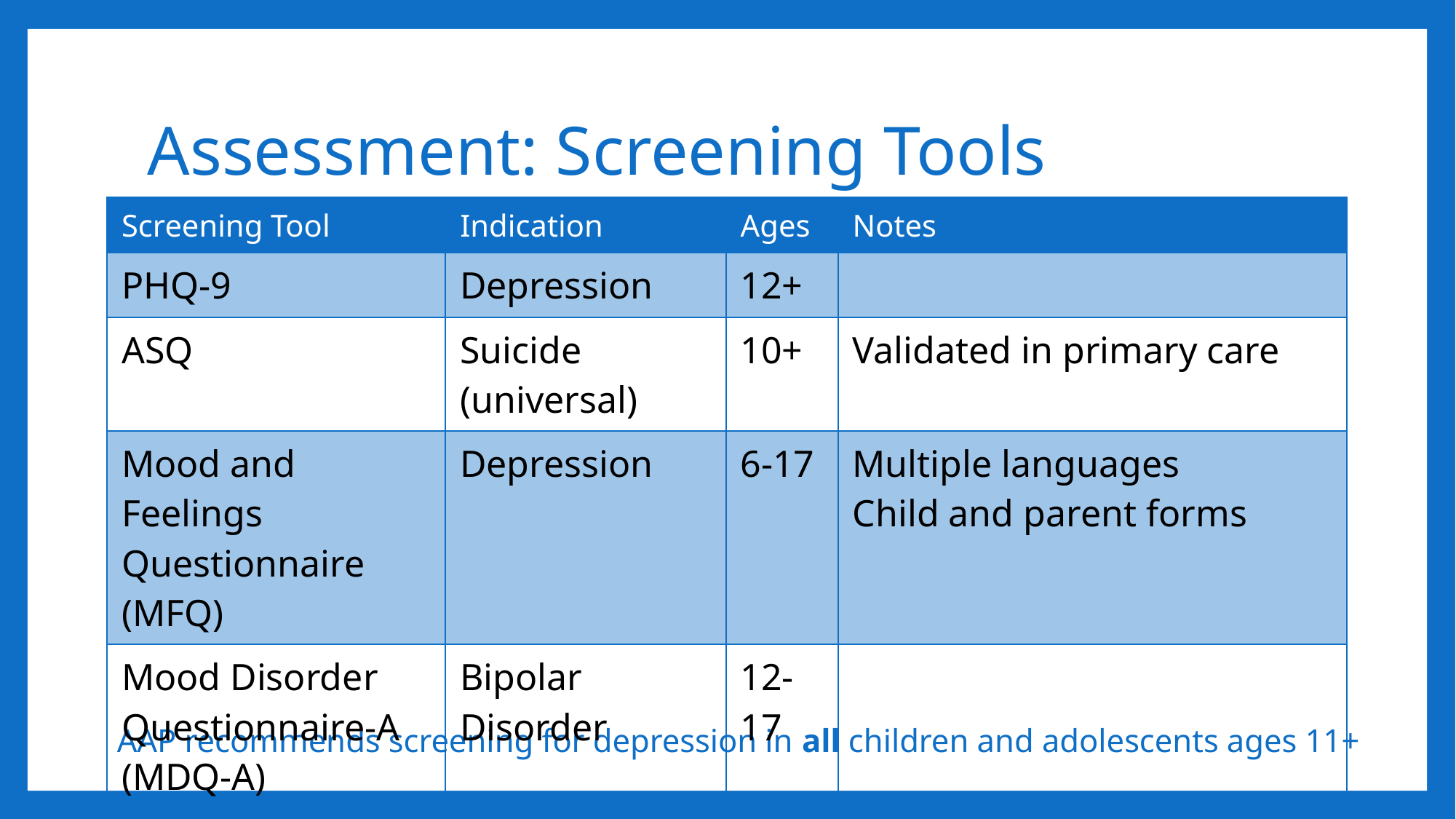

# Assessment: Screening Tools
| Screening Tool | Indication | Ages | Notes |
| --- | --- | --- | --- |
| PHQ-9 | Depression | 12+ | |
| ASQ | Suicide (universal) | 10+ | Validated in primary care |
| Mood and Feelings Questionnaire (MFQ) | Depression | 6-17 | Multiple languages Child and parent forms |
| Mood Disorder Questionnaire-A (MDQ-A) | Bipolar Disorder | 12-17 | |
AAP recommends screening for depression in all children and adolescents ages 11+

## Slide 11
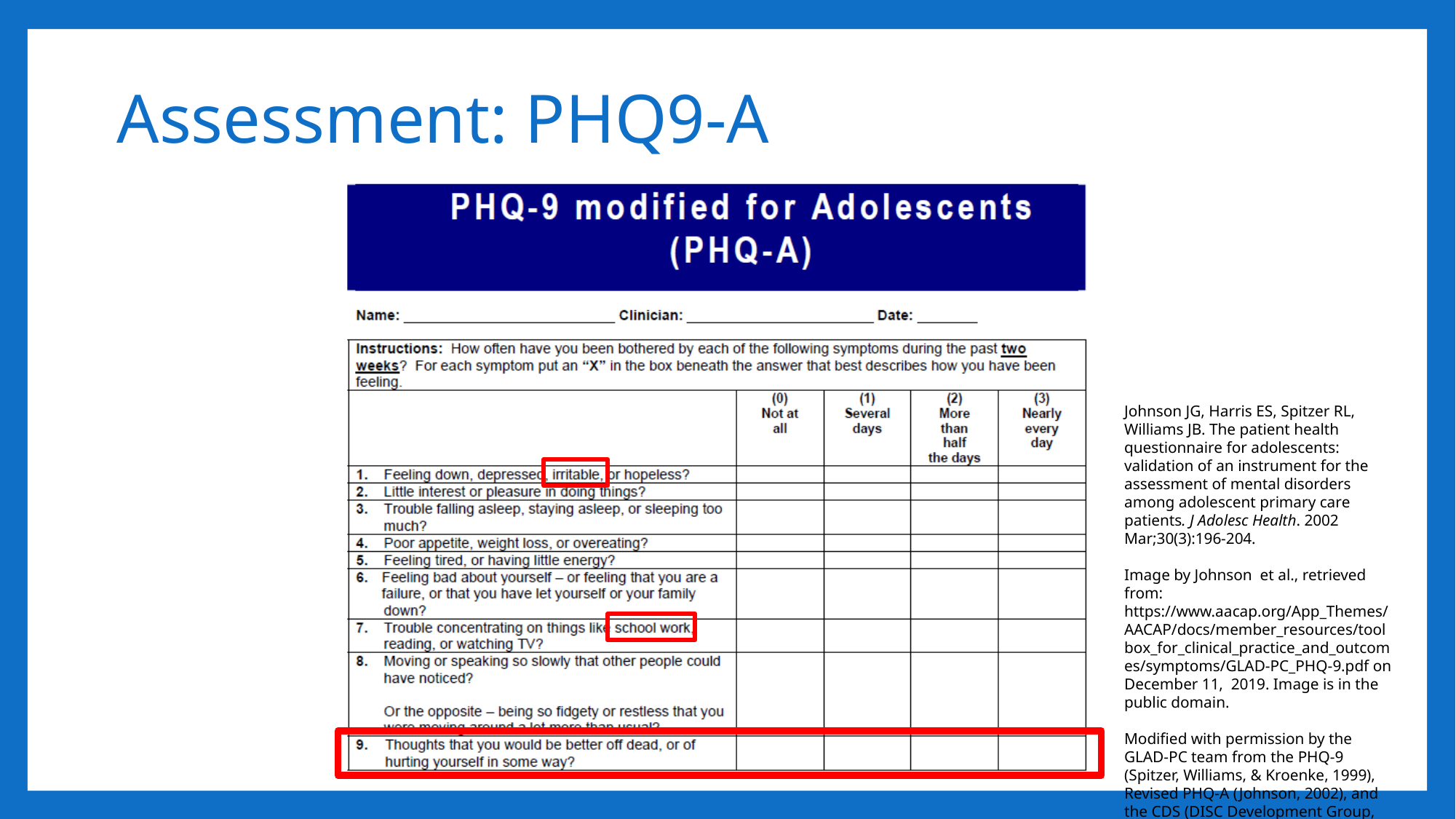

# Assessment: PHQ9-A
Johnson JG, Harris ES, Spitzer RL, Williams JB. The patient health questionnaire for adolescents: validation of an instrument for the assessment of mental disorders among adolescent primary care patients. J Adolesc Health. 2002 Mar;30(3):196-204.
Image by Johnson et al., retrieved from: https://www.aacap.org/App_Themes/AACAP/docs/member_resources/toolbox_for_clinical_practice_and_outcomes/symptoms/GLAD-PC_PHQ-9.pdf on December 11, 2019. Image is in the public domain.
Modified with permission by the GLAD-PC team from the PHQ-9 (Spitzer, Williams, & Kroenke, 1999), Revised PHQ-A (Johnson, 2002), and the CDS (DISC Development Group, 2000)

## Slide 12
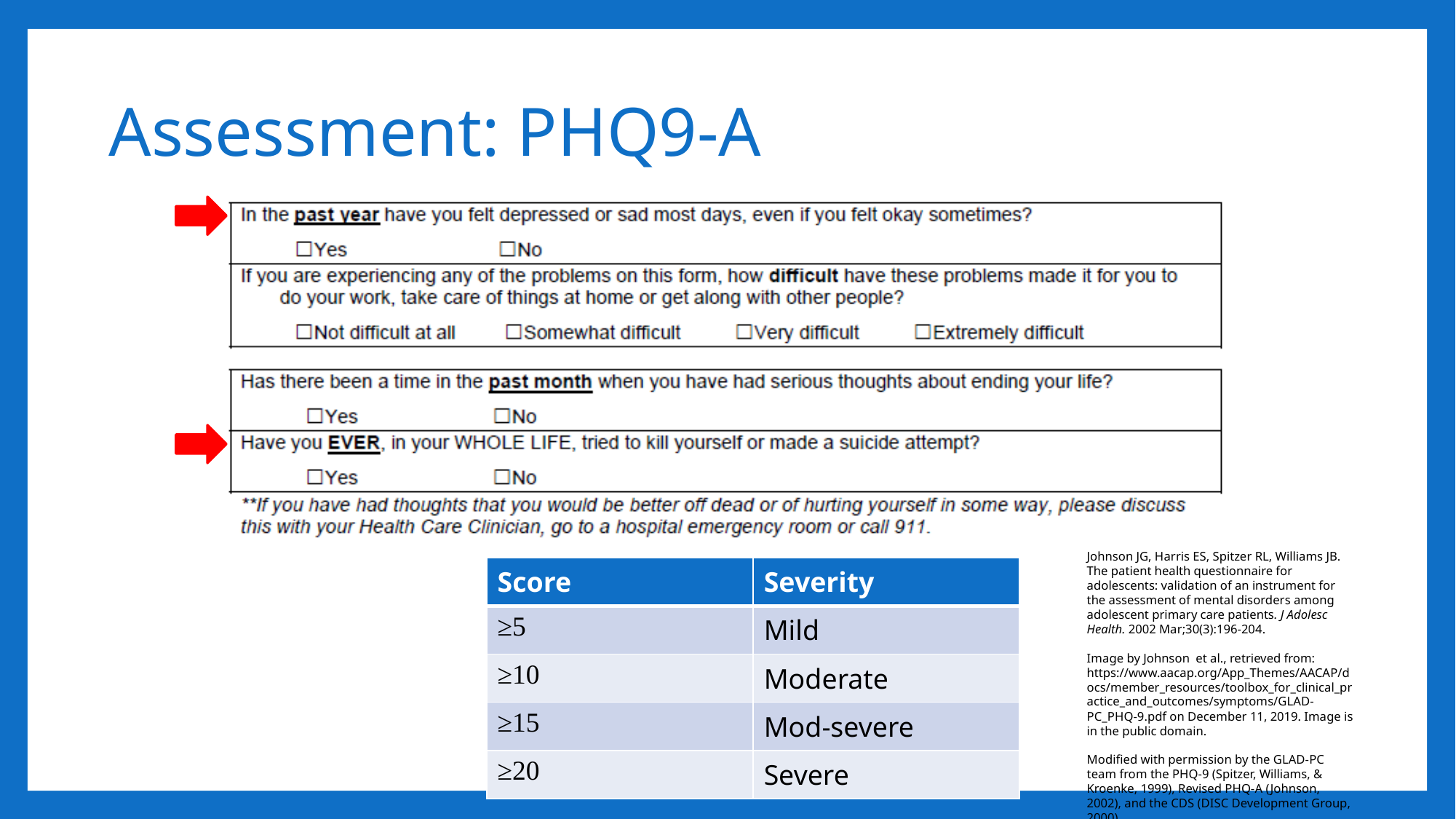

# Assessment: PHQ9-A
Johnson JG, Harris ES, Spitzer RL, Williams JB. The patient health questionnaire for adolescents: validation of an instrument for the assessment of mental disorders among adolescent primary care patients. J Adolesc Health. 2002 Mar;30(3):196-204.
Image by Johnson et al., retrieved from: https://www.aacap.org/App_Themes/AACAP/docs/member_resources/toolbox_for_clinical_practice_and_outcomes/symptoms/GLAD-PC_PHQ-9.pdf on December 11, 2019. Image is in the public domain.
Modified with permission by the GLAD-PC team from the PHQ-9 (Spitzer, Williams, & Kroenke, 1999), Revised PHQ-A (Johnson, 2002), and the CDS (DISC Development Group, 2000)
.
| Score | Severity |
| --- | --- |
| ≥5 | Mild |
| ≥10 | Moderate |
| ≥15 | Mod-severe |
| ≥20 | Severe |

## Slide 13
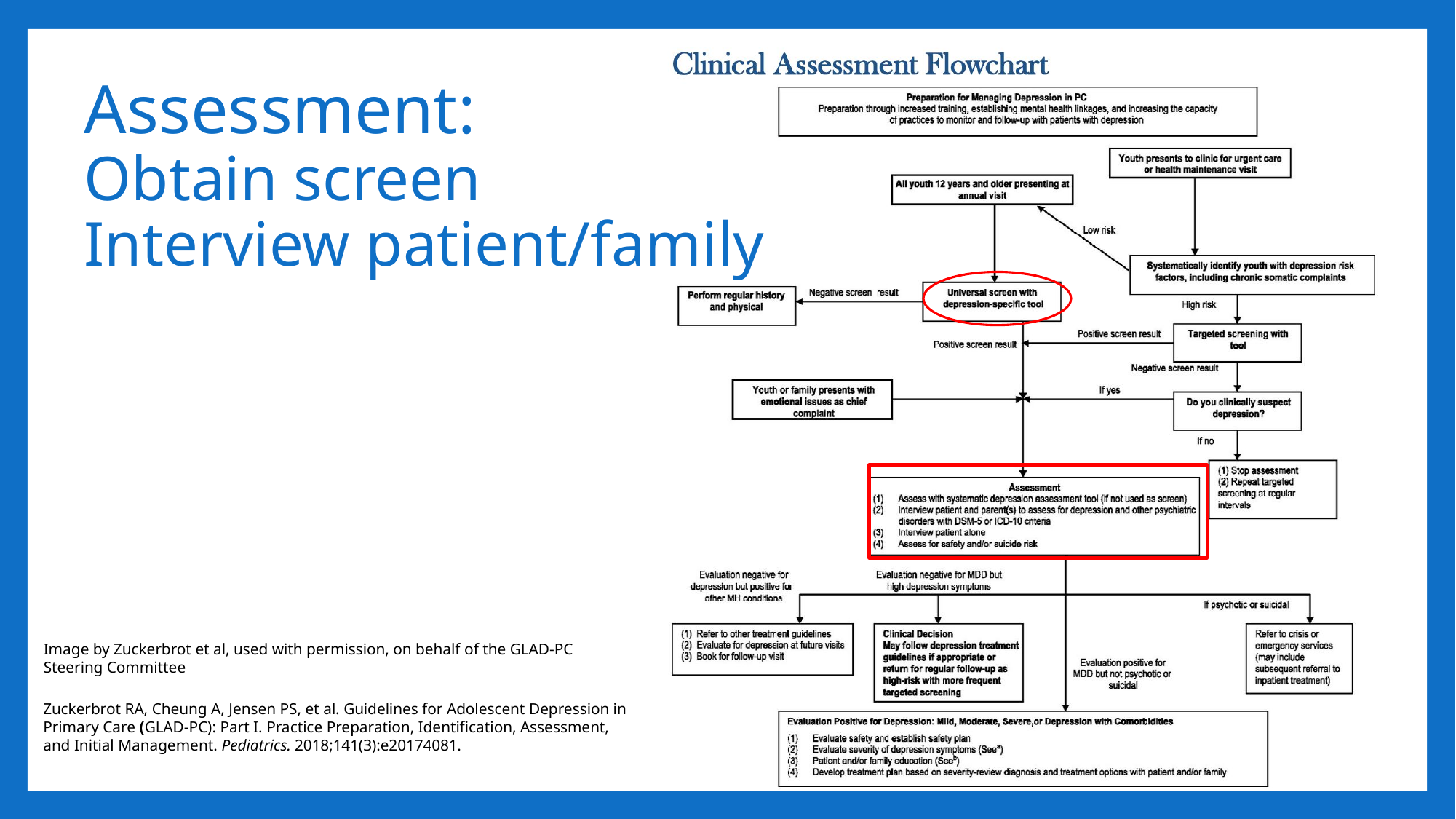

# Assessment: Obtain screen Interview patient/family
Image by Zuckerbrot et al, used with permission, on behalf of the GLAD-PC Steering Committee
Zuckerbrot RA, Cheung A, Jensen PS, et al. Guidelines for Adolescent Depression in Primary Care (GLAD-PC): Part I. Practice Preparation, Identification, Assessment, and Initial Management. Pediatrics. 2018;141(3):e20174081.

## Slide 14
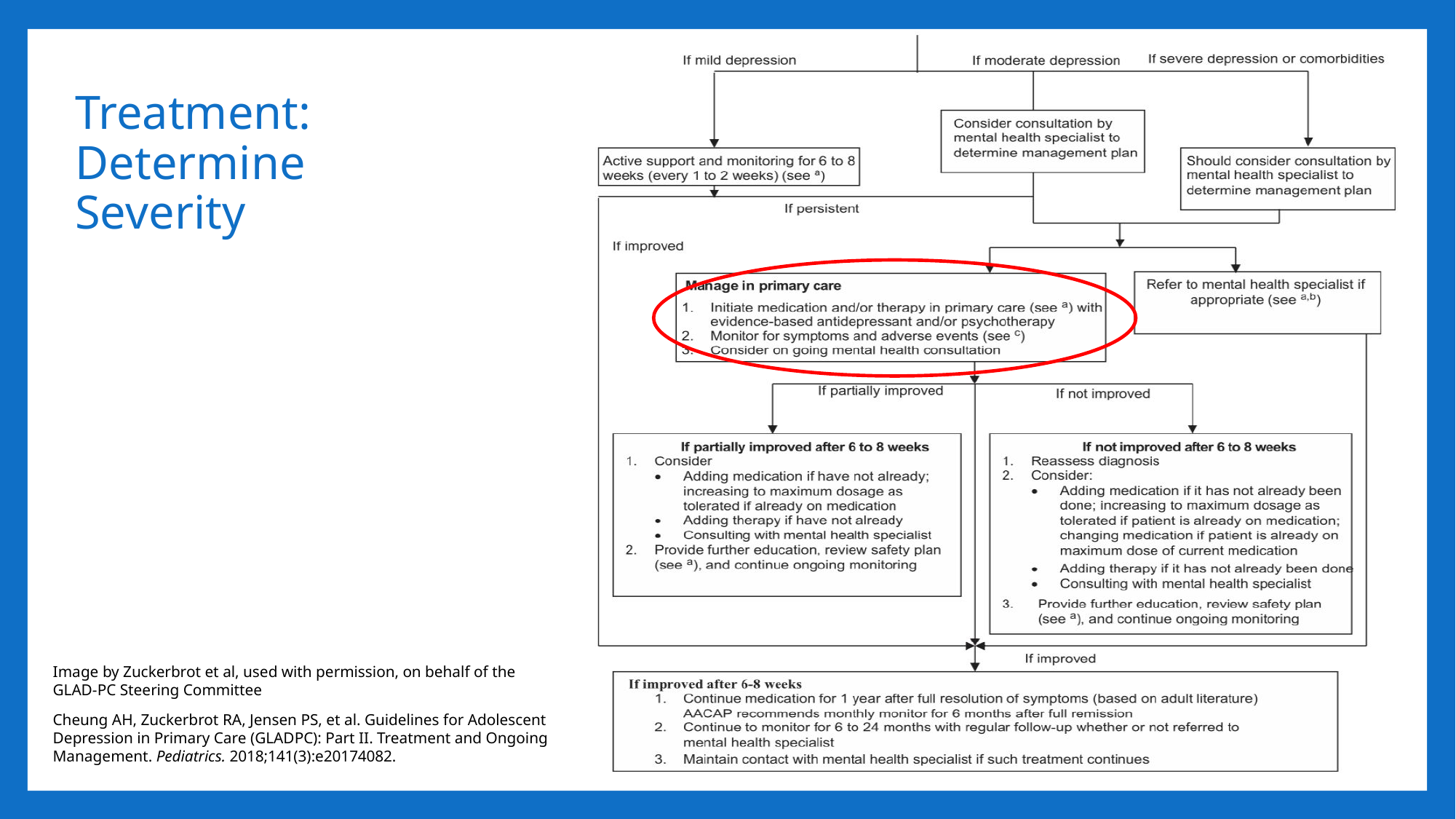

# Treatment: Determine Severity
Image by Zuckerbrot et al, used with permission, on behalf of the GLAD-PC Steering Committee
Cheung AH, Zuckerbrot RA, Jensen PS, et al. Guidelines for Adolescent Depression in Primary Care (GLADPC): Part II. Treatment and Ongoing Management. Pediatrics. 2018;141(3):e20174082.

## Slide 15
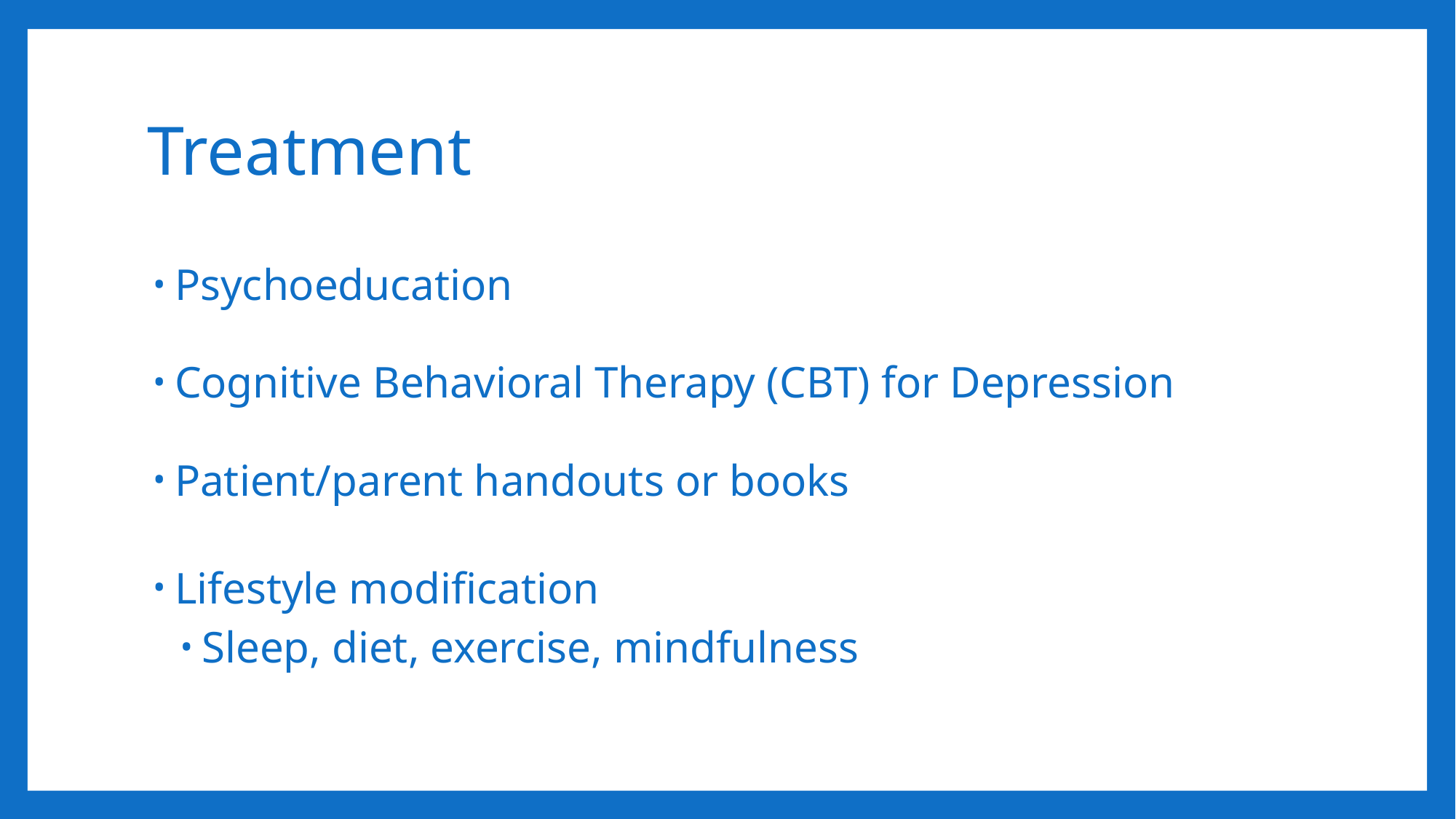

# Treatment
Psychoeducation
Cognitive Behavioral Therapy (CBT) for Depression
Patient/parent handouts or books
Lifestyle modification
Sleep, diet, exercise, mindfulness

## Slide 16
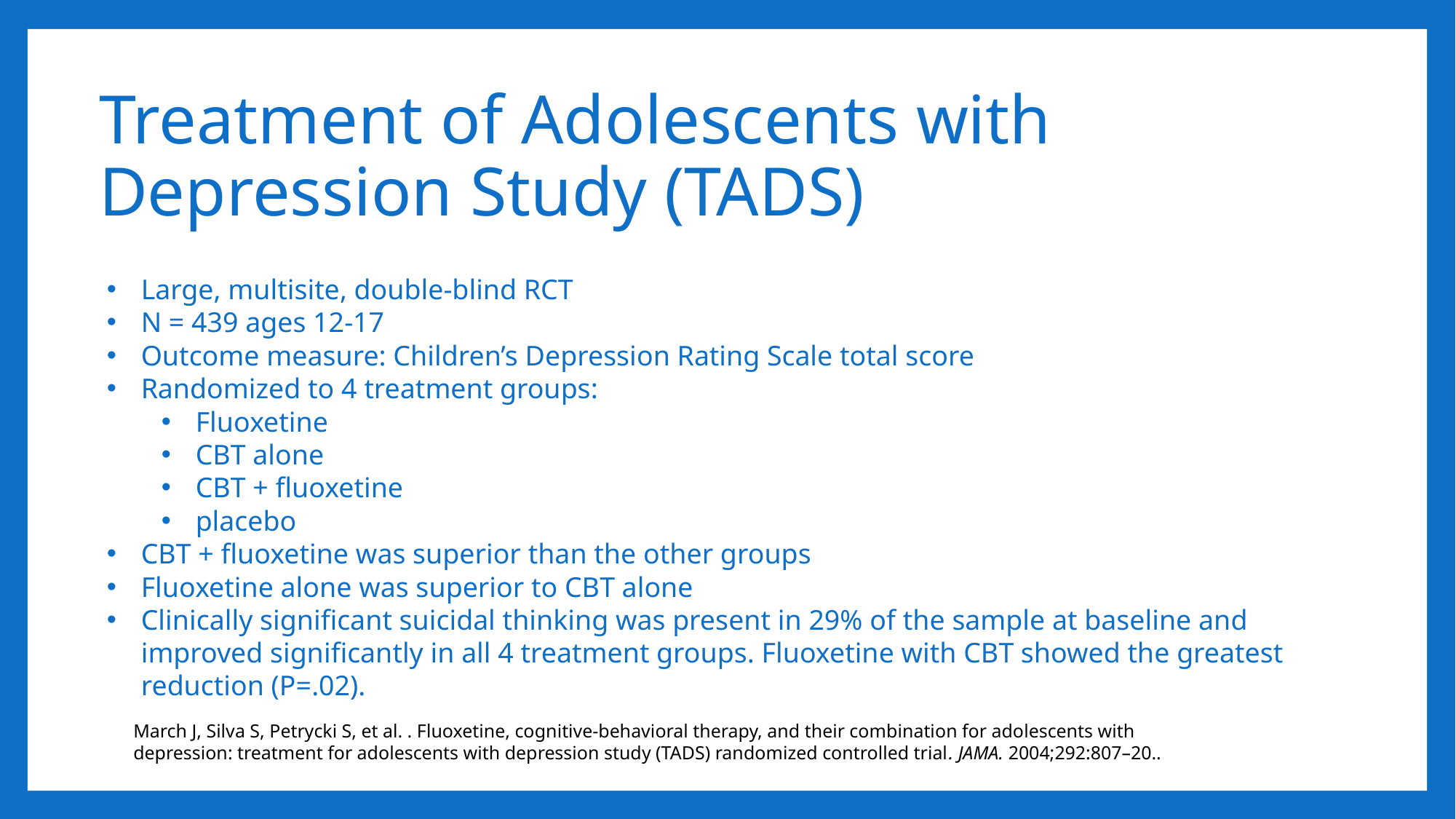

# Treatment of Adolescents with Depression Study (TADS)
Large, multisite, double-blind RCT
N = 439 ages 12-17
Outcome measure: Children’s Depression Rating Scale total score
Randomized to 4 treatment groups:
Fluoxetine
CBT alone
CBT + fluoxetine
placebo
CBT + fluoxetine was superior than the other groups
Fluoxetine alone was superior to CBT alone
Clinically significant suicidal thinking was present in 29% of the sample at baseline and improved significantly in all 4 treatment groups. Fluoxetine with CBT showed the greatest reduction (P=.02).
March J, Silva S, Petrycki S, et al. . Fluoxetine, cognitive-behavioral therapy, and their combination for adolescents with depression: treatment for adolescents with depression study (TADS) randomized controlled trial. JAMA. 2004;292:807–20..

## Slide 17
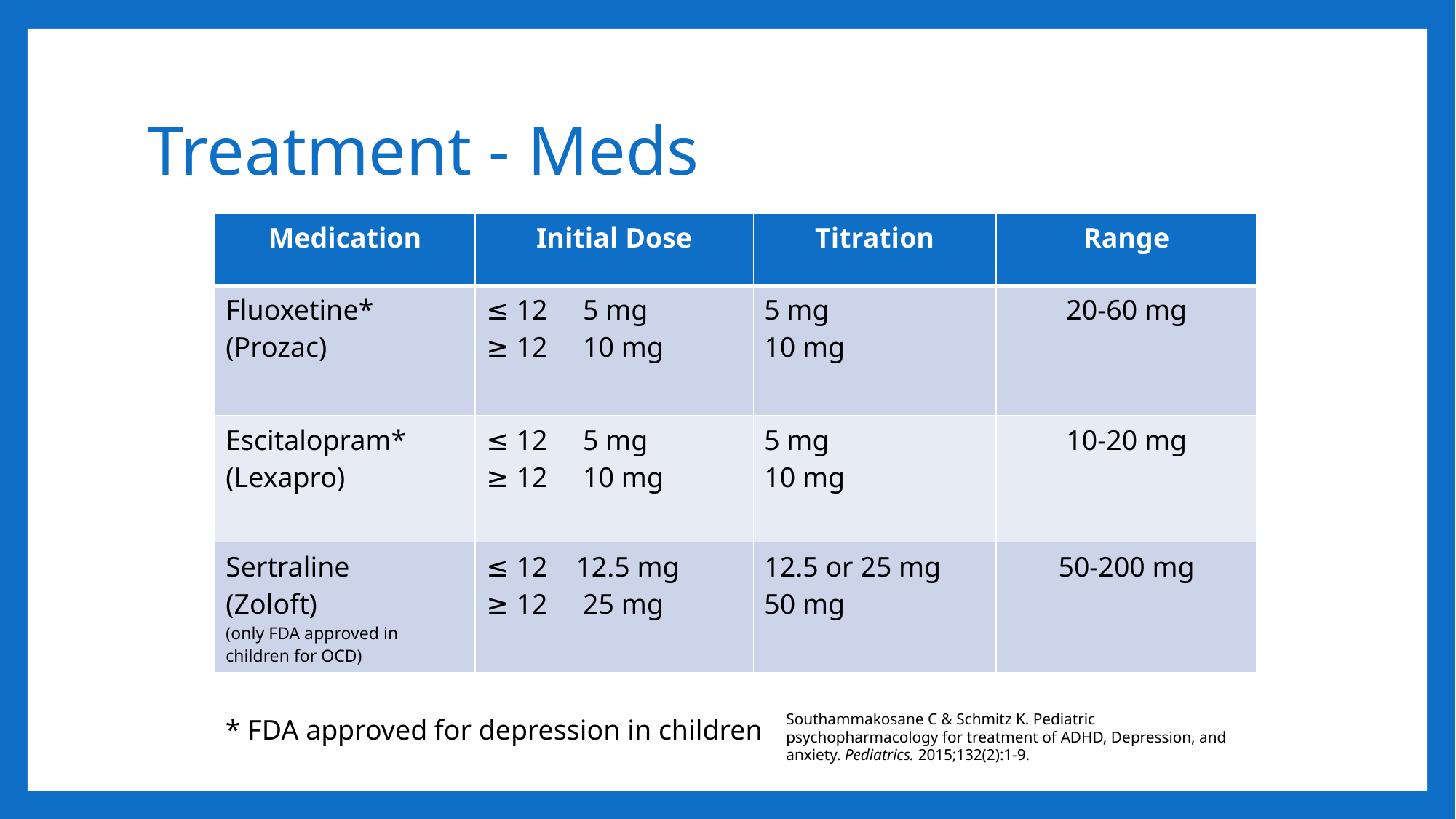

# Treatment - Meds
| Medication | Initial Dose | Titration | Range |
| --- | --- | --- | --- |
| Fluoxetine\* (Prozac) | ≤ 12     5 mg ≥ 12     10 mg | 5 mg 10 mg | 20-60 mg |
| Escitalopram\* (Lexapro) | ≤ 12 5 mg ≥ 12 10 mg | 5 mg 10 mg | 10-20 mg |
| Sertraline (Zoloft) (only FDA approved in children for OCD) | ≤ 12 12.5 mg ≥ 12 25 mg | 12.5 or 25 mg 50 mg | 50-200 mg |
Southammakosane C & Schmitz K. Pediatric psychopharmacology for treatment of ADHD, Depression, and anxiety. Pediatrics. 2015;132(2):1-9.
* FDA approved for depression in children

## Slide 18
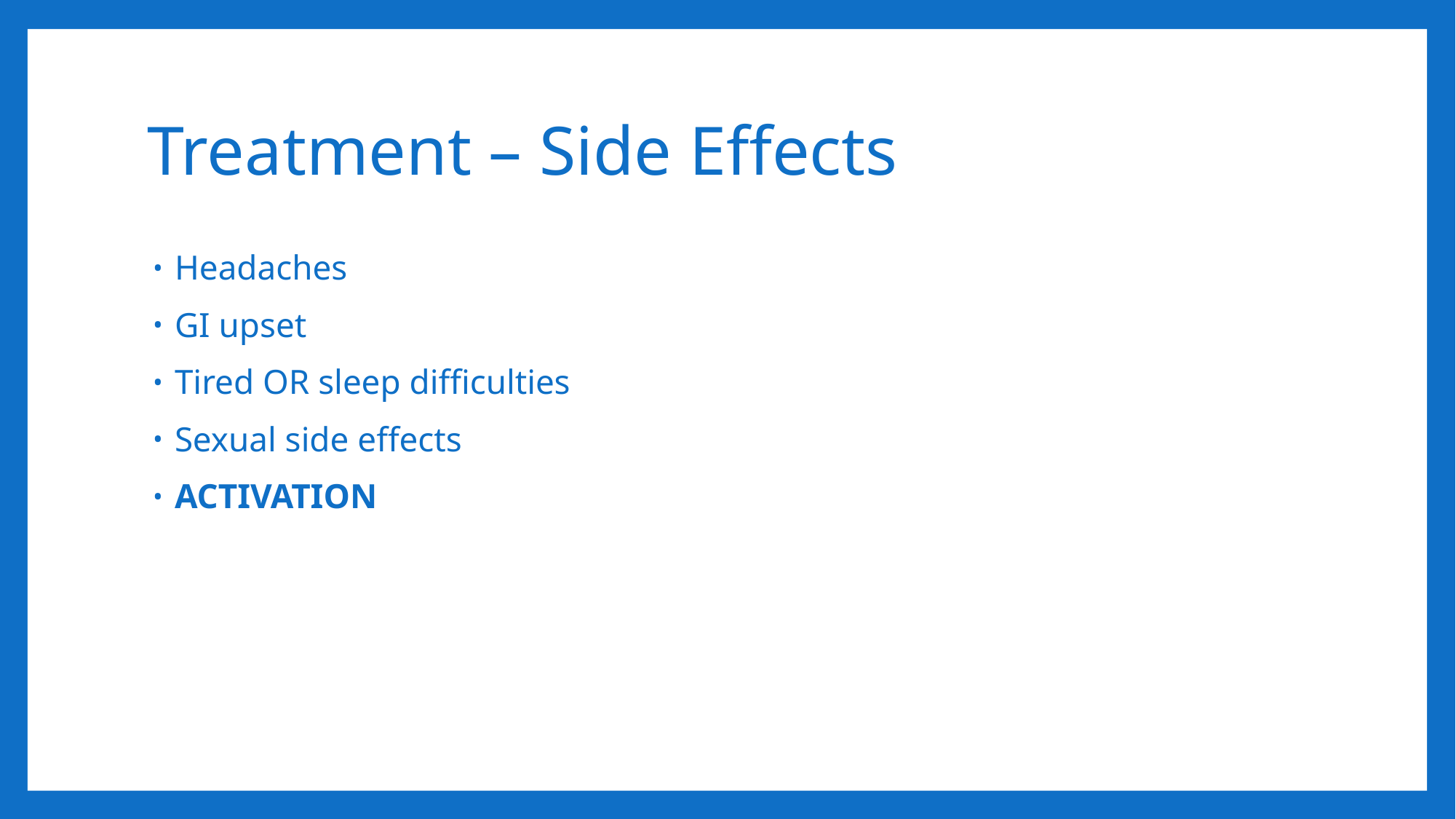

# Treatment – Side Effects
Headaches
GI upset
Tired OR sleep difficulties
Sexual side effects
ACTIVATION

## Slide 19
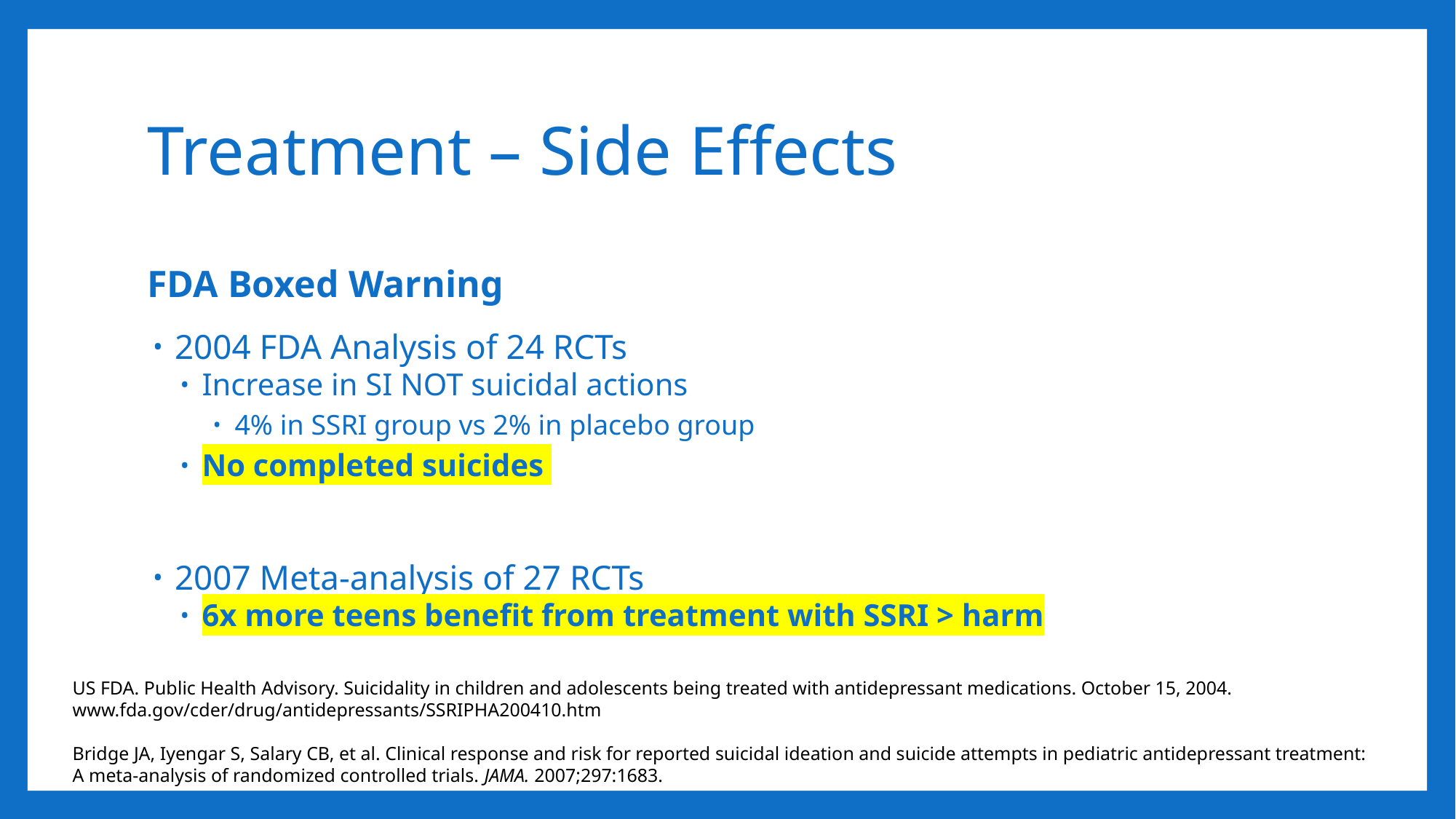

# Treatment – Side Effects
FDA Boxed Warning
2004 FDA Analysis of 24 RCTs
Increase in SI NOT suicidal actions
4% in SSRI group vs 2% in placebo group
No completed suicides
2007 Meta-analysis of 27 RCTs
6x more teens benefit from treatment with SSRI > harm
US FDA. Public Health Advisory. Suicidality in children and adolescents being treated with antidepressant medications. October 15, 2004. www.fda.gov/cder/drug/antidepressants/SSRIPHA200410.htm
Bridge JA, Iyengar S, Salary CB, et al. Clinical response and risk for reported suicidal ideation and suicide attempts in pediatric antidepressant treatment: A meta-analysis of randomized controlled trials. JAMA. 2007;297:1683.

## Slide 20
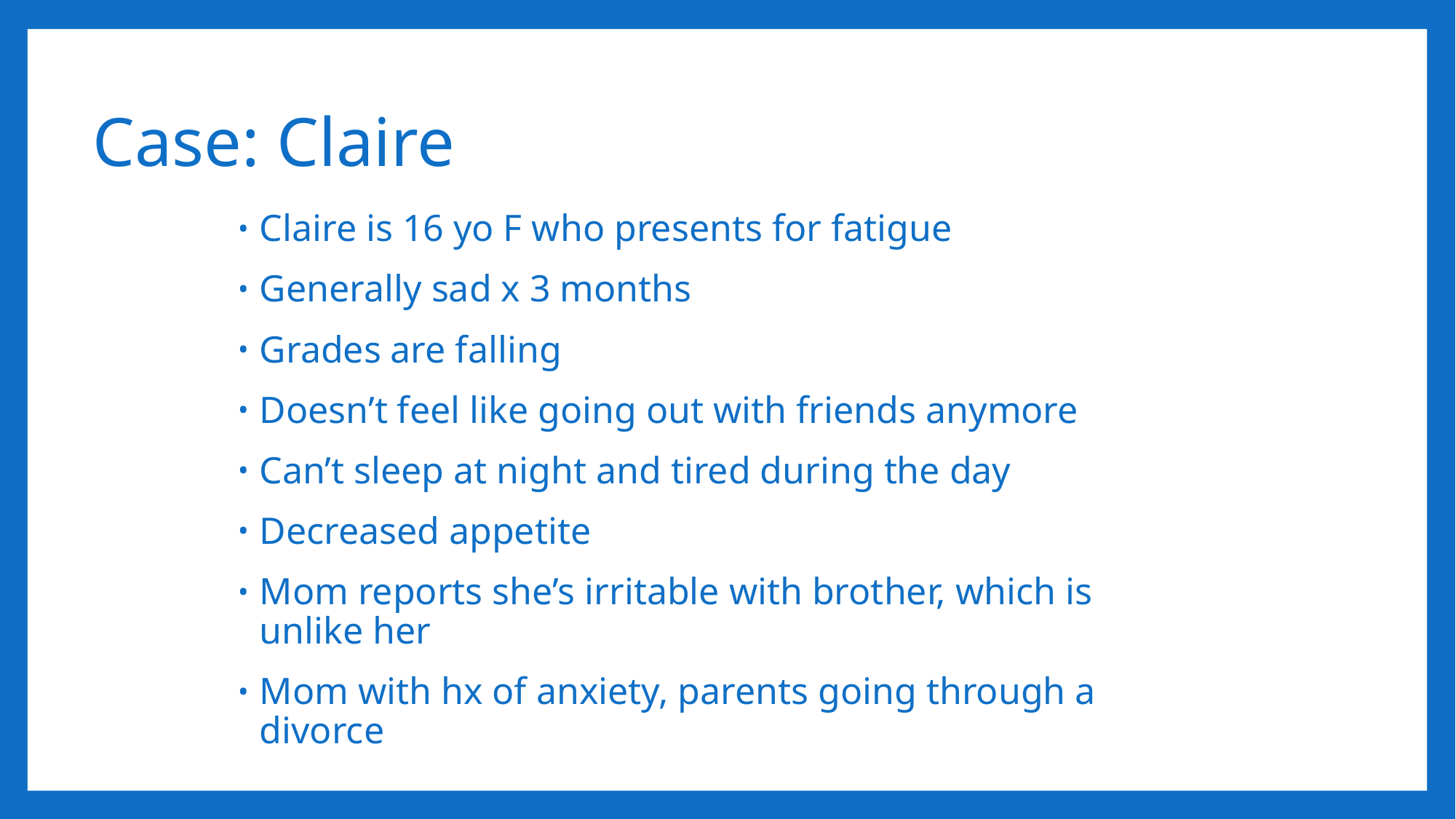

# Case: Claire
Claire is 16 yo F who presents for fatigue
Generally sad x 3 months
Grades are falling
Doesn’t feel like going out with friends anymore
Can’t sleep at night and tired during the day
Decreased appetite
Mom reports she’s irritable with brother, which is unlike her
Mom with hx of anxiety, parents going through a divorce

## Slide 21
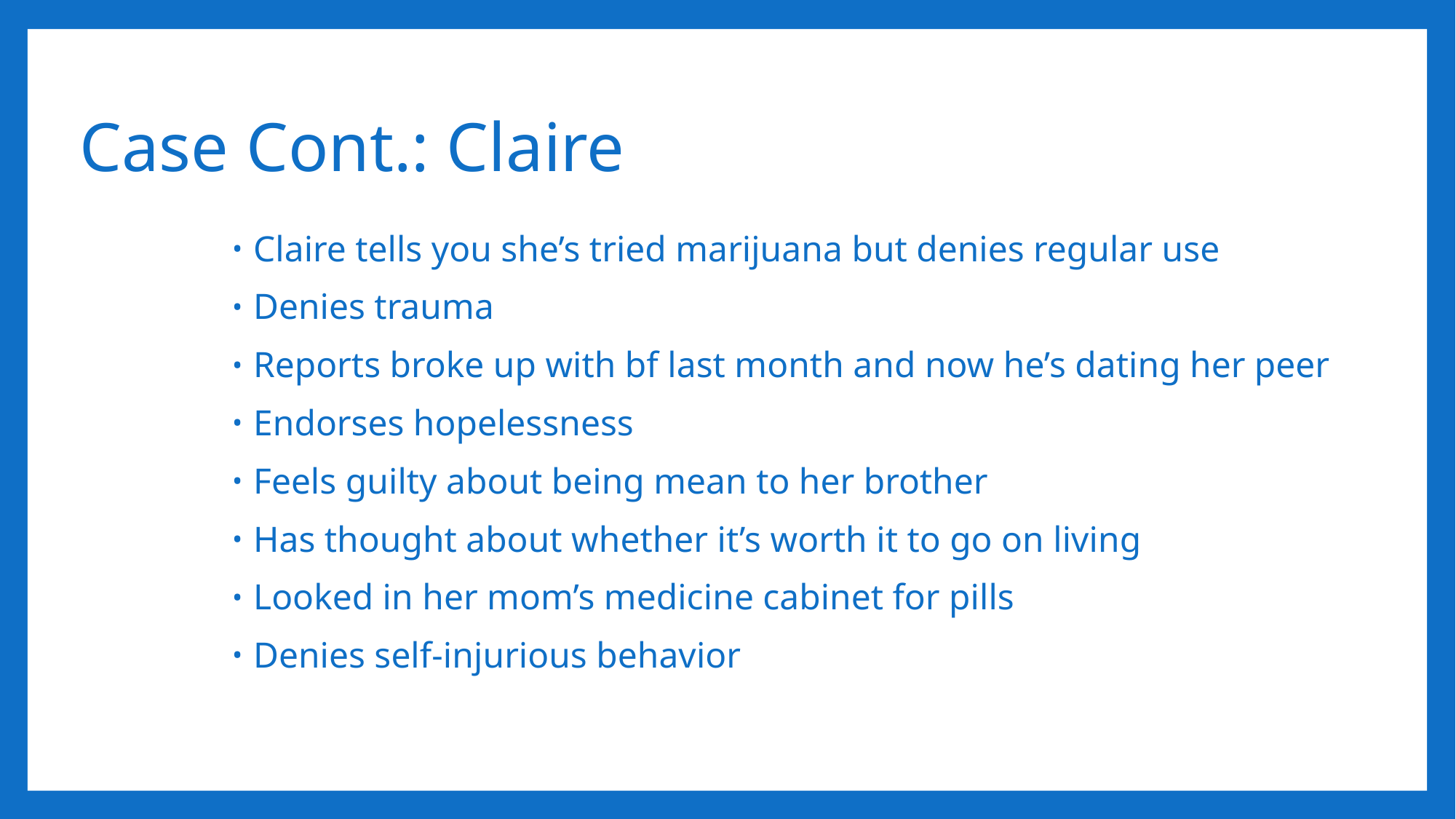

# Case Cont.: Claire
Claire tells you she’s tried marijuana but denies regular use
Denies trauma
Reports broke up with bf last month and now he’s dating her peer
Endorses hopelessness
Feels guilty about being mean to her brother
Has thought about whether it’s worth it to go on living
Looked in her mom’s medicine cabinet for pills
Denies self-injurious behavior

## Slide 22
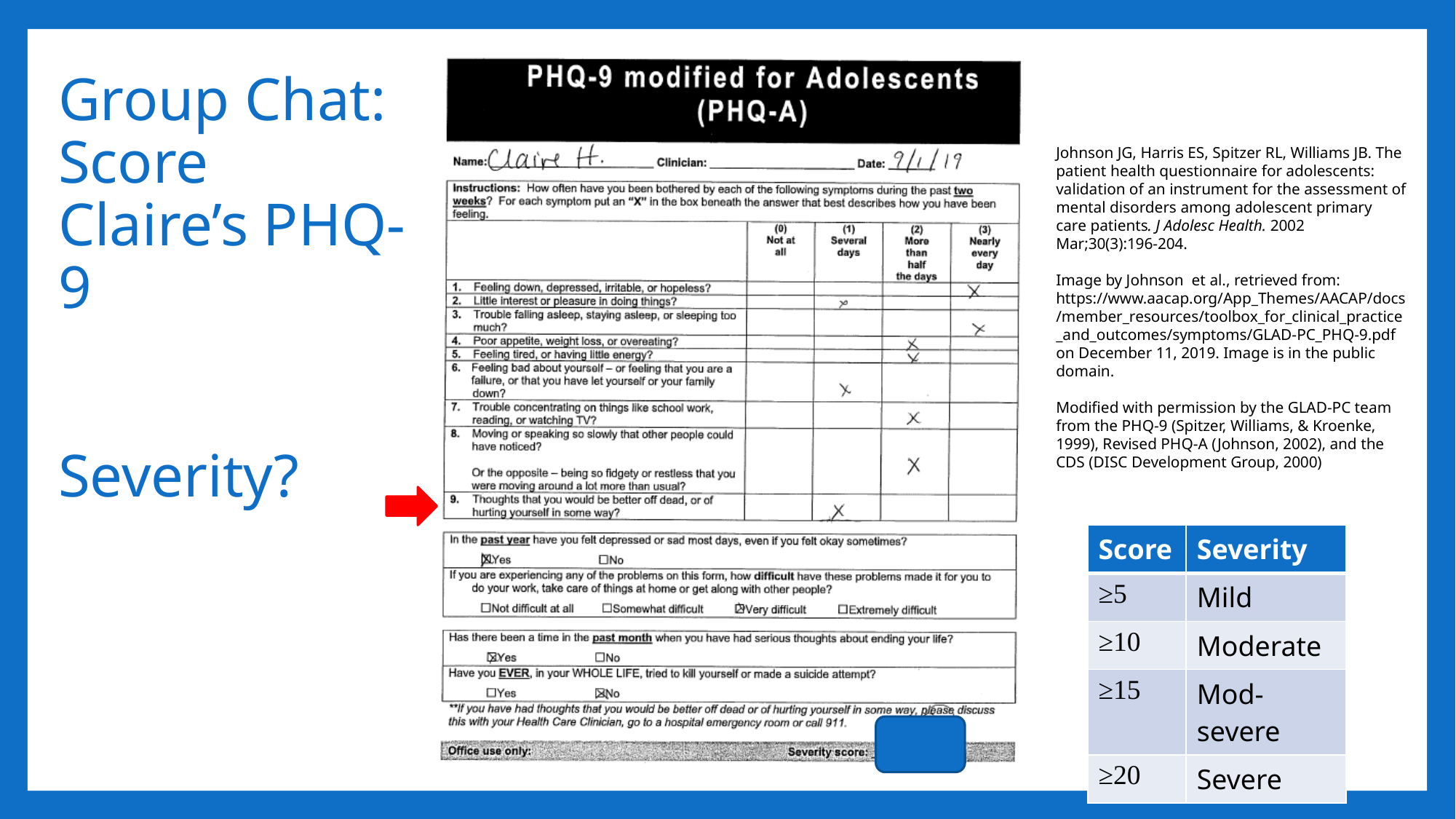

# Group Chat: Score Claire’s PHQ-9 Severity?
Johnson JG, Harris ES, Spitzer RL, Williams JB. The patient health questionnaire for adolescents: validation of an instrument for the assessment of mental disorders among adolescent primary care patients. J Adolesc Health. 2002 Mar;30(3):196-204.
Image by Johnson et al., retrieved from: https://www.aacap.org/App_Themes/AACAP/docs/member_resources/toolbox_for_clinical_practice_and_outcomes/symptoms/GLAD-PC_PHQ-9.pdf on December 11, 2019. Image is in the public domain.
Modified with permission by the GLAD-PC team from the PHQ-9 (Spitzer, Williams, & Kroenke, 1999), Revised PHQ-A (Johnson, 2002), and the CDS (DISC Development Group, 2000)
| Score | Severity |
| --- | --- |
| ≥5 | Mild |
| ≥10 | Moderate |
| ≥15 | Mod-severe |
| ≥20 | Severe |

## Slide 23
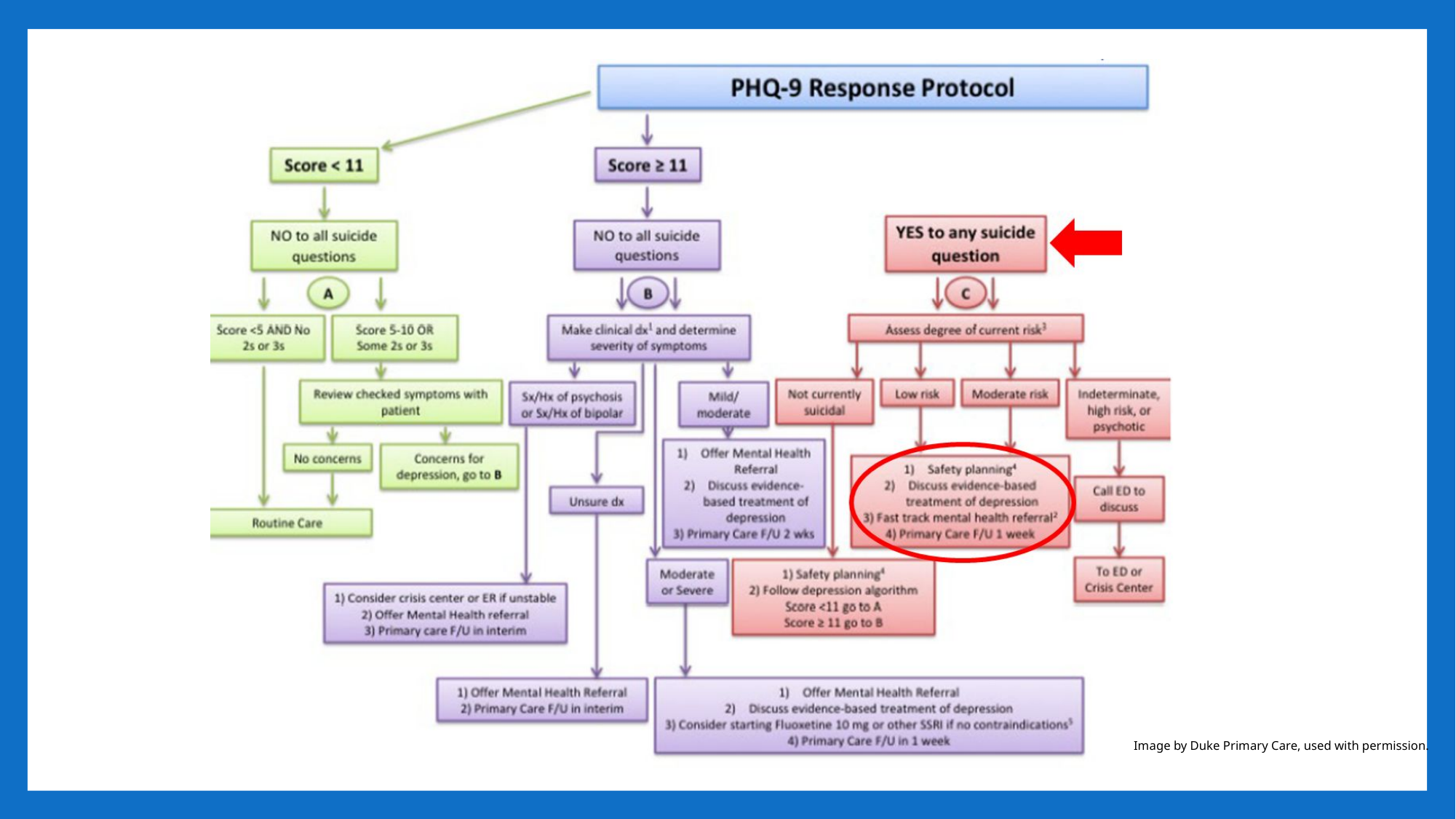

Image by Duke Primary Care, used with permission.

## Slide 24
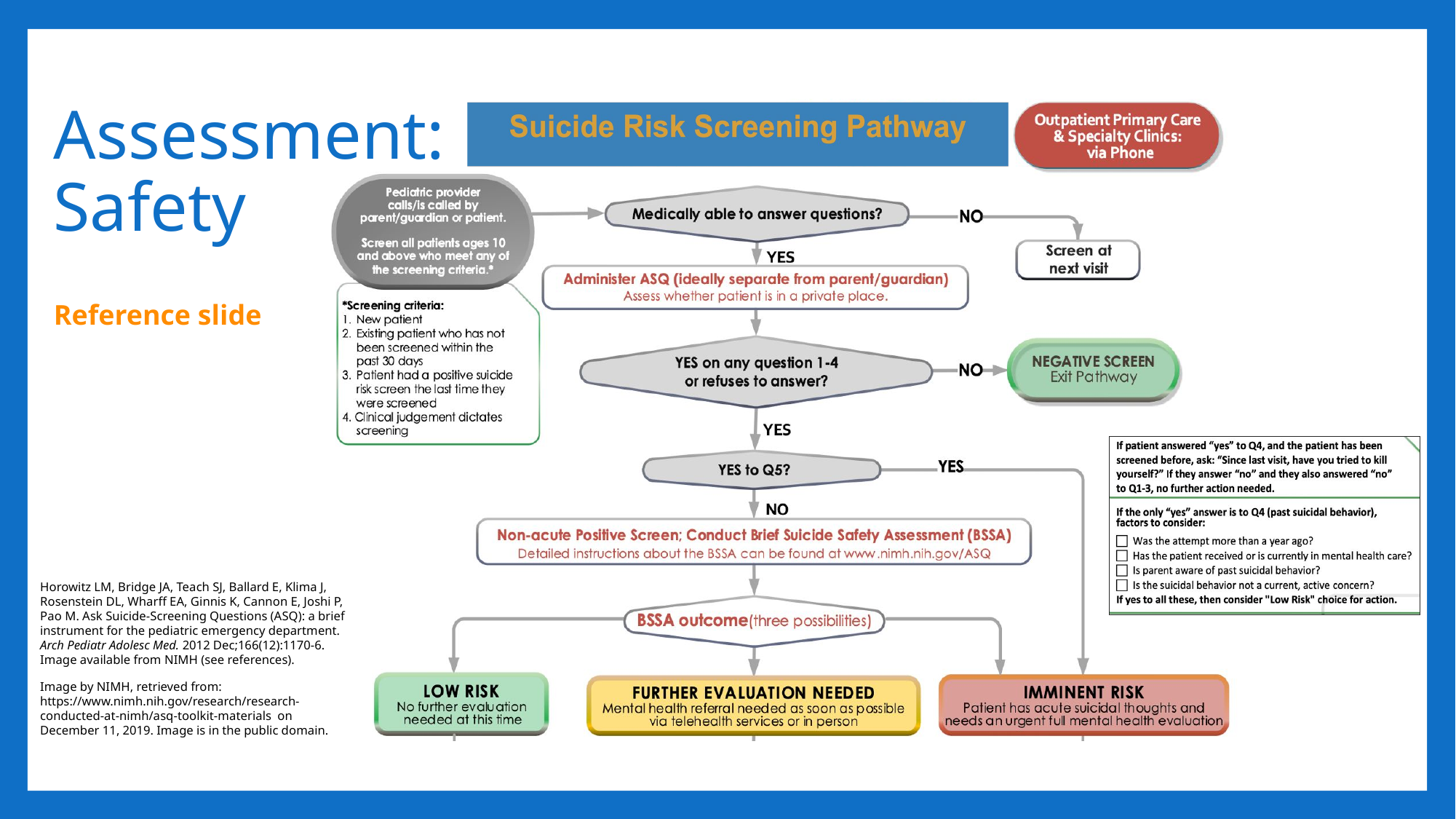

Assessment: Safety
Reference slide
Horowitz LM, Bridge JA, Teach SJ, Ballard E, Klima J, Rosenstein DL, Wharff EA, Ginnis K, Cannon E, Joshi P, Pao M. Ask Suicide-Screening Questions (ASQ): a brief instrument for the pediatric emergency department. Arch Pediatr Adolesc Med. 2012 Dec;166(12):1170-6. Image available from NIMH (see references).
Image by NIMH, retrieved from: https://www.nimh.nih.gov/research/research-conducted-at-nimh/asq-toolkit-materials on December 11, 2019. Image is in the public domain.

## Slide 25
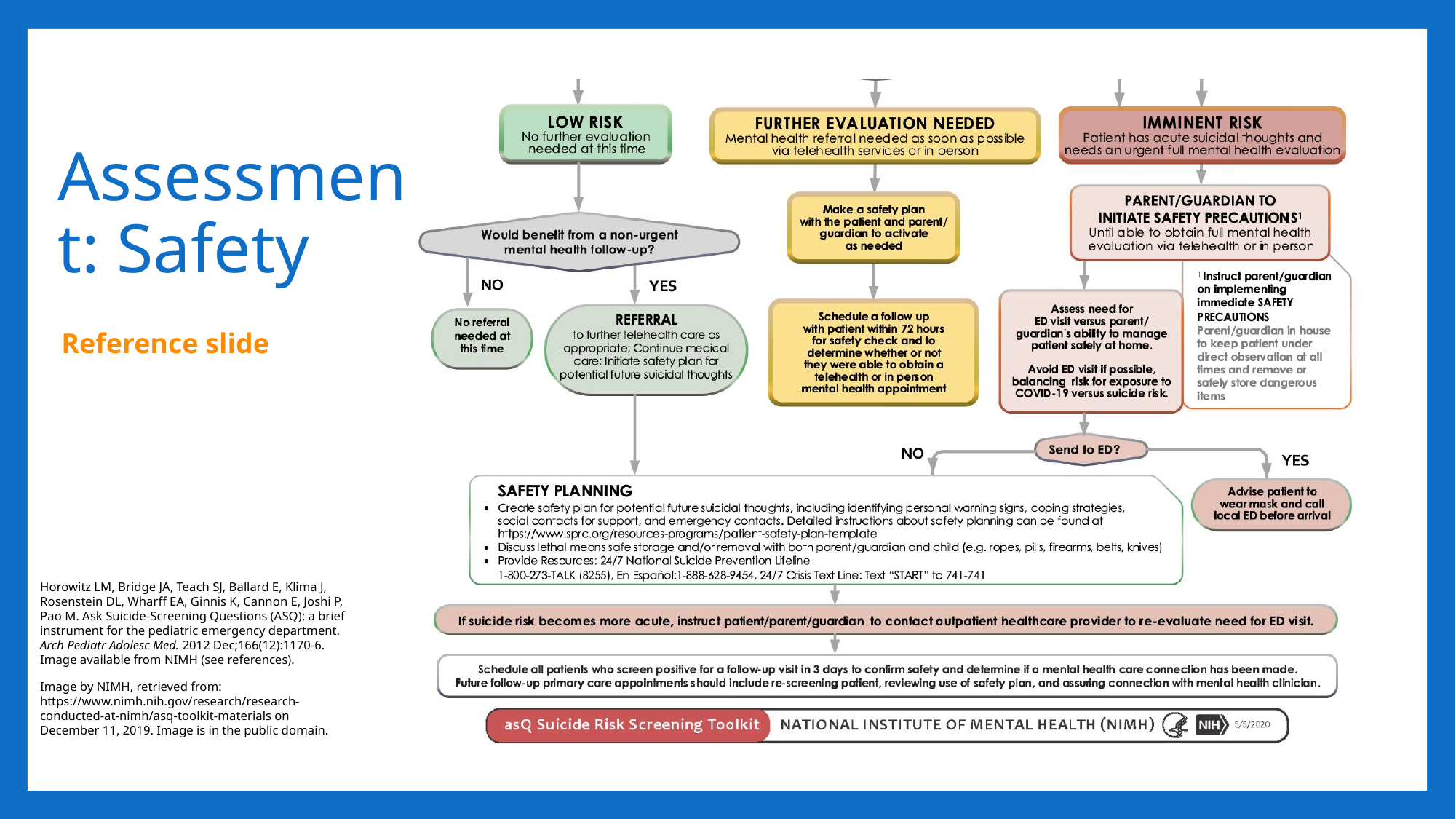

Assessment: Safety
Reference slide
Horowitz LM, Bridge JA, Teach SJ, Ballard E, Klima J, Rosenstein DL, Wharff EA, Ginnis K, Cannon E, Joshi P, Pao M. Ask Suicide-Screening Questions (ASQ): a brief instrument for the pediatric emergency department. Arch Pediatr Adolesc Med. 2012 Dec;166(12):1170-6. Image available from NIMH (see references).
Image by NIMH, retrieved from: https://www.nimh.nih.gov/research/research-conducted-at-nimh/asq-toolkit-materials on December 11, 2019. Image is in the public domain.

## Slide 26
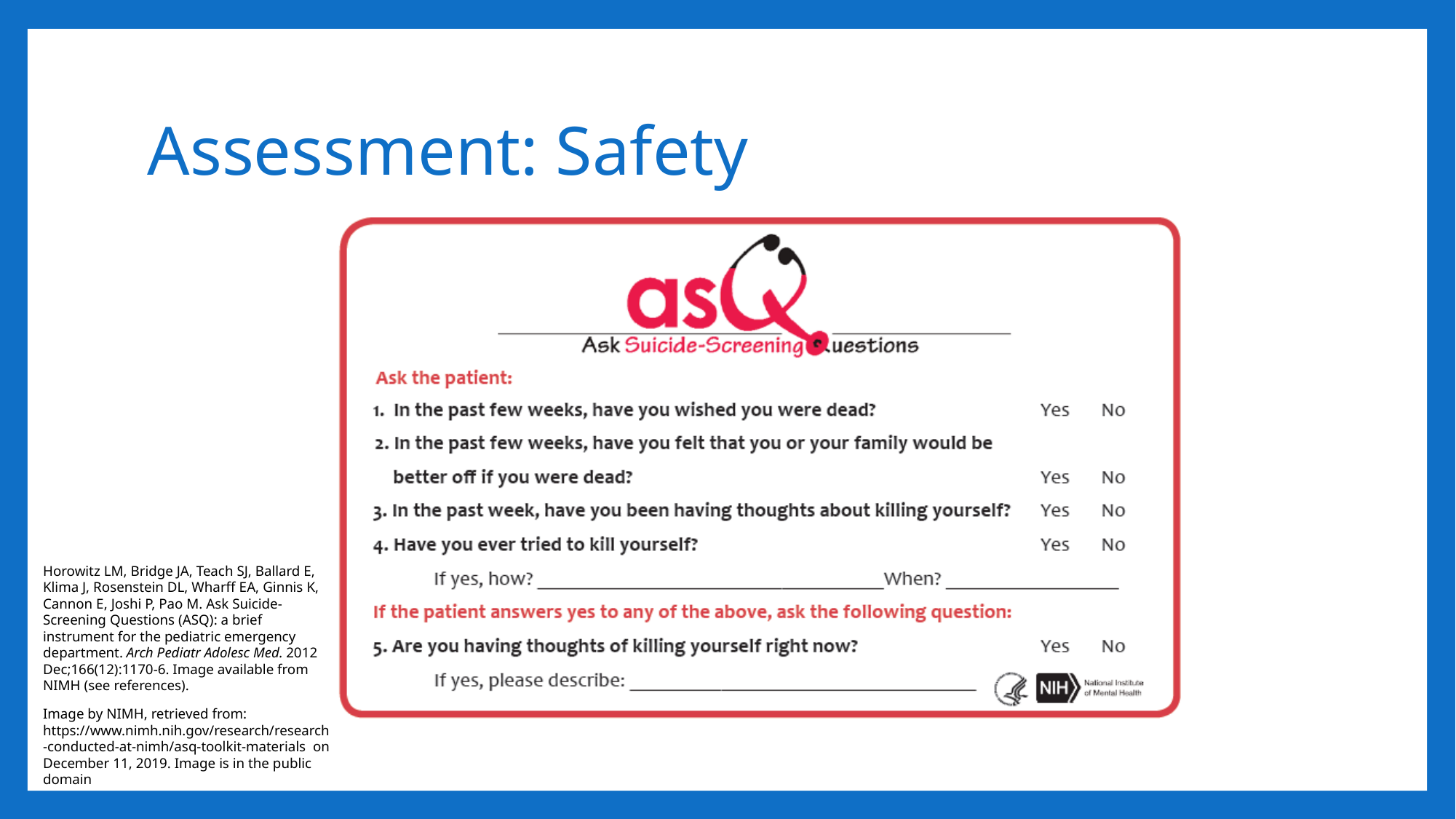

# Assessment: Safety
Horowitz LM, Bridge JA, Teach SJ, Ballard E, Klima J, Rosenstein DL, Wharff EA, Ginnis K, Cannon E, Joshi P, Pao M. Ask Suicide-Screening Questions (ASQ): a brief instrument for the pediatric emergency department. Arch Pediatr Adolesc Med. 2012 Dec;166(12):1170-6. Image available from NIMH (see references).
Image by NIMH, retrieved from: https://www.nimh.nih.gov/research/research-conducted-at-nimh/asq-toolkit-materials on December 11, 2019. Image is in the public domain

## Slide 27
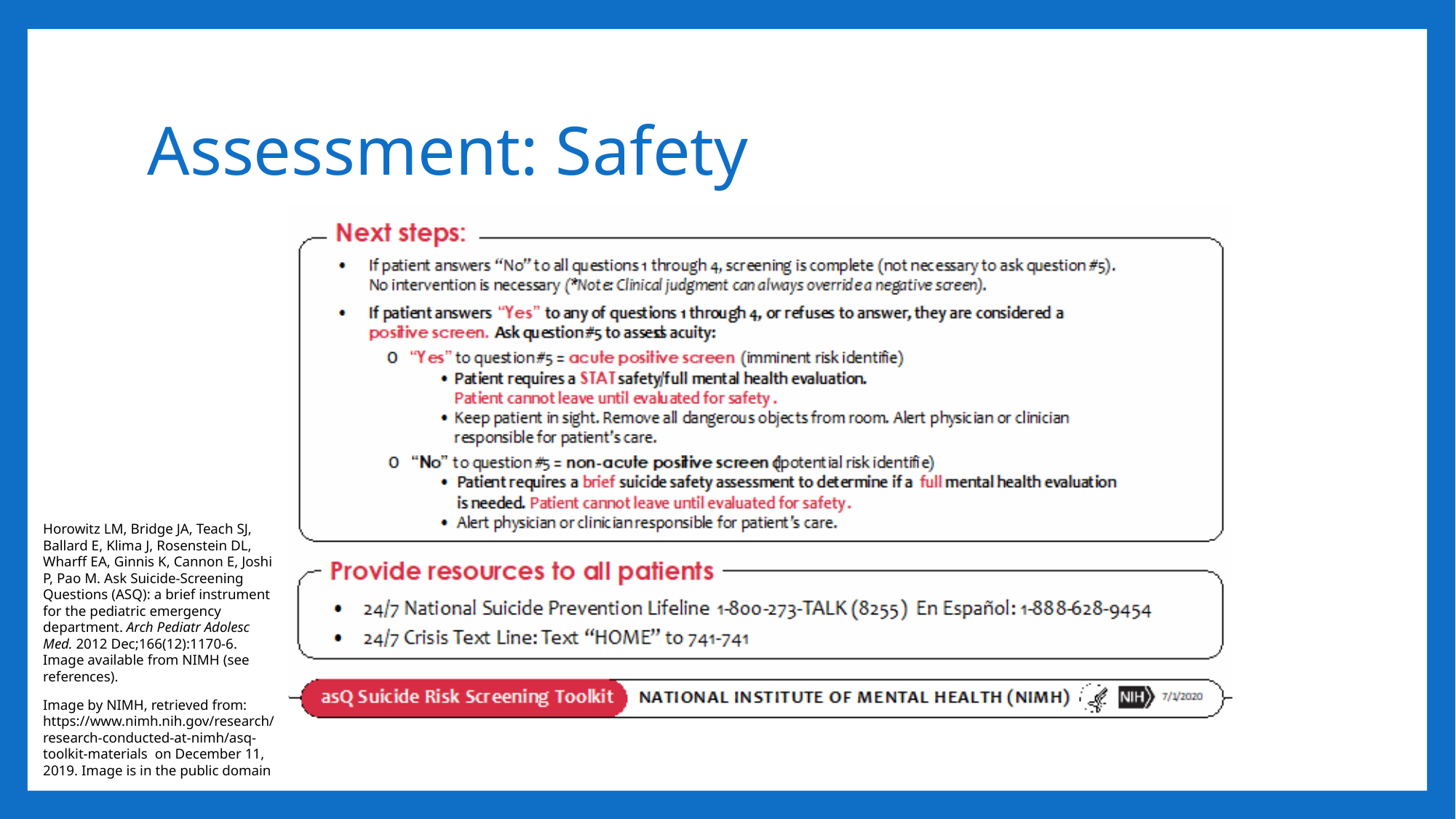

# Assessment: Safety
Horowitz LM, Bridge JA, Teach SJ, Ballard E, Klima J, Rosenstein DL, Wharff EA, Ginnis K, Cannon E, Joshi P, Pao M. Ask Suicide-Screening Questions (ASQ): a brief instrument for the pediatric emergency department. Arch Pediatr Adolesc Med. 2012 Dec;166(12):1170-6. Image available from NIMH (see references).
Image by NIMH, retrieved from: https://www.nimh.nih.gov/research/research-conducted-at-nimh/asq-toolkit-materials on December 11, 2019. Image is in the public domain

## Slide 28
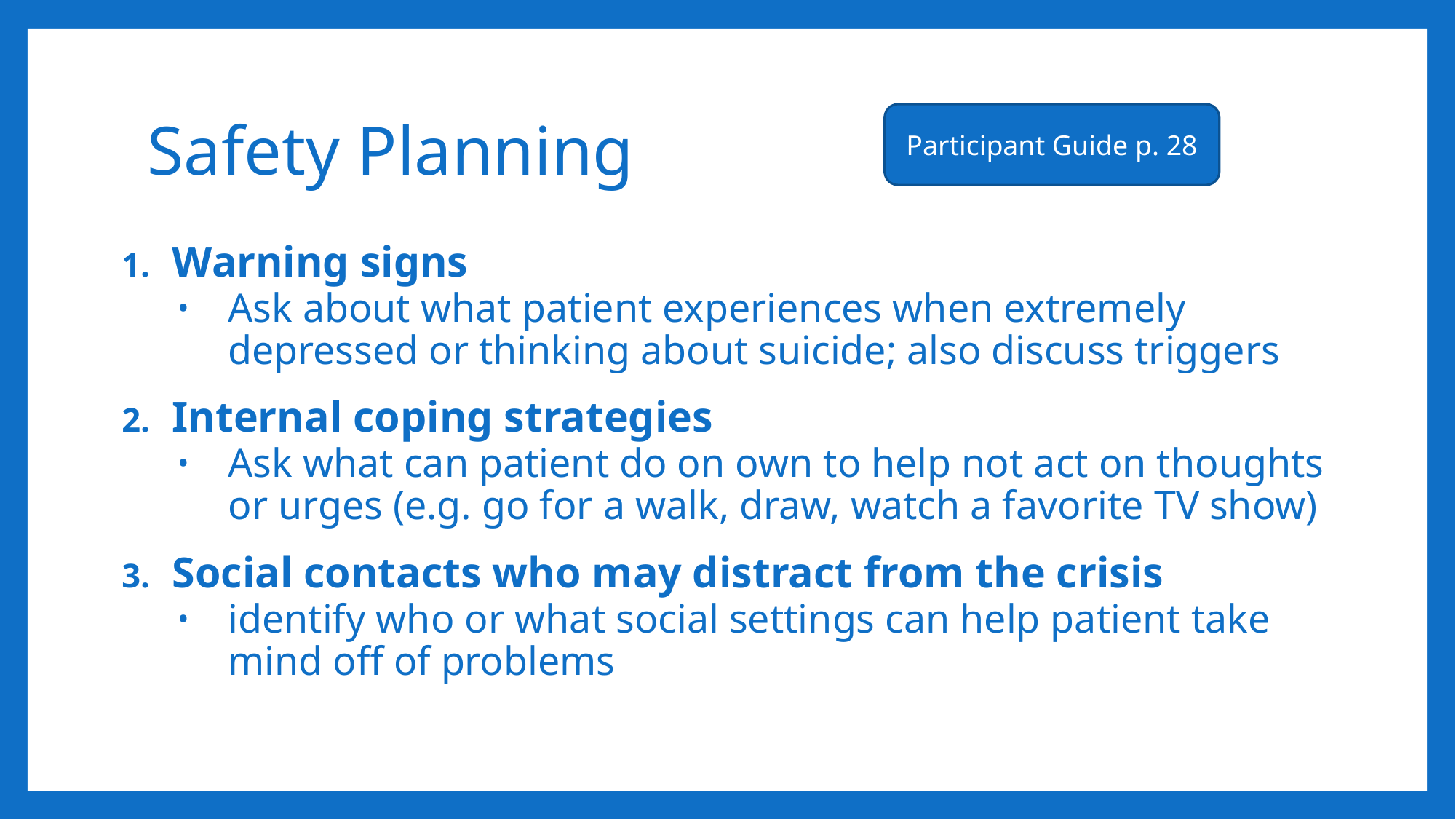

# Safety Planning
Participant Guide p. 28
Warning signs
Ask about what patient experiences when extremely depressed or thinking about suicide; also discuss triggers
Internal coping strategies
Ask what can patient do on own to help not act on thoughts or urges (e.g. go for a walk, draw, watch a favorite TV show)
Social contacts who may distract from the crisis
identify who or what social settings can help patient take mind off of problems

## Slide 29
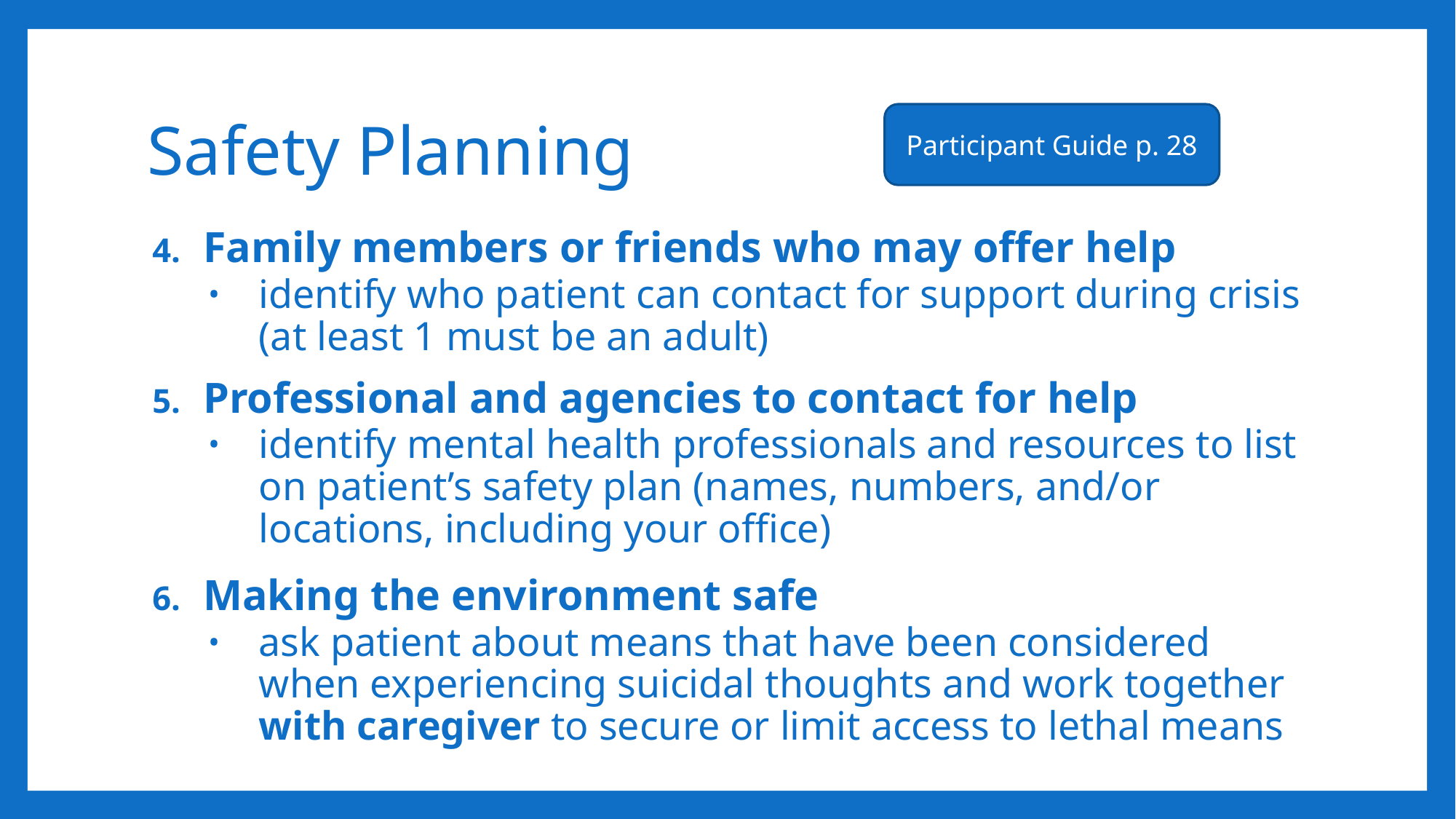

# Safety Planning
Participant Guide p. 28
Family members or friends who may offer help
identify who patient can contact for support during crisis (at least 1 must be an adult)
Professional and agencies to contact for help
identify mental health professionals and resources to list on patient’s safety plan (names, numbers, and/or locations, including your office)
Making the environment safe
ask patient about means that have been considered when experiencing suicidal thoughts and work together with caregiver to secure or limit access to lethal means

## Slide 30
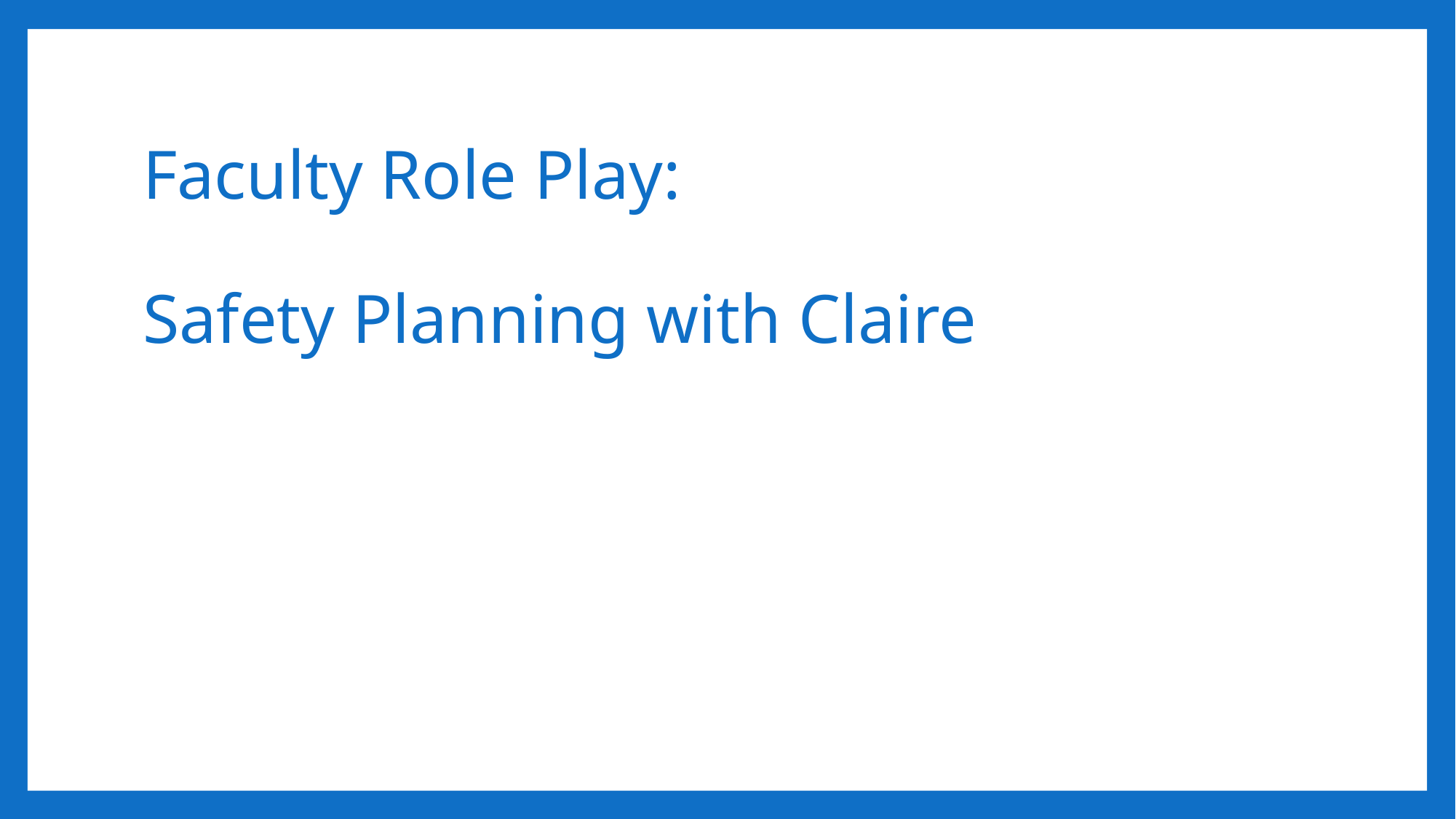

# Faculty Role Play: Safety Planning with Claire

## Slide 31
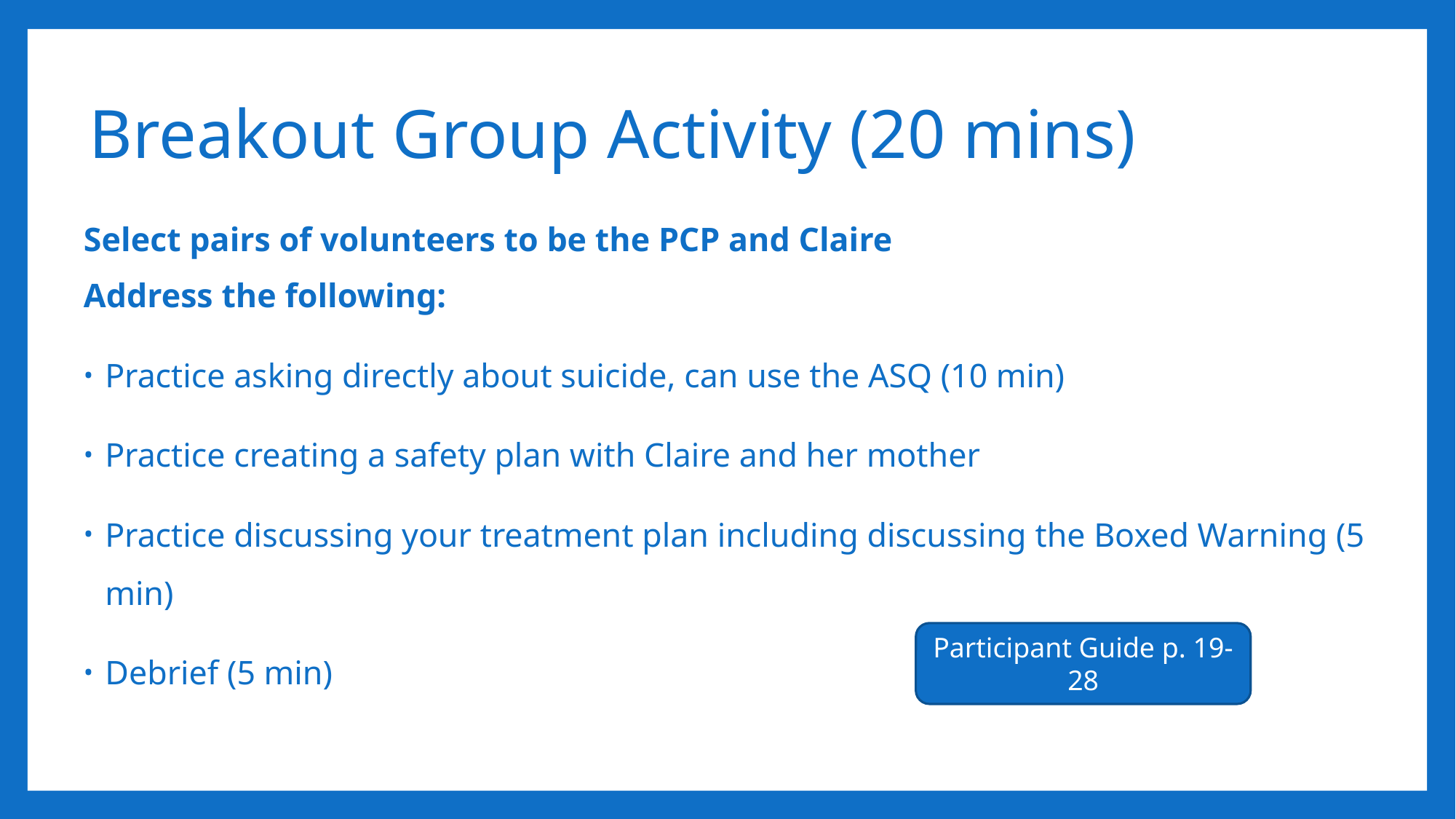

# Breakout Group Activity (20 mins)
Select pairs of volunteers to be the PCP and Claire
Address the following:
Practice asking directly about suicide, can use the ASQ (10 min)
Practice creating a safety plan with Claire and her mother
Practice discussing your treatment plan including discussing the Boxed Warning (5 min)
Debrief (5 min)
Participant Guide p. 19-28

## Slide 32
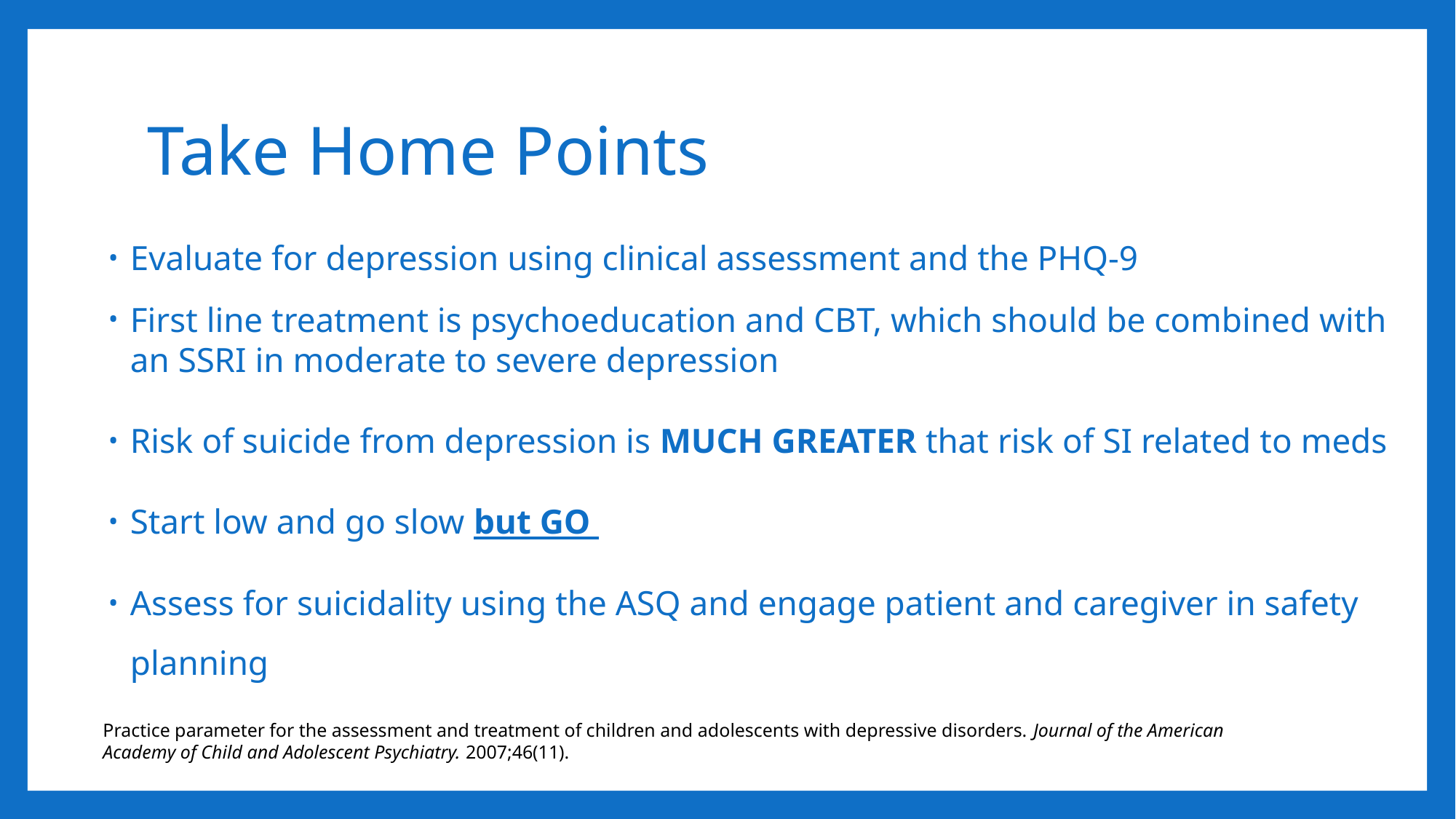

# Take Home Points
Evaluate for depression using clinical assessment and the PHQ-9
First line treatment is psychoeducation and CBT, which should be combined with an SSRI in moderate to severe depression
Risk of suicide from depression is MUCH GREATER that risk of SI related to meds
Start low and go slow but GO
Assess for suicidality using the ASQ and engage patient and caregiver in safety planning
Practice parameter for the assessment and treatment of children and adolescents with depressive disorders. Journal of the American Academy of Child and Adolescent Psychiatry. 2007;46(11).

## Slide 33
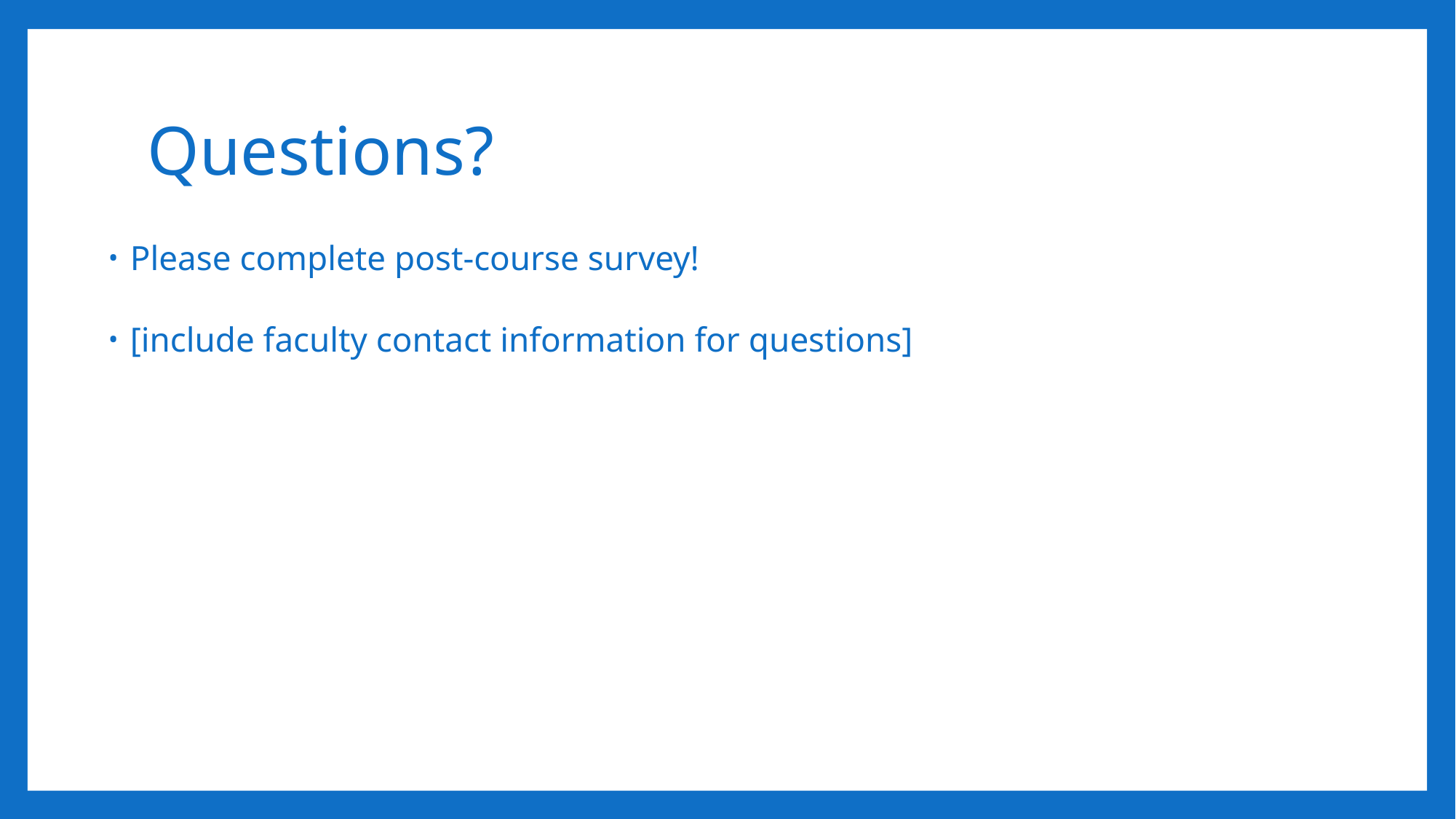

# Questions?
Please complete post-course survey!
[include faculty contact information for questions]
