# Supplementary material for: Be ExPeRT (Behavioral Health Expansion in Pediatric Residency Training): A Case-Based Seminar
Source: MedEdPORTAL. 2023 Aug 1;19:11326. doi: 10.15766/mep_2374-8265.11326 (PMC10392710; doi:10.15766/mep_2374-8265.11326)
Supplement: Supplementary file 1 — Facilitator Guide.docxBe ExPeRT Introduction.pptxADHD in Primary Care Pediatrics.pptxAnxiety in Primary Care Pediatrics.pptxDepression in Primary Care Pediatrics.pptxBe ExPeRT Reference Slides.pptxParticipant Guide.docxBe ExPeRT Postsurvey.docxBe ExPeRT Case Discussion Form.docxBe ExPeRT Presurvey.docx [file mep_2374-8265.11326-s001.zip › F. Be ExPeRT Reference Slides.pptx]

## Slide 1
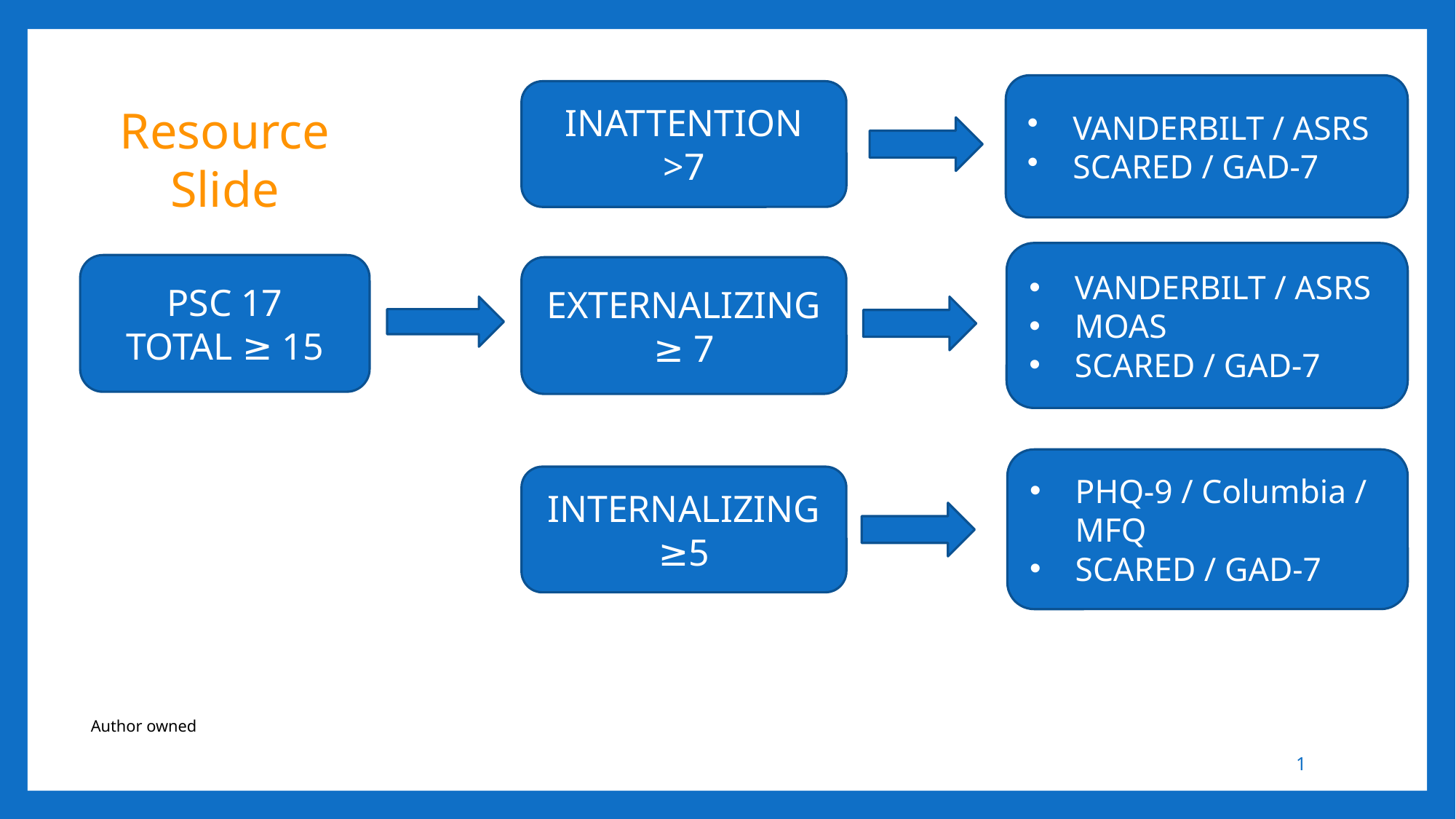

Resource Slide
VANDERBILT / ASRS
SCARED / GAD-7
INATTENTION
>7
VANDERBILT / ASRS
MOAS
SCARED / GAD-7
PSC 17
TOTAL ≥ 15
EXTERNALIZING
≥ 7
PHQ-9 / Columbia / MFQ
SCARED / GAD-7
INTERNALIZING
≥5
Author owned
1

## Slide 2
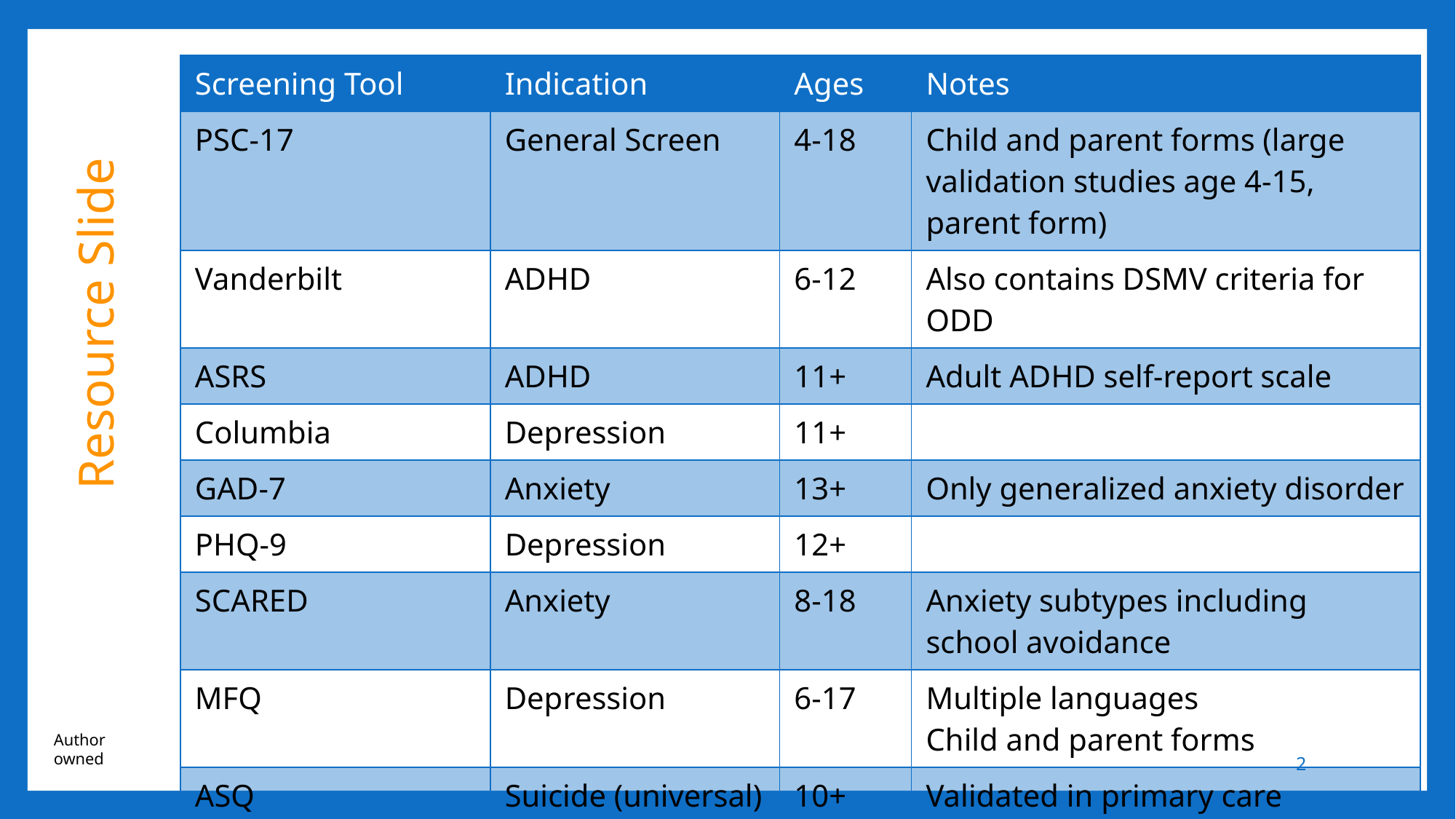

| Screening Tool | Indication | Ages | Notes |
| --- | --- | --- | --- |
| PSC-17 | General Screen | 4-18 | Child and parent forms (large validation studies age 4-15, parent form) |
| Vanderbilt | ADHD | 6-12 | Also contains DSMV criteria for ODD |
| ASRS | ADHD | 11+ | Adult ADHD self-report scale |
| Columbia | Depression | 11+ | |
| GAD-7 | Anxiety | 13+ | Only generalized anxiety disorder |
| PHQ-9 | Depression | 12+ | |
| SCARED | Anxiety | 8-18 | Anxiety subtypes including school avoidance |
| MFQ | Depression | 6-17 | Multiple languages Child and parent forms |
| ASQ | Suicide (universal) | 10+ | Validated in primary care |
| MDQ-A | Bipolar DO | 12-17 | |
Resource Slide
Author owned
2

## Slide 3
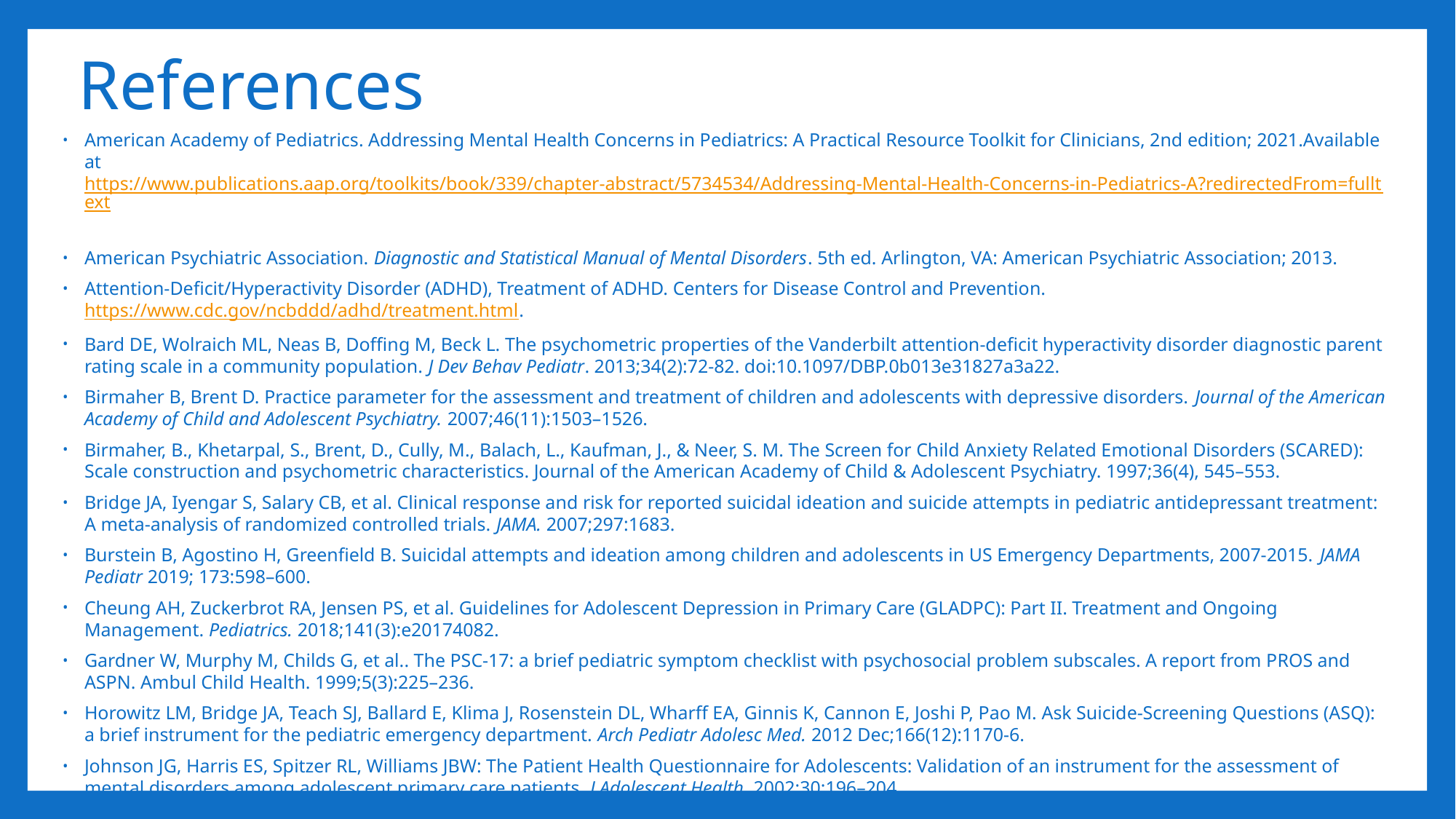

# References
American Academy of Pediatrics. Addressing Mental Health Concerns in Pediatrics: A Practical Resource Toolkit for Clinicians, 2nd edition; 2021.Available at https://www.publications.aap.org/toolkits/book/339/chapter-abstract/5734534/Addressing-Mental-Health-Concerns-in-Pediatrics-A?redirectedFrom=fulltext
American Psychiatric Association. Diagnostic and Statistical Manual of Mental Disorders. 5th ed. Arlington, VA: American Psychiatric Association; 2013.
Attention-Deficit/Hyperactivity Disorder (ADHD), Treatment of ADHD. Centers for Disease Control and Prevention. https://www.cdc.gov/ncbddd/adhd/treatment.html.
Bard DE, Wolraich ML, Neas B, Doffing M, Beck L. The psychometric properties of the Vanderbilt attention-deficit hyperactivity disorder diagnostic parent rating scale in a community population. J Dev Behav Pediatr. 2013;34(2):72-82. doi:10.1097/DBP.0b013e31827a3a22.
Birmaher B, Brent D. Practice parameter for the assessment and treatment of children and adolescents with depressive disorders. Journal of the American Academy of Child and Adolescent Psychiatry. 2007;46(11):1503–1526.
Birmaher, B., Khetarpal, S., Brent, D., Cully, M., Balach, L., Kaufman, J., & Neer, S. M. The Screen for Child Anxiety Related Emotional Disorders (SCARED): Scale construction and psychometric characteristics. Journal of the American Academy of Child & Adolescent Psychiatry. 1997;36(4), 545–553.
Bridge JA, Iyengar S, Salary CB, et al. Clinical response and risk for reported suicidal ideation and suicide attempts in pediatric antidepressant treatment: A meta-analysis of randomized controlled trials. JAMA. 2007;297:1683.
Burstein B, Agostino H, Greenfield B. Suicidal attempts and ideation among children and adolescents in US Emergency Departments, 2007-2015. JAMA Pediatr 2019; 173:598–600.
Cheung AH, Zuckerbrot RA, Jensen PS, et al. Guidelines for Adolescent Depression in Primary Care (GLADPC): Part II. Treatment and Ongoing Management. Pediatrics. 2018;141(3):e20174082.
Gardner W, Murphy M, Childs G, et al.. The PSC-17: a brief pediatric symptom checklist with psychosocial problem subscales. A report from PROS and ASPN. Ambul Child Health. 1999;5(3):225–236.
Horowitz LM, Bridge JA, Teach SJ, Ballard E, Klima J, Rosenstein DL, Wharff EA, Ginnis K, Cannon E, Joshi P, Pao M. Ask Suicide-Screening Questions (ASQ): a brief instrument for the pediatric emergency department. Arch Pediatr Adolesc Med. 2012 Dec;166(12):1170-6.
Johnson JG, Harris ES, Spitzer RL, Williams JBW: The Patient Health Questionnaire for Adolescents: Validation of an instrument for the assessment of mental disorders among adolescent primary care patients. J Adolescent Health. 2002;30:196–204.
March J, Silva S, Petrycki S, et al. . Fluoxetine, cognitive-behavioral therapy, and their combination for adolescents with depression: treatment for adolescents with depression study (TADS) randomized controlled trial. JAMA. 2004;292:807–20.

## Slide 4
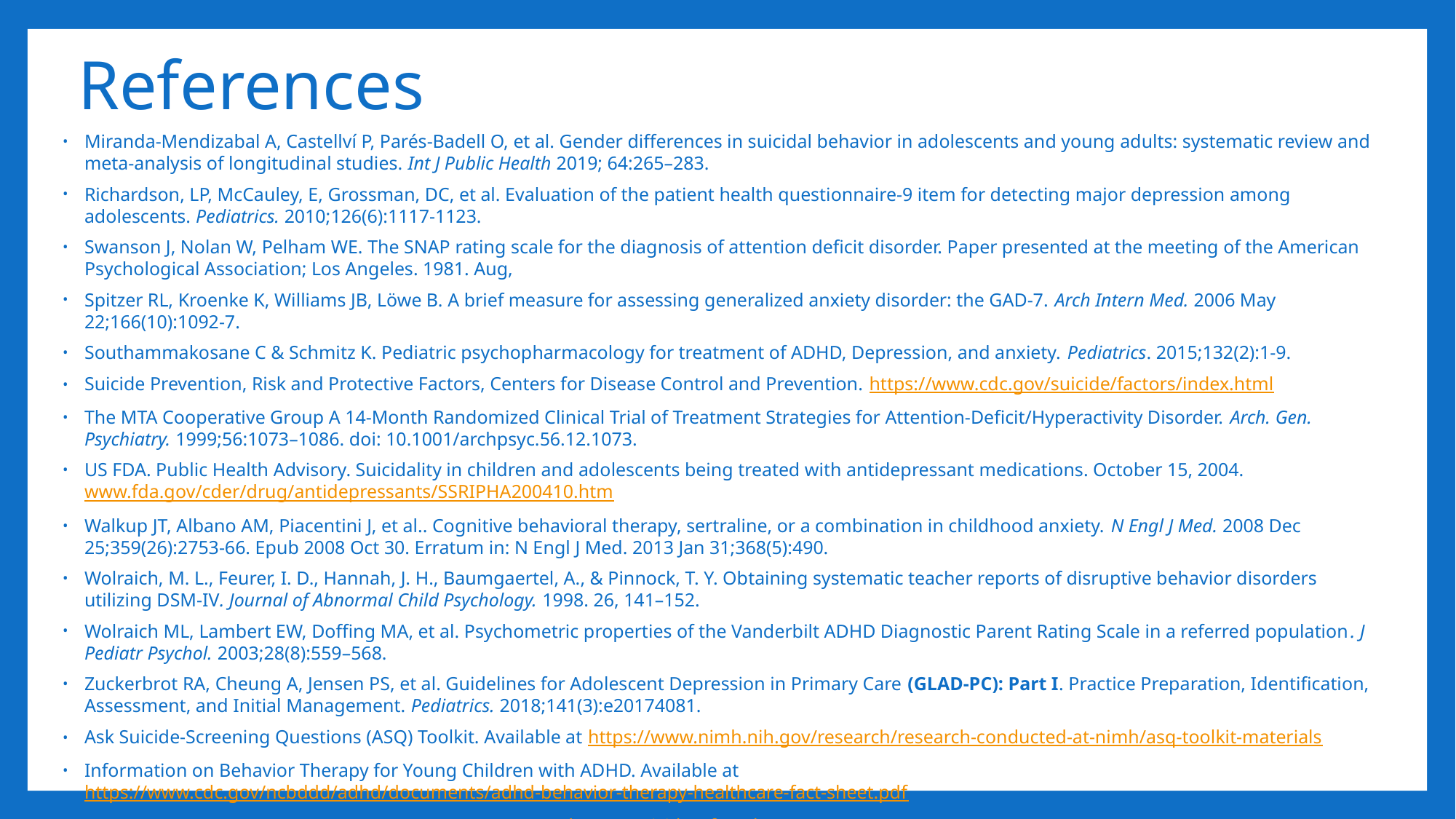

# References
Miranda-Mendizabal A, Castellví P, Parés-Badell O, et al. Gender differences in suicidal behavior in adolescents and young adults: systematic review and meta-analysis of longitudinal studies. Int J Public Health 2019; 64:265–283.
Richardson, LP, McCauley, E, Grossman, DC, et al. Evaluation of the patient health questionnaire-9 item for detecting major depression among adolescents. Pediatrics. 2010;126(6):1117-1123.
Swanson J, Nolan W, Pelham WE. The SNAP rating scale for the diagnosis of attention deficit disorder. Paper presented at the meeting of the American Psychological Association; Los Angeles. 1981. Aug,
Spitzer RL, Kroenke K, Williams JB, Löwe B. A brief measure for assessing generalized anxiety disorder: the GAD-7. Arch Intern Med. 2006 May 22;166(10):1092-7.
Southammakosane C & Schmitz K. Pediatric psychopharmacology for treatment of ADHD, Depression, and anxiety. Pediatrics. 2015;132(2):1-9.
Suicide Prevention, Risk and Protective Factors, Centers for Disease Control and Prevention. https://www.cdc.gov/suicide/factors/index.html
The MTA Cooperative Group A 14-Month Randomized Clinical Trial of Treatment Strategies for Attention-Deficit/Hyperactivity Disorder. Arch. Gen. Psychiatry. 1999;56:1073–1086. doi: 10.1001/archpsyc.56.12.1073.
US FDA. Public Health Advisory. Suicidality in children and adolescents being treated with antidepressant medications. October 15, 2004. www.fda.gov/cder/drug/antidepressants/SSRIPHA200410.htm
Walkup JT, Albano AM, Piacentini J, et al.. Cognitive behavioral therapy, sertraline, or a combination in childhood anxiety. N Engl J Med. 2008 Dec 25;359(26):2753-66. Epub 2008 Oct 30. Erratum in: N Engl J Med. 2013 Jan 31;368(5):490.
Wolraich, M. L., Feurer, I. D., Hannah, J. H., Baumgaertel, A., & Pinnock, T. Y. Obtaining systematic teacher reports of disruptive behavior disorders utilizing DSM-IV. Journal of Abnormal Child Psychology. 1998. 26, 141–152.
Wolraich ML, Lambert EW, Doffing MA, et al. Psychometric properties of the Vanderbilt ADHD Diagnostic Parent Rating Scale in a referred population. J Pediatr Psychol. 2003;28(8):559–568.
Zuckerbrot RA, Cheung A, Jensen PS, et al. Guidelines for Adolescent Depression in Primary Care (GLAD-PC): Part I. Practice Preparation, Identification, Assessment, and Initial Management. Pediatrics. 2018;141(3):e20174081.
Ask Suicide-Screening Questions (ASQ) Toolkit. Available at https://www.nimh.nih.gov/research/research-conducted-at-nimh/asq-toolkit-materials
Information on Behavior Therapy for Young Children with ADHD. Available at https://www.cdc.gov/ncbddd/adhd/documents/adhd-behavior-therapy-healthcare-fact-sheet.pdf.
Stanley-Brown Safety Planning Intervention. Available at https://suicidesafetyplan.com/
