# Supplementary material for: Be ExPeRT (Behavioral Health Expansion in Pediatric Residency Training): A Case-Based Seminar
Source: MedEdPORTAL. 2023 Aug 1;19:11326. doi: 10.15766/mep_2374-8265.11326 (PMC10392710; doi:10.15766/mep_2374-8265.11326)

**MedEdPORTAL**

**Appendix G: Participant Guide**

**Table of Contents**

1. **Unit 1: ADHD in Primary Care Pediatrics**
   1. Case Scenario Description and Screening Forms pp. 2-6
   2. Resources pp. 7-10
      1. PSC-17 Rating Scale
      2. Behavioral Therapies for ADHD
      3. ADHD Algorithm
2. **Unit 2: Anxiety in Primary Care Pediatrics**
   1. Case Scenario Description and Screening Forms pp. 11-15
   2. Resources pp. 16-29
      1. SCARED Scale & Scoring
      2. GAD-7 Rating Scale
      3. Managing Anxiety: Tips for Families (English & Spanish)
3. **Unit 3: Depression in Primary Care Pediatrics**
   1. Case Scenario Description and Screening Forms pp. 30-31
   2. Resources pp. 32-43
      1. Suicide Risk Screening Pathway
      2. ASQ Screening Form
      3. ASQ Toolkit: Youth Outpatient
      4. ASQ Toolkit: Parent/Guardian Flyer
      5. Sample Safety Plan Template
      6. Safety Plan Assessment Guide
      7. Anxiety and Depression Medication Guide
      8. GLAD-PC Clinical Assessment Flowchart
      9. Resource Slides

**Unit 1: ADHD**

**Case:** You have a clinic appointment with Johnny, an 8-year-old boy in 2nd grade with no past

psychiatric history and no past medical history who presents with his caregivers for difficulties at school.

**In your breakout group discuss the following questions:**

1) What would you ask parents to clarify diagnosis?

2) Score Johnny's Vanderbilt (page 3)

3) Before you start the treatment, what medical screening would you do?

4) What side effects would you counsel about?

5) You started the patient on a short acting stimulant. He is now doing great staying focused in the morning, but is distracted in the afternoon and is having trouble completing chores at home in the evening. What would you do next?

6) The parents have questions about stopping the medication during the weekends

Wolraich ML, Lambert EW, Doffing MA, et al. Psychometric properties of the Vanderbilt ADHD Diagnostic Parent Rating Scale in a referred population*. J Pediatr Psychol.* 2003;28(8):559–568.

The Vanderbilt screening scales are available at no cost at https://www.nichq.org/resource/nichq-vanderbilt-assessment-scales.

Image by Wolraich et al., used with permission.

Wolraich ML, Lambert EW, Doffing MA, et al. Psychometric properties of the Vanderbilt ADHD Diagnostic Parent Rating Scale in a referred population*. J Pediatr Psychol.* 2003;28(8):559–568.

Image by Wolraich et al., retrieved from: https://www.nichq.org/resource/nichq-vanderbilt-assessment-scales on December 11, 2019. Image is in the public domain.

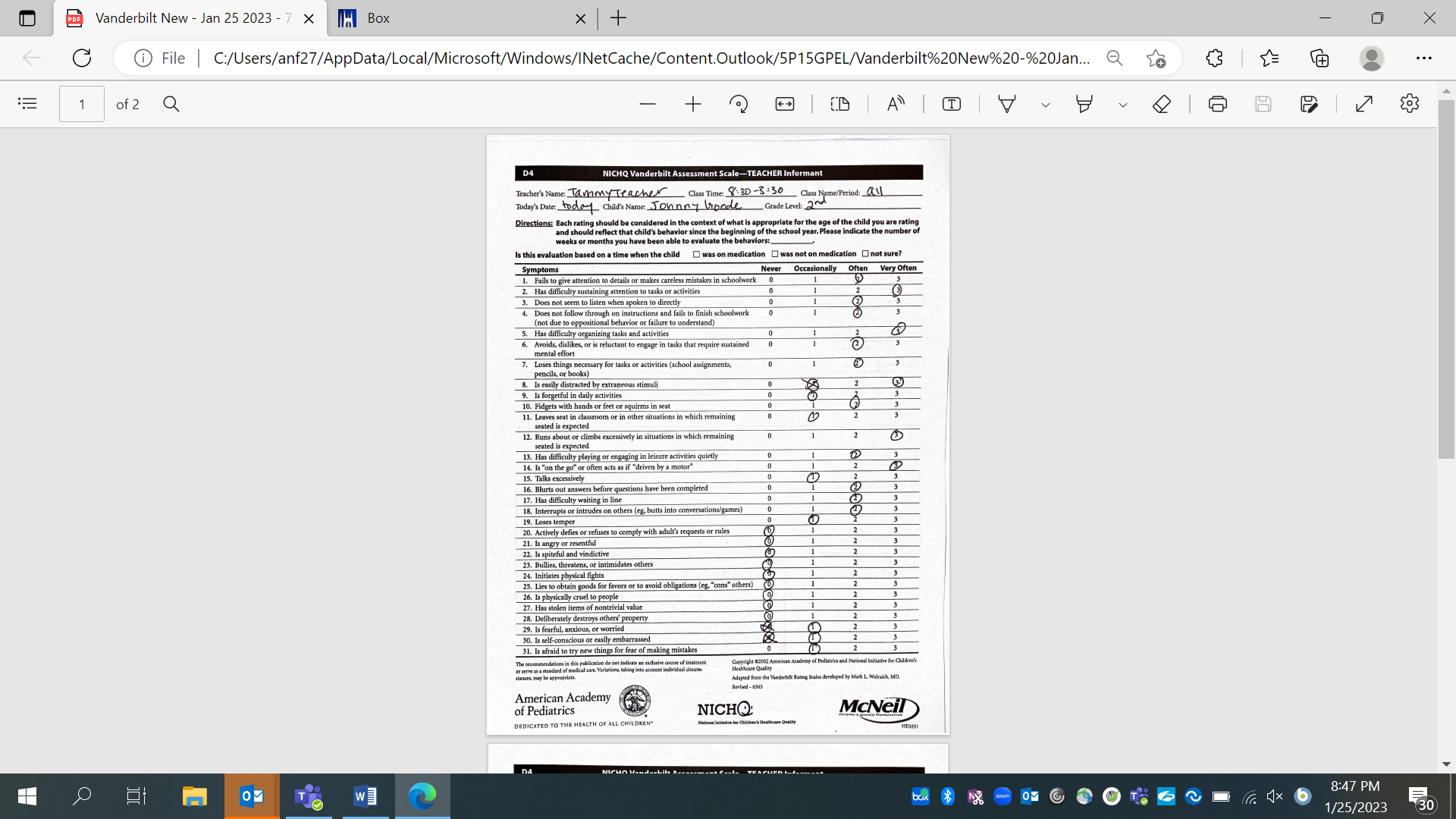


Wolraich, M. L., Feurer, I. D., Hannah, J. H., Baumgaertel, A., & Pinnock, T. Y. Obtaining systematic teacher reports of disruptive behavior disorders utilizing DSM-IV*. Journal of Abnormal Child Psychology*. 1998*.* 26, 141–152.

The Vanderbilt screening scales are available at no cost at https://www.nichq.org/resource/nichq-vanderbilt-assessment-scales.

Image by Wolraich et al., used with permission.


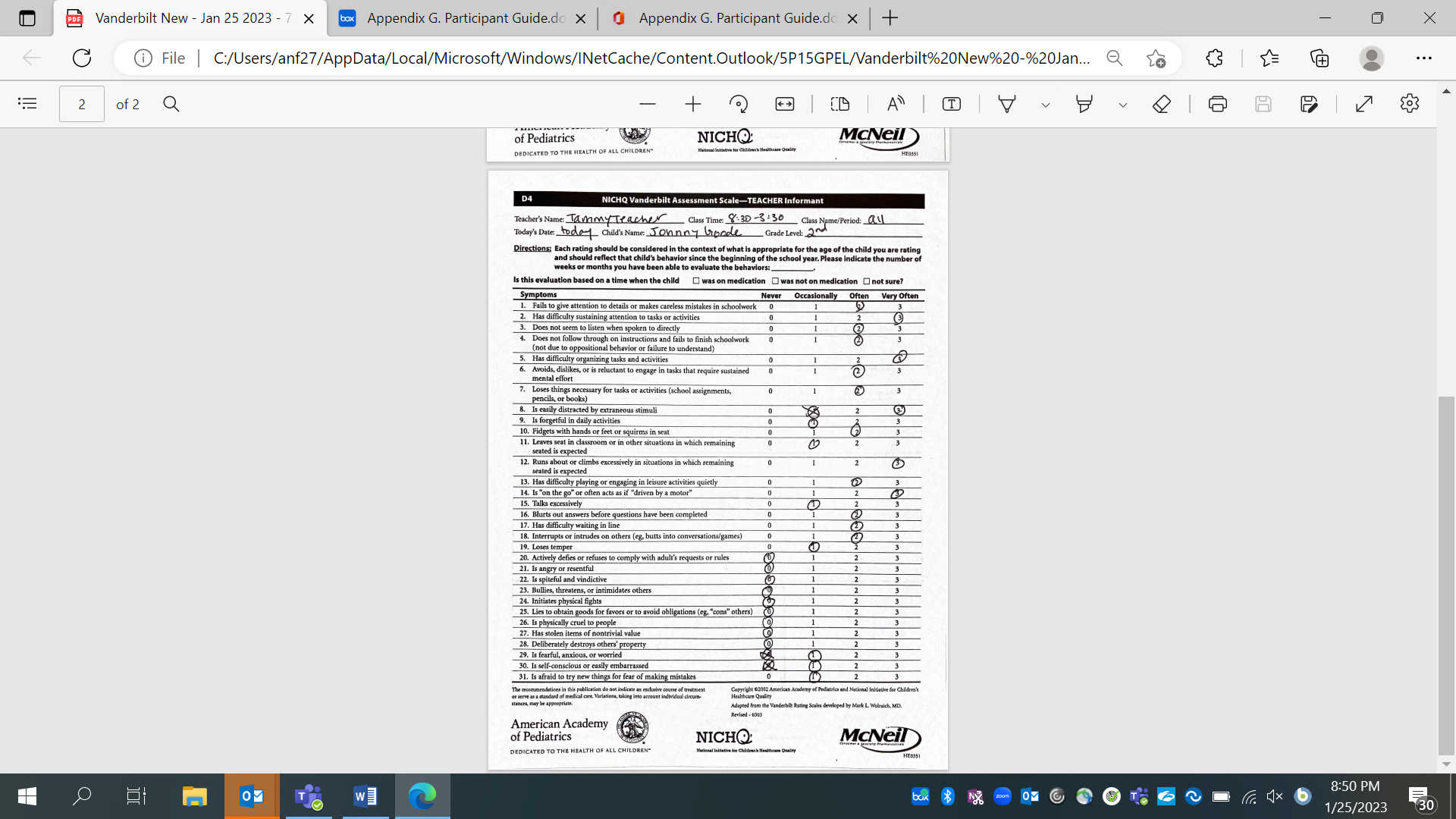

Wolraich, M. L., Feurer, I. D., Hannah, J. H., Baumgaertel, A., & Pinnock, T. Y. Obtaining systematic teacher reports of disruptive behavior disorders utilizing DSM-IV*. Journal of Abnormal Child Psychology*. 1998*.* 26, 141–152.

The Vanderbilt screening scales are available at no cost at https://www.nichq.org/resource/nichq-vanderbilt-assessment-scales.

Image by Wolraich et al., used with permission.


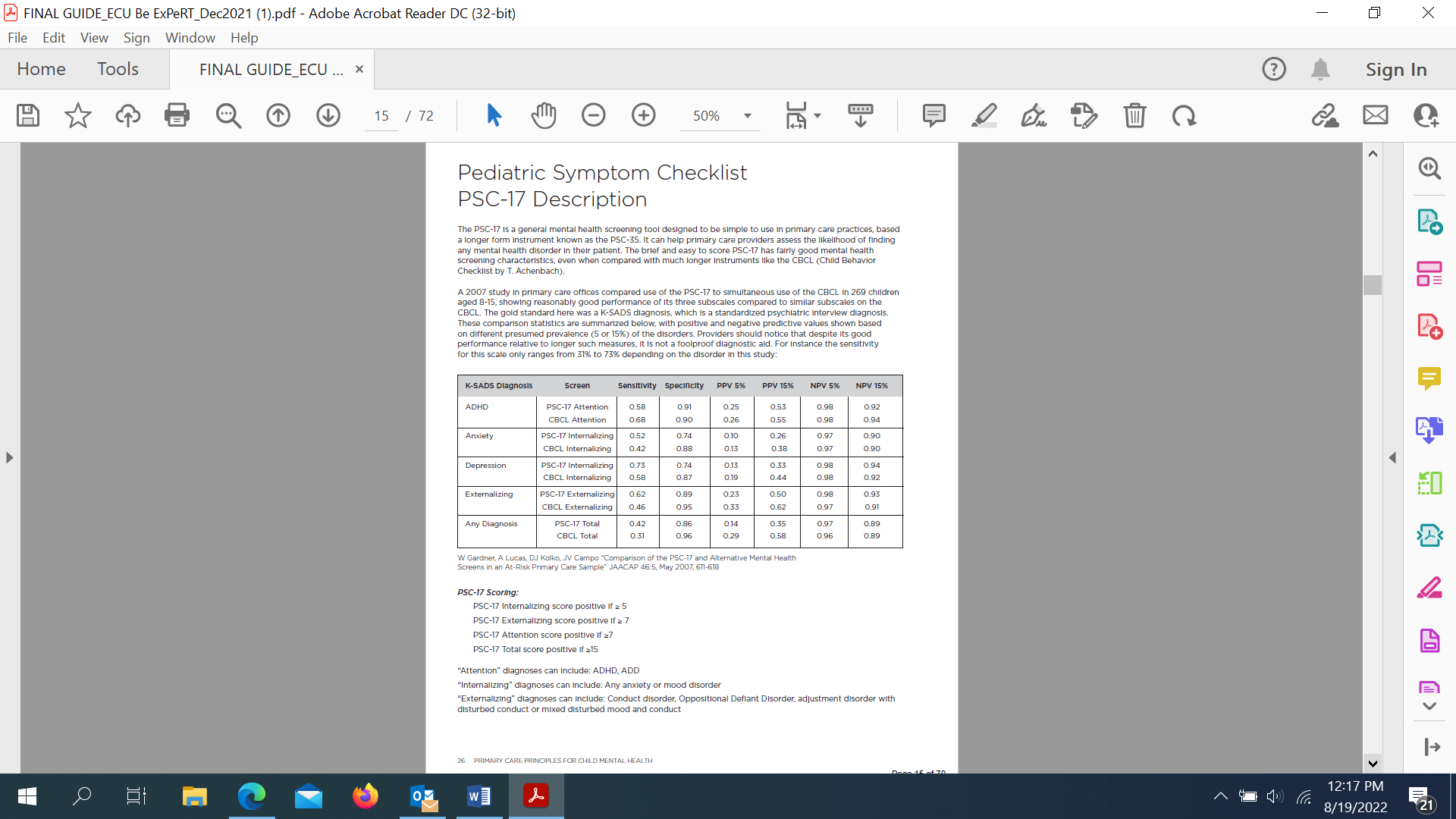


Gardner W, Murphy M, Childs G, et al.. The PSC-17: a brief pediatric symptom checklist with psychosocial problem subscales. A report from PROS and ASPN. *Ambul Child Health*. 1999;5(3):225–236

Image by Gardner et al., retrieved from: https://www.seattlechildrens.org/globalassets/documents/healthcare-professionals/pal/ratings/psc-17-rating-scale.pdf on December 11, 2019. The PSC-17 is freely reproduced.


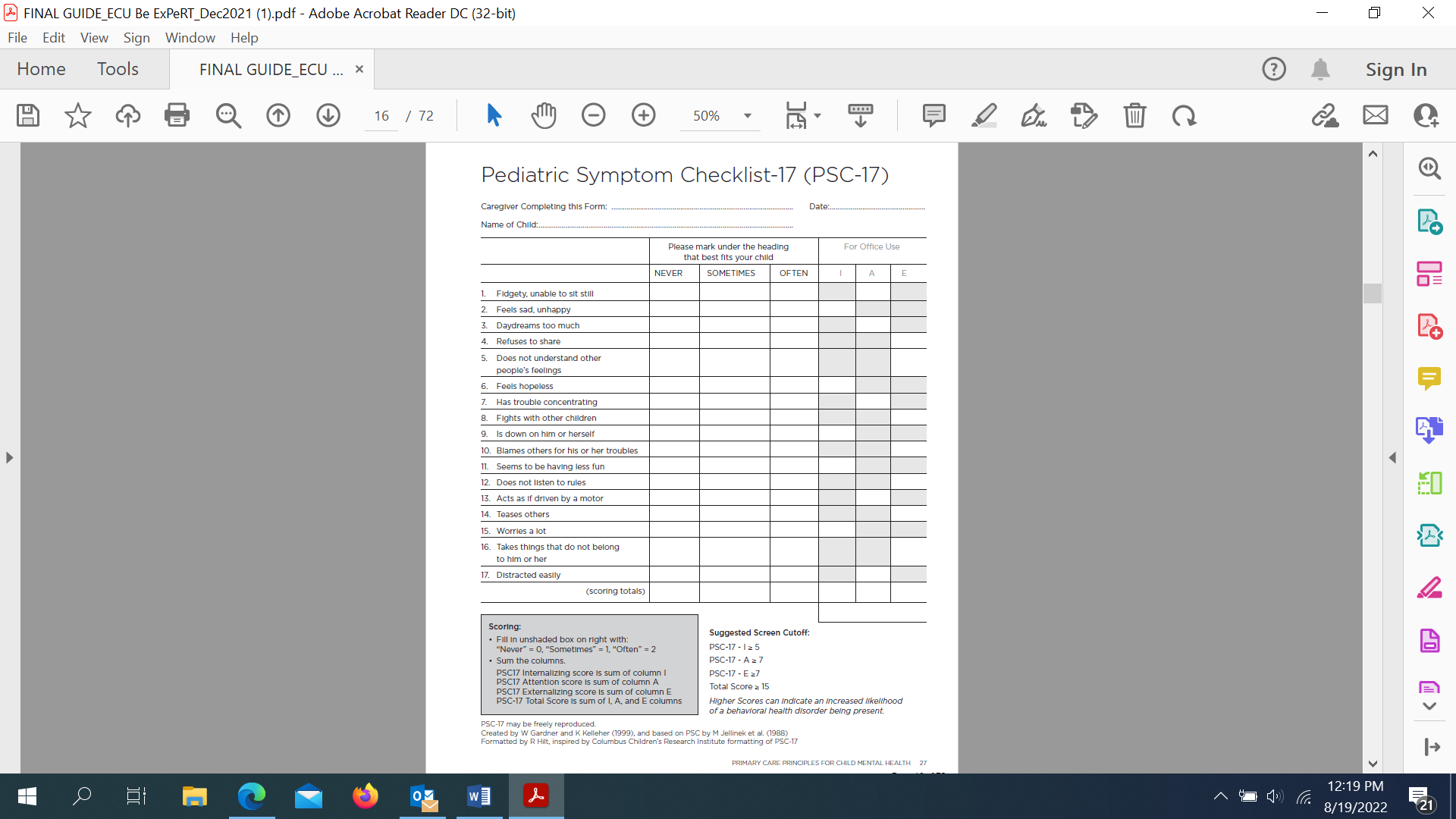


Gardner W, Murphy M, Childs G, et al.. The PSC-17: a brief pediatric symptom checklist with psychosocial problem subscales. A report from PROS and ASPN. *Ambul Child Health*. 1999;5(3):225–236

Image by Gardner et al., retrieved from: https://www.seattlechildrens.org/globalassets/documents/healthcare-professionals/pal/ratings/psc-17-rating-scale.pdf on December 11, 2019. The PSC-17 is freely reproduced.


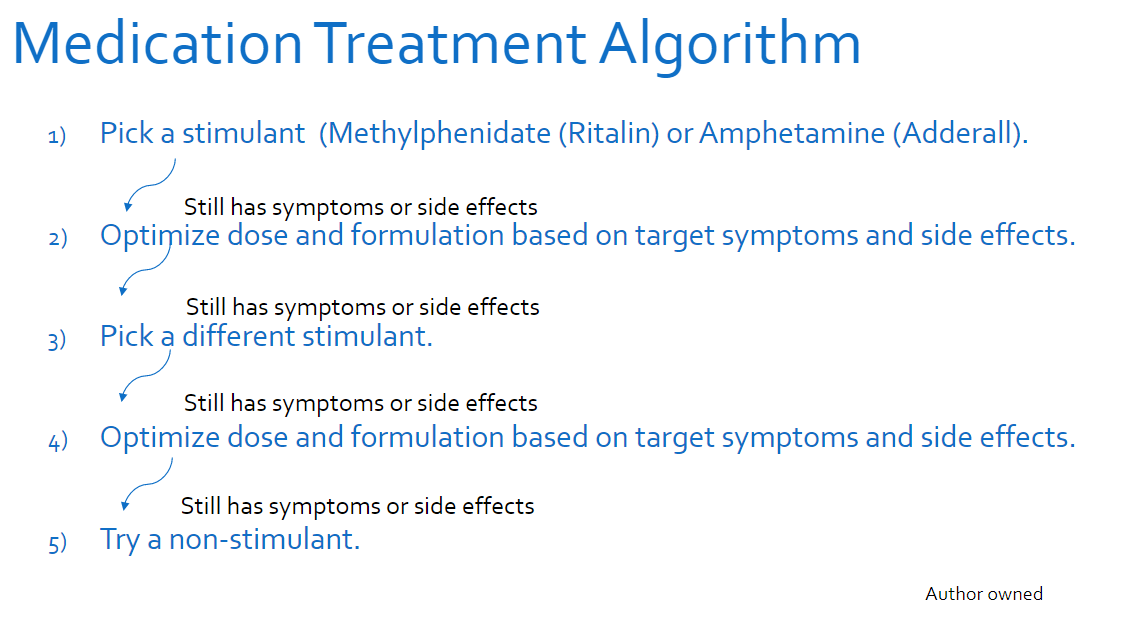


**Unit 2: Anxiety**

**Case:** Leila is a 10-year-old girl who presents at the recommendation of her school for daily belly aches causing her to miss a significant amount of school.

- **Leila**: *You worry about your parents’ safety since mom was in a car accident 3 years ago. You call mom repeatedly when she is not at home to make sure she is safe. You also really worry about doing well in school; you don’t speak up in class because of what other kids will think of your or if you get the answer wrong. You can't stop biting you nails which bothers mom. You almost threw up before a test last week because you felt so scared. You have trouble falling asleep at night for months, you worry about someone breaking into your home. It’s hard to concentrate on homework or chores and your parents have to remind you several times. You feel guilty because mom has to go to a meeting with your teacher and you worry about this impact on her baby brother. Your head and your belly hurt worse in the mornings and Sunday night, but not on weekends.*
- **Caregiver**: *You are concerned about a more serious problem with Leila’s stomach and would like more tests done. Your father was diagnosed with colon cancer last year. Leila typically gets good grades in school although they have declined slightly this year which is concerning to you. Leila has always been a “sensitive” and “shy” child. You’d rather Leila stay home than risk vomiting at school but can’t continue to miss work. You are not aware of any bullying. Leila has been complaining of stomach and head pain since school started and you really want to figure out what is going on. Leila had a baby brother born last summer and you had post-partum anxiety at that time which resolved without treatment.*

**In your breakout group:**

1. Role play being the PCP talking with Leila's reluctant mother
   1. Address the following:
      1. Describe the diagnosis and your recommended treatment plan
      2. Provide psychoeducation about anxiety to Leila and her mom
2. Debrief and discuss the following:
   1. Ways to engage a reluctant parent
   2. What medication would you start? How much? For how long?
   3. When would you follow up?

Birmaher, B., Khetarpal, S., Brent, D., Cully, M., Balach, L., Kaufman, J., & Neer, S. M. The Screen for Child Anxiety Related Emotional Disorders (SCARED): Scale construction and psychometric characteristics. *Journal of the American Academy of Child & Adolescent Psychiatry.* 1997;36(4), 545–553.

The SCARED is available at no cost at www.pediatricbipolar.pitt.edu under resources/instruments.

Image by Birmaher et al., used with permission.

Leila

Birmaher, B., Khetarpal, S., Brent, D., Cully, M., Balach, L., Kaufman, J., & Neer, S. M. The Screen for Child Anxiety Related Emotional Disorders (SCARED): Scale construction and psychometric characteristics. *Journal of the American Academy of Child & Adolescent Psychiatry.* 1997;36(4), 545–553.

The SCARED is available at no cost at www.pediatricbipolar.pitt.edu under resources/instruments.

Image by Birmaher et al., used with permission.

Leila

Birmaher, B., Khetarpal, S., Brent, D., Cully, M., Balach, L., Kaufman, J., & Neer, S. M. The Screen for Child Anxiety Related Emotional Disorders (SCARED): Scale construction and psychometric characteristics. *Journal of the American Academy of Child & Adolescent Psychiatry.* 1997;36(4), 545–553.

The SCARED is available at no cost at www.pediatricbipolar.pitt.edu under resources/instruments.

Image by Birmaher et al., used with permission.

Birmaher, B., Khetarpal, S., Brent, D., Cully, M., Balach, L., Kaufman, J., & Neer, S. M. The Screen for Child Anxiety Related Emotional Disorders (SCARED): Scale construction and psychometric characteristics. *Journal of the American Academy of Child & Adolescent Psychiatry.* 1997;36(4), 545–553.

The SCARED is available at no cost at www.pediatricbipolar.pitt.edu under resources/instruments.

Image by Birmaher et al., used with permission.


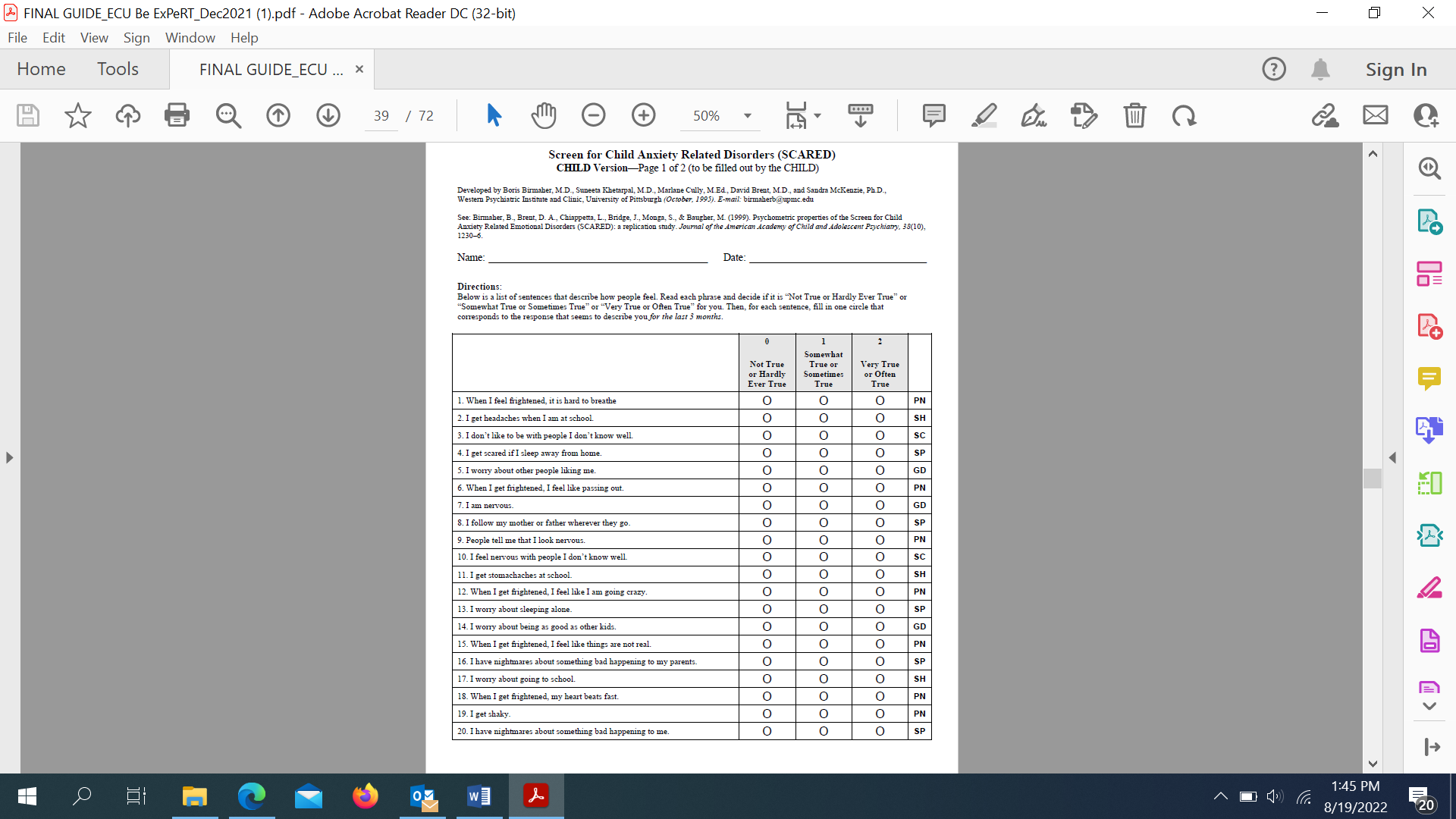


Birmaher, B., Khetarpal, S., Brent, D., Cully, M., Balach, L., Kaufman, J., & Neer, S. M. The Screen for Child Anxiety Related Emotional Disorders (SCARED): Scale construction and psychometric characteristics. *Journal of the American Academy of Child & Adolescent Psychiatry.* 1997;36(4), 545–553.

The SCARED is available at no cost at www.pediatricbipolar.pitt.edu under resources/instruments.

Image by Birmaher et al., used with permission.


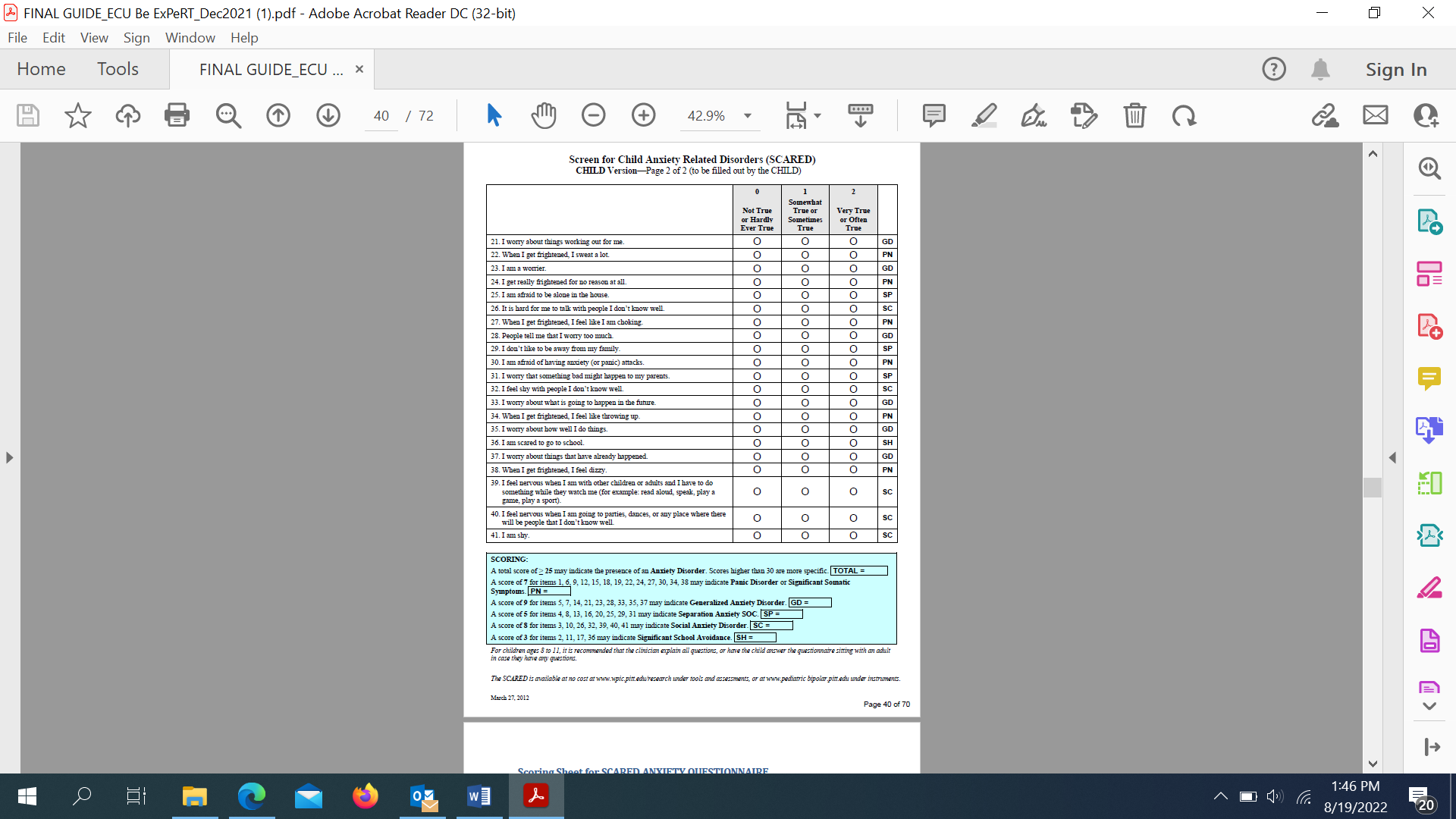


Birmaher, B., Khetarpal, S., Brent, D., Cully, M., Balach, L., Kaufman, J., & Neer, S. M. The Screen for Child Anxiety Related Emotional Disorders (SCARED): Scale construction and psychometric characteristics. *Journal of the American Academy of Child & Adolescent Psychiatry.* 1997;36(4), 545–553.

The SCARED is available at no cost at www.pediatricbipolar.pitt.edu under resources/instruments.

Image by Birmaher et al., used with permission.


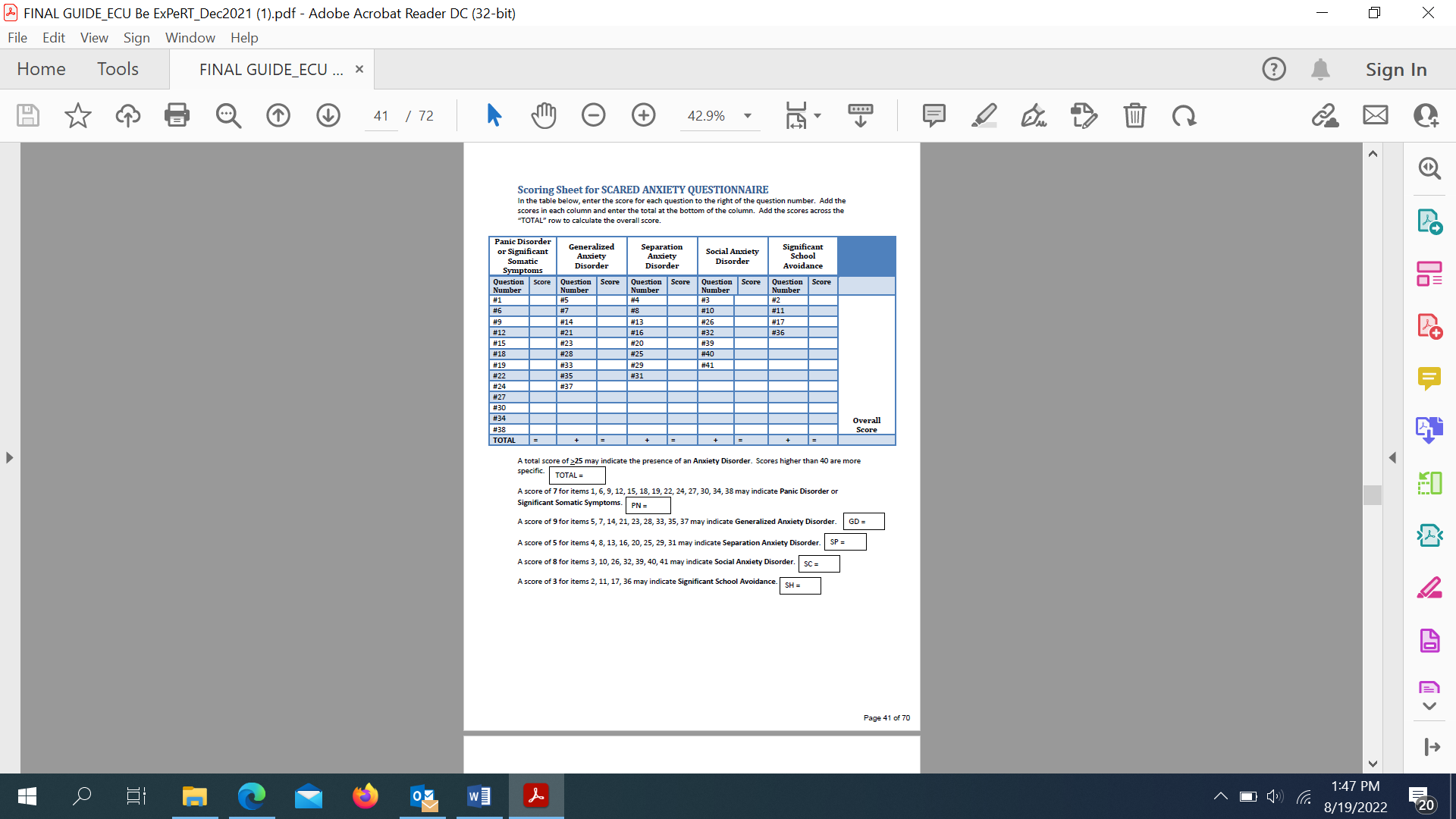


Birmaher, B., Khetarpal, S., Brent, D., Cully, M., Balach, L., Kaufman, J., & Neer, S. M. The Screen for Child Anxiety Related Emotional Disorders (SCARED): Scale construction and psychometric characteristics. *Journal of the American Academy of Child & Adolescent Psychiatry.* 1997;36(4), 545–553.

The SCARED is available at no cost at www.pediatricbipolar.pitt.edu under resources/instruments.

Image by Birmaher et al., used with permission.


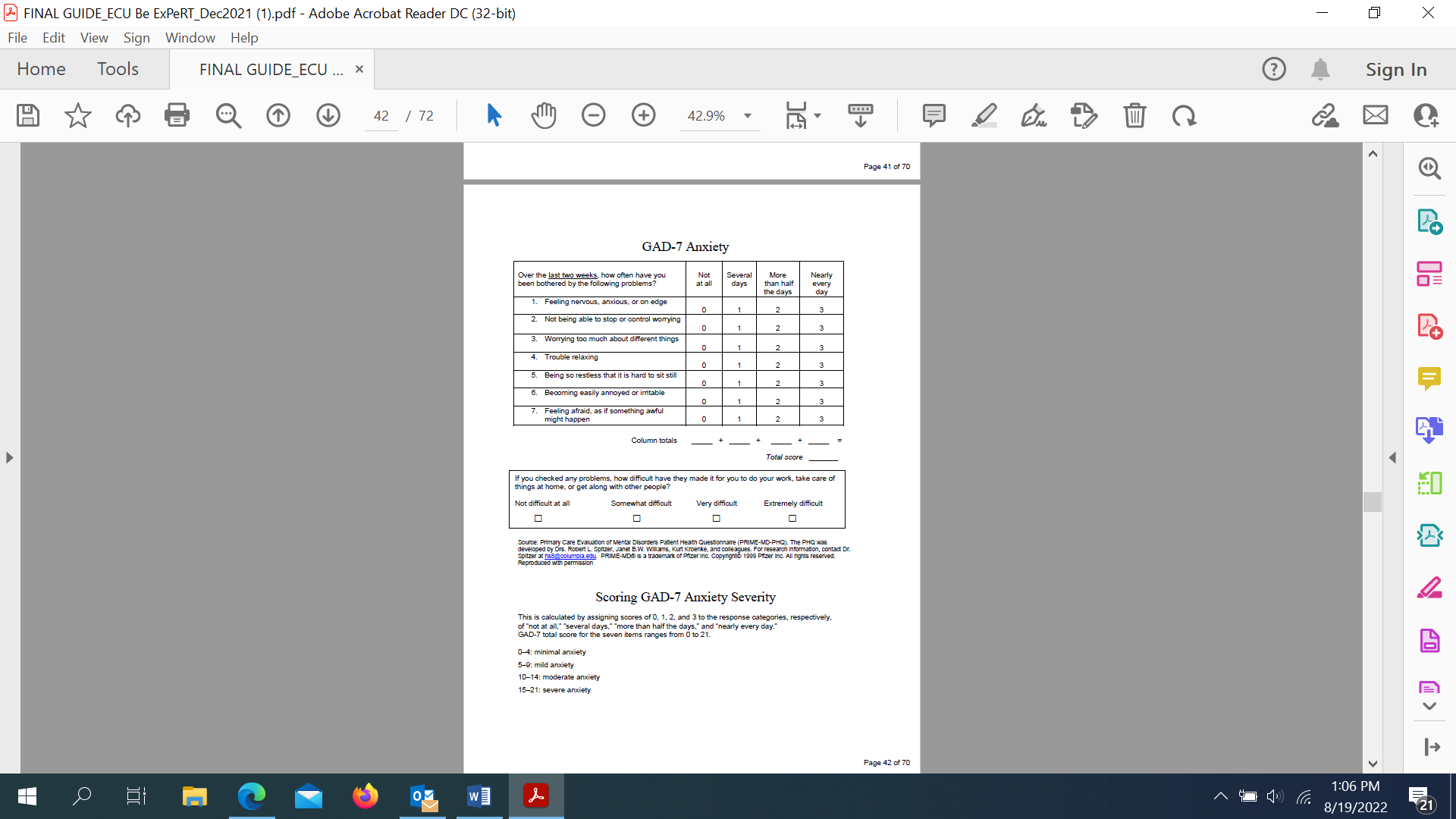


Spitzer RL, Kroenke K, Williams JB, Löwe B. A brief measure for assessing generalized anxiety disorder: the GAD-7. Arch Intern Med. 2006 May 22;166(10):1092-7.

Image by Spitzer et al., retrieved from: https://adaa.org/sites/default/files/GAD-7_Anxiety-updated_0.pdf on December 11, 2019. No permission required to reproduce, translate, display or distribute the GAD-7.

**Depression**

**Case:**

**Claire**: *You notice that you start to become suicidal when you feel that you’ve made a mistake or are*

*embarrassed in a social situation. You also notice that when you cannot stop crying you feel suicidal*

*and hopeless. Listening to music or playing your ukulele can help you feel better. You also feel better*

*when you are at swim practice or at the pool. You have a best friend whom you tell everything. You*

*are close with your swim coach and science teacher. You want to be a doctor when you grow up. You*

*don’t think you would actually ever kill yourself because your mom would be devastated. You are*

*hoping that your swim team makes it to finals this year but when you’re really depressed none of*

*that seems to matter.*

**Claire’s parent:** *You have a safe in which you can store medications. You can administer medications for Claire.*

*Claire’s father agrees that she can stay at your house until she is feeling better as the moving from*

*house to house in the context of your separation has been stressful for Claire.*

**In your breakout group:**

- Pair up and role play as Claire and her PCP
- Practice asking directly about suicide, can use the ASQ (10 min)
- Practice creating a safety plan with Claire and her mother
- Practice discussing your treatment plan including discussing the Boxed Warning (10 min)
- Discuss means restriction, if time


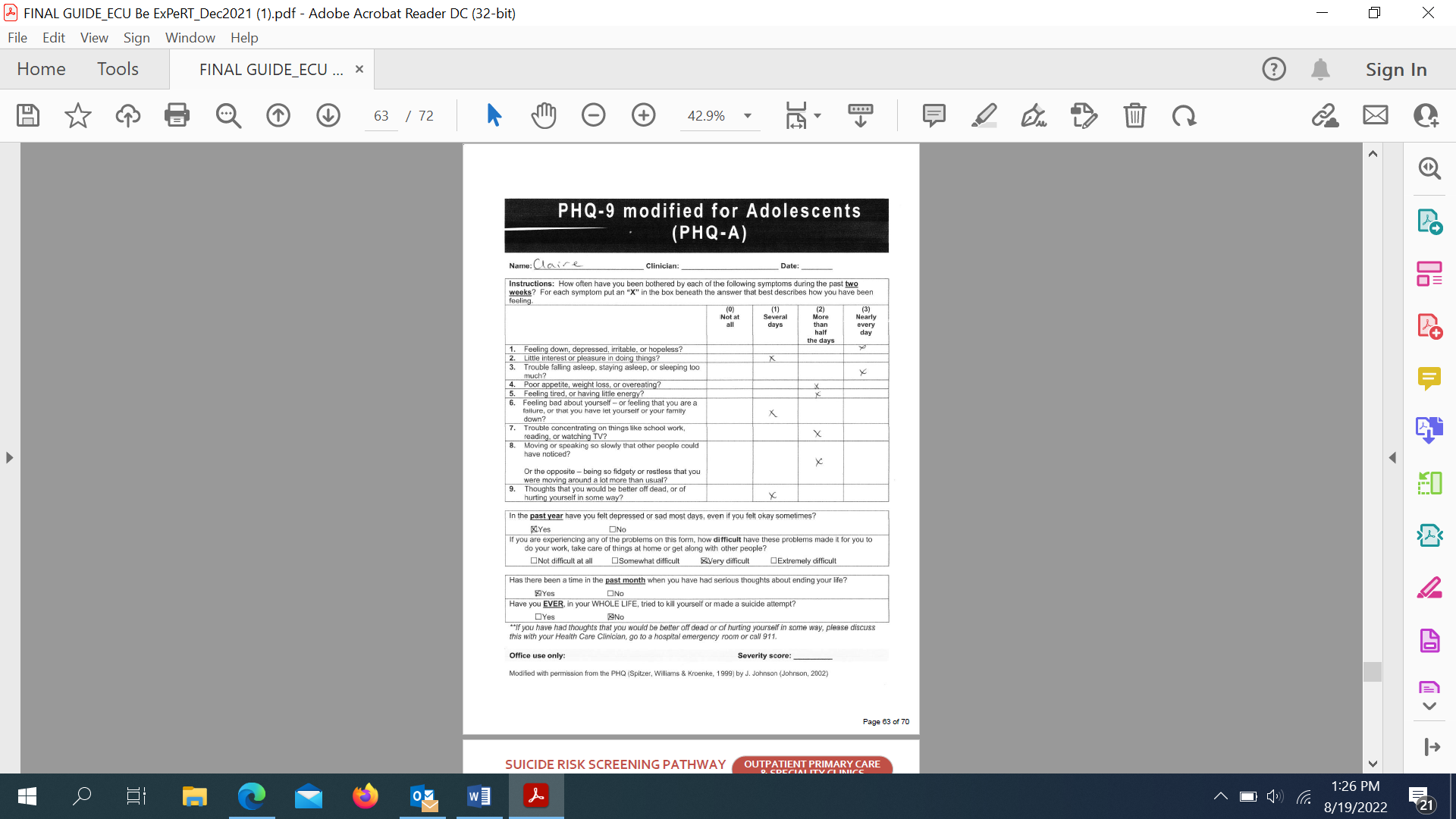


Johnson JG, Harris ES, Spitzer RL, Williams JB. The patient health questionnaire for adolescents: validation of an instrument for the assessment of mental disorders among adolescent primary care patients. J Adolesc Health. 2002 Mar;30(3):196-204.

Image by Johnson et al., retrieved from: https://www.aacap.org/App_Themes/AACAP/docs/member_resources/toolbox_for_clinical_practice_and_outcomes/symptoms/GLAD-PC_PHQ-9.pdf on August 19, 2022. Image is in the public domain.

Modified with permission by the GLAD-PC team from the PHQ-9 (Spitzer, Williams, & Kroenke, 1999), Revised PHQ-A (Johnson, 2002), and the CDS (DISC Development Group, 2000)


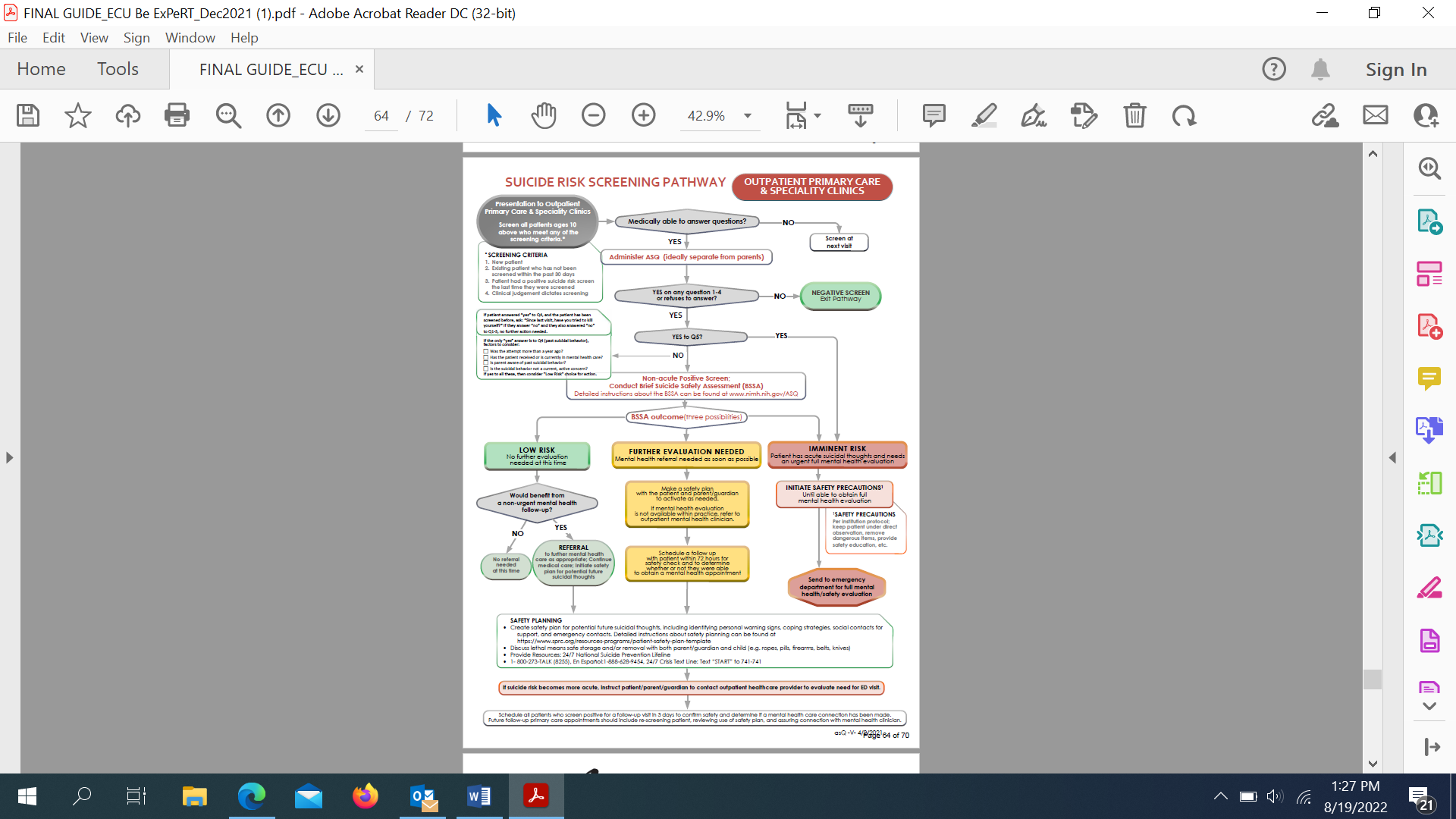


Horowitz LM, Bridge JA, Teach SJ, Ballard E, Klima J, Rosenstein DL, Wharff EA, Ginnis K, Cannon E, Joshi P, Pao M. Ask Suicide-Screening Questions (ASQ): a brief instrument for the pediatric emergency department. *Arch Pediatr Adolesc Med.* 2012 Dec;166(12):1170-6. Image available from NIMH (see references).

Image by NIMH, retrieved from: https://www.nimh.nih.gov/research/research-conducted-at-nimh/asq-toolkit-materials on December 11, 2019. Image is in the public domain.


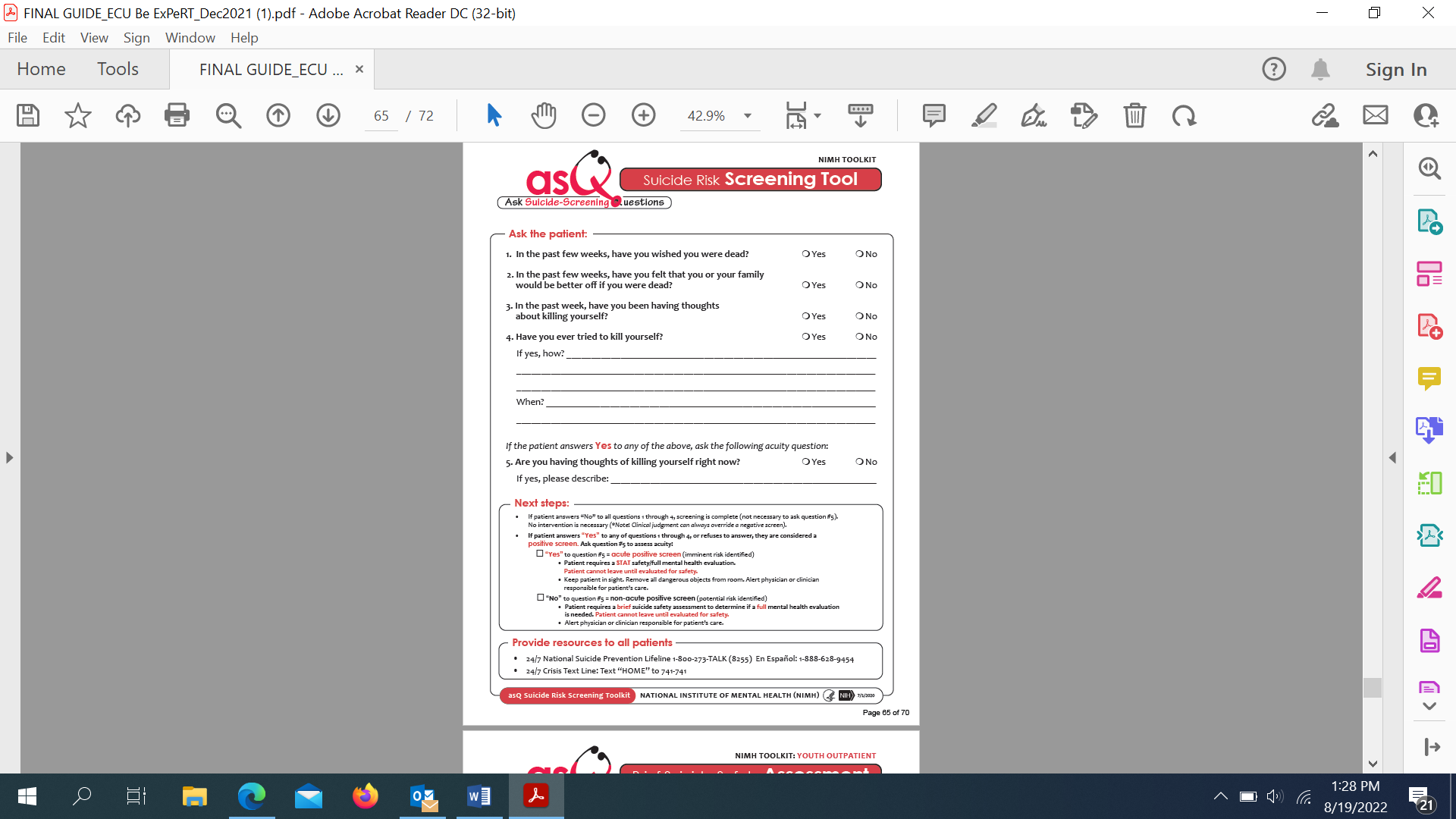


Horowitz LM, Bridge JA, Teach SJ, Ballard E, Klima J, Rosenstein DL, Wharff EA, Ginnis K, Cannon E, Joshi P, Pao M. Ask Suicide-Screening Questions (ASQ): a brief instrument for the pediatric emergency department. *Arch Pediatr Adolesc Med.* 2012 Dec;166(12):1170-6. Image available from NIMH (see references).

Image by NIMH, retrieved from: https://www.nimh.nih.gov/research/research-conducted-at-nimh/asq-toolkit-materials on December 11, 2019. Image is in the public domain.


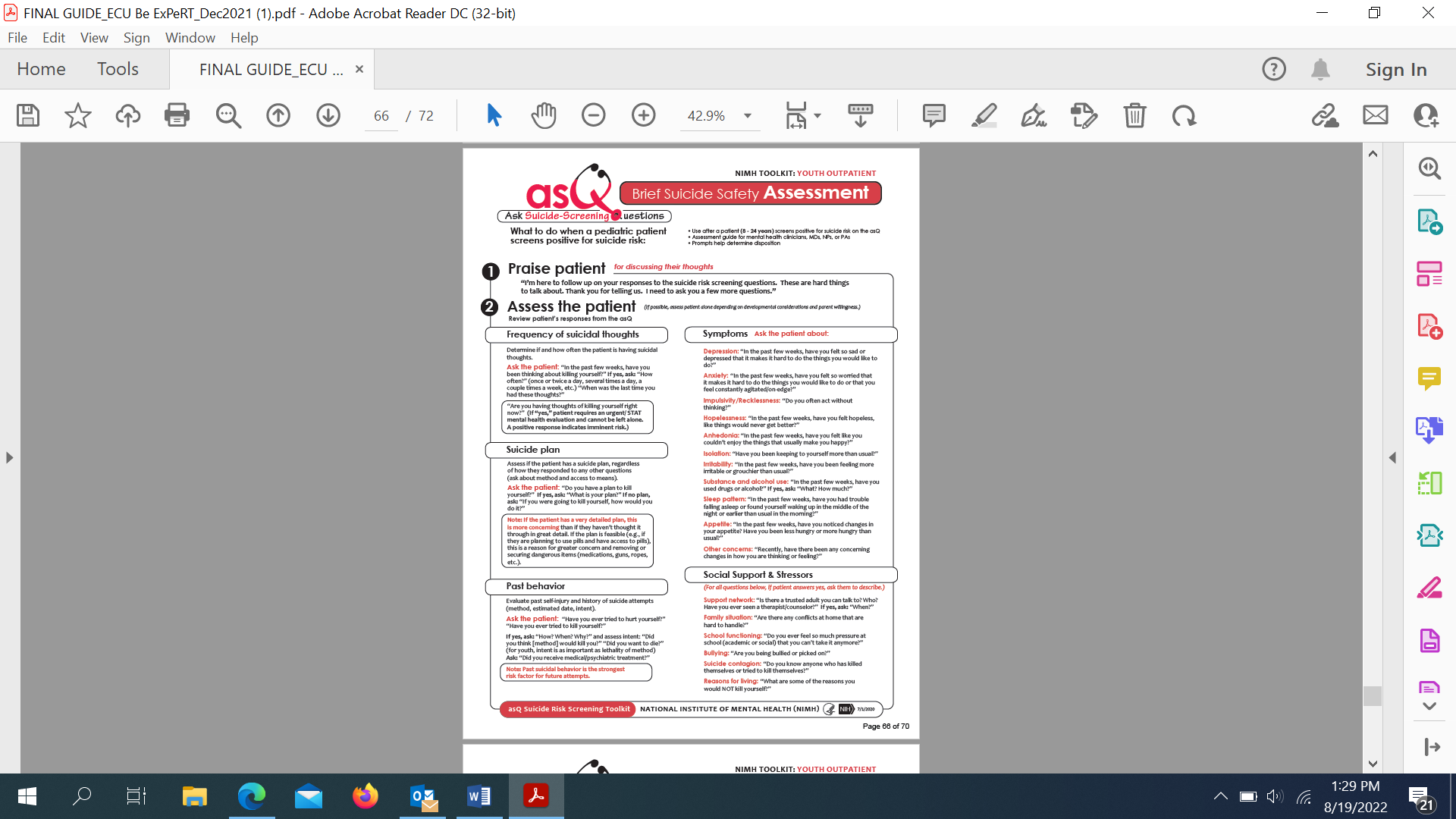


Horowitz LM, Bridge JA, Teach SJ, Ballard E, Klima J, Rosenstein DL, Wharff EA, Ginnis K, Cannon E, Joshi P, Pao M. Ask Suicide-Screening Questions (ASQ): a brief instrument for the pediatric emergency department. *Arch Pediatr Adolesc Med.* 2012 Dec;166(12):1170-6. Image available from NIMH (see references).

Image by NIMH, retrieved from: https://www.nimh.nih.gov/research/research-conducted-at-nimh/asq-toolkit-materials on December 11, 2019. Image is in the public domain.


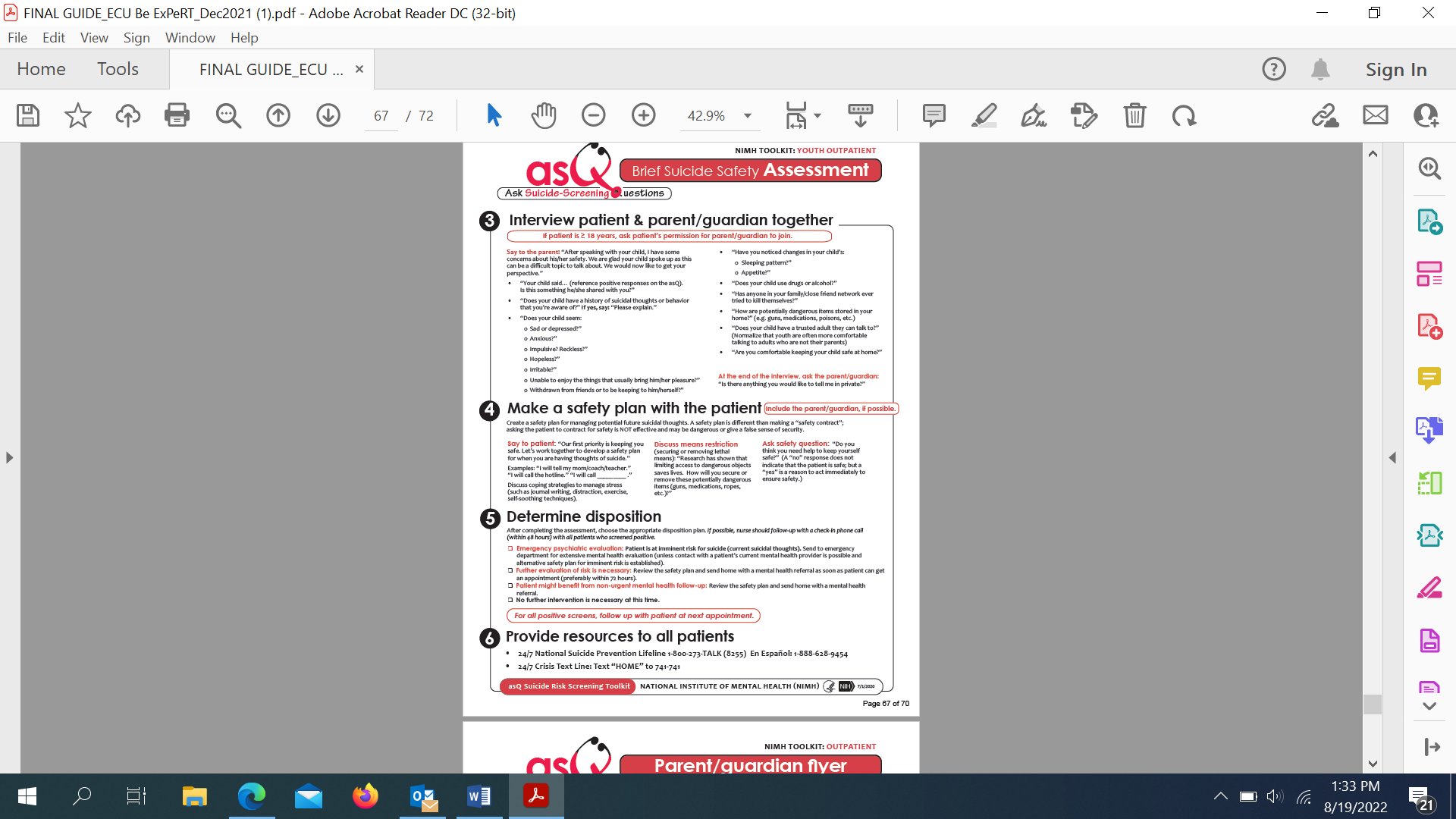


Horowitz LM, Bridge JA, Teach SJ, Ballard E, Klima J, Rosenstein DL, Wharff EA, Ginnis K, Cannon E, Joshi P, Pao M. Ask Suicide-Screening Questions (ASQ): a brief instrument for the pediatric emergency department. *Arch Pediatr Adolesc Med.* 2012 Dec;166(12):1170-6. Image available from NIMH (see references).

Image by NIMH, retrieved from: https://www.nimh.nih.gov/research/research-conducted-at-nimh/asq-toolkit-materials on December 11, 2019. Image is in the public domain.


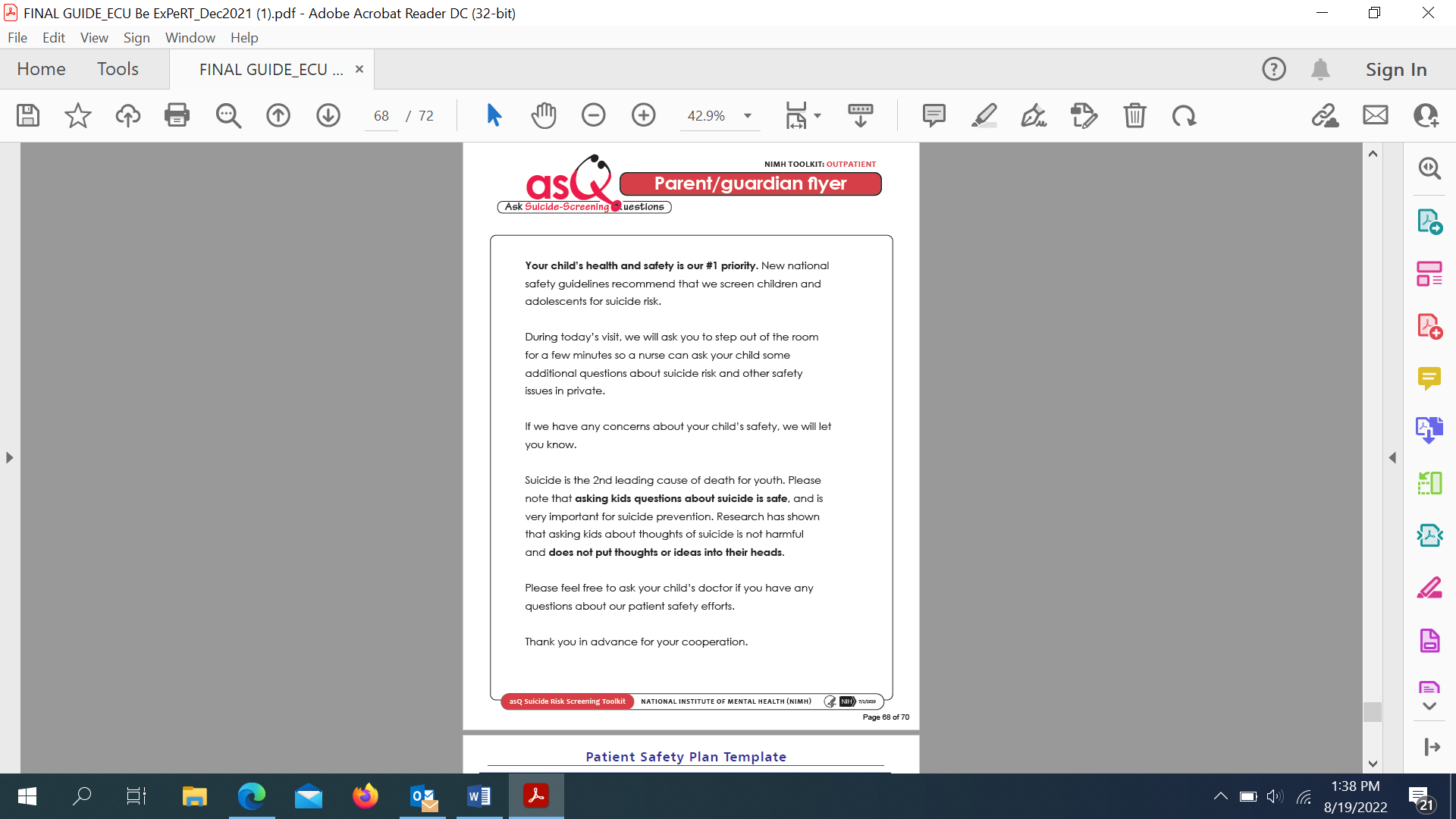


Horowitz LM, Bridge JA, Teach SJ, Ballard E, Klima J, Rosenstein DL, Wharff EA, Ginnis K, Cannon E, Joshi P, Pao M. Ask Suicide-Screening Questions (ASQ): a brief instrument for the pediatric emergency department. *Arch Pediatr Adolesc Med.* 2012 Dec;166(12):1170-6. Image available from NIMH (see references).

Image by NIMH, retrieved from: https://www.nimh.nih.gov/research/research-conducted-at-nimh/asq-toolkit-materials on December 11, 2019. Image is in the public domain.

**My Safety Plan**

Warning signals

How do I know when I am starting to feel very down, suicidal, angry, or distressed? (Do particular thoughts or images come into my mind? Do I feel certain sensations in my body? Does my behavior change? Do certain situations trigger these feelings?)

______________________________________________________________________________________________________________________________________________________________________________________________________________________________________________________________________________________________________

Coping behaviors (e.g., relaxation techniques, physical activities, reading, journaling, art, music, counting to 10, etc.)

What can I do myself to help myself feel better and take my mind off my problems?

1. ______________________________________________________________________________

2. ______________________________________________________________________________

3. ______________________________________________________________________________

4. ______________________________________________________________________________

People and places that help me feel better

1. Name________________________________________________ Phone_________________________

2. Name________________________________________________ Phone_________________________

3. Place__________________________________________________________________________

4. Place__________________________________________________________________________

Adults I can ask for help (family members, school personnel, religious leaders, etc.)

1. Name _______________________________________________ Phone__________________________

2. Name _______________________________________________ Phone__________________________

3. Name _______________________________________________ Phone__________________________

Mental health services I can contact during a crisis (therapist, psychiatrist, etc.)

1. Clinician____________________________________________ Phone___________________________

Pager or Emergency Contact #___________________________________

2. Clinician____________________________________________ Phone___________________________

Pager or Emergency Contact #___________________________________

3. Suicide Prevention Lifeline: 1-800-273-TALK (8255) (Hours 24/7)

4. Suicide Prevention Lifeline Web-Chat (Hours 2 PM to 2 AM)

http://www.suicidepreventionlifeline.org/GetHelp/LifelineChat.aspx

5. Patient’s Insurance Behavioral Health Access Line, if available

6. Emergency Services: call 911 or 988 or go to your nearest emergency room

How can I limit access to things that could harm me?

(Examples: remove guns or other weapons from the home, have medications locked up or managed by adults, etc.)

1. ______________________________________________________

2. ______________________________________________________

Reasons for living: What is important to me and worth living for?

_____________________________________________________________________

Guidance for safety planning:

1. **Warning signs**
   - Ask about what patient experiences when extremely depressed or thinking about suicide; also discuss triggers
2. **Internal coping strategies**
   - Ask what can patient do on own to help not act on thoughts or urges (e.g. go for a walk, draw, watch a favorite TV show)
3. **Social contacts who may distract from the crisis**
   - Identify who or what social settings can help patient take mind off of problems
4. **Family members or friends who may offer help**
   - Identify who patient can contact for support during crisis (at least 1 must be an adult)
5. **Professional and agencies to contact for help**
   - Identify mental health professionals and resources to list on patient’s safety plan (names, numbers, and/or locations, including your office)
6. **Making the environment safe**
   - Ask patient about means that have been considered when experiencing suicidal thoughts and work together **with caregiver** to secure or limit access to lethal means
   - Discuss locking up all medications; discuss safe gun storage

|  | **Generic** | **Trade Name** | **Available Forms** | **FDA Indication (pediatrics)** | **Starting Dose** | **Titration** | **Dose Range** | **Notes** |
| --- | --- | --- | --- | --- | --- | --- | --- | --- |
| **SSRIs** | Fluoxetine | Prozac | Liquid & tablet | MDD 8-17  OCD 7-17 | ≤ 12 5mg  ≥ 12 10mg | 10-20mg | 20-60mg | More med interactions |
|  | Sertraline | Zoloft | Liquid & tablet | OCR 6-17 | ≤ 12 12.5mg  ≥ 12 25mg | 12.5-25mg | 50-200mg |  |
|  | Escitalopram | Lexapro |  | MDD 12-17 | ≤ 12 5mg  ≥ 12 10mg | 5mg | 10-20mg |  |
|  | Citalopram | Celexa |  |  | 10-20mg | 10mg | 20-40mg | QTc prolongation doses >40mg |
| **SNRIs** | Duloxetine | Cymbalta |  | GAD 7-17 | 20-30mg | 20-30mg | 60-120mg | Pain benefit |
|  | Venlafaxine | Effexor | IR & ER |  | ≤ 12 37.5mg  ≥ 12 75mg | 37.5-75mg | 150-300mg | Pain benefit  Monitor BP |


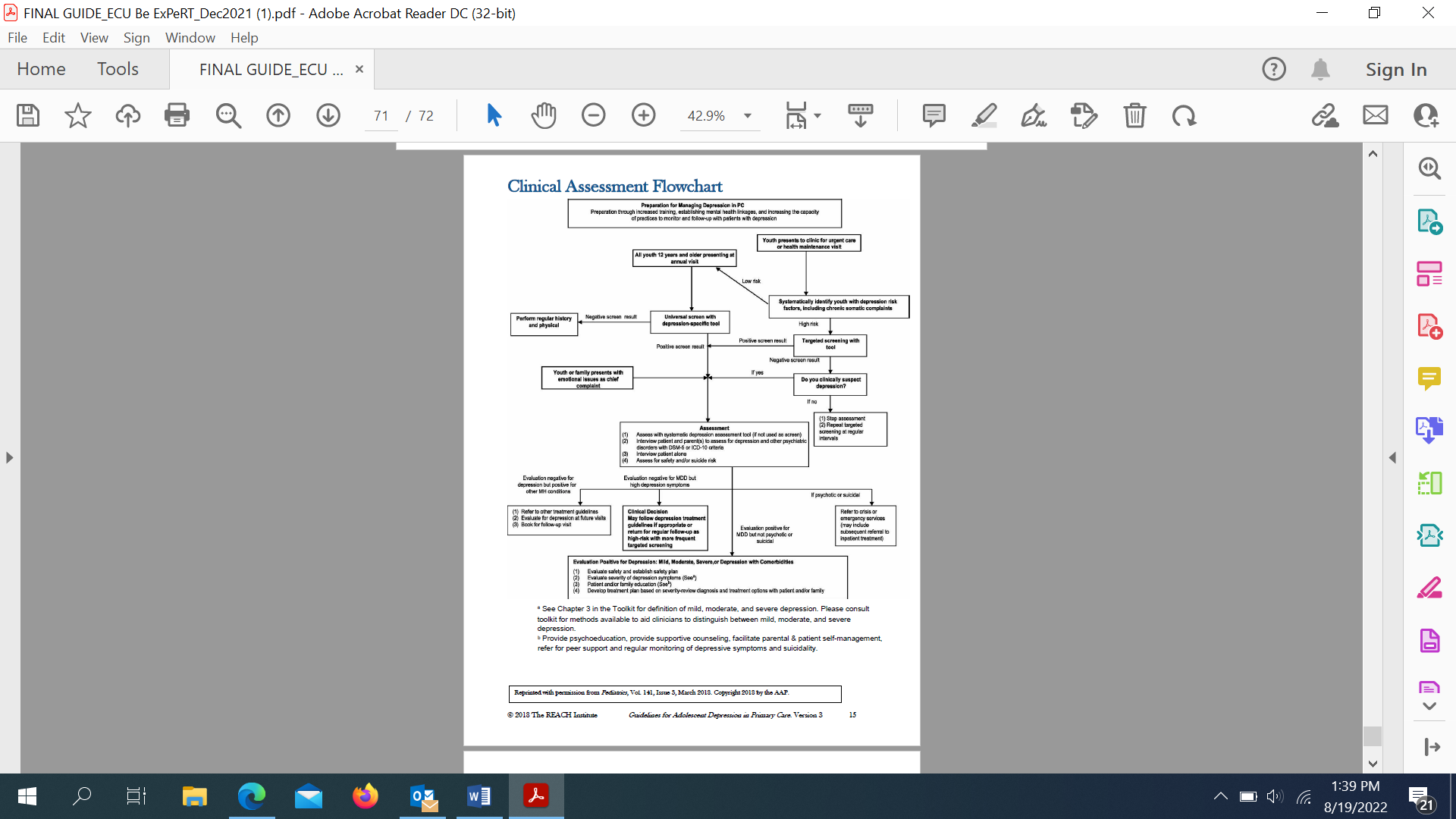


Zuckerbrot RA, Cheung A, Jensen PS, et al. Guidelines for Adolescent Depression in Primary Care **(GLAD-PC): Part I**. Practice Preparation, Identification, Assessment, and Initial Management. *Pediatrics.* 2018;141(3):e20174081.

Image by Zuckerbrot et al, used with permission, on behalf of the GLAD-PC Steering Committee


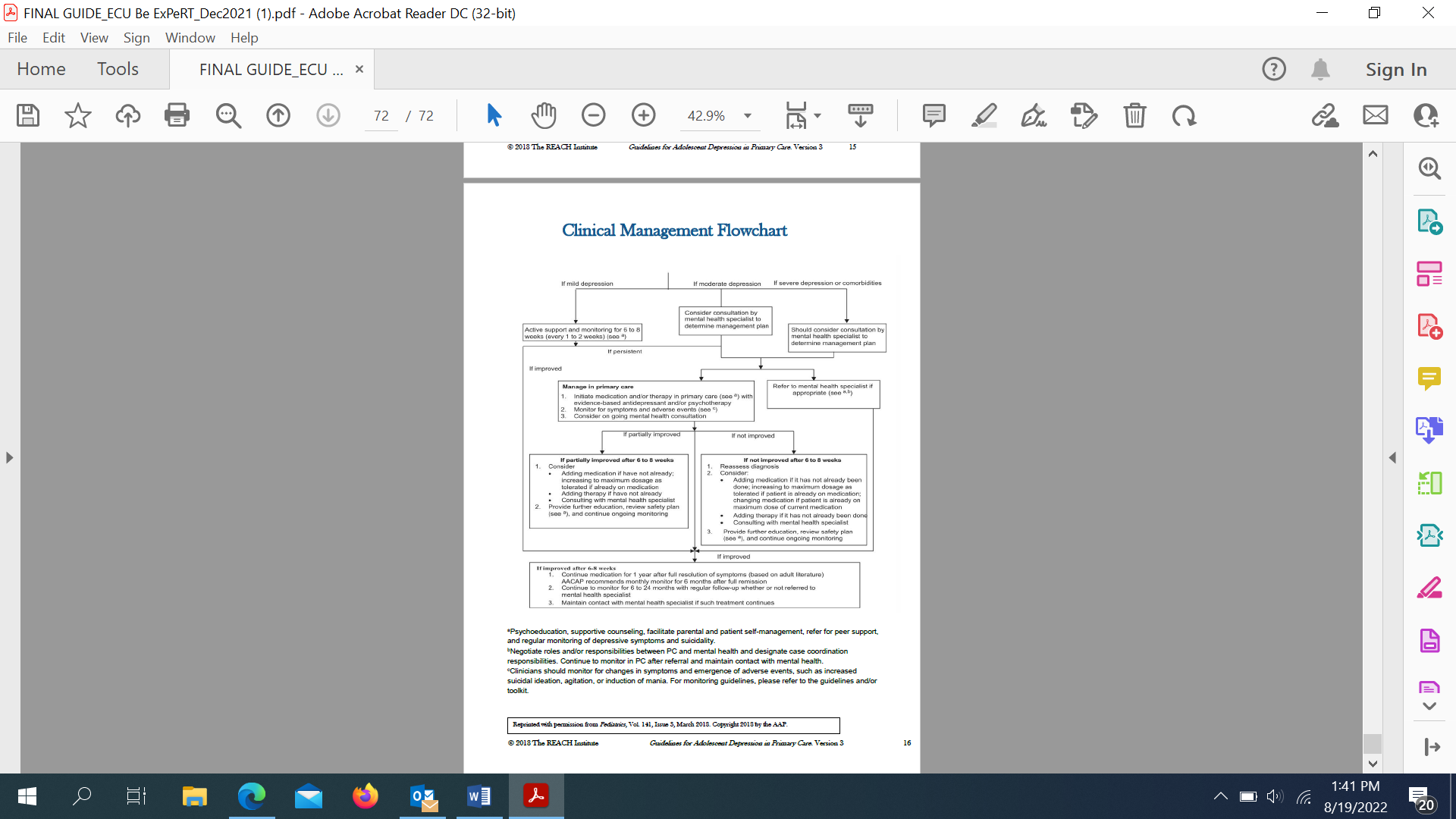


Cheung AH, Zuckerbrot RA, Jensen PS, et al. Guidelines for Adolescent Depression in Primary Care **(GLADPC): Part II**. Treatment and Ongoing Management. *Pediatrics.* 2018;141(3):e20174082.

Image by Zuckerbrot et al, used with permission, on behalf of the GLAD-PC Steering Committee


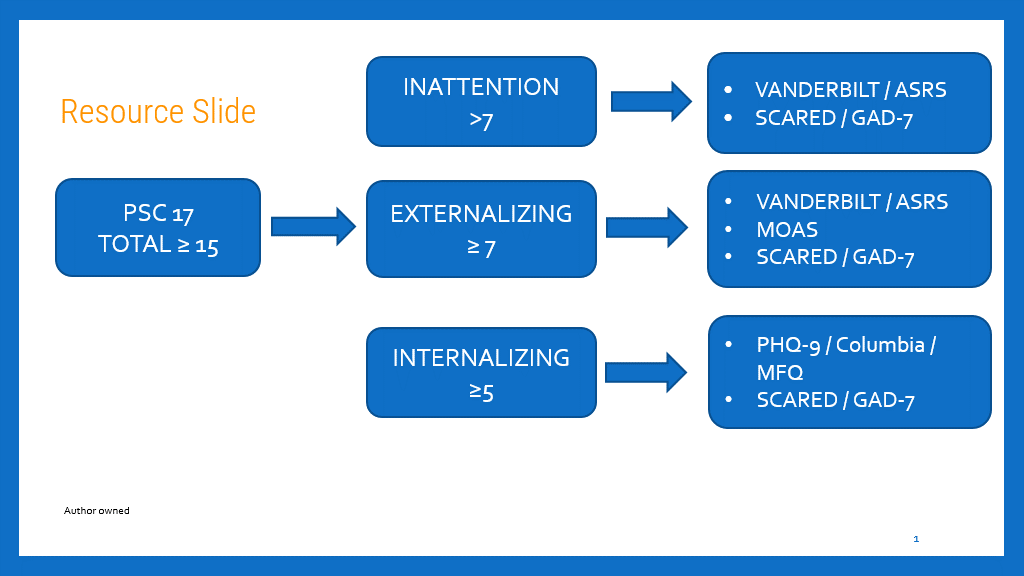


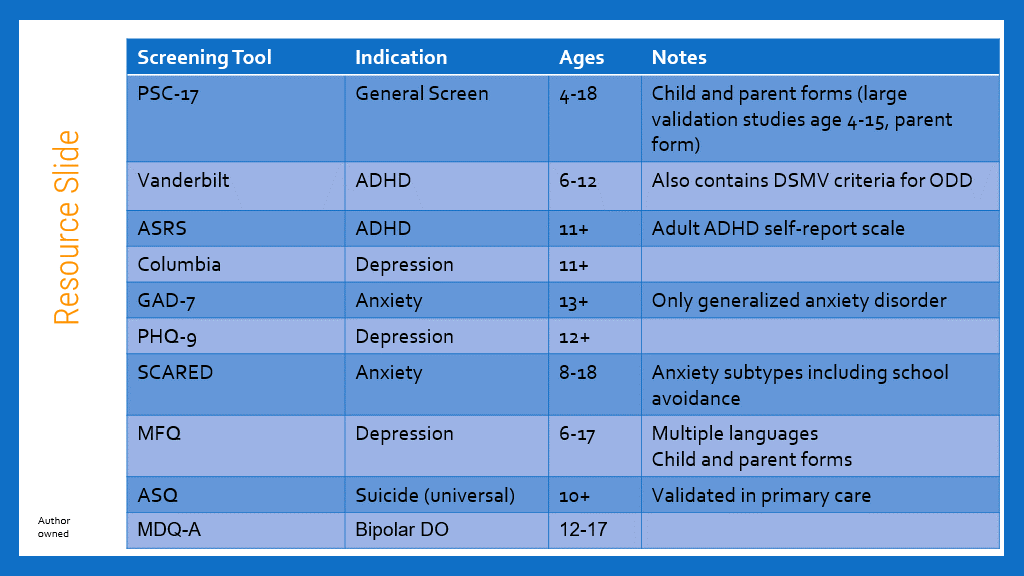

Supplement: Supplementary file 1 — Facilitator Guide.docxBe ExPeRT Introduction.pptxADHD in Primary Care Pediatrics.pptxAnxiety in Primary Care Pediatrics.pptxDepression in Primary Care Pediatrics.pptxBe ExPeRT Reference Slides.pptxParticipant Guide.docxBe ExPeRT Postsurvey.docxBe ExPeRT Case Discussion Form.docxBe ExPeRT Presurvey.docx [file mep_2374-8265.11326-s001.zip › G. Participant Guide.docx]
