# Supplementary material for: Be ExPeRT (Behavioral Health Expansion in Pediatric Residency Training): A Case-Based Seminar
Source: MedEdPORTAL. 2023 Aug 1;19:11326. doi: 10.15766/mep_2374-8265.11326 (PMC10392710; doi:10.15766/mep_2374-8265.11326)
Supplement: Supplementary file 1 — Facilitator Guide.docxBe ExPeRT Introduction.pptxADHD in Primary Care Pediatrics.pptxAnxiety in Primary Care Pediatrics.pptxDepression in Primary Care Pediatrics.pptxBe ExPeRT Reference Slides.pptxParticipant Guide.docxBe ExPeRT Postsurvey.docxBe ExPeRT Case Discussion Form.docxBe ExPeRT Presurvey.docx [file mep_2374-8265.11326-s001.zip › I. Be ExPeRT Case Discussion Form.docx]

**Participant Name**: **Date:**

**I would like help with the following question(s) related to this case:**

**Patient age:**  **Patient Gender: Grade:**

**Case presentation summary:**

- - Chief Complaint:
  - Brief History of Presenting Illness:
  - Pertinent history:
    1. Medical:
    2. Developmental:
    3. Psychiatric:
  - School Performance:
    1. Educational / psychological testing:
  - Family / social history, psychosocial stressors
  - Rating Scales obtained: (include scores and interpretation)
  - Mental Status Exam: (describe how patient appears in the room)
  - Physical findings:
  - Medications and responses/side effects:
    1. Current
    2. Past
  - Differential Diagnoses:
  - Working Diagnosis:

**Summary of Next Steps**: (to be completed after case discussion)
