# Supplementary material for: Be ExPeRT (Behavioral Health Expansion in Pediatric Residency Training): A Case-Based Seminar
Source: MedEdPORTAL. 2023 Aug 1;19:11326. doi: 10.15766/mep_2374-8265.11326 (PMC10392710; doi:10.15766/mep_2374-8265.11326)
Supplement: Supplementary file 1 — Facilitator Guide.docxBe ExPeRT Introduction.pptxADHD in Primary Care Pediatrics.pptxAnxiety in Primary Care Pediatrics.pptxDepression in Primary Care Pediatrics.pptxBe ExPeRT Reference Slides.pptxParticipant Guide.docxBe ExPeRT Postsurvey.docxBe ExPeRT Case Discussion Form.docxBe ExPeRT Presurvey.docx [file mep_2374-8265.11326-s001.zip › J. Be ExPeRT Presurvey.docx]

Be ExPeRT Pre Survey

Q1 Last 4 Digits of Cell Phone Number

________________________________________________________________

Q2 Specialty

- Pediatrics (1)
- Med-Peds (2)
- Other (3) __________________________________________________

Q3 Year in training (PGY)

________________________________________________________________

Q5 I am **knowledgeable** about *assessing* and *diagnosing* the following disorder or symptom

|  | Not at All (1) | Small Amount (2) | Moderate Amount (3) | Great Deal (4) |
| --- | --- | --- | --- | --- |
| Major Depressive Disorder (1) |  |  |  |  |
| Suicide Risk (2) |  |  |  |  |
| Anxiety Disorder (3) |  |  |  |  |
| Attention Deficit Hyperactivity Disorder (4) |  |  |  |  |

Q6 I am **comfortable** with *assessing* and *diagnosing* the following disorder or symptom

|  | Not at All (1) | Small Amount (2) | Moderate Amount (3) | Great Deal (4) |
| --- | --- | --- | --- | --- |
| Major Depressive Disorder (1) |  |  |  |  |
| Suicide Risk (2) |  |  |  |  |
| Anxiety Disorder (3) |  |  |  |  |
| Attention Deficit Hyperactivity Disorder (4) |  |  |  |  |

Q7 I am **knowledgeable** about *treating* the following disorder or symptom

|  | Not at All (1) | Small Amount (2) | Moderate Amount (3) | Great Deal (4) |
| --- | --- | --- | --- | --- |
| Major Depressive Disorder (1) |  |  |  |  |
| Suicide Risk (2) |  |  |  |  |
| Anxiety Disorder (3) |  |  |  |  |
| Attention Deficit Hyperactivity Disorder (4) |  |  |  |  |
| Aggression/Agitation (5) |  |  |  |  |

Q8 I am **comfortable** with *treating* the following disorder or symptom

|  | Not at All (1) | Small Amount (2) | Moderate Amount (3) | Great Deal (4) |
| --- | --- | --- | --- | --- |
| Major Depressive Disorder (1) |  |  |  |  |
| Suicide Risk (2) |  |  |  |  |
| Anxiety Disorder (3) |  |  |  |  |
| Attention Deficit Hyperactivity Disorder (4) |  |  |  |  |
| Aggression/Agitation (5) |  |  |  |  |

Q9 Please check the statement which best reflects how you currently approach the assessment and diagnosis of children and adolescents.

|  | Assess and diagnose all cases (1) | Assess and diagnose most cases (2) | Assess and diagnose only less complicated cases (3) | Refer most cases if diagnosis is suspected (4) | Refer all suspected cases without assessing and diagnosing (5) |
| --- | --- | --- | --- | --- | --- |
| Major Depressive Disorder (1) |  |  |  |  |  |
| Suicide Risk (2) |  |  |  |  |  |
| Anxiety Disorder (3) |  |  |  |  |  |
| Attention Deficit Hyperactivity Disorder (4) |  |  |  |  |  |

Q11 Please check the statement which best reflects how you currently approach the treatment and management of children's and adolescents' behavioral and emotional difficulties.

|  | Manage all cases with no consultation/ collaboration with a mental health colleague (1) | Manage most cases with occasional consultation with a mental health colleague (2) | Manage only the less complicated cases (3) | Refer most cases if diagnosis is suspected (4) | Refer all suspected cases without assessing and diagnosing (5) | Co-manage (e.g. refill psychotropic meds) if child also being seen regularly by a child/adolescent psychiatrist (6) |
| --- | --- | --- | --- | --- | --- | --- |
| Major Depressive Disorder (1) |  |  |  |  |  |  |
| Suicide Risk (2) |  |  |  |  |  |  |
| Anxiety Disorder (3) |  |  |  |  |  |  |
| Attention Deficit Hyperactivity Disorder (4) |  |  |  |  |  |  |
| Aggression/Agitation (5) |  |  |  |  |  |  |

Q12 What psychotropic medications do you feel competent prescribing? Please check all that apply.

- Stimulant Medication (e.g. methylphenidate) (1)
- Selective Serotonin Reuptake Inhibitors (e.g. Fluoxetine) (2)
- Atypical Antipsychotics (e.g. Risperidone) (3)

Q13 Please check all that apply.

|  | Rarely (1) | For diagnosis and assessment some of the time (2) | For diagnosis and assessment most of the time (3) | As part of treatment monitoring some of the time (4) | As part of treatment monitoring most of the time (5) | Yes, my practice does this automatically (6) |
| --- | --- | --- | --- | --- | --- | --- |
| I use a general psychosocial screen (1) |  |  |  |  |  |  |
| For ADHD, I use rating scales (2) |  |  |  |  |  |  |
| For anxiety symptoms, I use rating scales (3) |  |  |  |  |  |  |
| For depressive symptoms, I use rating scales (4) |  |  |  |  |  |  |
| For suicidal ideation, I use rating scales (5) |  |  |  |  |  |  |
