# Supplementary material for: Be ExPeRT (Behavioral Health Expansion in Pediatric Residency Training): A Case-Based Seminar
Source: MedEdPORTAL. 2023 Aug 1;19:11326. doi: 10.15766/mep_2374-8265.11326 (PMC10392710; doi:10.15766/mep_2374-8265.11326)
Supplement: Supplementary file 1 — Facilitator Guide.docxBe ExPeRT Introduction.pptxADHD in Primary Care Pediatrics.pptxAnxiety in Primary Care Pediatrics.pptxDepression in Primary Care Pediatrics.pptxBe ExPeRT Reference Slides.pptxParticipant Guide.docxBe ExPeRT Postsurvey.docxBe ExPeRT Case Discussion Form.docxBe ExPeRT Presurvey.docx [file mep_2374-8265.11326-s001.zip › A. Facilitator Guide.docx]

**MedEdPORTAL**

**Appendix A: Facilitators’ Guide**

This training program is intended to serve as a stand-alone package. The package includes:

1. Facilitators’ Guide (Appendix A)
2. The training itself (Introductory slides, PowerPoint presentations with slide notes, and reference slides)
   1. Introduction ppt (Appendix B)
   2. ADHD ppt (Appendix C)
   3. Anxiety ppt (Appendix D)
   4. Depression ppt (Appendix E)
   5. References ppt (Appendix F)
3. Participant Resource Guide (Appendix G)
4. Pre- and postsurveys (Appendices H and J)
5. Case discussion worksheet (Appendix I)

**Intended Audience**: this training was originally developed for pediatric and medicine-pediatric residents in the United States. Adapted versions of the training have also been given to family medicine residents and physician assistant students. This training would be appropriate for trainees of varying disciplines as well as practicing pediatric primary care providers.

**Faculty Tips**: The training may be given by pediatric or child psychiatry faculty or pediatric or child psychiatry trainees. Faculty should familiarize themselves with the educational objectives, content, and cases in the PowerPoint presentations, as well as the goals for the discussion groups (see below). Role plays should be practiced prior to the presentation to ensure goals are being met. The recommended amount of preparation time is intended to be 1-2 hours per presentation. The training is meant to be given over a 3-hour block of time. However, it was created to be able to be given in separate 1-hour modules, for example as a noon conference series. Additional faculty may be needed depending on the number of learners, as it is important to have a facilitator in each discussion group. Discussion groups should not contain more than 10 learners. Local mental health resources are included in some presentations and should be modified to reflect resources specific to the institution delivering the training.

**Pre- post- survey**: The survey is meant to be given before and after the presentations to assess for improved knowledge. The assessment tool can also be divided by subject in the event that the content is divided into 3 separate modules. We recommend using on online platform such as SurveyMonkey or Qualtrics to deliver the survey. We recommend allowing time during class for participants to complete the post-survey.

**Unit 1: ADHD**

Facilitated Case Discussion in Small Groups (25-minutes): *Johnny is an 8-year-old boy with no past psychiatric history and no past medical history who presents with his caregivers for “difficulties at school”.*

**Goal**:  *discuss helpful clinical assessment questions to ask caregivers, practice scoring a Vanderbilt, discuss treatment with stimulants.*

Facilitate a discussion with your group to answer the questions in the first column. Possible answers are provided in second column.

| 1) What would you ask parents to clarify diagnosis? | Does Johnny have trouble sitting still? Playing quietly? Completing homework? Getting himself dressed? Do you have to repeat instructions several times? If you ask him to do 3 things does he come back after only completing the first? Make careless mistakes on written work? |
| --- | --- |
| 2) Score Johnny's Vanderbilt | 8 for inattention, 7 for hyperactivity. Interpretation is he has ADHD, combined type. Not ODD. Functional status is 4.125. He is impaired. Teacher score is 4 for inattention and 6 for hyperactivity: ADHD, hyperactive type. Functional status performance score is 4.625. Symptoms present in 2 settings, meets criteria for ADHD, hyperactive type. |
| 3) Before you start the treatment, what medical screening would you do? | Vitals, family history and personal history of cardiac disease, screen for other mental health conditions including tics. NO NEED for screening ECG unless indicated based on personal or family cardiac history |
| 4) What side effects would you counsel about? | GI distress, poor appetite, weight loss, insomnia, palpitations, headaches, anxiety/agitation, tics, irritability/psychiatric symptoms, increased blood pressure |
| 5) You started the patient on a short acting stimulant. He is now doing great staying focused in the morning, but is distracted in the afternoon and is having trouble completing chores at home in the evening. What would you do next? | Methylphenidate or amphetamine. Consider age of child. Consider parent preference. Consider benefits of long-acting vs short-acting.  Increase dose or switch to long-acting formulation. |
| 6) The parents have questions about stopping the medication during the weekends | Difficulty with weight gain or other side effects may provide support for med holidays but generally important to continue to treat given risk for motor vehicle accidents and psychosocial effects/development of behavioral health co-mordbidities |

**Unit 2: Anxiety**

1. Faculty Role Play Demonstration in Large Group (10-minute role play, 3-minute discussion): Faculty members act as Leila, caregiver, PCP.

*Leila is a 10-year-old girl who presents at the recommendation of her school for daily belly aches causing her to miss a significant amount of school.*

**Goal**: It should be clear that Leila has an anxiety disorder with significant functional impairment. Learners should consider treatment with both therapy and medication.

**PCP:** *Medical work up and exam are normal. No further medical work up is indicated. Model explanation of somatic symptoms, psychoeducation regarding anxiety, and discussion of treatment (therapy and SSRI).*

**Leila**: *You worry about your parents’ safety since mom was in a car accident 3 years ago. You call mom repeatedly when she is not at home to make sure she is safe. You also really worry about doing well in school; you don’t speak up in class because of what other kids will think of your or if you get the answer wrong. You can't stop biting you nails which bothers mom. You almost threw up before a test last week because you felt so scared. You have trouble falling asleep at night for months, you worry about someone breaking into your home. It’s hard to concentrate on homework or chores and your parents have to remind you several times. You feel guilty because mom has to go to a meeting with your teacher and you worry about this impact on her baby brother. Your head and your belly hurt worse in the mornings and Sunday night, but not on weekends.*

**Caregiver**: *You are concerned about a more serious problem with Leila’s stomach and would like more tests done. Your father was diagnosed with colon cancer last year. Leila typically gets good grades in school although they have declined slightly this year which is concerning to you. Leila has always been a “sensitive” and “shy” child. You’d rather Leila stay home than risk vomiting at school but can’t continue to miss work. You are not aware of any bullying. Leila has been complaining of stomach and head pain since school started and you really want to figure out what is going on. Leila had a baby brother born last summer and you had post-partum anxiety at that time which resolved without treatment.*

1. Participant Role Play and Discussion in Small Groups (15-minutes):

Faculty member acts as Leila’s anxious parent who is skeptical of anxiety treatment. Learner acts as PCP. Faculty should select a learner to be the PCP if none volunteers.

PCP should address the following:

| Describe the diagnosis and your recommended treatment plan | Anxiety disorder vs. school avoidance with functional symptoms/somatization. Therapy and SSRI |
| --- | --- |
| Provide psychoeducation about anxiety to Leila and her mom | Anxiety frequently presents with somatization in children. We’ve ruled out serious structural etiologies. Anxiety is common and treatable. |
| During debrief, discuss ways to engage a reluctant parent | Assess barriers, preconceptions, ask permission to continue to discuss medications |
| What medication would you start? How much? For how long? | SSRI, goal to get to lowest therapeutic dose, anxiety may need higher doses, average dose sertraline from CAMS was in the 120 mg range |
| When would you follow up? | 2-4 weeks, monthly until dose effective, in remission, connected to therapy |

**Unit 3: Depression**

1. Faculty Safety Planning Role Play in Large Group (7-minutes): Faculty members act as Claire, Claire’s parent and PCP.

**Goal:** Model completing a safety plan with a patient, discuss breaking confidentiality, discuss means restriction. The PCP has already established that the patient has SI, but no plan or intent and no prior history of attempts, and so is appropriate for safety planning. The PCP should convey this to the audience at the start of the role play.

**Claire**: *You notice that you start to become suicidal when you feel that you’ve made a mistake or are embarrassed in a social situation. You also notice that when you cannot stop crying you feel suicidal and hopeless. Listening to music or playing your ukulele can help you feel better. You also feel better when you are at swim practice or at the pool. You have a best friend whom you tell everything. You are close with your swim coach and science teacher. You want to be a doctor when you grow up. You don’t think you would actually ever kill yourself because your mom would be devastated. You are hoping that your swim team makes it to finals this year but when you’re really depressed none of that seems to matter.*

**Claire’s parent**: *You have a safe in which you can store medications. You can administer medications for Claire. Claire’s father agrees that she can stay at your house until she is feeling better as the moving from house to house in the context of your separation has been stressful for Claire.*

**PCP:**

- Engage Claire in a discussion about warning signs, coping strategies, people and settings that provide distraction and people she can call for help per the Stanley-Brown Safety Plan template.
- Inform Claire that you will have to break confidentiality and disclose safety concerns to her mom and ask if she would feel more comfortable telling her mother or if she would prefer that you do the talking.
- Engage mom in a discussion about means restriction and making the environment safe
- End with scheduling follow up in 2 weeks

1. Safety Planning and Discussion of Boxed Warning in Small Groups (25-minutes):

Select learners to role play as the PCP and Claire.

Learners should address the following in the role play:

- Practice asking directly about suicide, can use the ASQ (10 min)
- Practice creating a safety plan with Claire and her mother
- Practice discussing a treatment plan including discussing the Boxed Warning (10 min)
- Discuss means restriction, if time

Faculty member leads debrief (5 min)
