# Supplementary material for: Be ExPeRT (Behavioral Health Expansion in Pediatric Residency Training): A Case-Based Seminar
Source: MedEdPORTAL. 2023 Aug 1;19:11326. doi: 10.15766/mep_2374-8265.11326 (PMC10392710; doi:10.15766/mep_2374-8265.11326)
Supplement: Supplementary file 1 — Facilitator Guide.docxBe ExPeRT Introduction.pptxADHD in Primary Care Pediatrics.pptxAnxiety in Primary Care Pediatrics.pptxDepression in Primary Care Pediatrics.pptxBe ExPeRT Reference Slides.pptxParticipant Guide.docxBe ExPeRT Postsurvey.docxBe ExPeRT Case Discussion Form.docxBe ExPeRT Presurvey.docx [file mep_2374-8265.11326-s001.zip › Educational Summary Report_11326.pdf]

MedEdPORTAL® The AAMC Journal of Teaching and Learning Resources

Original Publication

Open Access

Be ExPeRT (Behavioral Health Expansion in Pediatric Residency Training): A Case-Based Seminar

Alison Manning, MD*, Matthew Weingard, MD, Jacqueline Fabricius, MD, Alexis French, PhD, Mya Sendak, MD, Naomi Davis, PhD

*Corresponding author: amanning1@lifespan.org

Abstract

Introduction: Pediatric residents report behavioral or mental health (B/MH) assessment and treatment as a training gap and often feel ill-equipped to address these issues in clinical practice. We developed a novel interactive training program to improve resident confidence in managing common pediatric B/MH conditions. **Methods:** The Be ExPeRT curriculum comprised a half-day interactive seminar on attention deficit hyperactivity disorder, anxiety, depression, and suicidality followed by monthly case-based discussions. Content included didactic material, role-play, and case discussion. The training was optional and open to pediatric or combined medicine-pediatrics trainees. **Results:** Twenty-three residents (70% female) participated in four separate seminars over 2 years. Of the participants attending the seminars, 17 (74%) completed the presurvey, and 16 (70%) completed the postsurvey. Statistically significant improvement was noted in comfort treating major depressive disorder (41% pre, 94% post, $p = .002$), suicide risk (29% pre, 94% post, $p < .001$), and anxiety (24% pre, 94% post, $p < .001$) following program participation. Twelve (75%) of the 16 participants completing the survey rated the training in the top 5%-10% with respect to other resident learning experiences. **Discussion:** We developed this curriculum to enhance trainee knowledge and comfort in addressing common pediatric B/MH conditions in primary care. Significant improvement was noted in self-reported comfort in treating major depressive disorder, suicide risk, and anxiety, and the program was well received. The curriculum can be adapted for use in any training program for primary care providers to provide B/MH education that may be lacking or supplement existing programming.

Keywords:
Case-Based Learning, Pediatrics, Primary Care, Psychiatry, Well-Being/Mental Health, Integrated Behavioral Health

Educational Objectives

By the end of this activity, participants will be able to:

1. Utilize evidence-based rating scales to guide diagnosis, assessment, and treatment of child and adolescent attention deficit hyperactivity disorder (ADHD), anxiety, and depression.
2. Describe evidence-based treatment recommendations for children and adolescents with ADHD, anxiety, and depression.
3. Screen for and assess suicide risk and initiate safety planning with pediatric patients and their families.

Citation:
Manning A, Weingard M, Fabricius J, French A, Sendak M, Davis N. Be ExPeRT (Behavioral Health Expansion in Pediatric Residency Training): a case-based seminar. *MedEdPORTAL*. 2023;19:11326.
https://doi.org/10.15766/mep_2374-8265.11326

Introduction

Almost 20% of children have a behavioral or mental health (B/MH) condition, and half receive no treatment.[1] As many as 70% of primary care visits involve a B/MH complaint, and primary care providers (PCPs) report feeling unprepared to address these issues.[2] The scarcity of B/MH specialists has hindered children and families in accessing treatment despite the increasing demand for it.[3] In fact, only half of referrals from PCPs successfully connect children to B/MH specialists, highlighting the need to at least begin B/MH assessment and treatment in primary care.[4,5]

Given the growing number of children with B/MH needs and the shortage of child and adolescent psychiatrists (CAPs), there is a mounting need for PCPs to manage B/MH issues.[4] Unfortunately, this need has been exponentially compounded by the COVID-19 pandemic, as evidenced by the American Academy of Pediatrics declaring a national emergency regarding children's B/MH.[3] Most graduating pediatric trainees report a lack of skills to treat B/MH

Copyright © 2023 Manning et al. This is an open-access publication distributed under the terms of the Creative Commons Attribution license.

conditions, and pediatric program directors consistently identify B/MH as a training gap.[4,5] Several organizations have recognized this essential need and formally called for intervention. The American Academy of Pediatrics published a policy statement in 2009 that set forth B/MH competencies for primary care pediatricians along with recommendations for achieving them.[6] The policy was updated in 2019 to incorporate new data on social determinants of health, trauma, and B/MH management in subspecialty settings.[7] The American Board of Pediatrics also called for improved B/MH education and labeled the training gap as a crisis in 2017.[8] Despite a nationally recognized, urgent need for enhanced B/MH education, widespread change has not been implemented, and the current literature lacks consensus on best practices for doing so.[8-10]

While the importance of suicide screening is well established, few resources exist to help trainees develop knowledge and skills related to suicide risk assessment and safety planning in primary care.[11,12] As of April 2022, there were three *MedEdPORTAL* resources on the implementation of suicide assessment during medical training. One is a team-based learning module for medical students on their psychiatry clerkship rotation.[13] Another is a web-based module designed for practicing PCPs and conducted with nurse practitioner and nurse midwife students.[14] The third resource directly targets residents; however, it was published in 2016 and is now outdated in relation to epidemiological data and validated screening tools.[15] With regard to other common pediatric B/MH conditions, Mian and colleagues described a video-based training for pediatric residents to improve identification and treatment of anxiety disorders in primary care settings.[16] There are also several *MedEdPORTAL* publications on trauma-informed care directed towards pediatric primary care health professionals as well as a resident curriculum on primary care of gender and sexual minority patients.[17-19] While these are all highly valuable, none deliver comprehensive content to enhance overall pediatric B/MH competencies and prepare graduating trainees to meet the needs of their patients. Our aim was to develop and pilot an evidenced-based training curriculum for residents to enhance their confidence and knowledge in addressing the most common pediatric M/BH concerns (attention deficit hyperactivity disorder [ADHD], anxiety, depression, and suicidality) that could be expanded to other programs that train PCPs.[20,21]

The purpose of this educational intervention is to provide a standardized, evidence-based curriculum built on the principles of adult learning theory that addresses the current educational gap in pediatric resident B/MH training. The target audience is pediatric trainees, including categorical trainees as well as medicine-pediatric combined trainees and pediatric-neurology combined trainees. This curriculum could be expanded to other educational programs with graduating PCPs such as nurse practitioner or physician assistant training programs.

Methods

Curriculum Development

The Be ExPeRT (Behavioral Health Expansion in Pediatric Residency Training) program comprised a half-day interactive seminar followed by monthly case-based discussions. The approach was based on Kolb's learning theory of adult pedagogy and emphasized interactive education through experiential learning, reflection, and active application of new material.[22] The monthly case discussions were informed by the Project Extension for Community Healthcare Outcomes method for enhancing professional learning and practice change.[23] We conducted a survey-based needs assessment with participants prior to the development of the curriculum to help inform its content. The seminar didactic materials (PowerPoint presentations, cases, role-plays, and reference guide) were developed by child psychiatry fellows in coordination with a faculty member trained in pediatrics and child and adolescent psychiatry. Cases and role-plays were created to represent typical cases seen in primary care. The seminar was facilitated by child and adolescent psychiatry fellows and faculty. We invited all pediatric residents who had previously completed the course to become cofacilitators for subsequent trainings. We encouraged all facilitators to review the facilitator guide (Appendix A), PowerPoint presentations including references (Appendices B-F), cases, and role-play descriptions prior to program implementation. We held 1-2 hours of preparation sessions with facilitators to review and practice presentations and role-plays. Faculty alternated facilitating the monthly discussion sessions.

Interactive Seminar

The interactive seminar was intended to take 3 hours and covered the topics of ADHD, anxiety, and depression with suicide and safety assessment. At the start of the seminar, a faculty member presented the introductory materials, gave an overview of the course, and reviewed the main learning objectives (Appendix B). We distributed the participant guide (Appendix G), which included validated screening tools, treatment algorithms, dosing guidelines, and nonpharmacologic resources. Residents were encouraged to refer to the guide during the seminar to support active learning. It is recommended that other groups delivering the training modify the slides and participant guide to include resources specific to their location/institution.

Copyright © 2023 Manning et al. This is an open-access publication distributed under the terms of the Creative Commons Attribution license.

For each activity in the seminar, the larger group was divided into three smaller groups, with a faculty facilitator in each group.

We began the first topic with a brief PowerPoint presentation outlining the evidence for assessment and treatment of pediatric ADHD, including dispelling common myths about diagnosis and treatment (Appendix C). This was followed by a discussion in small groups where participants applied what they had learned in the lecture to a case. During this small-group activity, participants practiced scoring Vanderbilt ADHD Screening Forms[24] and discussed assessment and treatment of ADHD in a child with school difficulties, including how to engage caregivers in a discussion of the diagnosis and treatment options.

The second hour began with a brief PowerPoint presentation (Appendix D) outlining the evidence for assessment and treatment of pediatric anxiety disorders, including when to treat with therapy, medications, or both. This was followed by a role-play by faculty members to demonstrate a clinical scenario involving a PCP, caregiver, and child presenting with anxiety, functional abdominal pain, and school avoidance. The second part of the hour involved small groups in which learners acted as the PCP and caregiver and engaged in a discussion about therapeutic and pharmacologic management of anxiety.

The last session taught the principles of assessment and treatment of pediatric depression, with an emphasis on suicide assessment and safety planning (Appendix E). This was followed by a faculty role-play of an adolescent with depression and suicidal thoughts who presented to their PCP with their caregiver. Faculty first demonstrated completing a suicide assessment and safety plan with the patient and caregiver, and then, learners paired up in small groups to role-play suicide assessment and safety planning.

Ten minutes were reserved at the end of the 3-hour seminar for learners to complete the postseminar survey (Appendix H) and sign up to present at a monthly case discussion conference.

Case Discussions

Following the interactive seminar, resident-led case discussion sessions occurred monthly and lasted 45 minutes during protected time for residents, typically at a noon conference. As noted above, upon completion of the initial seminar, residents signed up to present a case at a subsequent monthly case discussion conference. At least one faculty member was present at each monthly case conference to facilitate the discussion and answer questions. We encouraged residents to select a deidentified case from their current caseload that would be relevant to topics presented in the interactive seminar. Prior to each discussion, the presenting resident completed a case discussion worksheet (Appendix I) outlining the basic history of present illness, treatment course, current treatment, and any questions they wanted to discuss about the case. They emailed this form to the learners and facilitators. During the case discussion, the resident presented their case to the group, and the facilitator encouraged the other learners to ask questions and provide suggestions regarding further assessment and treatment. Residents were encouraged to continue attending discussion sessions throughout their residency training (i.e., to join discussion sessions of subsequent Be ExPeRT cohorts).

Conversion From In Vivo to Digital Platform Due to COVID-19

Initially, we held both the interactive seminar and the case discussions entirely in person, with the option of some residents calling into the case discussions if they were offsite for their rotation. Due to social distancing requirements related to the COVID-19 pandemic, we conducted the seminar and subsequent discussion sessions through digital-based conferences via Zoom beginning in March 2020. We utilized features of Zoom to engage participants, using breakout rooms to facilitate group discussion and implementing poll functionality and the group chat features to encourage active participation.

Research Assessment

We asked all participants who attended one of the four Be ExPeRT seminars from January 2019 to November 2020 at an academic medical center in the Southeast to complete surveys pre- and posttraining regarding their perceived knowledge about and comfort with assessment, diagnosis, and treatment of ADHD, anxiety, depression, and suicidality. Surveys developed by REACH (the Resource for Advancing Children's Health) were modified with permission for use with residents.[25] The surveys were administered using the Qualtrics platform. We emailed the presurvey (Appendix J) to all participants prior to the start of the training and gave them time during the half-day seminar to complete the postcourse survey (Appendix H).

This project was determined to be exempt from institutional review board approval. We described pre- and posttraining survey results with frequencies and percentages for the categorical variables. Differences between pre- and posttraining survey results were tested using Fisher's exact test. All analyses were performed using SPSS (IBM SPSS Statistics version 27).

Results

A total of 23 participants attended the four seminars, with approximately four to five participants attending each, except for

Copyright © 2023 Manning et al. This is an open-access publication distributed under the terms of the Creative Commons Attribution license.

the first one, which included nine participants. Participation in the monthly case sessions varied, and the mean number of sessions attended was 1.6. Of the 23 residents who participated in the seminars, more than half were pediatrics residents ($n = 15$), and about one-third were medicine-pediatrics combined residents ($n = 8$). Nearly three-quarters of participants completed the pre- and postsurveys (presurvey $n = 17$ [74%], postsurvey $n = 16$ [70%]).

Results from the pre- and postsurveys are listed in the Table. Prior to completing the Be ExPeRT seminar, nearly three-quarters of residents reported feeling knowledgeable about the assessment and diagnosis of each condition taught. A majority of residents indicated that they were comfortable with assessing and diagnosing major depressive disorder (65%), suicide risk (71%), and ADHD (82%), while only about half felt comfortable with assessing and diagnosing anxiety (47%), prior to program participation. Residents' knowledge about and comfort with treatment of the four conditions prior to participating in the seminar varied, with fewer residents reporting knowledge about and comfort with treatment of anxiety (35% and 24%, respectively) compared to the other conditions.

Results indicated that participation in the Be ExPeRT seminar significantly improved residents' knowledge about assessment and diagnosis (71% pre, 100% post, $p = .03$), as well as knowledge about and comfort with treatment of major depressive disorder (knowledge: 65% pre, 94% post, $p = .01$; comfort: 41% pre, 94% post, $p = .002$). Residents' knowledge about and comfort with treating suicide risk significantly improved from pre- to posttraining (knowledge: 53% pre, 94% post, $p = .01$; comfort: 29% pre, 94% post, $p < .001$). With regard to anxiety, a significant improvement was found when examining resident's knowledge about and comfort with assessment and diagnosis (knowledge: 71% pre, 100% post, $p = .03$; comfort: 47% pre, 88% post, $p = .02$), as well as knowledge about and comfort with treatment (knowledge: 35% pre, 94% post, $p < .001$; comfort: 24% pre, 94% post, $p < .001$) of this concern, following participation in the seminar. Notably, a majority of residents reported increased knowledge and comfort managing all domains of major depressive disorder, suicide risk, anxiety, and ADHD at posttraining. Following completion of the seminar, residents also demonstrated significant improvements in their approach to assessing and diagnosing as well as treating suicide risk, with a majority of residents indicating that they would assess and manage patients with a suicide risk (100% and 94%, respectively) rather than refer them to a specialist (Figure). Of the residents who completed the survey, three-quarters ($n = 12$) rated the training in the top 5%-10% with respect to other resident didactic or learning experiences.

Table. Residents' Knowledge and Comfort Regarding Assessment, Diagnosis, and Treatment of Pediatric B/MH Concerns

B/MH Concern	No. (%) Presurvey[a]	No. (%) Postsurvey[b]	p[c]
Major depressive disorder			
Knowledgeable about assessment and diagnosis	12 (71)	16 (100)	.03
Comfortable with assessment and diagnosis	11 (65)	15 (94)	.05
Knowledgeable about treatment[d]	11 (65)	15 (94)	.01
Comfortable with treatment	7 (41)	15 (94)	.002
Suicide risk			
Knowledgeable about assessment and diagnosis	13 (77)	15 (94)	.19
Comfortable with assessment and diagnosis	12 (71)	13 (81)	.38
Knowledgeable about treatment[d]	9 (53)	15 (94)	.01
Comfortable with treatment	5 (29)	15 (94)	<.001
Anxiety			
Knowledgeable about assessment and diagnosis	12 (71)	16 (100)	.03
Comfortable with assessment and diagnosis	8 (47)	14 (88)	.02
Knowledgeable about treatment[d]	6 (35)	15 (94)	<.001
Comfortable with treatment	4 (24)	15 (94)	<.001
Attention deficit hyperactivity disorder			
Knowledgeable about assessment and diagnosis	14 (82)	16 (100)	.13
Comfortable with assessment and diagnosis	14 (82)	15 (94)	.32
Knowledgeable about treatment	13 (77)	15 (94)	.07
Comfortable with treatment	13 (77)	15 (94)	.19

Abbreviation: B/MH, behavioral or mental health.
[a]Seventeen participants completed the presurvey.
[b]Sixteen participants completed the postsurvey.
[c]These p values are based on results from Fisher's exact test. They are unadjusted comparisons of ratings on the pre- versus postsurvey. Residents were considered to have perceived knowledge and comfort if they answered an item with "moderate amount" or "great deal."
[d]One resident did not complete these items on the postsurvey.

Copyright © 2023 Manning et al. This is an open-access publication distributed under the terms of the Creative Commons Attribution license.

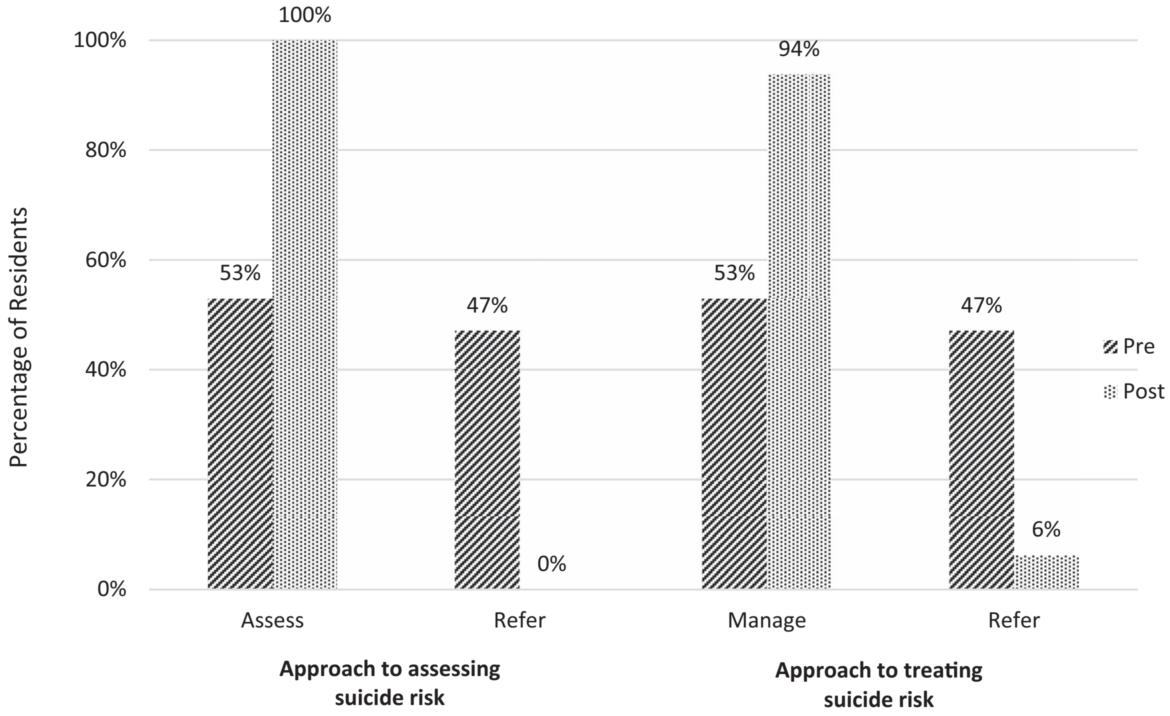

Figure. Residents' approach to assessing and treating suicide risk. Residents were considered to assess suicide risk if they indicated that they would assess and diagnose all cases, most cases, or only less complicated cases. For treating suicide risk, residents were considered to manage this concern if they indicated that they would manage all cases with no consultation with a mental health colleague, most cases with occasional consultation with a mental health colleague, or only less complicated cases. Residents were considered to refer patients with a suicide risk for assessment or treatment if they indicated they would refer most cases. For both items, the difference in approach from pre- to postsurvey was significant ($p < .05$). One resident did not complete the item about approach to assessing suicide risk.

Discussion

Effective B/MH education is needed to meet the demands for B/MH services in primary care and to address gaps in training. The implementation of this B/MH training program was found to be feasible in the context of a residency program and effective in imparting information to residents. The Be ExPeRT program was designed to incorporate active participation to increase retention of the material. The program was found to be acceptable to participants and led to improvement in residents' comfort in and knowledge of common B/MH conditions. Of note, a majority of residents rated themselves knowledgeable about and comfortable with the assessment and diagnosis of suicide risk, as well as the assessment, diagnosis, and treatment of ADHD, prior to receiving the training. These ratings may have been influenced by year of training, desirability bias, base rate of ADHD, or comfort of their supervisors in handling ADHD in their clinic settings.

This program has several limitations. Not all participants completed the pre- and postsurveys, which should be considered when interpreting the results, although we did have a fairly high response rate of around 70%. Additionally, the sample size was small, and a majority of participants were female; thus, results may not be generalizable to other programs. The survey questions we included to understand program efficacy are based on residents' perceived behavior and attitudes and do not measure actual practice behaviors. The gold standard for evaluating curriculum implementation is the assessment of practice change (e.g., use of screening tools, initiation of medications to treat B/MH conditions). To support this evaluation, it would be useful to examine participants' B/MH practice behaviors before and after completion of the Be ExPeRT program. Additionally, the opt-in nature of the program may have resulted in self-selection by the most interested and motivated residents. We expect that implementation on a larger scale, for example, mandatory participation within a residency program, would reach participants with lower baseline B/MH knowledge and comfort and result in greater gains following participation.

Despite the self-selected group, attendance at the monthly discussion sessions was suboptimal. Other programs could increase attendance by offering protected time for all trainees to participate and the option to earn a program certificate for attending a predetermined percentage of case discussion

Copyright © 2023 Manning et al. This is an open-access publication distributed under the terms of the Creative Commons Attribution license.

sessions. Also, we did not assess whether participation in case discussion sessions or quantity of sessions attended affected participants' comfort with or knowledge of mental health conditions. Another limitation is the potential burden on faculty facilitators. The training has been delivered with a full-time attending CAP and two child and adolescent psychiatry fellows as well as with two attending CAPs and an attending pediatrician. Allowing fellows or chief residents to teach could facilitate greater feasibility as well as provide an excellent opportunity for trainees to gain valuable teaching skills. It is unclear if the required length or modality of the training is necessary to achieve the reported results or simply if the time spent with child and adolescent psychiatry faculty and fellows affected resident comfort/confidence. Given the demands on faculty facilitators and concerns about sustainability, it will be important to examine potential modifications that could be made to the training, such as shortening its length or delivering content asynchronously, while maintaining its effectiveness. Although participants rated the seminar in the top 5%-10% of teaching activities, it would be beneficial to evaluate fidelity in future seminars to ensure that all instructors are delivering the same content and that participants are gaining similar knowledge and experience.

Future efforts at our institution will aim to increase participation, improve assessment of practice change, and gather additional implementation data to make the curriculum more easily replicable. We also plan to standardize B/MH competency through the use of a skills passport (similar to a procedure log). Other future considerations include developing recorded videos for dissemination as well as increasing the availability of professional development activities for faculty preceptors. This additional work would aim to increase faculty comfort level in supervising B/MH diagnosis and treatment, as this is sometimes a barrier to residents' ability to implement practice change in clinical settings. In addition, while this program was specifically developed for pediatric residents, modified versions have been given to family medicine residents and physician assistant students. The curriculum could also easily be expanded to any group of pediatric primary care clinicians.

In conclusion, there is a compelling need to enhance pediatric trainee education in addressing B/MH issues. Implementation of the Be ExPeRT curriculum improved residents' knowledge of and comfort with treatment of common pediatric B/MH conditions. The curriculum was found to be acceptable and feasible and could be easily generalized to other types of training programs across the country.

Appendices

A. Facilitator Guide.docx
B. Be ExPeRT Introduction.pptx
C. ADHD in Primary Care Pediatrics.pptx
D. Anxiety in Primary Care Pediatrics.pptx
E. Depression in Primary Care Pediatrics.pptx
F. Be ExPeRT Reference Slides.pptx
G. Participant Guide.docx
H. Be ExPeRT Postsurvey.docx
I. Be ExPeRT Case Discussion Form.docx
J. Be ExPeRT Presurvey.docx

All appendices are peer reviewed as integral parts of the Original Publication.

Alison Manning, MD: Assistant Professor and Clinician Educator, Department of Psychiatry and Human Behavior, The Warren Alpert Medical School of Brown University

Matthew Weingard, MD: Assistant Professor, Division of Pediatric Behavioral Health, Department of Pediatrics, University of Utah and Intermountain Primary Children's Hospital

Jacqueline Fabricius, MD: Pediatric Rheumatology Fellow, Department of Pediatrics, Northwestern University Feinberg School of Medicine

Alexis French, PhD: Medical Instructor, Division of Child & Family Mental Health & Community Psychiatry, Department of Psychiatry & Behavioral Sciences, Duke University School of Medicine

Mya Sendak, MD: Consulting Associate, Department of Pediatrics, Duke University School of Medicine

Naomi Davis, PhD: Assistant Professor, Division of Child & Family Mental Health & Community Psychiatry, Department of Psychiatry & Behavioral Sciences, Duke University School of Medicine

Acknowledgments
The authors would like to thank Chelsea Swanson, MPH, for her role in coordinating the experiential training, and Amanda Kimberg, MD, for her help with facilitating the training and reviewing the manuscript.

Disclosures
None to report.

Funding/Support
Funding for this work was provided by a grant from the Health Resources and Services Administration.

Prior Presentations
Fabricius J, Manning A. Be ExPeRT: Behavioral Health Expansion in Pediatric Residency Training: successful collaborative curriculum with pediatric psychiatrist. Poster presented at: Association of Pediatric Program Directors Annual Meeting; April 2, 2020; San Diego, CA.

Copyright © 2023 Manning et al. This is an open-access publication distributed under the terms of the Creative Commons Attribution license.

Ethical Approval
The Duke University Institutional Review Board deemed further review of this project not necessary.

Disclaimer
The project sponsor played no role in the study design, data collection, analysis, interpretation of data, writing, or revision of the manuscript.

References

1. Whitney DG, Peterson MD. US national and state-level prevalence of mental health disorders and disparities of mental health care use in children. *JAMA Pediatr*. 2019;173(4):389-391. https://doi.org/10.1001/jamapediatrics.2018.5399
2. Hunter CL, Goodie JL, Oordt MS, Dobmeyer AC. *Integrated Behavioral Health in Primary Care: Step-by-Step Guidance for Assessment and Intervention.* American Psychological Association; 2009.
3. American Academy of Pediatrics; American Academy of Child and Adolescent Psychiatry; Children's Hospital Association. AAP-AACAP-CHA declaration of a national emergency in child and adolescent mental health. American Academy of Pediatrics. Updated October 19, 2021. Accessed May 18, 2023. https://www.aap.org/en/advocacy/child-and-adolescent-healthy-mental-development/aap-aacap-cha-declaration-of-a-national-emergency-in-child-and-adolescent-mental-health/
4. Green C, Leyenaar JK, Turner AL, Leslie LK. Competency of future pediatricians caring for children with behavioral and mental health problems. *Pediatrics*. 2020;146(1):e20192884. https://doi.org/10.1542/peds.2019-2884
5. Fox HB, McManus MA, Klein JD, et al. Adolescent medicine training in pediatric residency programs. *Pediatrics*. 2010;125(1):165-172. https://doi.org/10.1542/peds.2008-3740
6. Committee on Psychosocial Aspects of Child and Family Health; Task Force on Mental Health. Policy statement—the future of pediatrics: mental health competencies for pediatric primary care. *Pediatrics*. 2009;124(1):410-421. https://doi.org/10.1542/peds.2009-1061
7. Green CM, Foy JM, Earls MF; Committee on Psychosocial Aspects of Child and Family Health; Mental Health Leadership Work Group. Achieving the pediatric mental health competencies. *Pediatrics*. 2019;144(5):e20192758. https://doi.org/10.1542/peds.2019-2758
8. McMillan JA, Land M Jr, Leslie LK. Pediatric residency education and the behavioral and mental health crisis: a call to action. *Pediatrics*. 2017;139(1):e20162141. https://doi.org/10.1542/peds.2016-2141
9. Foy JM, Green CM, Earls MF; Committee on Psychosocial Aspects of Child and Family Health; Mental Health Leadership Work Group. Mental health competencies for pediatric practice. *Pediatrics*. 2019;144(5):e20192757. https://doi.org/10.1542/peds.2019-2757
10. Rosenberg AA, Kamin C, Glicken AD, Jones MD Jr. Training gaps for pediatric residents planning a career in primary care: a qualitative and quantitative study. *J Grad Med Educ*. 2011;3(3):309-314. https://doi.org/10.4300/JGME-D-10-00151.1
11. Moscardini EH, Hill RM, Dodd CG, Do C, Kaplow JB, Tucker RP. Suicide safety planning: clinician training, comfort, and safety plan utilization. *Int J Environ Res Public Health*. 2020;17(18):6444. https://doi.org/10.3390/ijerph17186444
12. Kemper AR, Hostutler CA, Beck K, Fontanella CA, Bridge JA. Depression and suicide-risk screening results in pediatric primary care. *Pediatrics*. 2021;148(1):e2021049999. https://doi.org/10.1542/peds.2021-049999
13. Goldenberg M, Penaskovic K. Suicide risk assessment team-based learning (TBL) module. *MedEdPORTAL*. 2012;8:9088. https://doi.org/10.15766/mep_2374-8265.9088
14. Biddle VS. Online adolescent suicide risk assessment. *MedEdPORTAL*. 2013;9:9352. https://doi.org/10.15766/mep_2374-8265.9352
15. Stanley A, Chelvakumar G, Cody P, et al. Resident training curriculum in adolescent depression and suicide screening. *MedEdPORTAL*. 2016;12:10361. https://doi.org/10.15766/mep_2374-8265.10361
16. Mian ND, Pincus DB, Perrin EC, Bair-Merritt M. Identifying and making recommendations for pediatric anxiety disorders in primary care settings: a video-based training. *MedEdPORTAL*. 2020;16:11033. https://doi.org/10.15766/mep_2374-8265.11033
17. Chokshi B, Chen KLD, Beers L. Interactive case-based childhood adversity and trauma-informed care electronic modules for pediatric primary care. *MedEdPORTAL*. 2020;16:10990. https://doi.org/10.15766/mep_2374-8265.10990
18. Schmitz A, Light S, Barry C, Hodges K. Adverse childhood experiences and trauma-informed care: an online module for pediatricians. *MedEdPORTAL*. 2019;15:10851. https://doi.org/10.15766/mep_2374-8265.10851
19. Ufomata E, Eckstrand KL, Spagnoletti C, et al. Comprehensive curriculum for internal medicine residents on primary care of patients identifying as lesbian, gay, bisexual, or transgender. *MedEdPORTAL*. 2020;16:10875. https://doi.org/10.15766/mep_2374-8265.10875
20. Lebrun-Harris LA, Ghandour RM, Kogan MD, Warren MD. Five-year trends in US children's health and well-being, 2016-2020. *JAMA Pediatr*. 2022;176(7):e220056. https://doi.org/10.1001/jamapediatrics.2022.0056
21. Data and statistics on children's mental health. Centers for Disease Control and Prevention. Updated March 8, 2023. Accessed May 18, 2023. https://www.cdc.gov/childrensmentalhealth/data.html

Copyright © 2023 Manning et al. This is an open-access publication distributed under the terms of the Creative Commons Attribution license.

22. Kolb DA. *Experiential Learning: Experience as the Source of Learning and Development*. Prentice-Hall; 1983.

23. Arora S, Kalishman SG, Thornton KA, et al. Project ECHO: a telementoring network model for continuing professional development. *J Contin Educ Health Prof*. 2017;37(4):239-244.

24. Wolraich ML, Feurer ID, Hannah JN, Baumgaertel A, Pinnock TY. Obtaining systematic teacher reports of disruptive behavior disorders utilizing DSM-IV. *J Abnorm Child Psychol.* 1998;26(2):141-152. https://doi.org/10.1023/A:1022673906401

25. REACH Institute. Accessed May 18, 2023. https://thereachinstitute.org/

Received: September 11, 2022
Accepted: April 8, 2023
Published: August 1, 2023

Copyright © 2023 Manning et al. This is an open-access publication distributed under the terms of the Creative Commons Attribution license.
